# Systematic Identification of Survival-Associated Alternative Splicing Events in Kidney Renal Clear Cell Carcinoma

**DOI:** 10.1155/2021/5576933

**Published:** 2021-04-19

**Authors:** Yubin Wei, Zheng Zhang, Rui Peng, Yan Sun, Luyu Zhang, Handeng Liu

**Affiliations:** ^1^Laboratory of Tissue and Cell Biology, Experimental Teaching and Management Center, Chongqing Medical University, Chongqing, China; ^2^Department of Molecular Medicine and Cancer Research Center, Chongqing Medical University, Chongqing, China; ^3^Department of Bioinformatics, Chongqing Medical University, Chongqing, China

## Abstract

There is growing evidence that aberrant alternative splicing (AS) is highly correlated with driving tumorigenesis, but its function in kidney renal clear cell carcinoma (KIRC) remains to be discovered. In this study, we obtained the level-3 RNA sequencing and clinical data of KIRC from The Cancer Genome Atlas (TGCA). Combining with the splicing event detail information from TGCA SpliceSeq database, we established the independent prognosis signatures for KIRC with the univariate and multivariate Cox regression analyses. Then, we used the Kaplan-Meier analysis and receiver operating characteristic curves (ROCs) to assess the accuracy of prognosis signatures. We also constructed the regulatory network of splicing factors (SFs) and AS events. Our results showed that a total of 12029 survival-associated AS events of 5761 genes were found in 524 KIRC patients. All types of prognosis signatures displayed a satisfactory ability to reliably predict, especially in exon skip model which the area under curve of ROC was 0.802. Moreover, 18 splicing factors (SFs) highly correlated to AS events were identified. With the construction of the SF-AS interactive network, we found that SF powerfully promotes the occurrence of abnormal AS and may have a profound role in KIRC. Collectively, we screened survival-associated AS events and established prognosis signatures for KIRC, coupling with the SF-AS interactive network, which might provide a key perspective to clarify the potential mechanism of AS in KIRC.

## 1. Introduction

Renal cell carcinoma is the most comment histologic type of kidney cancer and about 70% are kidney renal clear cell carcinoma (KIRC) [1, 2]. As one of the aggressive cancers, KIRC has a higher transfer and relapse rate compared with other subtypes of RCC and accounts for most cancer-related deaths. Due to the therapeutic resistance, more effective therapies with prolonging survival time or a higher survival rate are required in the frontline treatment of KIRC [3].

**Figure 1:**
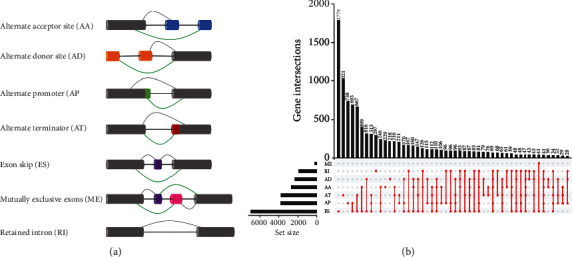
(a) Seven types of alternative splicing. (b) An Upset plot showing all the alternative splicing events in KIRC. Alternative splicing is common in KIRC. AA: alternate acceptor site; AD: alternative donor site; AP: alternate promoter; AT: alternative terminator; ES: exon skip; ME: mutually exclusive exon; RI: retained intron.

The alternative splicing (AS) of pre-RNA is the key means and critical step in the gene expression regulation [4, 5]. It is estimated that more than 98% of human genes generate multiple proteins through alternative splicing of pre-RNA [6]. It is conducive to generating genetic diversity and tissue specificity [7–9]. There are seven types of AS events (Figure 1(a)) [10], including alternate acceptor site (AA), alternative donor site (AD), alternate promoter (AP), alternative terminator (AT), exon skip (ES), mutually exclusive exons (MEs), and retained intron (RI). In cancer, the functional and nonfunctional final products come along with the disorder of alternative splicing [11]. And the aberrant AS may have a direct effect on the protein domain families which are mutated in the tumor, so it can probably cause the disruption of the protein-protein interaction in the cancer-related pathways [12]. An increasing number of studies focused on the potential regulatory mechanisms between tumors and ASs. For example, the splicing of FOX2 was observed to be changed in breast cancer that could regulate cancer cell proliferation [13]. Besides, the SVH-B is a specific splicing variant of armadillo repeat containing 10 (ARMC10, also known as SVH) overexpressing in hepatocellular carcinoma, and the cancer cells could obtain higher growth rate and high tumorigenicity as a result [14]. Nevertheless, there remains a lack of research on AS in KIRC, and intensive studies on exploring prognosis AS models in KIRC are necessary.

In addition, the splicing factors (SFs) can coregulate gene expression to drive cancer progression [15]. Previously, some studies showed that the deregulated expression of SFs can be found in cancer, such as the splicing factor SF2/ASF which is a puissant protooncogene that affects the regulation of alternative splicing of the vital target gene in various diseases [16], and the splicing factor SRSF10 is significantly upregulated in HPV16/18-positive cervical cancer and pivotal for the tumorigenic ability [17]. As we knew, there have not enough reports on the function of SF in KIRC. Hence, the detailed mechanism of SF to interact with AS that drives KIRC requires further research.

Consequently, in this study, we excavated the splicing data of KIRC available in TCGA database to screen the survival-associated AS events and structured the prognostic signatures. We also constructed the SF-AS network to investigate the potential regulated mechanism so that we may discover a new perspective of targeted therapy of KIRC. The work will provide a help to search for the molecular targets for the diagnosis and treatment of KIRC.

## 2. Materials and Methods

### 2.1. Collection of the Raw Data and the Clinical Data of KIRC

KIRC level-3 RNA sequencing data is available in The Cancer Genome Atlas (TGCA, http://tgca-data.nci.nih.gov/tgca/) [18]. The clinical information of KIRC can also be obtained from it. And the patients with an overall survival less than 90 days were excluded from the study in order to eliminate the influence of death not caused by KIRC. A total of 524 patients were included in our study.

The corresponding data for each AS event of KIRC were downloaded from TGCA SpliceSeq (http://bioinformatics.mdanderson.org/TCGASpliceSeq). The percent splicing in (PSI) values of the AS events for KIRC had been loaded into TCGA SpliceSeq and was applied to quantify the AS events which ranging from 0 to 1 [19, 20]. The percentage of samples with PSI > 75% and a standard deviation > 0.1 were set as the screening criteria to ensure the accuracy of the results.

### 2.2. Identification of Prognostic Signatures of AS Event

The univariate Cox regression analysis was used to identify survival-related AS events for each type of AS (*p* < 0.05). Also, the R software (Version 3.5.3) with the UpsetR package was carried out to visualize the multiple interactive sets of seven types of survival-associated AS events. Then, a tenfold cross-validation penalized least absolute shrinkage and selection operator (LASSO) logistic regression was used to select the survival-associated AS events as prognostic signatures. These AS events were included in the multivariate Cox regression model to construct the independent prognosis signature for KIRC. By using the formula: risk score = ∑_*i*_^*n*^PSI*i* × *iβ* (*β* was the regression coefficient from the multivariate Cox regression analysis), the risk score of each selected survival-associated AS event was obtained. The media value of the risk score was used as the cutoff threshold to divide the patients into high-risk and low-risk groups (Table S1-7). Further, the Kaplan-Meier analysis [21] and receiver operating characteristic (ROC) [22, 23] curves were employed to evaluate the predictive accuracy of the prognosis signatures.

### 2.3. Construction of SF-AS Regulatory Network

To explore the mutual regulation mechanisms of AS events and SF, we obtained the information of SF genes from the SpliceAid 2 database (http://www.introni.it/spliceaid.html, Table S8) to build a regulatory network. Correlations between SFs and survival-associated AS events were evaluated by using Pearson correlation analysis with the criteria: the absolute value of correlation coefficient > 0.8, *p* < 0.001. Additionally, the SF-AS correlation network was visualized by Cytoscape (Version 3.7.1).

## 3. Results

### 3.1. Alternative Splicing Events in KIRC

Overall, 524 KIRC patients with 72 non-KIRC patients were included in our study. The clinical information of KIRC patients were integrated in Table S1.

In total, 46415 AS events for KIRC patients were detected in 10601 genes, which contained 3821 AAs in 2863 genes, 3270 ADs in 2300 genes, 8632 ATs in 3770 genes, 9509 APs in 3805 genes, 18117 ESs in 6915 genes, 235 MEs in 227 genes, and 2831 RIs in 1902 genes by screening the AS events of KIRC (Figure 1(b)). Interestingly, the ES event was the most frequent AS events accounting for about 39%, while the ME event was rare among AS events of KIRC only accounting for about 0.5%. A gene corresponds to three AS events averagely suggested that a single gene may undergo multiple times of splicing.

### 3.2. Screening of Survival-Associated AS Events

**Figure 2:**
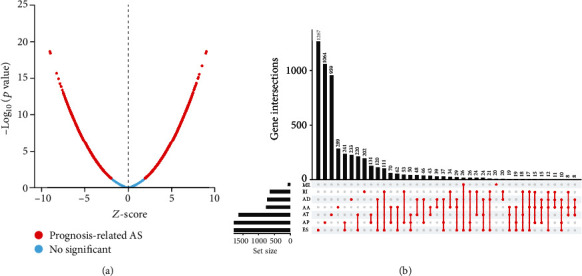
Survival-associated AS events in KIRC. (a) The red dots denote the AS events that are prognosis-related, and the blue dots denote nonsignificance. The AS events happened in KIRC were not all related to the occurrence of KIRC, and some of them were closely related to overall survival. (b) Upset plot of different types of survival-associated AS events. AS: alternative splicing; KIRC: kidney renal clear cell carcinoma.

**Figure 3:**
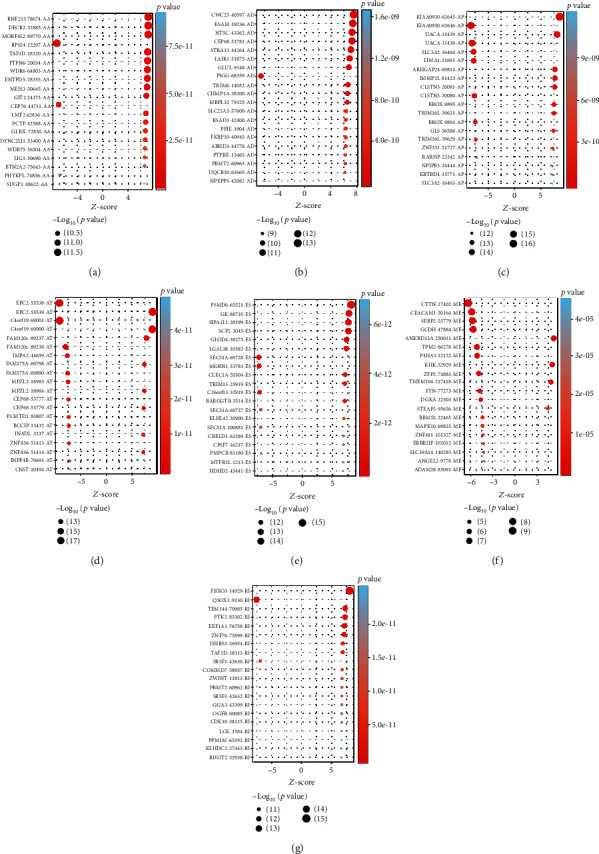
Top 20 survival-associated AS events in each type of AS events. (a) Alternate acceptor site; (b) alternative donor site; (c) alternate promoter; (d) alternative terminator; (e) exon skip; (f) mutually exclusive exons; (g) retained intron. AS: alternative splicing.

The Cox regression analysis and the median value of the PSI value were used to investigate survival-associated AS events (*p* < 0.05) (Figure 2(a)). In general, we found 12029 survival-associated AS events in 5761 genes, including 823 AAs in 718 genes, 710 ADs in 623 genes, 2675 ATs in 1615 genes, 3242 APs in 1851 genes, 3532 ESs in 2393 genes, 71 MEs in 71 genes, and 976 RIs in 743 genes (Figure 2(b)). The top 20 survival-related AS events in each type of AS event are presented in Figures 3(a)–3(g). These results indicate that AS events that happened in KIRC cases were not all related to the occurrence of KIRC, and some of them were closely related to overall survival.

### 3.3. Establishment of Prognosis Signatures of KIRP-Related AS Events

**Figure 4:**
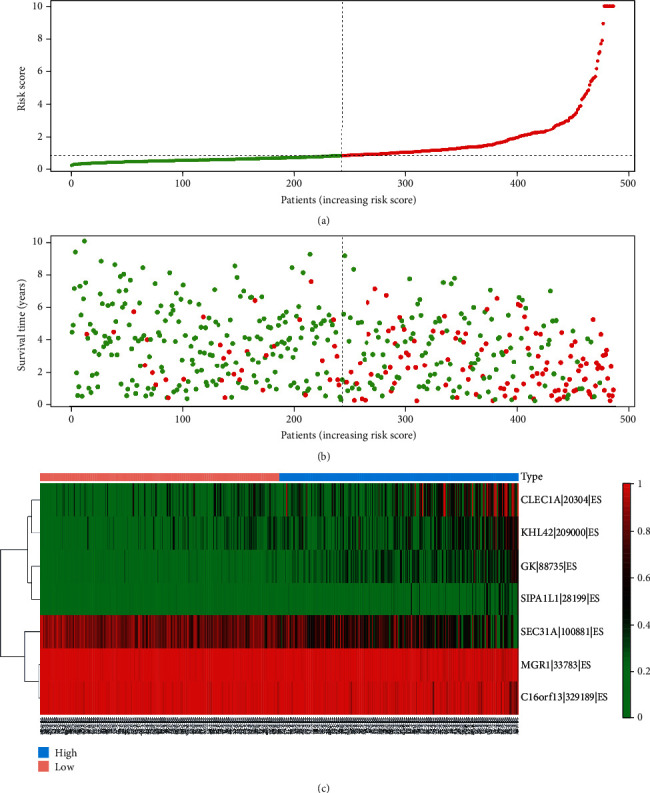
Details of prognosis signatures of ES. (a) The risk scores of KIRC patients' distribution basing on the median value. (b) The green dots mean survivors, and the red dots mean death cases. (c) The heat map shows the alteration of the percent spliced in value from low risk score to high risk score. The prognosis signature of ES has excellent capacity on prognosis of KIRC patients. ES: exon skip; KIRC: kidney renal clear cell carcinoma.

**Figure 5:**
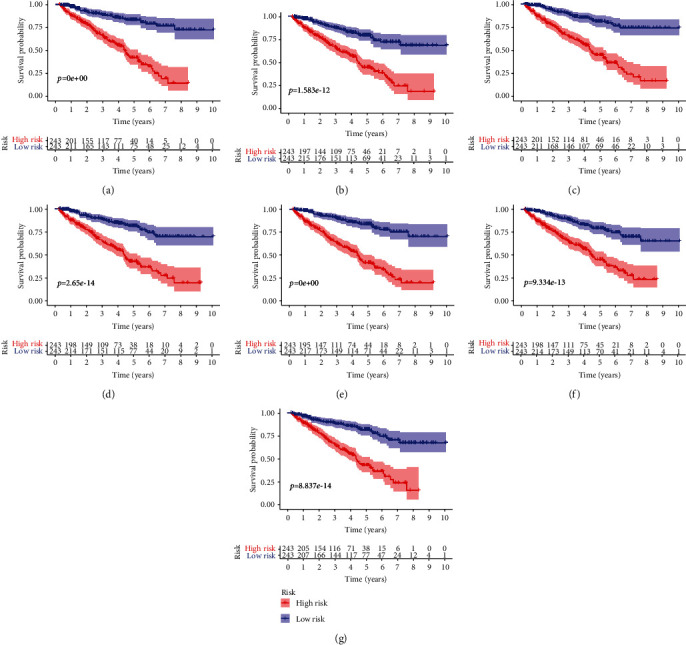
Kaplan-Meier curves of every type of prognosis signatures for each type of AS events. The red line means high-risk group, and blue line means low-risk group. The low-risk groups have a better ten-year survivorship than high-risk group in all types. (a) Alternate acceptor site; (b) alternative donor site; (c) alternate promoter; (d) alternative terminator; (e) exon skip; (f) mutually exclusive exons; (g) retained intron. AS: alternative splicing.

**Figure 6:**
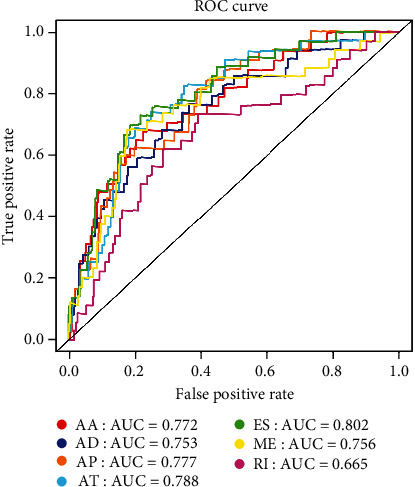
The ROC curve of every type of prognosis signatures for each type of AS events. All models have a satisfactory prognosis predicting efficiency, especially the ME, AT, and AP models. ROC: receiver operating characteristic; AS: alternative splicing; AA: alternate acceptor site; AD: alternative donor site; AP: alternate promoter; AT: alternative terminator; ES: exon skip; ME: mutually exclusive exon; RI: retained intron; AUC: the areas under the curve.

For the reliability of the prognosis signatures and avoiding the danger of overfitting, we conducted the LASSO regression in the top 20 AS events among seven types to select the survival-related AS events as prognosis signatures (Figure S1A-G). 40 AS events were screened out the risk scores increasing (Table S10), and the PSI value of the survival-associated AS events in KIRC changed significantly (Figure 4 and Figure S2-7). So, we can find that abnormal AS had a powerful capacity on prognosis of KIRC patients. By coupling with the Kaplan-Meier analysis, we could distinguish the poor or favorable overcome between the two groups in seven types of AS events. And the results reflect that low-risk groups have a better ten-year survivorship than high-risk group in all types (Figure 5). The ROC curve was used to evaluate the efficiency of predictive ability of every prognostic signature. The area under the curve (AUC) values of AA, AD, AP, AT, ES, ME, and RI were 0.772, 0.753, 0.777, 0.788, 0.802, 0.756, and 0.665, respectively (Figure 6). The larger the AUC value corresponds to the better classified ability of the prognosis signatures. So, our results indicate that the prognostic signatures of ME, AT, and AP have a considerable prognosis predicting efficiency to evaluate the efficiency of predictive ability of every prognostic signature.

### 3.4. Network of SF-AS

**Figure 7:**
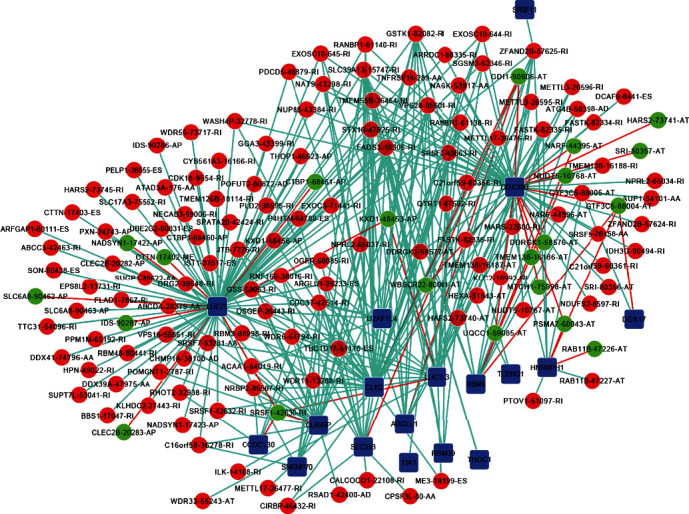
SF-AS regulatory network in KIRC. 18 splicing factors (blue square) were screened out aggregately. The red dots denote AS events with poor predictions, and the green dots denote AS events with good predictions. The red line means a negative regulatory relationship between SF and AS, and the blue line means a positive regulatory relationship between SF and AS. The AS events related to the poor predictions are positive correlations with SFs, and AS events related to favorable prediction are negative correlations. AS: alternative splicing; SF: splicing factor; KIRC: kidney renal clear cell carcinoma.

In order to better interpret the complex regulatory architecture of SF and AS events, we constructed a SF-AS interactive network. In addition, the Pearson correlation analysis was conducted to investigate the correlation between prognosis AS events and the differential expression of survival-related SF. We obtained 18 SFs and 143 survival-associated AS events (Figure 7). As depicted in the network, AS events associated with the poor prognosis are positive correlations with SFs; however, AS events associated with favorable prognosis are negative correlations. This revealed that SFs could negatively regulate the occurrence of favorable survival-associated AS events and enhance the expression of AS events related to worse prognosis; the result may be expected to contribute to the development of KIRC.

## 4. Discussion

AS is a prevalent mechanism for gene expression that lets single mRNA produce multiple different mRNAs [24]. Under normal physiological conditions, a variety of protein isoforms are generated by AS that can enrich biological functions. In some pathological situations, however, aberrant AS creates unusual protein isoforms that can even antagonize normal proteins and eliminate their physiological functions [25]. Anomalous AS could bring about activation of oncogenes and the inhibition of tumor suppressor genes [26]. Hence, aberrant AS is relevant to various diseases, including cancer [27]. The tumor cells present notable changes in the transcriptome by tumor-specific splicing isoforms. The progression of the tumor could be driven by these isoforms and their encoded proteins [28].

Nowadays, with the rapid development of technology, advancements in high-throughput sequencing that was employed in the analysis of transcriptomes greatly improved our grasp of AS. Obviously, AS plays an essential role in KIRC. Nevertheless, there is still a lack of research on abnormal AS in KIRC. Consequently, it is urgent to identify new biomarkers to apply in the recognition and treatment of KIRC. In our research, 46415 AS events in 10601 genes were detected in KIRC, explaining that AS is ubiquitous in KIRC. For the survival-associated AS events, we identified 12029 AS events in 5761 genes, which ES accounts for the most that is consistent with the previous study [29], suggesting that the dysregulation of ES plays an important role in the occurrence of KIRC. Next, we employed univariate and multivariate Cox analyses to build the prognosis signatures for investigating the prognostic ability of survival-associated AS events in KIRC. Coupling with the Kaplan-Meier and ROC analyses, those results showing that the prognosis signatures have excellent accuracy for predicting the prognosis and survival rate in KIRC. Also, the ES model has the most excellent predictive power with important clinical significance, in which the AUC is 0.802. Collectively, those findings may provide a new perspective of accurate individualized treatment in KIRC.

SF participates in the process of RNA modulation, and the common coregulated function of splicing factors almost appears in all major cancer types [15, 30]. The differential expression of critical SF may be involved in the dysregulation of splicing that could drive tumorigenesis [31, 32]. So, a comprehensive view of AS in KIRC will approach one step further by constructing the SF-AS interactive network. In this network, positive regulations between AS events and SFs are quite common in which 289 events had been detected. Notably, those positively regulated AS events by SFs were almost related to poor prognosis while negatively regulated AS events were related to favorable prognosis. Therefore, it is believed that SF is clearly a driving factor by disturbing normal AS in KIRC. Although the 18 SFs we screened out were highly correlated with KIRC, none of them have been studied in KIRC as we knew. Some of the SFs have been studied in other diseases that may explain these SFs' contribution in KIRC functionally. For example, ARGLU1 is a protein interacting with MED1 required for breast cancer cell growth [33]. Moreover, cell metabolism, growth, and survival are regulated by Akt, and its abnormal activity can cause tumors; as a downstream target of Akt signaling, CLK2 could be a therapeutic target of the Akt-driven tumors [34, 35]. Also, the growth of breast cancer cells could be suppressed under the downregulation of CLK2 [36]. DDX39B is necessary for cell growth and proliferation and could promote proliferation and colony formation. Its dysregulation can enhance tumorigenic ability [37]. Again, HNRNPH1 is required for the growth and survival of rhabdomyosarcoma cells [38], and the synergistic transcription of *U2AF1L4* with *PSENEN* is essential for the regulation of T-cell activity [39]. Therefore, we have reason to believe that those SFs could also promote the development of KIRC though most SFs have not been systematically and exhaustively studied in KIRC or other diseases. So, additional studies are needed to find out their specific functions in KIRC.

## 5. Conclusions

In short, we have conducted a comprehensive study on AS events of KIRC and established prognosis signatures of AS with high clinical predictive value. Through the AS-SF network, we have detected the SFs that can be a critical role in the process of KIRC. These findings enrich our understanding of the AS event function in KIRC and may reveal the potential mechanism to provide new targeted therapies.

## Data Availability

Data are available in supplementary information files.

## Supplementary Materials

Supplementary Materials Figure S1: the tenfold cross-validation penalized LASSO logistic regression for AA. LASSO: least absolute shrinkage and selection operator; AA: alternate acceptor site. Figure S2: the tenfold cross-validation penalized LASSO logistic regression for AD. LASSO: least absolute shrinkage and selection operator; AD: alternative donor site. Figure S3: the tenfold cross-validation penalized LASSO logistic regression for AP. LASSO: least absolute shrinkage and selection operator; AP: alternate promoter. Figure S4: The tenfold cross-validation penalized LASSO logistic regression for AT. LASSO: least absolute shrinkage and selection operator; AT: alternative terminator. Figure S5: the tenfold cross-validation penalized LASSO logistic regression for ES. LASSO: least absolute shrinkage and selection operator; ES: exon skip. Figure S6: the tenfold cross-validation penalized LASSO logistic regression for ME. LASSO: least absolute shrinkage and selection operator; ME: mutually exclusive exon. Figure S7: the tenfold cross-validation penalized LASSO logistic regression for RI. LASSO: least absolute shrinkage and selection operator; RI: retained intron exons. Figure S8: details of prognosis signatures of AA. (A) The risk scores of KIRC patients' distribution basing on the median value. (B) The green dots mean survivors, and the red dots mean death cases. (C) The heat map shows the alteration of the percent spliced in value from low risk score to high risk score. AA: alternate acceptor site; KIRC: kidney renal clear cell carcinoma. Figure S9: details of prognosis signatures of AD. (A) The risk scores of KIRC patients' distribution basing on the median value. (B) The green dots mean survivors, and the red dots mean death cases. (C) The heat map shows the alteration of the percent spliced in value from low risk score to high risk score. AD: alternative donor site; KIRC: kidney renal clear cell carcinoma. Figure S10: details of prognosis signatures of AP. (A) The risk scores of KIRC patients' distribution basing on the median value. (B) The green dots mean survivors, and the red dots mean death cases. (C) The heat map shows the alteration of the percent spliced in value from low risk score to high risk score. AP: alternate promoter; KIRC: kidney renal clear cell carcinoma. Figure S11: details of prognosis signatures of AT. (A) The risk scores of KIRC patients' distribution basing on the median value. (B) The green dots mean survivors, and the red dots mean death cases. (C) The heat map shows the alteration of the percent spliced in value from low risk score to high risk score. AT: alternative terminator; KIRC: kidney renal clear cell carcinoma. Figure S12: details of prognosis signatures of ME. (A) The risk scores of KIRC patients' distribution basing on the median value. (B) The green dots mean survivors, and the red dots mean death cases. (C) The heat map shows the alteration of the percent spliced in value from low risk score to high risk score. ME: mutually exclusive exon; KIRC: kidney renal clear cell carcinoma. Figure S13: details of prognosis signatures of RI. (A) The risk scores of KIRC patients' distribution basing on the median value. (B) The green dots mean survivors, and the red dots mean death cases. (C) The heat map shows the alteration of the percent spliced in value from low risk score to high risk score. RI: retained intron; KIRC: kidney renal clear cell carcinoma. Table S1: the clinical information of the KIRC patients. In survival time, 0 means death case and 1 means the patients alive. T: tumor; M: metastasis; N: lymph node. Table S2: the list of splicing factor genes collected from the SpliceAid 2 database. Table S3: result of the LASSO logistic regression with tenfold cross-validation to select the survival-associated AS events as prognostic signatures. LASSO: the least absolute shrinkage and selection operator; Coef: coefficient; HR: hazard ratio; HR 95: hazard ratio 95% confidence interval; L: low; H: high. Table S4: the risk score of KIRC patients in AA prognosis signatures. AA: alternate acceptor site; KIRC: kidney renal clear cell carcinoma. Table S5: the risk score of KIRC patients in AD prognosis signatures. AD: alternative donor site; KIRC: kidney renal clear cell carcinoma. Table S6: the risk score of KIRC patients in AP prognosis signatures. AP: alternate promoter; KIRC: kidney renal clear cell carcinoma. Table S7: the risk score of KIRC patients in AT prognosis signatures. AT: alternative terminator; KIRC: kidney renal clear cell carcinoma. Table S8: the risk score of KIRC patients in ES prognosis signatures. ES: exon skip; KIRC: kidney renal clear cell carcinoma. Table S9: the risk score of KIRC patients in ME prognosis signatures. ME: mutually exclusive exon; KIRC: kidney renal clear cell carcinoma. Table S10: the risk score of KIRC patients in RI prognosis signatures. RI: retained intron; KIRC: kidney renal clear cell carcinoma.
