# Supplementary material for: Systematic Identification of Survival-Associated Alternative Splicing Events in Kidney Renal Clear Cell Carcinoma
Source: Comput Math Methods Med. 2021 Apr 19;2021:5576933. doi: 10.1155/2021/5576933 (PMC8075682; doi:10.1155/2021/5576933)
Supplement: Supplementary Materials — Figure S1: the tenfold cross-validation penalized LASSO logistic regression for AA. LASSO: least absolute shrinkage and selection operator; AA: alternate acceptor site. Figure S2: the tenfold cross-validation penalized LASSO logistic regression for AD. LASSO: least absolute shrinkage and selection operator; AD: alternative donor site. Figure S3: the tenfold cross-validation penalized LASSO logistic regression for AP. LASSO: least absolute shrinkage and selection operator; AP: alternate promoter. Figure S4: The tenfold cross-validation penalized LASSO logistic regression for AT. LASSO: least absolute shrinkage and selection operator; AT: alternative terminator. Figure S5: the tenfold cross-validation penalized LASSO logistic regression for ES. LASSO: least absolute shrinkage and selection operator; ES: exon skip. Figure S6: the tenfold cross-validation penalized LASSO logistic regression for ME. LASSO: least absolute shrinkage and selection operator; ME: mutually exclusive exon. Figure S7: the tenfold cross-validation penalized LASSO logistic regression for RI. LASSO: least absolute shrinkage and selection operator; RI: retained intron exons. Figure S8: details of prognosis signatures of AA. (A) The risk scores of KIRC patients' distribution basing on the median value. (B) The green dots mean survivors, and the red dots mean death cases. (C) The heat map shows the alteration of the percent spliced in value from low risk score to high risk score. AA: alternate acceptor site; KIRC: kidney renal clear cell carcinoma. Figure S9: details of prognosis signatures of AD. (A) The risk scores of KIRC patients' distribution basing on the median value. (B) The green dots mean survivors, and the red dots mean death cases. (C) The heat map shows the alteration of the percent spliced in value from low risk score to high risk score. AD: alternative donor site; KIRC: kidney renal clear cell carcinoma. Figure S10: details of prognosis signatures of AP. (A) The risk scores of KIRC pati [file 5576933.f1.zip › 5576933.f1/Supplementary materials - TableS1-S10 (1).docx]

**Legends**

Table S1: The clinical information of the KIRC patients. In survival time, 0 means death case and 1 means the patients alive. T, tumor; M, metastasis; N, lymph node.

Table S2: The list of splicing factors genes collected from the SpliceAid 2 database.

Table S3: Result of the LASSO logistic regression with tenfold cross-validation to select the survival-associated AS events as prognostic signatures. LASSO, the least absolute shrinkage and selection operator; Coef, coefficient; HR, Hazard ratio; HR 95, Hazard ratio 95% confidence interval; L, low; H, high.

Table S4: The risk score of KIRC patients in AA prognosis signatures. AA, alternate acceptor site; KIRC, kidney renal clear cell carcinoma.

Table S5: The risk score of KIRC patients in AD prognosis signatures. AD, alternative donor site; KIRC, kidney renal clear cell carcinoma.

Table S6: The risk score of KIRC patients in AP prognosis signatures. AP, alternate promoter; KIRC, kidney renal clear cell carcinoma.

Table S7: The risk score of KIRC patients in AT prognosis signatures. AT, alternative terminator; KIRC, kidney renal clear cell carcinoma.

Table S8: The risk score of KIRC patients in ES prognosis signatures. ES, exon skip; KIRC, kidney renal clear cell carcinoma.

Table S9: The risk score of KIRC patients in ME prognosis signatures. ME, mutually exclusive exons; KIRC, kidney renal clear cell carcinoma.

Table S10: The risk score of KIRC patients in RI prognosis signatures. RI, retained intron; KIRC, kidney renal clear cell carcinoma.

Table S1

| **Id** | **Survival time** | **Status** | **Age** | **Gender** | **Grade** | **Stage** | **T** | **M** | **N** |
| --- | --- | --- | --- | --- | --- | --- | --- | --- | --- |
| TCGA-B8-5551 | 15 | 0 | 65 | FEMALE | G3 | Stage I | T1b | M0 | N0 |
| TCGA-B4-5835 | 16 | 0 | 64 | FEMALE | G2 | Stage I | T1 | M0 | N0 |
| TCGA-A3-3308 | 16 | 0 | 77 | FEMALE | G2 | Stage III | T3b | M0 | N0 |
| TCGA-B0-4813 | 18 | 1 | 68 | MALE | G3 | Stage III | T3b | M0 | NX |
| TCGA-CZ-4856 | 18 | 0 | 62 | FEMALE | G2 | Stage I | T1b | M0 | N0 |
| TCGA-B8-4619 | 19 | 0 | 58 | MALE | G2 | Stage I | T1a | M0 | N0 |
| TCGA-B8-5162 | 22 | 0 | 62 | MALE | G2 | Stage II | T2a | M0 | NX |
| TCGA-B8-5164 | 23 | 0 | 65 | MALE | G3 | Stage III | T3a | M0 | N0 |
| TCGA-CZ-5453 | 25 | 0 | 67 | MALE | G2 | Stage II | T2 | M0 | NX |
| TCGA-BP-5202 | 29 | 0 | 75 | MALE | G2 | Stage III | T3a | M0 | NX |
| TCGA-B4-5834 | 38 | 0 | 59 | MALE | G1 | Stage I | T1 | M0 | N0 |
| TCGA-CW-6087 | 41 | 1 | 61 | MALE | G4 | Stage IV | T3a | M1 | N1 |
| TCGA-B0-4698 | 42 | 1 | 75 | MALE | G4 | Stage IV | T4 | M0 | NX |
| TCGA-AS-3778 | 43 | 0 | 35 | MALE | G1 | Stage I | T1a | M0 | NX |
| TCGA-B0-4690 | 43 | 1 | 65 | MALE | G3 | Stage IV | T4 | M1 | N0 |
| TCGA-B2-4098 | 51 | 1 | 72 | FEMALE | G2 | Stage I | T1b | M0 | NX |
| TCGA-B8-A54G | 53 | 0 | 50 | MALE | G3 | Stage I | T1a | MX | NX |
| TCGA-CZ-5468 | 59 | 1 | 84 | MALE | G4 | Stage IV | T3b | M1 | NX |
| TCGA-B0-5119 | 59 | 0 | 61 | FEMALE | G2 | Stage I | T1b | M0 | N0 |
| TCGA-GK-A6C7 | 61 | 0 | 76 | FEMALE | unknow | Stage I | T1a | MX | NX |
| TCGA-BP-5173 | 62 | 1 | 75 | MALE | G2 | Stage I | T1a | M0 | NX |
| TCGA-B0-4706 | 65 | 1 | 61 | MALE | G4 | Stage III | T3a | M0 | NX |
| TCGA-B0-5096 | 68 | 1 | 72 | FEMALE | GX | Stage III | T3a | M0 | N1 |
| TCGA-B0-4849 | 69 | 1 | 51 | MALE | G3 | Stage III | T3a | M0 | NX |
| TCGA-CZ-5467 | 73 | 1 | 86 | FEMALE | G4 | Stage III | T3a | M0 | N0 |
| TCGA-B0-4693 | 77 | 1 | 72 | FEMALE | G4 | Stage III | T3a | M0 | N0 |
| TCGA-CJ-4918 | 93 | 1 | 64 | MALE | G4 | Stage IV | T3a | M1 | N0 |
| TCGA-B0-4710 | 96 | 0 | 75 | FEMALE | G3 | Stage III | T3a | M0 | N0 |
| TCGA-B0-4714 | 99 | 1 | 81 | MALE | G3 | Stage IV | T3b | M1 | NX |
| TCGA-B0-4688 | 101 | 1 | 46 | MALE | G4 | Stage IV | T4 | M1 | N0 |
| TCGA-B0-4694 | 106 | 1 | 72 | MALE | G4 | Stage III | T3b | M0 | NX |
| TCGA-BP-4327 | 109 | 1 | 75 | FEMALE | G2 | Stage II | T2 | M0 | N0 |
| TCGA-B0-4699 | 110 | 1 | 74 | MALE | G4 | Stage IV | T4 | M1 | N0 |
| TCGA-BP-4989 | 118 | 0 | 58 | MALE | G3 | Stage III | T3a | M0 | N0 |
| TCGA-EU-5905 | 119 | 0 | 67 | FEMALE | G3 | Stage I | T1 | M0 | NX |
| TCGA-EU-5907 | 127 | 0 | 81 | MALE | G3 | Stage III | T3a | M0 | NX |
| TCGA-A3-3346 | 137 | 1 | 68 | MALE | G3 | Stage I | T1b | M0 | NX |
| TCGA-B0-4691 | 139 | 1 | 55 | MALE | G3 | Stage IV | T2 | M1 | N0 |
| TCGA-CJ-4920 | 139 | 1 | 64 | FEMALE | G2 | Stage I | T1b | M0 | NX |
| TCGA-B4-5836 | 141 | 0 | 61 | FEMALE | G2 | Stage I | T1b | M0 | N0 |
| TCGA-B8-A54I | 150 | 0 | 48 | MALE | G3 | Stage I | T1b | MX | NX |
| TCGA-B4-5832 | 155 | 0 | 65 | MALE | G2 | Stage III | T3b | M0 | N0 |
| TCGA-BP-4771 | 162 | 1 | 62 | MALE | G4 | Stage IV | T3a | M1 | N0 |
| TCGA-CW-5584 | 164 | 1 | 74 | MALE | G3 | Stage III | T3b | M0 | N1 |
| TCGA-B4-5838 | 166 | 0 | 52 | MALE | G2 | unknow | T3 | M0 | N1 |
| TCGA-CZ-4865 | 166 | 1 | 70 | FEMALE | G2 | Stage I | T1a | M0 | NX |
| TCGA-B0-4814 | 168 | 1 | 58 | MALE | G3 | Stage IV | T4 | M1 | N0 |
| TCGA-B4-5378 | 175 | 0 | 62 | MALE | G2 | Stage I | T1 | M0 | N0 |
| TCGA-BP-4993 | 177 | 0 | 58 | MALE | G3 | Stage I | T1a | M0 | NX |
| TCGA-B8-4622 | 181 | 0 | 57 | MALE | G3 | Stage IV | T3a | M1 | N0 |
| TCGA-B0-4703 | 182 | 1 | 51 | MALE | G4 | Stage IV | T3a | M1 | N0 |
| TCGA-BP-4761 | 182 | 0 | 57 | MALE | G4 | Stage III | T3a | M0 | N1 |
| TCGA-B0-4819 | 183 | 1 | 60 | FEMALE | G4 | Stage IV | T3b | M1 | NX |
| TCGA-B2-4101 | 188 | 0 | 52 | MALE | G3 | Stage II | T2a | M0 | NX |
| TCGA-BP-5169 | 193 | 0 | 70 | MALE | G4 | Stage I | T1b | M0 | N0 |
| TCGA-B8-5549 | 194 | 0 | 53 | MALE | G3 | Stage I | T1b | M0 | N0 |
| TCGA-B0-4713 | 202 | 1 | 76 | FEMALE | G2 | Stage III | T3b | M0 | NX |
| TCGA-B2-4102 | 202 | 0 | 61 | MALE | G2 | Stage I | T1b | M0 | NX |
| TCGA-BP-4803 | 204 | 0 | 79 | MALE | G3 | Stage III | T3a | M0 | NX |
| TCGA-B0-4841 | 204 | 1 | 63 | MALE | G3 | Stage IV | T2 | M1 | NX |
| TCGA-BP-4967 | 205 | 0 | 76 | MALE | G2 | Stage III | T3a | M0 | N0 |
| TCGA-CZ-4860 | 206 | 1 | 60 | MALE | G4 | Stage IV | T4 | M1 | NX |
| TCGA-EU-5906 | 206 | 0 | 55 | MALE | G2 | Stage I | T1b | M0 | NX |
| TCGA-BP-4974 | 211 | 1 | 58 | MALE | G4 | Stage IV | T3a | M1 | N0 |
| TCGA-BP-4807 | 211 | 0 | 42 | MALE | G3 | Stage I | T1a | M0 | NX |
| TCGA-B0-5084 | 222 | 1 | 33 | MALE | G3 | Stage IV | T3a | M1 | N1 |
| TCGA-CJ-6033 | 224 | 1 | 54 | FEMALE | G4 | Stage IV | T3a | M1 | N0 |
| TCGA-B8-4620 | 226 | 0 | 70 | FEMALE | G2 | Stage III | T3a | M0 | N0 |
| TCGA-B0-4701 | 238 | 1 | 66 | FEMALE | G3 | Stage IV | T3a | M1 | N0 |
| TCGA-B8-5159 | 240 | 0 | 61 | FEMALE | G3 | Stage I | T1a | M0 | N0 |
| TCGA-G6-A5PC | 242 | 1 | 54 | FEMALE | G4 | Stage IV | T1b | M1 | N0 |
| TCGA-B0-5095 | 245 | 1 | 81 | MALE | G3 | Stage III | T3a | M0 | N0 |
| TCGA-B8-4154 | 255 | 0 | 73 | FEMALE | G2 | Stage I | T1a | M0 | N0 |
| TCGA-B8-A54H | 256 | 0 | 69 | FEMALE | G3 | Stage II | T2a | MX | N0 |
| TCGA-B2-5636 | 265 | 0 | 79 | MALE | G2 | Stage I | T1a | M0 | NX |
| TCGA-B8-4151 | 280 | 0 | 51 | FEMALE | G2 | Stage III | T3a | M0 | N0 |
| TCGA-BP-5177 | 293 | 0 | 46 | FEMALE | G3 | Stage I | T1a | M0 | NX |
| TCGA-B8-5158 | 293 | 0 | 56 | MALE | G4 | Stage III | T3a | M0 | N1 |
| TCGA-B0-4828 | 307 | 1 | 79 | MALE | G3 | Stage IV | T2 | M1 | NX |
| TCGA-CZ-5462 | 311 | 1 | 83 | MALE | G3 | Stage IV | T1b | M1 | NX |
| TCGA-B0-4844 | 313 | 1 | 60 | MALE | G3 | Stage IV | T3a | M1 | NX |
| TCGA-G6-A8L6 | 313 | 1 | 55 | MALE | G3 | Stage IV | T2a | MX | NX |
| TCGA-B2-5635 | 315 | 0 | 74 | MALE | G2 | Stage I | T1a | M0 | NX |
| TCGA-A3-3363 | 319 | 0 | 50 | MALE | G2 | Stage II | T2 | M0 | N0 |
| TCGA-B0-4843 | 320 | 1 | 57 | MALE | G3 | Stage III | T3a | M0 | N0 |
| TCGA-A3-A8OW | 323 | 0 | 37 | MALE | G2 | Stage III | T3a | MX | NX |
| TCGA-B2-5641 | 324 | 0 | 79 | MALE | G3 | Stage I | T1a | M0 | N0 |
| TCGA-BP-4770 | 329 | 1 | 73 | FEMALE | G4 | Stage IV | T4 | M0 | N0 |
| TCGA-CZ-5461 | 330 | 1 | 52 | MALE | G4 | Stage IV | T1b | M1 | NX |
| TCGA-B0-5094 | 333 | 1 | 62 | MALE | G2 | Stage IV | T3b | M1 | N0 |
| TCGA-BP-4798 | 334 | 1 | 74 | MALE | G4 | unknow | T3b | M1 | N0 |
| TCGA-CJ-4644 | 336 | 1 | 48 | FEMALE | G3 | Stage IV | T3a | M1 | N0 |
| TCGA-A3-A8OV | 340 | 0 | 75 | MALE | G2 | Stage I | T1a | MX | NX |
| TCGA-B0-5080 | 342 | 1 | 63 | MALE | G3 | Stage IV | T3a | M1 | N0 |
| TCGA-BP-4352 | 344 | 1 | 74 | FEMALE | G4 | Stage IV | T3b | M1 | N0 |
| TCGA-BP-4782 | 354 | 0 | 55 | FEMALE | G2 | Stage I | T1a | M0 | NX |
| TCGA-DV-5569 | 355 | 0 | 29 | FEMALE | G2 | Stage I | T1a | M0 | NX |
| TCGA-T7-A92I | 356 | 0 | 47 | FEMALE | G1 | Stage I | T1a | MX | NX |
| TCGA-B2-5633 | 358 | 0 | 56 | MALE | G2 | Stage I | T1b | M0 | N0 |
| TCGA-B0-5113 | 359 | 0 | 69 | FEMALE | G2 | Stage III | T3a | M0 | N0 |
| TCGA-6D-AA2E | 362 | 0 | 68 | FEMALE | G2 | Stage I | T1b | MX | NX |
| TCGA-B2-3923 | 362 | 0 | 59 | MALE | G2 | Stage II | T2 | M0 | NX |
| TCGA-B0-5081 | 362 | 1 | 79 | FEMALE | G2 | Stage III | T3b | M0 | N0 |
| TCGA-B4-5377 | 365 | 0 | 68 | FEMALE | G3 | Stage IV | T3 | M1 | N0 |
| TCGA-DV-A4VZ | 365 | 0 | 53 | MALE | G2 | Stage I | T1a | MX | NX |
| TCGA-AK-3465 | 369 | 0 | 71 | FEMALE | GX | Stage I | T1b | M0 | NX |
| TCGA-DV-5568 | 370 | 0 | 26 | MALE | G2 | Stage I | T1a | M0 | NX |
| TCGA-B2-3924 | 371 | 0 | 73 | MALE | G2 | Stage I | T1b | M0 | NX |
| TCGA-BP-4349 | 372 | 0 | 68 | FEMALE | G2 | Stage I | T1a | M0 | NX |
| TCGA-CZ-5986 | 373 | 0 | 61 | MALE | G3 | Stage I | T1 | M0 | N0 |
| TCGA-BP-4756 | 374 | 0 | 62 | FEMALE | G2 | Stage I | T1b | M0 | N0 |
| TCGA-B2-4099 | 374 | 0 | 83 | MALE | G3 | Stage I | T1a | M0 | NX |
| TCGA-BP-4353 | 375 | 1 | 61 | MALE | G2 | Stage I | T1 | M0 | N0 |
| TCGA-B8-4148 | 379 | 0 | 63 | FEMALE | G3 | Stage I | T1a | M0 | N0 |
| TCGA-3Z-A93Z | 385 | 0 | 69 | MALE | G2 | Stage I | T1a | M0 | N0 |
| TCGA-B8-5552 | 392 | 0 | 41 | FEMALE | G2 | Stage I | T1b | M0 | NX |
| TCGA-BP-4768 | 400 | 0 | 72 | FEMALE | G2 | Stage I | T1a | M0 | N0 |
| TCGA-B8-4153 | 405 | 0 | 74 | MALE | G3 | Stage III | T3a | M0 | NX |
| TCGA-BP-5187 | 406 | 0 | 54 | MALE | G2 | Stage I | T1a | M0 | NX |
| TCGA-BP-5194 | 408 | 0 | 39 | MALE | G2 | Stage I | T1a | M0 | NX |
| TCGA-BP-4776 | 411 | 0 | 52 | MALE | G2 | Stage I | T1a | M0 | NX |
| TCGA-B2-5639 | 417 | 0 | 46 | MALE | G3 | Stage IV | T3 | M1 | NX |
| TCGA-B8-A8YJ | 431 | 0 | 60 | FEMALE | G2 | Stage I | T1b | unknow | NX |
| TCGA-B8-4621 | 431 | 0 | 63 | MALE | G3 | Stage I | T1b | M0 | N0 |
| TCGA-CJ-4638 | 431 | 1 | 46 | FEMALE | G4 | Stage IV | T3a | M1 | N1 |
| TCGA-BP-4970 | 433 | 0 | 44 | MALE | G3 | Stage III | T1a | M0 | N1 |
| TCGA-B8-5550 | 434 | 0 | 71 | MALE | G3 | Stage III | T3a | M0 | N0 |
| TCGA-B8-5553 | 435 | 0 | 67 | FEMALE | G2 | Stage I | T1b | M0 | N0 |
| TCGA-CZ-5987 | 445 | 1 | 60 | MALE | G2 | Stage IV | T3b | M1 | NX |
| TCGA-CZ-4861 | 446 | 1 | 63 | MALE | G2 | Stage II | T2 | M0 | NX |
| TCGA-B0-5402 | 449 | 0 | 64 | MALE | G4 | Stage IV | T4 | M0 | NX |
| TCGA-BP-4977 | 454 | 0 | 57 | MALE | G3 | Stage I | T1b | M0 | NX |
| TCGA-B0-4823 | 454 | 1 | 88 | MALE | G2 | Stage I | T1a | M0 | N0 |
| TCGA-B0-5092 | 459 | 1 | 53 | FEMALE | G3 | Stage IV | T1a | M1 | N0 |
| TCGA-A3-A6NJ | 468 | 0 | 57 | FEMALE | G1 | Stage I | T1a | MX | NX |
| TCGA-B8-A54K | 469 | 0 | 61 | MALE | G1 | Stage I | T1a | MX | NX |
| TCGA-BP-4335 | 475 | 1 | 65 | FEMALE | G3 | Stage IV | T3a | M1 | N0 |
| TCGA-B0-4810 | 478 | 1 | 47 | MALE | G3 | Stage III | T3a | M0 | N1 |
| TCGA-AK-3430 | 480 | 1 | 61 | MALE | G3 | Stage III | T3b | M0 | N1 |
| TCGA-BP-4787 | 480 | 1 | 59 | FEMALE | G4 | Stage IV | T3a | M1 | N0 |
| TCGA-B0-5694 | 480 | 1 | 71 | MALE | G3 | Stage III | T3a | M0 | N0 |
| TCGA-B0-5099 | 485 | 1 | 88 | FEMALE | G3 | Stage III | T3b | M0 | NX |
| TCGA-B0-5120 | 493 | 0 | 72 | FEMALE | G2 | Stage I | T1a | M0 | N0 |
| TCGA-B8-A7U6 | 495 | 0 | 54 | FEMALE | G3 | Stage I | T1a | unknow | NX |
| TCGA-MW-A4EC | 498 | 0 | 72 | FEMALE | G2 | Stage I | T1a | MX | NX |
| TCGA-BP-4992 | 501 | 0 | 66 | MALE | G4 | Stage I | T1b | M0 | NX |
| TCGA-B8-5546 | 505 | 0 | 38 | FEMALE | G2 | Stage I | T1b | M0 | N0 |
| TCGA-B2-A4SR | 507 | 0 | 61 | MALE | unknow | Stage II | T2a | M0 | NX |
| TCGA-B0-4818 | 510 | 1 | 68 | FEMALE | G3 | Stage II | T2 | M0 | NX |
| TCGA-B8-4146 | 511 | 0 | 41 | FEMALE | G2 | Stage I | T1b | M0 | NX |
| TCGA-B8-A54F | 519 | 0 | 49 | FEMALE | G2 | Stage I | T1a | MX | NX |
| TCGA-B8-5545 | 522 | 0 | 42 | MALE | G2 | Stage I | T1a | M0 | N0 |
| TCGA-B8-A54J | 528 | 0 | 60 | MALE | G2 | Stage II | T2a | MX | NX |
| TCGA-B0-5117 | 535 | 0 | 40 | MALE | G2 | Stage I | T1b | M0 | NX |
| TCGA-EU-5904 | 551 | 0 | 47 | FEMALE | G1 | Stage I | T1 | M0 | NX |
| TCGA-CJ-5681 | 552 | 1 | 44 | FEMALE | G3 | Stage IV | T3a | M1 | NX |
| TCGA-B0-5121 | 554 | 0 | 56 | MALE | G2 | Stage I | T1b | M0 | N0 |
| TCGA-CZ-5455 | 561 | 1 | 63 | MALE | G4 | Stage IV | T3b | M1 | NX |
| TCGA-A3-3352 | 561 | 1 | 74 | MALE | G3 | Stage III | T3a | M0 | N0 |
| TCGA-BP-4340 | 562 | 1 | 70 | FEMALE | G2 | Stage I | T1b | M0 | N0 |
| TCGA-BP-5000 | 563 | 0 | 40 | MALE | G3 | Stage I | T1b | M0 | NX |
| TCGA-B0-5088 | 563 | 1 | 53 | MALE | G3 | Stage I | T1b | M0 | N0 |
| TCGA-A3-3380 | 567 | 0 | 54 | MALE | G2 | Stage I | T1 | M0 | N0 |
| TCGA-CW-6097 | 571 | 1 | 32 | MALE | G4 | Stage III | T3a | M0 | NX |
| TCGA-CJ-4923 | 572 | 1 | 63 | FEMALE | G4 | Stage IV | T3a | M1 | NX |
| TCGA-CJ-5678 | 574 | 1 | 62 | MALE | G3 | Stage IV | T2b | M1 | N0 |
| TCGA-A3-3382 | 574 | 0 | 69 | MALE | G3 | Stage I | T1b | M0 | NX |
| TCGA-B0-4697 | 578 | 1 | 46 | FEMALE | G4 | Stage IV | T3b | M1 | NX |
| TCGA-B0-5109 | 587 | 1 | 69 | MALE | G4 | Stage III | T3b | M0 | N1 |
| TCGA-MM-A563 | 591 | 0 | 41 | MALE | G2 | unknow | T3 | MX | NX |
| TCGA-B0-4707 | 600 | 1 | 63 | MALE | G4 | Stage III | T3a | M0 | NX |
| TCGA-BP-5198 | 603 | 0 | 72 | MALE | G3 | Stage III | T3b | M0 | N0 |
| TCGA-MM-A564 | 607 | 0 | 68 | MALE | G2 | Stage II | T2a | MX | NX |
| TCGA-B0-4718 | 616 | 0 | 57 | MALE | G2 | Stage III | T3a | M0 | NX |
| TCGA-A3-3387 | 617 | 0 | 49 | MALE | G2 | Stage I | T1a | M0 | N0 |
| TCGA-BP-4795 | 620 | 0 | 74 | FEMALE | G2 | Stage I | T1a | M0 | N0 |
| TCGA-A3-3378 | 630 | 0 | 60 | MALE | G3 | Stage I | T1 | M0 | N0 |
| TCGA-B0-5075 | 637 | 1 | 77 | FEMALE | G2 | Stage III | T3a | M0 | N0 |
| TCGA-BP-4334 | 645 | 1 | 56 | MALE | G3 | Stage III | T3a | M0 | N0 |
| TCGA-CJ-4868 | 646 | 1 | 42 | MALE | G3 | Stage IV | T3a | M1 | N0 |
| TCGA-B0-5399 | 652 | 0 | 46 | MALE | G2 | Stage I | T1b | M0 | N0 |
| TCGA-B0-5116 | 657 | 0 | 52 | MALE | G3 | Stage III | T3b | M0 | N0 |
| TCGA-CZ-5463 | 662 | 0 | 76 | MALE | G2 | Stage II | T2 | M0 | NX |
| TCGA-B0-5097 | 665 | 0 | 59 | FEMALE | G2 | Stage III | T3b | M0 | N0 |
| TCGA-CJ-5679 | 679 | 1 | 73 | MALE | G4 | Stage III | T3b | M0 | NX |
| TCGA-AK-3455 | 683 | 1 | 71 | FEMALE | G3 | Stage III | T3b | M0 | NX |
| TCGA-CZ-5466 | 685 | 0 | 67 | MALE | G2 | Stage III | T3a | M0 | NX |
| TCGA-A3-A6NL | 689 | 0 | 49 | FEMALE | G2 | Stage I | T1b | MX | NX |
| TCGA-CZ-5988 | 693 | 0 | 38 | MALE | G2 | Stage I | T1b | M0 | N0 |
| TCGA-BP-5186 | 693 | 0 | 50 | FEMALE | G2 | Stage I | T1a | M0 | N0 |
| TCGA-MM-A84U | 700 | 0 | 58 | FEMALE | G2 | Stage I | T1a | MX | NX |
| TCGA-BP-4169 | 701 | 1 | 76 | FEMALE | G2 | Stage II | T2 | M0 | N0 |
| TCGA-B8-4143 | 709 | 1 | 66 | FEMALE | G3 | Stage IV | T3a | M1 | N0 |
| TCGA-BP-5192 | 714 | 0 | 59 | MALE | G2 | Stage I | T1a | M0 | NX |
| TCGA-CZ-5454 | 722 | 1 | 63 | MALE | G2 | Stage IV | T2 | M1 | N0 |
| TCGA-DV-5574 | 723 | 0 | 37 | MALE | G2 | Stage I | T1a | M0 | NX |
| TCGA-DV-5576 | 727 | 1 | 55 | FEMALE | G2 | Stage I | T1a | M0 | NX |
| TCGA-A3-3313 | 735 | 1 | 59 | MALE | G3 | Stage I | T1b | M0 | N0 |
| TCGA-A3-3372 | 735 | 0 | 64 | MALE | G2 | Stage III | T3 | M0 | NX |
| TCGA-BP-5195 | 749 | 0 | 75 | MALE | G2 | Stage I | T1a | M0 | NX |
| TCGA-CJ-4893 | 750 | 0 | 76 | FEMALE | G3 | Stage I | T1b | M0 | NX |
| TCGA-CJ-5680 | 768 | 1 | 65 | FEMALE | G4 | Stage IV | T3a | M1 | NX |
| TCGA-B0-5085 | 770 | 1 | 76 | FEMALE | G3 | Stage III | T3a | M0 | N0 |
| TCGA-CZ-4853 | 774 | 0 | 82 | MALE | G2 | Stage I | T1a | M0 | NX |
| TCGA-CJ-5677 | 782 | 1 | 54 | FEMALE | G4 | Stage IV | T3a | M1 | NX |
| TCGA-BP-4986 | 785 | 0 | 75 | MALE | G3 | Stage I | T1a | M0 | N0 |
| TCGA-B0-4847 | 793 | 1 | 60 | MALE | G3 | Stage IV | T3a | M1 | NX |
| TCGA-B0-5115 | 797 | 0 | 43 | MALE | G3 | Stage IV | T2 | M1 | N0 |
| TCGA-CJ-4891 | 819 | 1 | 57 | FEMALE | G4 | Stage III | T3c | M0 | N0 |
| TCGA-BP-5189 | 822 | 1 | 60 | MALE | G4 | Stage I | T1b | M0 | NX |
| TCGA-BP-4988 | 828 | 1 | 72 | MALE | G2 | Stage I | T1a | M0 | N0 |
| TCGA-B8-A54D | 830 | 0 | 69 | MALE | G2 | Stage III | T3a | MX | NX |
| TCGA-B0-4838 | 834 | 1 | 69 | FEMALE | G3 | Stage I | T1b | M0 | N0 |
| TCGA-BP-5006 | 840 | 0 | 61 | MALE | G2 | Stage I | T1a | M0 | N0 |
| TCGA-CJ-4894 | 841 | 1 | 58 | MALE | G3 | Stage III | T3a | M0 | N0 |
| TCGA-BP-4329 | 845 | 1 | 75 | MALE | G2 | Stage III | T3a | M0 | N0 |
| TCGA-AK-3461 | 853 | 0 | 72 | MALE | G2 | Stage I | T1a | M0 | NX |
| TCGA-A3-3383 | 861 | 0 | 52 | MALE | G2 | Stage I | T1 | M0 | NX |
| TCGA-B0-4696 | 866 | 1 | 58 | MALE | G3 | Stage III | T3a | M0 | N0 |
| TCGA-A3-3365 | 873 | 0 | 46 | MALE | G2 | Stage I | T1a | M0 | NX |
| TCGA-AK-3454 | 874 | 0 | 84 | MALE | G3 | Stage I | T1b | M0 | NX |
| TCGA-BP-5010 | 878 | 1 | 63 | MALE | G4 | Stage III | T3a | M0 | N0 |
| TCGA-B0-4848 | 883 | 1 | 54 | MALE | G3 | Stage III | T3b | M0 | NX |
| TCGA-B0-4827 | 885 | 1 | 77 | FEMALE | G4 | Stage III | T3b | M0 | N0 |
| TCGA-AK-3426 | 885 | 1 | 37 | MALE | G3 | Stage III | T3a | M0 | N1 |
| TCGA-B8-A54E | 909 | 0 | 62 | FEMALE | G3 | Stage I | T1b | MX | NX |
| TCGA-A3-3351 | 910 | 0 | 42 | MALE | G2 | Stage II | T2a | M0 | N0 |
| TCGA-DV-5567 | 910 | 0 | 40 | FEMALE | G2 | Stage I | T1a | M0 | NX |
| TCGA-B0-5108 | 911 | 0 | 54 | MALE | G2 | Stage III | T3a | M0 | N0 |
| TCGA-B0-5107 | 927 | 1 | 65 | FEMALE | G4 | Stage IV | T2 | M1 | N0 |
| TCGA-BP-5175 | 932 | 0 | 60 | MALE | G3 | Stage I | T1a | M0 | NX |
| TCGA-BP-4998 | 932 | 0 | 49 | MALE | G3 | Stage I | T1a | M0 | NX |
| TCGA-CJ-4887 | 932 | 1 | 48 | MALE | G3 | Stage IV | T3a | M1 | NX |
| TCGA-A3-3343 | 945 | 0 | 79 | MALE | G3 | Stage II | T2 | M0 | N0 |
| TCGA-CZ-5469 | 946 | 1 | 41 | MALE | G2 | Stage II | T2 | M0 | N0 |
| TCGA-BP-5201 | 951 | 0 | 63 | MALE | G4 | Stage IV | T3b | M1 | N0 |
| TCGA-AK-3460 | 951 | 0 | 58 | MALE | G2 | Stage I | T1a | M0 | NX |
| TCGA-BP-4985 | 952 | 1 | 72 | MALE | G4 | Stage III | T3a | M0 | N0 |
| TCGA-BP-4355 | 953 | 1 | 59 | FEMALE | G4 | Stage III | T3a | M0 | NX |
| TCGA-BP-5191 | 967 | 0 | 79 | MALE | G2 | Stage III | T3a | M0 | N0 |
| TCGA-BP-4351 | 970 | 0 | 51 | FEMALE | G2 | Stage III | T3a | M0 | N0 |
| TCGA-BP-4164 | 992 | 1 | 51 | FEMALE | G2 | Stage III | T3a | M0 | NX |
| TCGA-DV-5575 | 1006 | 0 | 52 | FEMALE | G2 | Stage I | T1a | M0 | NX |
| TCGA-BP-5190 | 1011 | 0 | 61 | MALE | G3 | Stage I | T1a | M0 | NX |
| TCGA-BP-4982 | 1014 | 0 | 42 | MALE | G3 | Stage I | T1b | M0 | NX |
| TCGA-BP-5196 | 1018 | 0 | 53 | MALE | G2 | Stage I | T1a | M0 | NX |
| TCGA-A3-A6NI | 1018 | 0 | 47 | MALE | G3 | Stage I | T1a | MX | NX |
| TCGA-B0-4817 | 1019 | 1 | 81 | MALE | G3 | Stage III | T3c | M0 | N0 |
| TCGA-BP-4354 | 1034 | 1 | 40 | MALE | G4 | Stage IV | T4 | M1 | N1 |
| TCGA-A3-3336 | 1043 | 0 | 75 | FEMALE | G4 | Stage I | T1 | M0 | NX |
| TCGA-B0-5083 | 1045 | 1 | 63 | MALE | G3 | Stage I | T1a | M0 | N0 |
| TCGA-BP-5200 | 1063 | 0 | 44 | MALE | G4 | Stage II | T2 | M0 | NX |
| TCGA-BP-5008 | 1071 | 0 | 46 | MALE | G2 | Stage I | T1a | M0 | NX |
| TCGA-CW-5590 | 1075 | 1 | 51 | MALE | G3 | Stage IV | T3a | M1 | NX |
| TCGA-B0-5700 | 1082 | 0 | 77 | MALE | G2 | Stage I | T1a | M0 | N0 |
| TCGA-G6-A8L8 | 1091 | 1 | 62 | FEMALE | G3 | Stage I | T1b | MX | NX |
| TCGA-BP-5009 | 1092 | 1 | 52 | MALE | G3 | Stage I | T1b | M0 | NX |
| TCGA-B0-5110 | 1092 | 0 | 71 | FEMALE | G2 | Stage I | T1a | M0 | N0 |
| TCGA-BP-4981 | 1097 | 1 | 75 | FEMALE | G3 | Stage III | T3a | M0 | NX |
| TCGA-A3-3323 | 1106 | 0 | 53 | MALE | G1 | Stage I | T1b | M0 | NX |
| TCGA-BP-4797 | 1107 | 0 | 34 | MALE | G3 | Stage III | T3b | M0 | N0 |
| TCGA-B0-4822 | 1111 | 1 | 78 | MALE | G4 | Stage II | T2 | M0 | NX |
| TCGA-BP-4790 | 1111 | 1 | 76 | MALE | G2 | Stage I | T1a | M0 | NX |
| TCGA-A3-3306 | 1120 | 0 | 67 | MALE | G3 | Stage I | T1b | M0 | N0 |
| TCGA-B0-4852 | 1121 | 1 | 78 | FEMALE | G2 | Stage II | T2 | M0 | N0 |
| TCGA-BP-4987 | 1124 | 0 | 41 | FEMALE | G2 | Stage I | T1b | M0 | NX |
| TCGA-BP-4801 | 1124 | 0 | 57 | MALE | G2 | Stage I | T1a | M0 | NX |
| TCGA-BP-5004 | 1126 | 0 | 53 | MALE | G3 | Stage I | T1a | M0 | NX |
| TCGA-A3-3319 | 1130 | 0 | 70 | MALE | G2 | Stage I | T1b | M0 | NX |
| TCGA-DV-5573 | 1130 | 0 | 41 | MALE | G2 | Stage I | T1a | M0 | NX |
| TCGA-B0-5400 | 1132 | 0 | 59 | FEMALE | G4 | Stage III | T3b | M0 | N0 |
| TCGA-BP-5185 | 1132 | 0 | 56 | MALE | G3 | Stage I | T1a | M0 | NX |
| TCGA-BP-5184 | 1133 | 0 | 54 | MALE | G3 | Stage I | T1a | M0 | NX |
| TCGA-BP-4799 | 1133 | 1 | 70 | MALE | G3 | Stage III | T3b | M0 | N0 |
| TCGA-BP-4332 | 1133 | 0 | 36 | MALE | G2 | Stage III | T3a | M0 | N0 |
| TCGA-A3-3326 | 1137 | 0 | 47 | MALE | G1 | Stage I | T1a | M0 | NX |
| TCGA-BP-5007 | 1140 | 0 | 45 | MALE | G2 | Stage II | T2 | M0 | N0 |
| TCGA-AK-3456 | 1143 | 0 | 48 | MALE | G3 | Stage II | T2 | M0 | N0 |
| TCGA-BP-5182 | 1165 | 0 | 56 | MALE | G3 | Stage I | T1a | M0 | N0 |
| TCGA-AK-3458 | 1168 | 0 | 48 | MALE | G3 | Stage I | T1b | M0 | NX |
| TCGA-A3-3325 | 1170 | 1 | 52 | MALE | G2 | Stage I | T1a | M0 | NX |
| TCGA-CJ-4913 | 1173 | 1 | 45 | FEMALE | G4 | Stage III | T3a | M0 | NX |
| TCGA-BP-5001 | 1177 | 0 | 43 | FEMALE | G2 | Stage I | T1b | M0 | NX |
| TCGA-A3-3324 | 1186 | 0 | 51 | MALE | G3 | Stage I | T1b | M0 | NX |
| TCGA-A3-3311 | 1191 | 1 | 57 | MALE | G2 | Stage I | T1 | M0 | NX |
| TCGA-CJ-4895 | 1200 | 1 | 62 | MALE | G4 | Stage IV | T3a | M1 | NX |
| TCGA-B0-4846 | 1200 | 1 | 52 | MALE | G2 | Stage IV | T3a | M1 | N0 |
| TCGA-B0-5703 | 1203 | 0 | 73 | MALE | G3 | Stage I | T1b | M0 | N0 |
| TCGA-AK-3447 | 1217 | 0 | 83 | MALE | G2 | Stage II | T2 | M0 | NX |
| TCGA-B0-4821 | 1230 | 1 | 68 | FEMALE | G3 | Stage III | T3b | M0 | N0 |
| TCGA-AS-3777 | 1238 | 0 | 63 | MALE | unknow | Stage I | T1a | M0 | NX |
| TCGA-B0-4836 | 1238 | 1 | 61 | MALE | G3 | Stage IV | T3b | M1 | NX |
| TCGA-A3-3331 | 1257 | 0 | 86 | FEMALE | G2 | Stage I | T1 | M0 | N0 |
| TCGA-BP-4999 | 1266 | 0 | 56 | MALE | G2 | Stage I | T1a | M0 | NX |
| TCGA-BP-4763 | 1270 | 1 | 79 | FEMALE | G2 | Stage I | T1a | M0 | NX |
| TCGA-AK-3445 | 1280 | 0 | 69 | MALE | G3 | Stage III | T3a | M0 | NX |
| TCGA-BP-5183 | 1291 | 0 | 57 | MALE | G3 | Stage III | T3a | M0 | NX |
| TCGA-A3-3358 | 1307 | 0 | 57 | FEMALE | G2 | Stage I | T1a | M0 | N0 |
| TCGA-BP-4994 | 1308 | 0 | 54 | MALE | G3 | Stage I | T1a | M0 | NX |
| TCGA-A3-3374 | 1314 | 0 | 51 | FEMALE | G2 | Stage I | T1b | M0 | N0 |
| TCGA-CZ-4864 | 1315 | 1 | 86 | MALE | G3 | Stage II | T2 | M0 | N0 |
| TCGA-B0-5077 | 1317 | 1 | 77 | MALE | G3 | Stage I | T1a | M0 | N0 |
| TCGA-DV-5565 | 1329 | 0 | 59 | MALE | G2 | Stage I | T1a | M0 | NX |
| TCGA-B0-4712 | 1337 | 1 | 76 | MALE | G3 | Stage IV | T3a | M1 | NX |
| TCGA-BP-4762 | 1343 | 1 | 42 | MALE | G3 | Stage I | T1a | M0 | NX |
| TCGA-BP-5199 | 1355 | 0 | 58 | MALE | G4 | Stage II | T2 | M0 | N0 |
| TCGA-BP-4347 | 1367 | 0 | 74 | MALE | G2 | Stage III | T3b | M0 | NX |
| TCGA-BP-4995 | 1371 | 0 | 68 | MALE | G3 | Stage I | T1b | M0 | N0 |
| TCGA-B0-4816 | 1371 | 1 | 49 | MALE | G3 | Stage II | T2 | M0 | N0 |
| TCGA-CJ-4916 | 1373 | 0 | 69 | FEMALE | G3 | Stage III | T3a | M0 | NX |
| TCGA-CZ-5465 | 1377 | 0 | 76 | FEMALE | G2 | Stage III | T3b | M0 | NX |
| TCGA-B0-4837 | 1378 | 1 | 63 | MALE | G3 | Stage I | T1b | M0 | N0 |
| TCGA-BP-4973 | 1384 | 0 | 47 | MALE | G3 | Stage III | T3a | M0 | NX |
| TCGA-A3-3328 | 1385 | 0 | 79 | MALE | G2 | Stage I | T1b | M0 | N0 |
| TCGA-A3-3349 | 1385 | 0 | 34 | FEMALE | G2 | Stage I | T1b | M0 | N0 |
| TCGA-AK-3453 | 1397 | 0 | 58 | FEMALE | G2 | Stage II | T2 | M0 | NX |
| TCGA-DV-5566 | 1398 | 0 | 67 | FEMALE | G2 | Stage I | T1a | M0 | NX |
| TCGA-CZ-4854 | 1404 | 1 | 68 | MALE | G2 | Stage I | T1b | M0 | N0 |
| TCGA-BP-4991 | 1413 | 0 | 54 | MALE | G2 | Stage I | T1a | M0 | NX |
| TCGA-BP-4983 | 1413 | 0 | 67 | FEMALE | G4 | Stage III | T3a | M0 | NX |
| TCGA-CJ-4635 | 1416 | 0 | 48 | MALE | G3 | Stage I | T1b | M0 | NX |
| TCGA-B0-4811 | 1417 | 1 | 48 | MALE | G3 | Stage III | T3a | M0 | N0 |
| TCGA-B0-5695 | 1420 | 0 | 61 | FEMALE | G2 | Stage I | T1b | M0 | N0 |
| TCGA-AK-3443 | 1423 | 0 | 45 | MALE | GX | Stage II | T2 | M0 | N0 |
| TCGA-CZ-5460 | 1430 | 0 | 55 | MALE | G2 | Stage IV | T3b | M1 | NX |
| TCGA-CZ-4857 | 1432 | 1 | 56 | MALE | G3 | Stage IV | T3a | M1 | N0 |
| TCGA-BP-4975 | 1433 | 0 | 40 | MALE | G3 | Stage I | T1b | M0 | NX |
| TCGA-CJ-4872 | 1435 | 0 | 51 | MALE | G4 | Stage I | T1b | M0 | N0 |
| TCGA-A3-3307 | 1436 | 0 | 66 | MALE | G3 | Stage III | T3b | M0 | N0 |
| TCGA-CJ-4901 | 1450 | 0 | 47 | MALE | G3 | Stage III | T3b | M0 | NX |
| TCGA-BP-4804 | 1459 | 0 | 59 | MALE | G2 | Stage I | T1b | M0 | NX |
| TCGA-B0-5710 | 1459 | 0 | 57 | MALE | G2 | Stage I | T1b | M0 | N0 |
| TCGA-BP-4766 | 1462 | 0 | 43 | FEMALE | G3 | Stage I | T1a | M0 | NX |
| TCGA-BP-5168 | 1463 | 1 | 75 | MALE | G2 | Stage I | T1a | M0 | NX |
| TCGA-AK-3444 | 1471 | 0 | 80 | FEMALE | G2 | Stage I | T1b | M0 | NX |
| TCGA-A3-3322 | 1478 | 0 | 51 | MALE | G2 | Stage I | T1a | M0 | NX |
| TCGA-AK-3451 | 1481 | 0 | 48 | MALE | G3 | Stage II | T2 | M0 | N0 |
| TCGA-B0-5692 | 1487 | 0 | 66 | FEMALE | G3 | Stage III | T3b | M0 | N0 |
| TCGA-BP-4971 | 1487 | 0 | 40 | MALE | G3 | Stage III | T3a | M0 | N0 |
| TCGA-BP-4789 | 1489 | 0 | 48 | MALE | G2 | Stage I | T1a | M0 | NX |
| TCGA-A3-3317 | 1491 | 0 | 67 | MALE | G2 | Stage II | T2 | M0 | N0 |
| TCGA-CZ-5984 | 1491 | 0 | 51 | MALE | G3 | Stage I | T1b | M0 | N0 |
| TCGA-CZ-5464 | 1492 | 0 | 69 | MALE | G2 | Stage IV | T3b | M1 | NX |
| TCGA-A3-3316 | 1493 | 0 | 57 | MALE | G3 | Stage II | T2 | M0 | NX |
| TCGA-BP-4346 | 1493 | 1 | 57 | MALE | G3 | Stage III | T3b | M0 | N0 |
| TCGA-BP-5181 | 1495 | 0 | 58 | FEMALE | G2 | Stage I | T1b | M0 | NX |
| TCGA-CJ-4905 | 1496 | 0 | 62 | FEMALE | G2 | Stage I | T1a | M0 | NX |
| TCGA-CJ-4870 | 1498 | 0 | 58 | FEMALE | G2 | Stage III | T3a | M0 | NX |
| TCGA-CJ-4907 | 1499 | 0 | 58 | MALE | G3 | Stage III | T3b | M0 | NX |
| TCGA-BP-4972 | 1502 | 0 | 43 | FEMALE | G3 | Stage III | T3a | M0 | NX |
| TCGA-AK-3450 | 1508 | 0 | 85 | FEMALE | G2 | Stage I | T1a | M0 | N0 |
| TCGA-A3-3320 | 1508 | 0 | 52 | FEMALE | G1 | Stage I | T1b | M0 | NX |
| TCGA-CZ-5459 | 1515 | 0 | 63 | MALE | G3 | Stage III | T3b | M0 | NX |
| TCGA-BP-4345 | 1516 | 0 | 62 | MALE | G3 | Stage III | T3b | M0 | N0 |
| TCGA-CJ-4902 | 1520 | 0 | 61 | MALE | G3 | Stage III | T3a | M0 | NX |
| TCGA-CJ-4892 | 1521 | 0 | 65 | FEMALE | G2 | Stage I | T1b | M0 | N0 |
| TCGA-CJ-4899 | 1528 | 0 | 42 | MALE | G2 | Stage I | T1b | M0 | NX |
| TCGA-CJ-4908 | 1531 | 0 | 38 | MALE | G2 | Stage I | T1a | M0 | NX |
| TCGA-CZ-5457 | 1547 | 0 | 62 | MALE | G4 | Stage III | T3a | M0 | NX |
| TCGA-CZ-5452 | 1556 | 0 | 69 | MALE | G2 | Stage II | T2 | M0 | N0 |
| TCGA-CZ-5458 | 1558 | 0 | 43 | MALE | G3 | Stage III | T3a | M0 | NX |
| TCGA-CZ-5456 | 1558 | 0 | 57 | MALE | G3 | Stage II | T2 | M0 | N0 |
| TCGA-A3-3362 | 1559 | 0 | 60 | FEMALE | G2 | Stage I | T1a | M0 | N0 |
| TCGA-CJ-4903 | 1560 | 0 | 50 | MALE | G3 | Stage I | T1b | M0 | NX |
| TCGA-CJ-4888 | 1567 | 1 | 59 | MALE | G4 | Stage IV | T3a | M1 | NX |
| TCGA-B0-5098 | 1584 | 1 | 53 | FEMALE | G3 | Stage I | T1 | M0 | NX |
| TCGA-B0-4815 | 1588 | 1 | 65 | MALE | G4 | Stage III | T3a | M0 | NX |
| TCGA-BP-4341 | 1589 | 1 | 67 | MALE | G2 | Stage III | T3a | M0 | NX |
| TCGA-BP-5176 | 1590 | 1 | 78 | FEMALE | G2 | Stage I | T1a | M0 | NX |
| TCGA-B0-5106 | 1598 | 1 | 64 | MALE | G2 | Stage I | T1a | M0 | N0 |
| TCGA-CZ-5989 | 1599 | 0 | 60 | MALE | G2 | Stage II | T2 | M0 | N0 |
| TCGA-B0-5702 | 1605 | 0 | 71 | MALE | G2 | Stage I | T1b | M0 | N0 |
| TCGA-A3-3347 | 1610 | 1 | 76 | FEMALE | G2 | Stage III | T1b | M0 | N1 |
| TCGA-A3-3373 | 1621 | 0 | 54 | FEMALE | G3 | Stage I | T1b | M0 | N0 |
| TCGA-A3-3329 | 1624 | 0 | 75 | MALE | G2 | Stage I | T1b | M0 | N0 |
| TCGA-BP-4326 | 1625 | 1 | 53 | FEMALE | G2 | Stage I | T1b | M0 | N0 |
| TCGA-CJ-6028 | 1625 | 1 | 58 | MALE | G4 | Stage IV | T3a | M1 | NX |
| TCGA-DV-A4VX | 1626 | 1 | 59 | MALE | G4 | Stage IV | T3b | MX | N0 |
| TCGA-CJ-4642 | 1628 | 0 | 47 | MALE | G2 | Stage II | T2 | M0 | NX |
| TCGA-CZ-5985 | 1629 | 0 | 58 | MALE | G2 | Stage II | T2 | M0 | N0 |
| TCGA-BP-4976 | 1632 | 0 | 77 | MALE | G3 | Stage I | T1a | M0 | NX |
| TCGA-B0-4839 | 1639 | 1 | 80 | FEMALE | G2 | Stage I | T1b | M0 | N0 |
| TCGA-CJ-4912 | 1657 | 0 | 61 | MALE | G3 | Stage II | T2 | M0 | NX |
| TCGA-B0-4824 | 1657 | 1 | 49 | FEMALE | G3 | Stage I | T1a | M0 | N0 |
| TCGA-CJ-4641 | 1661 | 1 | 55 | FEMALE | G4 | Stage IV | T3a | M1 | NX |
| TCGA-BP-4344 | 1666 | 0 | 75 | FEMALE | G2 | Stage I | T1a | M0 | NX |
| TCGA-CZ-5451 | 1668 | 0 | 74 | MALE | G3 | Stage II | T2 | M0 | N0 |
| TCGA-BP-4177 | 1670 | 0 | 65 | MALE | G2 | Stage I | T1a | M0 | NX |
| TCGA-CZ-4863 | 1691 | 0 | 51 | FEMALE | G3 | Stage III | T3b | M0 | N0 |
| TCGA-A3-3376 | 1696 | 1 | 51 | MALE | G2 | Stage I | T1a | M0 | N0 |
| TCGA-CJ-4900 | 1714 | 1 | 69 | FEMALE | G4 | Stage IV | T4 | M1 | N1 |
| TCGA-B0-4842 | 1724 | 1 | 73 | FEMALE | G4 | Stage III | T3a | M0 | N0 |
| TCGA-B0-5696 | 1727 | 0 | 69 | MALE | G4 | Stage III | T3a | M0 | N0 |
| TCGA-BP-4777 | 1731 | 0 | 46 | MALE | G3 | Stage I | T1a | M0 | NX |
| TCGA-B0-5701 | 1732 | 0 | 65 | MALE | G4 | Stage III | T3b | M0 | N0 |
| TCGA-AK-3440 | 1745 | 0 | 58 | MALE | G3 | Stage I | T1a | M0 | NX |
| TCGA-BP-4968 | 1746 | 0 | 40 | MALE | G3 | Stage I | T1b | M0 | N0 |
| TCGA-CJ-4884 | 1759 | 0 | 72 | FEMALE | G3 | Stage III | T3a | M0 | NX |
| TCGA-CZ-4866 | 1768 | 0 | 79 | FEMALE | G3 | Stage I | T1 | M0 | NX |
| TCGA-CJ-4873 | 1776 | 0 | 85 | FEMALE | G3 | Stage III | T3a | M0 | N0 |
| TCGA-BP-4962 | 1785 | 0 | 58 | MALE | G2 | Stage II | T2 | M0 | NX |
| TCGA-CZ-4859 | 1787 | 0 | 59 | FEMALE | G2 | Stage I | T1 | M0 | N0 |
| TCGA-CJ-4904 | 1792 | 0 | 60 | FEMALE | G3 | Stage IV | T3a | M1 | N0 |
| TCGA-CJ-4643 | 1793 | 0 | 67 | FEMALE | G3 | Stage II | T2b | M0 | N0 |
| TCGA-BP-4969 | 1794 | 0 | 63 | FEMALE | G2 | Stage I | T1a | M0 | NX |
| TCGA-CJ-4897 | 1808 | 0 | 79 | FEMALE | G3 | Stage III | T3a | M0 | NX |
| TCGA-CJ-4634 | 1820 | 0 | 60 | FEMALE | G2 | Stage I | T1b | M0 | NX |
| TCGA-BP-4963 | 1834 | 0 | 63 | MALE | G3 | Stage I | T1b | M0 | NX |
| TCGA-B0-5697 | 1835 | 0 | 50 | MALE | G2 | Stage I | T1a | M0 | N0 |
| TCGA-BP-4775 | 1843 | 0 | 55 | FEMALE | G2 | Stage I | T1a | M0 | NX |
| TCGA-CZ-4862 | 1843 | 0 | 46 | MALE | G2 | Stage I | T1b | M0 | NX |
| TCGA-AK-3431 | 1853 | 0 | 62 | FEMALE | G3 | Stage II | T2 | M0 | NX |
| TCGA-BP-4784 | 1854 | 0 | 67 | FEMALE | G2 | Stage I | T1a | M0 | NX |
| TCGA-CJ-6027 | 1855 | 0 | 77 | MALE | G4 | Stage I | T1a | M0 | NX |
| TCGA-BP-4964 | 1862 | 0 | 54 | FEMALE | G2 | Stage I | T1a | M0 | N0 |
| TCGA-B0-5713 | 1865 | 0 | 75 | FEMALE | G3 | Stage III | T3b | M0 | N0 |
| TCGA-BP-4965 | 1871 | 0 | 46 | MALE | G2 | Stage I | T1a | M0 | NX |
| TCGA-BP-4769 | 1876 | 0 | 63 | MALE | G2 | Stage I | T1a | M0 | NX |
| TCGA-BP-4174 | 1879 | 0 | 49 | MALE | G3 | Stage II | T2 | M0 | N0 |
| TCGA-CJ-5682 | 1883 | 0 | 60 | MALE | G4 | Stage IV | T3a | M1 | NX |
| TCGA-CJ-4882 | 1883 | 0 | 57 | MALE | G3 | Stage III | T3a | M0 | NX |
| TCGA-BP-4774 | 1885 | 0 | 57 | FEMALE | G2 | Stage I | T1a | M0 | NX |
| TCGA-A3-3335 | 1886 | 0 | 41 | MALE | G4 | Stage II | T2a | M0 | N0 |
| TCGA-BP-4330 | 1888 | 0 | 60 | FEMALE | G2 | Stage III | T3a | M0 | N0 |
| TCGA-CJ-5683 | 1889 | 0 | 78 | MALE | G3 | Stage I | T1b | M0 | NX |
| TCGA-BP-4173 | 1893 | 0 | 47 | MALE | G3 | Stage II | T2 | M0 | N0 |
| TCGA-CJ-6031 | 1906 | 0 | 54 | MALE | G3 | Stage I | T1b | M0 | NX |
| TCGA-BP-5178 | 1912 | 1 | 71 | MALE | G4 | Stage IV | T3a | M1 | NX |
| TCGA-BP-4343 | 1912 | 1 | 64 | MALE | G3 | Stage III | T3a | M0 | N0 |
| TCGA-B0-5100 | 1913 | 1 | 72 | MALE | G3 | Stage III | T3a | M0 | NX |
| TCGA-CJ-4636 | 1924 | 0 | 51 | MALE | G3 | Stage III | T3a | M0 | N0 |
| TCGA-BP-4961 | 1935 | 0 | 47 | MALE | G2 | Stage I | T1a | M0 | NX |
| TCGA-CZ-4858 | 1943 | 0 | 39 | MALE | G4 | Stage II | T2 | M0 | NX |
| TCGA-CJ-5671 | 1943 | 0 | 51 | MALE | G3 | Stage I | T1a | M0 | NX |
| TCGA-CJ-4889 | 1946 | 0 | 63 | FEMALE | G4 | Stage I | T1a | M0 | NX |
| TCGA-CJ-4886 | 1952 | 0 | 42 | FEMALE | G3 | Stage I | T1a | M0 | NX |
| TCGA-CJ-4876 | 1955 | 0 | 57 | MALE | G3 | Stage II | T2b | M0 | N0 |
| TCGA-BP-4176 | 1955 | 0 | 64 | MALE | G2 | Stage I | T1b | M0 | NX |
| TCGA-CW-5580 | 1964 | 1 | 73 | FEMALE | G3 | Stage IV | T3a | M1 | NX |
| TCGA-CJ-5672 | 1972 | 1 | 84 | MALE | G3 | Stage I | T1a | M0 | NX |
| TCGA-B0-4700 | 1980 | 1 | 60 | MALE | G4 | Stage IV | T4 | M1 | NX |
| TCGA-B0-4845 | 1986 | 1 | 70 | MALE | G2 | Stage IV | T3a | M1 | NX |
| TCGA-A3-3385 | 1993 | 0 | 46 | FEMALE | G2 | Stage I | T1a | M0 | N0 |
| TCGA-CJ-4640 | 1998 | 0 | 49 | MALE | G4 | Stage III | T3a | M0 | N0 |
| TCGA-DV-A4W0 | 2008 | 0 | 55 | MALE | G3 | Stage I | T1b | MX | NX |
| TCGA-CJ-4881 | 2014 | 0 | 41 | MALE | G3 | Stage III | T3a | M0 | NX |
| TCGA-CW-5588 | 2017 | 0 | 78 | FEMALE | G2 | Stage I | T1a | M0 | NX |
| TCGA-AK-3429 | 2017 | 0 | 54 | FEMALE | G2 | Stage II | T2 | M0 | N0 |
| TCGA-CJ-5686 | 2038 | 0 | 59 | FEMALE | G3 | Stage I | T1b | M0 | NX |
| TCGA-CZ-5982 | 2042 | 0 | 59 | FEMALE | G2 | Stage I | T1a | M0 | NX |
| TCGA-AK-3436 | 2044 | 0 | 40 | MALE | G2 | Stage IV | T2 | M1 | N0 |
| TCGA-BP-4781 | 2080 | 0 | 78 | MALE | G3 | Stage I | T1a | M0 | NX |
| TCGA-CJ-4890 | 2085 | 0 | 72 | MALE | G4 | Stage IV | T3a | M1 | N0 |
| TCGA-AK-3434 | 2087 | 0 | 72 | MALE | G2 | Stage I | T1b | M0 | NX |
| TCGA-B0-4834 | 2090 | 1 | 49 | MALE | G3 | Stage I | T1a | M0 | N0 |
| TCGA-CJ-4885 | 2125 | 0 | 64 | MALE | G3 | Stage IV | T3a | M1 | NX |
| TCGA-G6-A8L7 | 2133 | 0 | 81 | FEMALE | G3 | Stage I | T1b | MX | N0 |
| TCGA-B0-4945 | 2145 | 1 | 75 | FEMALE | G2 | Stage I | T1a | M0 | N0 |
| TCGA-BP-4960 | 2172 | 0 | 46 | MALE | G3 | Stage II | T2 | M0 | N0 |
| TCGA-BP-4765 | 2184 | 0 | 43 | MALE | G2 | Stage I | T1a | M0 | NX |
| TCGA-CJ-4878 | 2186 | 0 | 71 | FEMALE | G2 | Stage III | T3a | M0 | NX |
| TCGA-AK-3433 | 2192 | 0 | 48 | FEMALE | GX | Stage II | T2 | M0 | N0 |
| TCGA-BP-4758 | 2208 | 0 | 40 | MALE | G2 | Stage I | T1a | M0 | NX |
| TCGA-AK-3428 | 2223 | 0 | 62 | MALE | G2 | Stage III | T3b | M0 | N0 |
| TCGA-CW-5587 | 2226 | 0 | 62 | FEMALE | G2 | Stage III | T3b | M0 | N0 |
| TCGA-CJ-4637 | 2227 | 1 | 52 | FEMALE | G4 | Stage IV | T2b | M1 | NX |
| TCGA-CJ-5684 | 2231 | 0 | 61 | MALE | G2 | Stage III | T3a | M0 | NX |
| TCGA-BP-4342 | 2256 | 1 | 79 | MALE | G3 | Stage II | T2 | M0 | N0 |
| TCGA-BP-5174 | 2257 | 0 | 45 | FEMALE | G2 | Stage I | T1a | M0 | NX |
| TCGA-BP-5180 | 2263 | 0 | 53 | MALE | G2 | Stage I | T1a | M0 | NX |
| TCGA-A3-3367 | 2270 | 0 | 72 | MALE | G3 | Stage I | T1b | M0 | N0 |
| TCGA-CW-5591 | 2271 | 0 | 56 | MALE | G2 | Stage IV | T3a | M1 | N0 |
| TCGA-A3-3370 | 2274 | 0 | 48 | FEMALE | G2 | Stage I | T1b | M0 | N0 |
| TCGA-CJ-4874 | 2283 | 0 | 73 | FEMALE | G3 | Stage I | T1b | M0 | N0 |
| TCGA-CJ-6030 | 2299 | 1 | 65 | MALE | G3 | Stage I | T1a | M0 | N0 |
| TCGA-CJ-4639 | 2308 | 0 | 49 | FEMALE | G2 | Stage II | T2 | M0 | N0 |
| TCGA-BP-4170 | 2343 | 1 | 72 | FEMALE | G2 | Stage I | T1b | M0 | N0 |
| TCGA-CJ-4875 | 2353 | 0 | 67 | MALE | G3 | Stage IV | T3a | M1 | NX |
| TCGA-BP-4760 | 2361 | 0 | 69 | MALE | G2 | Stage I | T1a | M0 | NX |
| TCGA-BP-4759 | 2372 | 0 | 50 | MALE | G2 | Stage I | T1a | M0 | NX |
| TCGA-CW-5589 | 2378 | 0 | 52 | MALE | G2 | Stage I | T1a | M0 | NX |
| TCGA-B0-4833 | 2386 | 1 | 82 | FEMALE | G2 | Stage I | T1b | M0 | N0 |
| TCGA-B0-5690 | 2408 | 0 | 53 | FEMALE | G1 | Stage I | T1b | M0 | NX |
| TCGA-BP-5170 | 2412 | 0 | 55 | MALE | G2 | Stage I | T1a | M0 | NX |
| TCGA-B0-5706 | 2414 | 0 | 45 | MALE | G2 | Stage II | T2 | M0 | N0 |
| TCGA-CJ-4871 | 2423 | 0 | 63 | MALE | G4 | Stage IV | T3a | M1 | NX |
| TCGA-CJ-5675 | 2430 | 0 | 70 | MALE | G3 | Stage II | T2a | M0 | NX |
| TCGA-BP-4331 | 2454 | 1 | 52 | MALE | G2 | Stage I | T1a | M0 | N0 |
| TCGA-CW-5583 | 2489 | 0 | 51 | FEMALE | G2 | Stage I | T1a | M0 | NX |
| TCGA-A3-3359 | 2504 | 0 | 82 | FEMALE | G2 | Stage I | T1a | M0 | N0 |
| TCGA-CJ-6032 | 2548 | 0 | 63 | FEMALE | G3 | Stage II | T2 | M0 | NX |
| TCGA-CW-6090 | 2552 | 0 | 68 | MALE | G3 | Stage I | T1b | M0 | NX |
| TCGA-CJ-4869 | 2554 | 0 | 49 | MALE | G2 | Stage III | T2 | M0 | N1 |
| TCGA-AK-3427 | 2566 | 0 | 65 | MALE | GX | Stage I | T1a | M0 | N0 |
| TCGA-CJ-5676 | 2575 | 0 | 47 | MALE | G3 | Stage III | T3b | M0 | NX |
| TCGA-B0-5698 | 2583 | 0 | 77 | MALE | G3 | Stage I | T1b | M0 | N0 |
| TCGA-BP-4159 | 2601 | 1 | 70 | MALE | G2 | Stage I | T1b | M0 | N0 |
| TCGA-CW-5585 | 2609 | 0 | 51 | MALE | G2 | Stage IV | T3b | M1 | N0 |
| TCGA-BP-4959 | 2660 | 0 | 49 | MALE | G3 | Stage I | T1b | M0 | NX |
| TCGA-A3-3357 | 2688 | 0 | 62 | MALE | G3 | Stage II | T2 | M0 | N0 |
| TCGA-CW-6096 | 2701 | 0 | 44 | FEMALE | G2 | Stage I | T1a | M0 | NX |
| TCGA-BP-4167 | 2718 | 0 | 59 | MALE | G2 | Stage III | T3a | M0 | NX |
| TCGA-B0-5712 | 2722 | 0 | 68 | FEMALE | G3 | Stage IV | T2 | M1 | N0 |
| TCGA-B0-5699 | 2741 | 0 | 53 | MALE | G2 | Stage I | T1 | M0 | N0 |
| TCGA-BP-4161 | 2746 | 0 | 74 | MALE | G3 | Stage I | T1b | M0 | NX |
| TCGA-B0-5102 | 2764 | 1 | 74 | FEMALE | G3 | Stage I | T1 | M0 | NX |
| TCGA-CW-5581 | 2799 | 0 | 44 | MALE | G3 | Stage I | T1b | M0 | NX |
| TCGA-B0-5707 | 2828 | 0 | 39 | FEMALE | G3 | Stage I | T1a | M0 | N0 |
| TCGA-BP-4163 | 2839 | 0 | 60 | FEMALE | G3 | Stage III | T3a | M0 | N0 |
| TCGA-BP-4338 | 2859 | 0 | 43 | MALE | G3 | Stage I | T1b | M0 | N0 |
| TCGA-BP-4160 | 2881 | 0 | 67 | MALE | G2 | Stage III | T3a | M0 | N0 |
| TCGA-B0-5711 | 2931 | 0 | 50 | MALE | G3 | Stage III | T3b | M0 | NX |
| TCGA-B0-5812 | 2963 | 0 | 53 | MALE | G3 | Stage I | T1b | M0 | NX |
| TCGA-BP-4325 | 2964 | 0 | 64 | FEMALE | G2 | Stage I | T1b | M0 | N0 |
| TCGA-BP-4165 | 3037 | 0 | 64 | FEMALE | G1 | Stage I | T1b | M0 | N0 |
| TCGA-BP-4162 | 3074 | 0 | 65 | FEMALE | G2 | Stage I | T1b | M0 | N0 |
| TCGA-B0-5693 | 3076 | 0 | 47 | FEMALE | G2 | Stage I | T1b | M0 | NX |
| TCGA-B0-5709 | 3117 | 0 | 62 | FEMALE | G3 | Stage III | T3a | M0 | NX |
| TCGA-CW-6093 | 3146 | 0 | 73 | MALE | G1 | Stage I | T1a | M0 | NX |
| TCGA-CW-6088 | 3222 | 0 | 60 | MALE | G2 | Stage I | T1b | M0 | N0 |
| TCGA-AK-3425 | 3343 | 0 | 68 | MALE | G2 | Stage I | T1 | M0 | N0 |
| TCGA-BP-4158 | 3377 | 0 | 69 | MALE | G2 | Stage I | T1b | M0 | N0 |
| TCGA-B0-5691 | 3431 | 0 | 66 | FEMALE | G3 | Stage I | T1a | M0 | N0 |
| TCGA-B0-5705 | 3668 | 0 | 65 | FEMALE | G2 | Stage I | T1 | M0 | N0 |

Table S2

| **Splicing factors** | | | | | | | | | | | |
| --- | --- | --- | --- | --- | --- | --- | --- | --- | --- | --- | --- |
| CFAP20 | DDX46 | FUBP1 | HSPA5 | LUC7L | PLRG1 | PUF60 | RNF20 | SNRNP27 | SRSF4 | U2SURP |  |
| CHERP | DDX5 | FUBP3 | HSPA8 | LUC7L2 | PNN | QKI | RNF213 | SNRNP35 | SRSF5 | UBL5 |  |
| CIRBP | DDX50 | FUS | HSPB1 | LUC7L3 | PPIE | RALY | RNF34 | SNRNP40 | SRSF6 | USP39 |  |
| CLASRP | DDX6 | GEMIN2 | HTATSF1 | MAGOH | PPIG | RALYL | RNF40 | SNRNP48 | SRSF7 | WBP11 |  |
| CLK1 | DGCR14 | GEMIN5 | IGF2BP3 | MATR3 | PPIH | RAVER1 | RNPC3 | SNRNP70 | SRSF8 | WBP4 |  |
| CLK2 | DHX15 | GNB2L1 | IK | MBNL1 | PPIL1 | RAVER2 | RNPS1 | SNRPA | SRSF9 | WDR77 |  |
| CLK3 | DHX16 | GPATCH1 | ILF2 | MBNL2 | PPIL2 | RBBP6 | RNU1-1 | SNRPA1 | SSB | WDR83 |  |
| CLK4 | DHX30 | GPATCH3 | ILF3 | MBNL3 | PPIL3 | RBFOX2 | RNU2-1 | SNRPB | SUGP1 | WTAP |  |
| CLNS1A | DHX34 | GPATCH8 | INTS1 | MFAP1 | PPIL4 | RBM10 | RNU4-1 | SNRPB2 | SYF2 | XAB2 |  |
| CPSF6 | DHX35 | GPKOW | INTS3 | MFSD11 | PPM1G | RBM14 | RNU5A-1 | SNRPC | SYNCRIP | YBX1 |  |
| CRNKL1 | DHX36 | GRSF1 | INTS4 | MOV10 | PPP1CA | RBM15 | RNU6-1 | SNRPD1 | TAF15 | YBX3 |  |
| CSN3 | DHX38 | HNRNPA0 | INTS5 | MSI1 | PPP1R8 | RBM15B | SAP18 | SNRPD2 | TCERG1 | ZC3H11A |  |

Table S3

| **ID** | **Coef** | **HR** | **HR 95(L)** | **HR 95(H)** | **pvalue** |
| --- | --- | --- | --- | --- | --- |
| RPS24\|12297\|AA | -1.71553 | 0.179869 | 0.064002 | 0.505497169 | 0.001138 |
| TAF1D\|18320\|AA | 2.206104 | 9.080269 | 1.688368 | 48.83490495 | 0.010165 |
| WDR6\|64803\|AA | 1.924074 | 6.848806 | 1.822711 | 25.73427244 | 0.004388 |
| MEIS3\|50645\|AA | 1.437793 | 4.21139 | 1.480527 | 11.97939136 | 0.007025 |
| CEP76\|44711\|AA | -2.0827 | 0.124594 | 0.035737 | 0.434382417 | 0.001081 |
| LIG1\|50690\|AA | 1.882606 | 6.570606 | 0.957745 | 45.07760354 | 0.055362 |
| ESAM\|19236\|AD | 9.137187 | 9294.583 | 109.206 | 791067.3964 | 5.58E-05 |
| STRA13\|44264\|AD | 2.145476 | 8.546106 | 0.907704 | 80.46228655 | 0.060749 |
| PIGG\|68359\|AD | -2.24608 | 0.105813 | 0.009362 | 1.196001374 | 0.069477 |
| PRMT2\|60963\|AD | 1.100315 | 3.005113 | 1.069228 | 8.446002864 | 0.036895 |
| UQCR10\|61665\|AD | 15.67514 | 6421335 | 17032.09 | 2420932874 | 2.23E-07 |
| UACA\|31439\|AP | 2.086507 | 8.056726 | 2.945196 | 22.03956668 | 4.83E-05 |
| LIMA1\|21691\|AP | -1.60919 | 0.200051 | 0.064474 | 0.620719967 | 0.005346 |
| ARHGAP24\|69814\|AP | 1.771781 | 5.88132 | 1.365412 | 25.33296451 | 0.017408 |
| BROX\|9895\|AP | 0.813004 | 2.254671 | 0.760275 | 6.686447085 | 0.142698 |
| TRIM16L\|39631\|AP | 1.233983 | 3.434883 | 1.400962 | 8.421653003 | 0.007001 |
| EPC2\|55538\|AT | -3.25994 | 0.038391 | 0.002916 | 0.505444723 | 0.013183 |
| C4orf19\|69001\|AT | -1.94486 | 0.143007 | 0.052747 | 0.387718288 | 0.000132 |
| FAM120C\|89237\|AT | 0.996501 | 2.708786 | 0.701534 | 10.45925066 | 0.148265 |
| BCCIP\|13432\|AT | -4.28957 | 0.013711 | 0.000817 | 0.230071543 | 0.002872 |
| INPP4B\|70691\|AT | -2.13958 | 0.117704 | 0.00674 | 2.055576845 | 0.142597 |
| GK\|88735\|ES | 1.652801 | 5.221586 | 1.196076 | 22.79533794 | 0.027943 |
| SIPA1L1\|28199\|ES | 1.767574 | 5.856629 | 1.12512 | 30.48573201 | 0.035727 |
| MGRN1\|33783\|ES | -6.1403 | 0.002154 | 1.06E-06 | 4.361247946 | 0.113922 |
| CLEC1A\|20304\|ES | 0.805223 | 2.237196 | 0.955529 | 5.237984331 | 0.063574 |
| C16orf13\|32919\|ES | -4.57887 | 0.010266 | 0.000207 | 0.509379273 | 0.021528 |
| KLHL42\|20900\|ES | 0.99535 | 2.70567 | 0.731759 | 10.00418089 | 0.135733 |
| SEC31A\|100881\|ES | -1.41715 | 0.242403 | 0.095389 | 0.615997925 | 0.0029 |
| SERP2\|25779\|ME | -4.4807 | 0.011326 | 0.000679 | 0.1890423 | 0.00181 |
| ANKRD13A\|250011\|ME | 2.91313 | 18.41434 | 1.301874 | 260.4614345 | 0.031152 |
| TPM2\|86278\|ME | -6.21401 | 0.002001 | 8.85E-05 | 0.045264065 | 9.42E-05 |
| P4HA1\|12122\|ME | -2.99479 | 0.050047 | 0.004286 | 0.584420192 | 0.016925 |
| STEAP3\|95656\|ME | 0.763826 | 2.146472 | 1.212945 | 3.798474347 | 0.008719 |
| ZNF611\|101327\|ME | -1.53637 | 0.21516 | 0.066213 | 0.69916197 | 0.010614 |
| ERBB2IP\|102012\|ME | -1.00161 | 0.367289 | 0.178364 | 0.756325457 | 0.006572 |
| SLC39A14\|140283\|ME | -1.4564 | 0.233073 | 0.057761 | 0.940477135 | 0.040738 |
| ADAM28\|83091\|ME | -2.30862 | 0.099398 | 0.011779 | 0.838777637 | 0.033878 |
| QSOX1\|9130\|RI | -6.32711 | 0.001787 | 0.000167 | 0.019183291 | 1.74E-07 |
| DHRS1\|26954\|RI | 1.833112 | 6.253315 | 1.508167 | 25.92813382 | 0.01153 |
| TAF1D\|18313\|RI | 1.766397 | 5.849738 | 1.167288 | 29.3153314 | 0.031708 |

Table S4

| ID | RPS24\|12297\|AA | TAF1D\|18320\|AA | WDR6\|64803\|AA | MEIS3\|50645\|AA | CEP76\|44711\|AA | LIG1\|50690\|AA | Risk score | risk |
| --- | --- | --- | --- | --- | --- | --- | --- | --- |
| TCGA-CJ-4637 | 0.3085 | 0.2242 | 0.2162 | 0.4862 | 0.3861 | 0.0458 | 3.754196 | high |
| TCGA-CZ-4861 | 0.267 | 0.1068 | 0.1997 | 0.2245 | 0.7956 | 0.0388 | 0.870238 | low |
| TCGA-BP-4964 | 0.5643 | 0.1188 | 0.2532 | 0.3996 | 1 | 0.059 | 0.519172 | low |
| TCGA-CJ-4887 | 0.4549 | 0.2833 | 0.2527 | 0.3554 | 0.761 | 0.0351 | 1.327531 | high |
| TCGA-BP-5198 | 0.2622 | 0.2828 | 0.1274 | 0.5851 | 0.8705 | 0 | 1.503565 | high |
| TCGA-BP-4967 | 0.4703 | 0.2676 | 0.1177 | 0.1699 | 0.902 | 0.1216 | 0.647258 | low |
| TCGA-A3-3351 | 0.4251 | 0.1965 | 0.1419 | 0.3587 | 1 | 0.0611 | 0.597916 | low |
| TCGA-B0-4710 | 0.3386 | 0.2598 | 0.1391 | 0.5855 | 0.9389 | 0.169 | 1.52932 | high |
| TCGA-B0-5083 | 0.6565 | 0.2176 | 0.0369 | 0.5627 | 0.8499 | 0.01277 | 0.5759 | low |
| TCGA-BP-4803 | 0.4431 | 0.1606 | 0.1727 | 0.4005 | 0.8862 | 0 | 0.681786 | low |
| TCGA-CJ-4889 | 0.2804 | 0.2828 | 0.1421 | 0.3644 | 1 | 0.0518 | 0.91887 | high |
| TCGA-B0-5080 | 0.1284 | 0.346 | 0.1768 | 0.6747 | 0.7797 | 0.0363 | 3.518818 | high |
| TCGA-CW-5587 | 0.4999 | 0.1143 | 0.1141 | 0.124 | 0.8643 | 0 | 0.350879 | low |
| TCGA-A3-3376 | 0.2624 | 0.2119 | 0.3124 | 0.4505 | 0.8575 | 0.1114 | 1.91613 | high |
| TCGA-CJ-4897 | 0.6351 | 0.1102 | 0.1145 | 0.2652 | 0.7541 | 0.0121 | 0.435083 | low |
| TCGA-CW-6088 | 0.5055 | 0.1601 | 0.0801 | 0.2614 | 0.9421 | 0.0672 | 0.423471 | low |
| TCGA-B8-5552 | 0.3813 | 0.1672 | 0.1638 | 0.3081 | 1 | 0.0388 | 0.561917 | low |
| TCGA-CW-5589 | 0.4995 | 0.0694 | 0.0412 | 0.363 | 0.87284 | 0 | 0.382855 | low |
| TCGA-B0-4833 | 0.5453 | 0.2224 | 0.3544 | 0.4673 | 0.788 | 0.1747 | 1.745484 | high |
| TCGA-CZ-4859 | 0.8029 | 0.1571 | 0.0208 | 0.3172 | 0.9308 | 0.0209 | 0.229103 | low |
| TCGA-A3-3320 | 0.761 | 0.3274 | 0.2901 | 0.2633 | 0.8814 | 0.1014 | 0.718268 | low |
| TCGA-MM-A564 | 0.2954 | 0.1275 | 0.1679 | 0.7064 | 0.705333 | 0.3263 | 3.383435 | high |
| TCGA-AS-3777 | 0.835 | 0.1582 | 0.0986 | 0.2729 | 1 | 0.0274 | 0.207606 | low |
| TCGA-B0-5095 | 0.1715 | 0.3449 | 0.2634 | 0.5187 | 0.5533 | 0.0503 | 5.063041 | high |
| TCGA-BP-4795 | 0.4903 | 0.2121 | 0.1453 | 0.5599 | 1 | 0.2201 | 1.003423 | high |
| TCGA-BP-4331 | 0.5915 | 0.2227 | 0.2941 | 0.2737 | 0.748 | 0.0597 | 0.952054 | high |
| TCGA-A3-A6NJ | 0.4385 | 0.1566 | 0.1244 | 0.3565 | 0.85831 | 0.106 | 0.753877 | low |
| TCGA-B0-4701 | 0.5249 | 0.2347 | 0.1995 | 0.5627 | 0.6798 | 0.1414 | 1.860689 | high |
| TCGA-A3-3367 | 0.6539 | 0.1671 | 0.2801 | 0.1618 | 1 | 0.024 | 0.346901 | low |
| TCGA-B0-5402 | 0.4829 | 0.1374 | 0.2312 | 0.1766 | 0.697 | 0 | 0.727736 | low |
| TCGA-B0-5691 | 0.5077 | 0.1824 | 0.0834 | 0.1714 | 1 | 0.0304 | 0.324079 | low |
| TCGA-BP-4342 | 0.5823 | 0.154 | 0.2076 | 0.5823 | 0.8499 | 0.0781 | 0.918304 | high |
| TCGA-A3-3373 | 0.4975 | 0.2731 | 0.1405 | 0.4516 | 0.8214 | 0.023 | 0.962325 | high |
| TCGA-BP-4341 | 0.5976 | 0.2088 | 0.1878 | 0.5627 | 0.850513 | 0.4137 | 1.774768 | high |
| TCGA-CW-6090 | 0.427 | 0.0882 | 0.167 | 0.584 | 0.8094 | 0.0304 | 0.95591 | high |
| TCGA-A3-3307 | 0.5215 | 0.2363 | 0.2312 | 0.4107 | 0.869033 | 0.0957 | 0.992635 | high |
| TCGA-B2-3924 | 0.6113 | 0.503 | 0.3297 | 0.57576 | 0.8499 | 0.25359 | 3.289903 | high |
| TCGA-B0-5706 | 0.2974 | 0.213 | 0.0661 | 0.32191 | 0.856475 | 0 | 0.760574 | low |
| TCGA-BP-4963 | 0.5011 | 0.2069 | 0.2627 | 0.2535 | 1 | 0.0401 | 0.55984 | low |
| TCGA-BP-4343 | 0.2672 | 0.2181 | 0.2312 | 0.7782 | 0.7797 | 0.0173 | 2.600129 | high |
| TCGA-B0-5703 | 0.4165 | 0.1829 | 0.0567 | 0.3638 | 0.881411 | 0.0185 | 0.594896 | low |
| TCGA-CJ-6027 | 0.5543 | 0.1558 | 0.0681 | 0.4986 | 0.8214 | 0.0781 | 0.695854 | low |
| TCGA-B0-5108 | 0.387 | 0.2219 | 0.2312 | 0.5412 | 0.7218 | 0.0722 | 1.899538 | high |
| TCGA-BP-4965 | 0.5145 | 0.1513 | 0.1661 | 0.2491 | 0.934 | 0.0799 | 0.493833 | low |
| TCGA-A3-A6NL | 0.4606 | 0.1634 | 0.1627 | 0.3804 | 1 | 0.0263 | 0.525964 | low |
| TCGA-CJ-5672 | 0.5531 | 0.3236 | 0.0911 | 0.2729 | 0.8499 | 0 | 0.620632 | low |
| TCGA-B8-5158 | 0.4606 | 0.2892 | 0.199 | 0.3358 | 0.8947 | 0.0266 | 0.869877 | low |
| TCGA-CJ-4876 | 0.5475 | 0.2163 | 0.3755 | 0.407 | 0.785067 | 0.024 | 1.241227 | high |
| TCGA-B0-5115 | 0.5917 | 0.1347 | 0.2871 | 0.4739 | 0.6479 | 0.177 | 1.584187 | high |
| TCGA-CJ-4916 | 0.4836 | 0.193 | 0.2615 | 0.378 | 1 | 0.1536 | 0.826649 | low |
| TCGA-B0-4848 | 0.3981 | 0.3403 | 0.1307 | 0.3653 | 0.8947 | 0.0159 | 0.97181 | high |
| TCGA-B4-5377 | 0.7504 | 0.155 | 0.0613 | 0.3525 | 0.7905 | 0.0077 | 0.37079 | low |
| TCGA-B0-4712 | 0.0879 | 0.286 | 0.2312 | 0.8017 | 0.5602 | 0 | 6.496868 | high |
| TCGA-A3-3324 | 0.4889 | 0.2467 | 0.2609 | 0.5138 | 0.8415 | 0.0461 | 1.272298 | high |
| TCGA-CZ-5465 | 0.6983 | 0.1047 | 0.3326 | 0.2513 | 0.937 | 0.0659 | 0.434848 | low |
| TCGA-CZ-4860 | 0.09 | 0.046 | 0.3804 | 0.6881 | 0.586 | 0 | 4.088826 | high |
| TCGA-B0-5102 | 0.7339 | 0.1637 | 0.417 | 0.2047 | 0.883175 | 0.0691 | 0.57686 | low |
| TCGA-B0-5690 | 0.5135 | 0.1779 | 0.1037 | 0.3735 | 0.8575 | 0.0163 | 0.578817 | low |
| TCGA-BP-4160 | 0.5099 | 0.1692 | 0.042 | 0.2905 | 0.876414 | 0 | 0.419825 | low |
| TCGA-B0-4697 | 0.11 | 0.321 | 0.2962 | 0.8373 | 0.579172 | 0.2734 | 12.96251 | high |
| TCGA-AK-3454 | 0.6371 | 0.1632 | 0.2537 | 0.3088 | 0.739 | 0.0834 | 0.800492 | low |
| TCGA-B0-5693 | 0.6187 | 0.0614 | 0.0922 | 0.2211 | 0.8643 | 0.1014 | 0.339782 | low |
| TCGA-BP-4985 | 0.3941 | 0.2151 | 0.1492 | 0.5763 | 0.860922 | 0.0488 | 1.189246 | high |
| TCGA-B0-4718 | 0.592 | 0.2347 | 0.2918 | 0.4911 | 0.902 | 0.1886 | 1.229448 | high |
| TCGA-B0-5109 | 0.5318 | 0.2916 | 0.2312 | 0.7023 | 0.6798 | 0.1424 | 2.713471 | high |
| TCGA-A3-3359 | 0.5568 | 0.2491 | 0.1987 | 0.3398 | 1 | 0 | 0.518345 | low |
| TCGA-DV-5569 | 0.5726 | 0.1006 | 0.1686 | 0.2956 | 0.6536 | 0 | 0.662427 | low |
| TCGA-B2-A4SR | 0.5357 | 0.2553 | 0.1121 | 0.3794 | 0.6769 | 0.0804 | 1.113329 | high |
| TCGA-B0-4815 | 0.3291 | 0.3416 | 0.3517 | 0.3398 | 0.531 | 0.1433 | 4.386456 | high |
| TCGA-AK-3461 | 0.4696 | 0.2924 | 0.1662 | 0.4107 | 0.832 | 0.1389 | 1.269723 | high |
| TCGA-CJ-4892 | 0.6083 | 0.2002 | 0.2962 | 0.637 | 0.8499 | 0.192 | 1.545953 | high |
| TCGA-B0-5695 | 0.7207 | 0.1363 | 0.0459 | 0.3525 | 1 | 0.07 | 0.264188 | low |
| TCGA-BP-4975 | 0.4887 | 0.1022 | 0.1875 | 0.3543 | 0.8762 | 0.0288 | 0.575217 | low |
| TCGA-AK-3456 | 0.4948 | 0.4717 | 0.0972 | 0.1637 | 0.902 | 0.1079 | 0.904002 | high |
| TCGA-BP-4159 | 0.4236 | 0.2091 | 0.2083 | 0.4723 | 0.83765 | 0.0449 | 1.121631 | high |
| TCGA-CZ-5458 | 0.5214 | 0.1127 | 0.1957 | 0.2186 | 0.8214 | 0.0427 | 0.535276 | low |
| TCGA-AK-3431 | 0.4365 | 0.1777 | 0.0943 | 0.4144 | 1 | 0 | 0.49566 | low |
| TCGA-B8-A8YJ | 0.1106 | 0.2292 | 0.2369 | 0.6128 | 0.86716 | 0.0611 | 2.514722 | high |
| TCGA-BP-5182 | 0.5443 | 0.1399 | 0.1486 | 0.4419 | 1 | 0.0397 | 0.47169 | low |
| TCGA-B2-4101 | 0.5005 | 0.1615 | 0.2068 | 0.347 | 0.8004 | 0.0844 | 0.857916 | low |
| TCGA-CJ-5681 | 0.4431 | 0.3222 | 0.0618 | 0.0604 | 0.9063 | 0.7093 | 1.758681 | high |
| TCGA-BP-4994 | 0.7418 | 0.2206 | 0.1618 | 0.0583 | 0.9485 | 0.086 | 0.288248 | low |
| TCGA-CJ-4903 | 0.5735 | 0.1462 | 0.2987 | 0.4554 | 0.8762 | 0.0212 | 0.773828 | low |
| TCGA-BP-4760 | 0.7385 | 0.2838 | 0.1219 | 0.4075 | 1 | 0 | 0.389573 | low |
| TCGA-BP-5190 | 0.6577 | 0.2486 | 0.3681 | 0.2434 | 0.7956 | 0.0715 | 0.919736 | high |
| TCGA-B0-5710 | 0.3704 | 0.099 | 0.0847 | 0.4026 | 0.9189 | 0.0351 | 0.56976 | low |
| TCGA-CJ-4875 | 0.5179 | 0.2106 | 0.3282 | 0.4911 | 0.8643 | 0.1152 | 1.337616 | high |
| TCGA-CJ-4874 | 0.4703 | 0.0672 | 0.0311 | 0.2345 | 0.739 | 0 | 0.43158 | low |
| TCGA-B2-5641 | 0.5874 | 0.2273 | 0.0629 | 0.1035 | 1 | 0 | 0.256984 | low |
| TCGA-CW-5584 | 0.4182 | 0.2006 | 0.103 | 0.316 | 0.7797 | 0.0256 | 0.788456 | low |
| TCGA-B8-4143 | 0.3466 | 0.1687 | 0.5272 | 0.5928 | 0.7676 | 0.084 | 3.203028 | high |
| TCGA-B0-5120 | 0.5309 | 0.1703 | 0.2155 | 0.5027 | 1 | 0.0623 | 0.668516 | low |
| TCGA-EU-5905 | 0.6745 | 0.0345 | 0.1946 | 0.363 | 0.6506 | 0.0334 | 0.596653 | low |
| TCGA-BP-4338 | 0.2839 | 0.215 | 0.0699 | 0.2649 | 0.863533 | 0.0331 | 0.760976 | low |
| TCGA-A3-3358 | 0.4373 | 0.187 | 0.1561 | 0.2324 | 0.902 | 0.024 | 0.562027 | low |
| TCGA-B0-4818 | 0.5226 | 0.2347 | 0.2303 | 0.4169 | 0.7124 | 0.1583 | 1.550293 | high |
| TCGA-BP-4976 | 0.5858 | 0.0527 | 0.0556 | 0.1641 | 0.9189 | 0 | 0.223311 | low |
| TCGA-BP-4345 | 0.1023 | 0.4937 | 0.239 | 0.6247 | 1 | 0.198 | 4.58197 | high |
| TCGA-CW-6093 | 0.5971 | 0.1273 | 0.1132 | 0.3915 | 0.7676 | 0 | 0.548204 | low |
| TCGA-BP-4330 | 0.5994 | 0.1785 | 0.1659 | 0.5174 | 0.937 | 0.1067 | 0.696547 | low |
| TCGA-EU-5906 | 0.4341 | 0.1191 | 0.0637 | 0.2768 | 0.8426 | 0.0251 | 0.492297 | low |
| TCGA-B0-4846 | 0.465 | 0.2676 | 0.2383 | 0.349 | 0.8176 | 0.0449 | 1.099679 | high |
| TCGA-CJ-6030 | 0.2854 | 0.249 | 0.1875 | 0.2633 | 0.761 | 0.0351 | 1.271964 | high |
| TCGA-BP-4983 | 0.0284 | 0.1284 | 0.3214 | 0.5412 | 0.902 | 0.084 | 2.38928 | high |
| TCGA-B0-5697 | 0.5992 | 0.2847 | 0.0959 | 0.4502 | 0.8415 | 0 | 0.697488 | low |
| TCGA-BP-4346 | 0.5444 | 0.3208 | 0.1914 | 0.3449 | 0.7905 | 0.1865 | 1.353988 | high |
| TCGA-B0-4691 | 0.4862 | 0.4044 | 0.2109 | 0.6044 | 0.7797 | 0.0894 | 2.310923 | high |
| TCGA-A3-3328 | 0.9536 | 0.5486 | 0.1621 | 0.0499 | 0.9465 | 0.0534 | 0.385819 | low |
| TCGA-BP-4765 | 0.5167 | 0.174 | 0.2042 | 0.3832 | 0.8499 | 0.059 | 0.773113 | low |
| TCGA-CZ-4858 | 0.06 | 0.2945 | 0.3981 | 0.2785 | 1 | 0.0611 | 2.025603 | high |
| TCGA-B0-5117 | 0.868 | 0.4083 | 0.2533 | 0.3554 | 1 | 0.06817 | 0.55769 | low |
| TCGA-CZ-4865 | 0.4971 | 0.3953 | 0.3898 | 0.2868 | 1 | 0.0438 | 1.152325 | high |
| TCGA-CJ-6028 | 0.3176 | 0.1124 | 0.1074 | 0.2434 | 1 | 0 | 0.422061 | low |
| TCGA-6D-AA2E | 0.4046 | 0.3789 | 0.0772 | 0.2404 | 0.80865 | 0.1917 | 1.313992 | high |
| TCGA-CJ-4884 | 0.5279 | 0.2697 | 0.359 | 0.3787 | 0.9396 | 0.0304 | 0.985404 | high |
| TCGA-DV-5568 | 0.4134 | 0.2655 | 0.1954 | 0.378 | 0.847543 | 0 | 0.991208 | high |
| TCGA-BP-4961 | 0.4565 | 0.1125 | 0.1401 | 0.2729 | 0.6389 | 0.1355 | 1.011886 | high |
| TCGA-BP-4761 | 0.6096 | 0.2543 | 0.0859 | 0.8278 | 0.8146 | 0.0191 | 1.185869 | high |
| TCGA-CJ-5677 | 0.3098 | 0.3549 | 0.2028 | 0.4237 | 0.79722 | 0.0212 | 1.805409 | high |
| TCGA-CZ-5460 | 0.5347 | 0.2486 | 0.1307 | 0.2161 | 0.832 | 0.0304 | 0.593409 | low |
| TCGA-CJ-4900 | 0.3238 | 0.2537 | 0.2832 | 0.2785 | 1 | 0.1414 | 1.097901 | high |
| TCGA-CZ-5451 | 0.5639 | 0.0988 | 0.0187 | 0.0569 | 0.88335 | 0.0424 | 0.239025 | low |
| TCGA-B8-A54F | 0.5056 | 0.1807 | 0.1185 | 0.4577 | 0.844375 | 0.1152 | 0.848763 | low |
| TCGA-B0-4838 | 0.4499 | 0.4563 | 0.4417 | 0.6002 | 0.8094 | 0.0468 | 3.707657 | high |
| TCGA-B0-4699 | 0.435 | 0.4915 | 0.3293 | 0.6157 | 0.8838 | 0.137 | 3.436641 | high |
| TCGA-BP-4781 | 0.5237 | 0.1475 | 0.064 | 0.1386 | 1 | 0.0173 | 0.261752 | low |
| TCGA-CJ-4644 | 0.4676 | 0.2512 | 0.0362 | 0.3449 | 0.8862 | 0 | 0.566786 | low |
| TCGA-B0-5701 | 0.3771 | 0.2415 | 0.0859 | 0 | 0.828525 | 0 | 0.489649 | low |
| TCGA-B0-5694 | 0.5102 | 0.2347 | 0.0617 | 0.3915 | 0.859438 | 0.0468 | 0.658792 | low |
| TCGA-B8-4621 | 0.6684 | 0.3855 | 0.1472 | 0.4563 | 0.886322 | 0.024 | 0.820943 | low |
| TCGA-BP-5006 | 0.5357 | 0.0926 | 0.2774 | 0.3228 | 0.85097 | 0.0416 | 0.637317 | low |
| TCGA-BP-5181 | 0.7101 | 0.0332 | 0.0527 | 0.4543 | 1 | 0.013 | 0.225775 | low |
| TCGA-B0-5709 | 0.3375 | 0.1084 | 0.0873 | 0.3002 | 0.9189 | 0.0288 | 0.527582 | low |
| TCGA-BP-4167 | 0.1545 | 0.2415 | 0.23522 | 0.6291 | 1 | 0.0659 | 1.871163 | high |
| TCGA-CJ-4638 | 0.6137 | 0.2216 | 0.575 | 0.7202 | 0.7106 | 0.0957 | 3.450325 | high |
| TCGA-EU-5907 | 0.6868 | 0.1074 | 0.1192 | 0.31289 | 0.7586 | 0.0524 | 0.457042 | low |
| TCGA-CJ-4869 | 0.4121 | 0.167 | 0.1963 | 0.6002 | 0.8643 | 0.0107 | 1.086054 | high |
| TCGA-3Z-A93Z | 0.4938 | 0.1165 | 0.0913 | 0.3362 | 0.8416 | 0.0315 | 0.514693 | low |
| TCGA-BP-5196 | 0.3097 | 0.2947 | 0.4004 | 0.6397 | 0.856067 | 0.0468 | 2.928664 | high |
| TCGA-AK-3433 | 0.9197 | 0.3071 | 0.176 | 0.24891 | 1 | 0 | 0.265516 | low |
| TCGA-A3-3346 | 0.1409 | 0.2087 | 0.1982 | 0.5587 | 0.6769 | 0.1034 | 3.153769 | high |
| TCGA-AK-3440 | 0.912 | 0.1751 | 0.2463 | 0.0387 | 0.9083 | 0 | 0.205961 | low |
| TCGA-B8-A54D | 0.5239 | 0.4235 | 0.3041 | 0.45264 | 0.7963 | 0.1558 | 2.378964 | high |
| TCGA-BP-5200 | 0.5307 | 0.1451 | 0.0773 | 0.4178 | 0.9421 | 0.0085 | 0.437527 | low |
| TCGA-B0-5400 | 0.1785 | 0.4682 | 0.6005 | 0.5627 | 0.586 | 0.0444 | 12.36201 | high |
| TCGA-BP-4969 | 0.6237 | 0.2071 | 0.2933 | 0.349 | 0.874 | 0.4001 | 1.413907 | high |
| TCGA-BP-4756 | 0.7839 | 0.3607 | 0.1703 | 0.0183 | 1 | 0.0894 | 0.316923 | low |
| TCGA-BP-4776 | 0.4728 | 0.1709 | 0.2446 | 0.4379 | 0.84335 | 0.1917 | 1.259841 | high |
| TCGA-CJ-4912 | 0.3631 | 0.2028 | 0.2739 | 0.7128 | 0.755757 | 0.1466 | 2.825547 | high |
| TCGA-CZ-5985 | 0.6519 | 0.1137 | 0.1857 | 0.0439 | 0.7797 | 0.0281 | 0.347253 | low |
| TCGA-B0-5098 | 0.2393 | 0.4208 | 0.5239 | 0.2905 | 0.5756 | 0.0826 | 6.427458 | high |
| TCGA-B8-5545 | 0.4063 | 0.1896 | 0.0635 | 0.526 | 0.9308 | 0 | 0.684898 | low |
| TCGA-BP-4758 | 0.5412 | 0.1179 | 0.2379 | 0.4739 | 0.7676 | 0.0363 | 0.905559 | high |
| TCGA-B0-4696 | 0.0218 | 0.4631 | 0.1528 | 0.7528 | 0.8762 | 0.18 | 6.265128 | high |
| TCGA-A3-3322 | 0.6174 | 0.1387 | 0.2 | 0.586 | 0.586 | 0.0168 | 1.278544 | high |
| TCGA-B0-5107 | 0.4278 | 0.3022 | 0.3036 | 0.3638 | 0.8272 | 0.1062 | 1.612037 | high |
| TCGA-DV-5565 | 0.3921 | 0.3404 | 0.2312 | 0.4661 | 0.7901 | 0.03692 | 1.781829 | high |
| TCGA-CZ-5989 | 0.5865 | 0.1889 | 0.3022 | 0.512 | 0.9233 | 0.061 | 0.88731 | high |
| TCGA-BP-4173 | 0.3032 | 0.2233 | 0.1389 | 0.3915 | 0.8415 | 0.0732 | 1.159762 | high |
| TCGA-CJ-4870 | 0.7089 | 0.0858 | 0.0393 | 0.2105 | 0.8705 | 0.0508 | 0.24523 | low |
| TCGA-BP-4974 | 0.4324 | 0.1766 | 0.3985 | 0.4943 | 0.8444 | 0.2477 | 2.210709 | high |
| TCGA-B0-5075 | 0.6203 | 0.1487 | 0.3545 | 0.1574 | 0.6389 | 0.0606 | 0.919544 | high |
| TCGA-BP-4162 | 0.4763 | 0.1812 | 0.2279 | 0.3554 | 0.586 | 0.025 | 1.375897 | high |
| TCGA-B0-4839 | 0.4649 | 0.1897 | 0.1219 | 0.7202 | 0.804786 | 0.0659 | 1.34892 | high |
| TCGA-B8-5553 | 0.5539 | 0.115 | 0.0559 | 0.2161 | 0.832 | 0.0154 | 0.359981 | low |
| TCGA-CJ-4893 | 0.3887 | 0.2879 | 0.134 | 0.2926 | 0.7797 | 0.051 | 1.082575 | high |
| TCGA-CJ-6033 | 0.3284 | 0.1497 | 0.281 | 0.5096 | 1 | 0.0221 | 0.960328 | high |
| TCGA-B0-4827 | 0.333 | 0.4864 | 0.0586 | 0.3695 | 1 | 0 | 1.023747 | high |
| TCGA-BP-4977 | 0.5798 | 0.2561 | 0.2081 | 0.3433 | 1 | 0.057 | 0.576546 | low |
| TCGA-BP-5187 | 0.4099 | 0.2141 | 0.2504 | 0.4419 | 1 | 0.0321 | 0.838944 | low |
| TCGA-CJ-4882 | 0.2358 | 0.4627 | 0.1895 | 0.495 | 0.815929 | 0.1389 | 3.37062 | high |
| TCGA-CJ-4899 | 0.4511 | 0.2153 | 0.1397 | 0.4003 | 0.8094 | 0.1237 | 1.054385 | high |
| TCGA-B8-4620 | 0.5479 | 0.2128 | 0.3601 | 0.6202 | 0.6798 | 0.0906 | 2.291529 | high |
| TCGA-CJ-4868 | 0.1932 | 0.3151 | 0.3306 | 0.4654 | 0.7023 | 0.0659 | 3.635363 | high |
| TCGA-BP-5001 | 0.6382 | 0.3072 | 0.298 | 0.6587 | 0.7797 | 0.059 | 1.734782 | high |
| TCGA-DV-5575 | 0.5721 | 0.1611 | 0.0833 | 0.2224 | 0.6798 | 0.0304 | 0.580293 | low |
| TCGA-CJ-4920 | 0.438 | 0.2502 | 0.3229 | 0.3335 | 1 | 0.0534 | 0.886531 | high |
| TCGA-B0-4707 | 0.6428 | 0.4083 | 0.2515 | 0.895 | 0.2614 | 0.1114 | 8.97478 | high |
| TCGA-AK-3426 | 0.2596 | 0.4073 | 0.2527 | 0.8428 | 0.7022 | 0.2974 | 9.106332 | high |
| TCGA-B0-4713 | 0.5722 | 0.2986 | 0.7386 | 0.7023 | 0.7781 | 0.1784 | 5.951744 | high |
| TCGA-B0-5705 | 0.612 | 0.1094 | 0.0668 | 0.4502 | 1 | 0.0427 | 0.341411 | low |
| TCGA-BP-5199 | 0.4238 | 0.3051 | 0.2057 | 0.5627 | 0.7124 | 0.1437 | 2.454835 | high |
| TCGA-CZ-5988 | 0.5075 | 0.1798 | 0.0274 | 0.4093 | 1 | 0.02175 | 0.400861 | low |
| TCGA-BP-5004 | 0.5706 | 0.0912 | 0.2727 | 0.2926 | 1 | 0.0746 | 0.443006 | low |
| TCGA-A3-3370 | 0.5166 | 0.1854 | 0.3169 | 0.3206 | 0.8575 | 0.0478 | 0.867547 | low |
| TCGA-B0-5696 | 0.3479 | 0.1447 | 0.1013 | 0.3535 | 0.8499 | 0.0198 | 0.706893 | low |
| TCGA-BP-4797 | 0.566 | 0.287 | 0.168 | 0.3002 | 0.8444 | 0.0221 | 0.712043 | low |
| TCGA-CZ-5982 | 0.5925 | 0.2386 | 0.105 | 0.349 | 0.8499 | 0.023 | 0.575413 | low |
| TCGA-BP-4991 | 0.5377 | 0.1325 | 0.2126 | 0.2819 | 0.9444 | 0.1715 | 0.606884 | low |
| TCGA-BP-4762 | 0.3759 | 0.0864 | 0.1168 | 0.4079 | 0.7956 | 0.0416 | 0.770006 | low |
| TCGA-A3-3362 | 0.5181 | 0.1599 | 0.2724 | 0.2814 | 0.761 | 0.0971 | 0.952082 | high |
| TCGA-A3-A8OW | 0.5422 | 0.1691 | 0.109 | 0.2968 | 0.85435 | 0.1095 | 0.58656 | low |
| TCGA-CJ-4872 | 0.2834 | 0.1626 | 0.1739 | 0.5296 | 0.9233 | 0.2067 | 1.484587 | high |
| TCGA-B0-4847 | 0.4208 | 0.3151 | 0.3772 | 0.6587 | 0.744417 | 0.2354 | 4.478231 | high |
| TCGA-B0-4822 | 0.1981 | 0.1833 | 0.1757 | 0.6006 | 1 | 0.3381 | 2.182161 | high |
| TCGA-BP-5175 | 0.5109 | 0.3194 | 0.4526 | 0.4911 | 0.7228 | 0.1583 | 3.183937 | high |
| TCGA-BP-4340 | 0.5939 | 0.2412 | 0.2438 | 0.2917 | 0.8094 | 0.0766 | 0.835771 | low |
| TCGA-BP-4987 | 0.758 | 0.158 | 0.2372 | 0.3818 | 0.761 | 0.0201 | 0.586734 | low |
| TCGA-BP-5189 | 0.3819 | 0.3358 | 0.2334 | 0.3726 | 0.9503 | 0.0388 | 1.132787 | high |
| TCGA-BP-5000 | 0.483 | 0.3214 | 0.1746 | 0.6587 | 0.8499 | 0.0262 | 1.496415 | high |
| TCGA-AK-3451 | 0.6471 | 0.2016 | 0.11273 | 0.39203 | 0.908967 | 0.00859 | 0.448716 | low |
| TCGA-BP-5007 | 0.567 | 0.1623 | 0.2253 | 0.2883 | 0.8732 | 0.1507 | 0.710936 | low |
| TCGA-A3-3363 | 0.7712 | 0.4721 | 0.1753 | 0.114 | 0.7064 | 0.0321 | 0.793956 | low |
| TCGA-AK-3455 | 0.5759 | 0.2197 | 0.1751 | 0.5627 | 1 | 0 | 0.618992 | low |
| TCGA-B8-4146 | 0.6024 | 0.1592 | 0.1307 | 0.37009 | 0.9444 | 0 | 0.404478 | low |
| TCGA-BP-4770 | 0.0036 | 0.3027 | 0.2161 | 0.7309 | 0.832 | 0 | 3.880077 | high |
| TCGA-A3-3378 | 0.2789 | 0.4998 | 0.2271 | 0.3621 | 0.8176 | 0.1041 | 2.815869 | high |
| TCGA-DV-5573 | 0.5732 | 0.2175 | 0.195 | 0.4911 | 1 | 0.015 | 0.596723 | low |
| TCGA-BP-4962 | 0.5053 | 0.2214 | 0.1339 | 0.3675 | 0.832 | 0.0163 | 0.715949 | low |
| TCGA-B8-5546 | 0.6256 | 0.3427 | 0.0492 | 0.0447 | 0.832 | 0.148 | 0.520964 | low |
| TCGA-BP-4347 | 0.4768 | 0.2933 | 0.2912 | 0.3525 | 0.8598 | 0.1193 | 1.337048 | high |
| TCGA-A3-3343 | 0.6352 | 0.1245 | 0.1999 | 0.6457 | 0.8176 | 0.0561 | 0.870347 | low |
| TCGA-CJ-4918 | 0.3518 | 0.1501 | 0.2485 | 0.3375 | 1 | 0.1014 | 0.786292 | low |
| TCGA-BP-5201 | 0.4984 | 0.1906 | 0.2058 | 0.2688 | 0.8862 | 0.07 | 0.666568 | low |
| TCGA-CZ-5459 | 0.5097 | 0.0968 | 0.0852 | 0.1743 | 0.880225 | 0.0251 | 0.342347 | low |
| TCGA-BP-5170 | 0.5743 | 0.0455 | 0.1307 | 0.55 | 1 | 0.0401 | 0.410895 | low |
| TCGA-B0-5094 | 0.4481 | 0.2514 | 0.3696 | 0.3149 | 0.4144 | 0.0555 | 3.163164 | high |
| TCGA-BP-4973 | 0.5178 | 0.1851 | 0.2239 | 0.3002 | 0.8214 | 0.0746 | 0.796573 | low |
| TCGA-CJ-5684 | 0.5275 | 0.0523 | 0.0394 | 0.3915 | 1 | 0.0432 | 0.303658 | low |
| TCGA-BP-4771 | 0.3075 | 0.4098 | 0.2845 | 0.5352 | 0.8947 | 0.1747 | 3.062763 | high |
| TCGA-B0-5116 | 0.54 | 0.1712 | 0.1902 | 0.4855 | 0.7124 | 0.0212 | 1.032392 | high |
| TCGA-CW-5585 | 0.6557 | 0.0804 | 0.082 | 0.2764 | 0.9233 | 0.0334 | 0.274715 | low |
| TCGA-CJ-4881 | 0.3594 | 0.3566 | 0.3108 | 0.7827 | 0.852944 | 0.1355 | 3.790658 | high |
| TCGA-A3-3323 | 0.521 | 0.1549 | 0.3047 | 0.7379 | 1 | 0.1678 | 1.334726 | high |
| TCGA-A3-3380 | 0.5809 | 0.1382 | 0.3483 | 0.5145 | 0.8643 | 0.0965 | 1.062052 | high |
| TCGA-B0-4834 | 0.8463 | 0.331 | 0.4306 | 0.1201 | 0.9444 | 0.04439 | 0.525502 | low |
| TCGA-AK-3450 | 0.4694 | 0.273 | 0.1149 | 0.2614 | 1 | 0.0341 | 0.514678 | low |
| TCGA-CZ-5461 | 0.3461 | 0.0908 | 0.0303 | 0.3915 | 0.9189 | 0.0225 | 0.504951 | low |
| TCGA-A3-3347 | 0.3111 | 0.3209 | 0.4432 | 0.5224 | 0.9083 | 0.1466 | 3.073263 | high |
| TCGA-B0-5110 | 0.5434 | 0.211 | 0.2822 | 0.5702 | 1 | 0.0957 | 0.954961 | high |
| TCGA-AK-3460 | 0.587 | 0.0837 | 0.2668 | 0.369 | 0.9139 | 0.0274 | 0.511762 | low |
| TCGA-CJ-4908 | 0.3928 | 0.254 | 0.258 | 0.4566 | 0.8214 | 0.0962 | 1.600235 | high |
| TCGA-BP-4774 | 0.5906 | 0.1371 | 0.5167 | 0.6166 | 0.8862 | 0.1334 | 1.708824 | high |
| TCGA-B0-4842 | 0.2404 | 0.286 | 0.17784 | 0.6318 | 0.6305 | 0.03901 | 3.286358 | high |
| TCGA-B0-5812 | 0.4306 | 0.0656 | 0.084 | 0.3721 | 0.8705 | 0 | 0.472378 | low |
| TCGA-B0-4816 | 0.2807 | 0.271 | 0.3511 | 0.3002 | 0.7541 | 0.0449 | 2.009354 | high |
| TCGA-BP-4988 | 0.2296 | 0.4079 | 0.2555 | 0.6318 | 0.7872 | 0.3377 | 6.44054 | high |
| TCGA-B0-4945 | 0.4313 | 0.2359 | 0.3939 | 0.2754 | 0.872689 | 0.1583 | 1.455307 | high |
| TCGA-DV-5576 | 0.7266 | 0.3029 | 0.1498 | 0.349 | 0.902 | 0.0143 | 0.506842 | low |
| TCGA-MM-A84U | 0.4306 | 0.3099 | 0.1272 | 0.4472 | 0.831557 | 0.0647 | 1.200796 | high |
| TCGA-BP-4998 | 0.4457 | 0.3448 | 0.1503 | 0.2555 | 0.952 | 0.1277 | 0.878669 | low |
| TCGA-A3-3387 | 0.4095 | 0.1427 | 0.2507 | 0.4049 | 0.8947 | 0.0575 | 0.888753 | high |
| TCGA-BP-4968 | 0.58 | 0.2191 | 0.2697 | 0.4911 | 0.9272 | 0.0474 | 0.845275 | low |
| TCGA-BP-4995 | 0.6784 | 0.2014 | 0.1541 | 0.1652 | 0.8094 | 0.0185 | 0.416439 | low |
| TCGA-CJ-4890 | 0.1584 | 0.3129 | 0.2382 | 0.5627 | 0.8094 | 0.0534 | 2.889488 | high |
| TCGA-B0-5092 | 0.307 | 0.342 | 0.3858 | 0.4768 | 0.6798 | 0.0547 | 3.681063 | high |
| TCGA-DV-A4VZ | 0.746 | 0.2661 | 0.2454 | 0.2087 | 0.85576 | 0.094 | 0.568102 | low |
| TCGA-A3-3385 | 0.5927 | 0.1623 | 0.2123 | 0.39918 | 0.8643 | 0.0416 | 0.645514 | low |
| TCGA-B0-4823 | 0.491 | 0.0707 | 0.0465 | 0.3325 | 0.832 | 0.0727 | 0.470301 | low |
| TCGA-BP-4992 | 0.3248 | 0.5542 | 0.2998 | 0.73175 | 0.804575 | 0.0561 | 5.390632 | high |
| TCGA-B8-4151 | 0.7246 | 0.0842 | 0.2312 | 0.1386 | 1 | 0.023 | 0.224874 | low |
| TCGA-B4-5838 | 0.5919 | 0.1486 | 0.1361 | 0.3554 | 0.832 | 0 | 0.502999 | low |
| TCGA-BP-4986 | 0.3713 | 0.1133 | 0.3789 | 0.2739 | 0.7905 | 0.0566 | 1.169265 | high |
| TCGA-CZ-5984 | 0.5871 | 0.1656 | 0.1651 | 0.4093 | 0.8762 | 0.0198 | 0.569548 | low |
| TCGA-BP-4344 | 0.4279 | 0.1173 | 0.2695 | 0.5007 | 0.86593 | 0.0894 | 1.092371 | high |
| TCGA-CZ-4854 | 0.5316 | 0.2235 | 0.4372 | 0.407 | 0.7956 | 0.0971 | 1.638297 | high |
| TCGA-CJ-6032 | 0.5378 | 0.2392 | 0.0585 | 0.2489 | 0.8862 | 0.0321 | 0.472666 | low |
| TCGA-BP-4355 | 0.5338 | 0.2565 | 0.206 | 0.6224 | 0.7023 | 0.3111 | 2.786349 | high |
| TCGA-B0-4814 | 0.486 | 0.1794 | 0.1729 | 0.4768 | 0.2981 | 0.0328 | 2.668484 | high |
| TCGA-BP-4759 | 0.5295 | 0.0575 | 0.1343 | 0.4809 | 0.8947 | 0 | 0.47965 | low |
| TCGA-B0-5097 | 0.1776 | 0.2985 | 0.2419 | 0.6547 | 1 | 0.0205 | 1.967708 | high |
| TCGA-CJ-4923 | 0.1711 | 0.2595 | 0.3025 | 0.5174 | 0.8205 | 0.2126 | 3.513684 | high |
| TCGA-DV-A4VX | 0.658 | 0.4567 | 0.1044 | 0.50093 | 0.81366 | 0.1311 | 1.366692 | high |
| TCGA-CJ-5671 | 0.2263 | 0.3853 | 0.0973 | 0.4974 | 0.817011 | 0 | 1.864409 | high |
| TCGA-BP-5191 | 0.4982 | 0.1899 | 0.30971 | 0.6389 | 0.7041 | 0.06835 | 2.016566 | high |
| TCGA-BP-4158 | 0.6003 | 0.097 | 0.1483 | 0.3596 | 0.8499 | 0.0221 | 0.457685 | low |
| TCGA-BP-4768 | 0.6759 | 0.1376 | 0.2312 | 0.2848 | 0.7956 | 0.1207 | 0.624362 | low |
| TCGA-B2-5639 | 0.4188 | 0.2565 | 0.0477 | 0.1944 | 0.863571 | 0 | 0.538215 | low |
| TCGA-B0-5084 | 0.5303 | 0.1456 | 0.5261 | 0.7982 | 0.6937 | 0.0623 | 3.334286 | high |
| TCGA-CJ-5680 | 0.6839 | 0.2407 | 0.129 | 0.1643 | 1 | 0 | 0.278002 | low |
| TCGA-CJ-4639 | 0.5933 | 0.11 | 0.3276 | 0.3915 | 0.8094 | 0.0416 | 0.795349 | low |
| TCGA-B8-5159 | 0.6559 | 0.1019 | 0.0965 | 0.1023 | 1 | 0.0354 | 0.19724 | low |
| TCGA-CJ-4641 | 0.4103 | 0.2979 | 0.4172 | 0.3771 | 0.8094 | 0.0965 | 2.126638 | high |
| TCGA-B0-5692 | 0.5016 | 0.1168 | 0.1141 | 0.1829 | 0.586 | 0.0427 | 0.740798 | low |
| TCGA-CJ-4895 | 0.3499 | 0.331 | 0.2312 | 0.5174 | 0.8724 | 0.0262 | 1.66774 | high |
| TCGA-BP-4959 | 0.6766 | 0.1682 | 0.1519 | 0.2434 | 1 | 0 | 0.2809 | low |
| TCGA-BP-4787 | 0.3647 | 0.4151 | 0.3876 | 0.5894 | 0.5149 | 0.1448 | 7.720662 | high |
| TCGA-AK-3465 | 0.7622 | 0.4214 | 0.3123 | 0.1065 | 1 | 0.1583 | 0.638766 | low |
| TCGA-BP-5009 | 0.4371 | 0.3382 | 0.3974 | 0.4169 | 0.832 | 0.059 | 2.011605 | high |
| TCGA-EU-5904 | 0.5385 | 0.074 | 0.0628 | 0.2004 | 0.86674 | 0.0551 | 0.335328 | low |
| TCGA-A3-3306 | 0.6983 | 0.1852 | 0.2248 | 0.5174 | 1 | 0.1014 | 0.580211 | low |
| TCGA-BP-5192 | 0.6901 | 0.0834 | 0.106 | 0.369 | 1 | 0 | 0.249643 | low |
| TCGA-BP-4784 | 0.6674 | 0.1025 | 0.085 | 0.559 | 0.902 | 0 | 0.41905 | low |
| TCGA-B8-4153 | 0.5519 | 0.0937 | 0.123 | 0.4457 | 0.8722 | 0.0906 | 0.577993 | low |
| TCGA-B0-5081 | 0.2311 | 0.2775 | 0.4612 | 0.5736 | 0.761 | 0.186 | 5.224928 | high |
| TCGA-G6-A8L6 | 0.5395 | 0.0879 | 0.1627 | 1 | 0.7804 | 0.0579 | 1.58926 | high |
| TCGA-AK-3445 | 0.1661 | 0.3046 | 0.3026 | 0.4093 | 0.6536 | 0.0906 | 3.771335 | high |
| TCGA-BP-4351 | 0.3705 | 0.4122 | 0.2951 | 0.6789 | 0.7124 | 0.1373 | 4.724709 | high |
| TCGA-B0-5100 | 0.627 | 0.2712 | 0.5658 | 0.736 | 0.804 | 0.0534 | 2.874654 | high |
| TCGA-B0-5700 | 0.5757 | 0.046 | 0.0456 | 0.4288 | 0.8191 | 0.0468 | 0.431994 | low |
| TCGA-AK-3429 | 0.5094 | 0.1669 | 0.0266 | 0.586 | 0.6798 | 0 | 0.934813 | high |
| TCGA-BP-4798 | 0.1089 | 0.4431 | 0.376 | 0.8314 | 0.7905 | 0.1784 | 10.58453 | high |
| TCGA-BP-4769 | 0.6177 | 0.3608 | 0.2527 | 0.6642 | 0.9139 | 0.0341 | 1.348064 | high |
| TCGA-CJ-5678 | 0.4099 | 0.0672 | 0.15916 | 0.3915 | 0.8112 | 0 | 0.66039 | low |
| TCGA-A3-3316 | 0.2415 | 0.2273 | 0.337 | 0.5296 | 0.8499 | 0.1389 | 2.582603 | high |
| TCGA-B8-5550 | 0.3572 | 0.2857 | 0.0933 | 0.3023 | 1 | 0 | 0.612219 | low |
| TCGA-B0-5699 | 0.6443 | 0.0951 | 0.1155 | 0.117 | 0.866178 | 0.031 | 0.275185 | low |
| TCGA-CJ-4888 | 0.1367 | 0.4174 | 0.3823 | 0.4592 | 0.7676 | 0.086 | 4.981451 | high |
| TCGA-BP-4354 | 0.1218 | 0.3427 | 0.3202 | 0.48 | 0.8575 | 0.0746 | 3.216235 | high |
| TCGA-BP-4989 | 0.284 | 0.3865 | 0.3712 | 0.5537 | 0.761 | 0.2331 | 5.419736 | high |
| TCGA-CJ-4873 | 0.3703 | 0.2347 | 0.4265 | 0.3335 | 0.803133 | 0.2025 | 2.343279 | high |
| TCGA-B0-4824 | 0.5864 | 0.2905 | 0.3491 | 0.2434 | 0.7218 | 0.2201 | 1.695512 | high |
| TCGA-CZ-5455 | 0.4871 | 0.118 | 0.1549 | 0.229 | 0.6606 | 0.0341 | 0.741352 | low |
| TCGA-BP-4164 | 0.7132 | 0.2017 | 0.2749 | 0.4617 | 0.833457 | 0.0375 | 0.747775 | low |
| TCGA-CW-5581 | 0.53 | 0.1729 | 0.0836 | 0.1962 | 0.9139 | 0 | 0.357775 | low |
| TCGA-A3-3313 | 0.7359 | 0.4681 | 0.391 | 0.1104 | 0.9503 | 0.1237 | 0.900622 | high |
| TCGA-B0-4845 | 0.5677 | 0.3522 | 0.3397 | 0.5346 | 0.7541 | 0.1277 | 2.352684 | high |
| TCGA-B4-5378 | 0.7301 | 0.1682 | 0.1505 | 0.2327 | 0.9444 | 0.0296 | 0.298767 | low |
| TCGA-BP-5174 | 0.6827 | 0.3298 | 0.2097 | 0.2822 | 0.862989 | 0.086 | 0.733842 | low |
| TCGA-BP-4801 | 0.4939 | 0.0429 | 0.0884 | 0.3638 | 1 | 0 | 0.306719 | low |
| TCGA-CJ-5686 | 0.4429 | 0.1811 | 0.0977 | 0.0985 | 0.6089 | 0.0552 | 0.790936 | low |
| TCGA-A3-3349 | 0.4227 | 0.2042 | 0.167 | 0.4002 | 0.697 | 0.0686 | 1.296849 | high |
| TCGA-DV-5567 | 0.8146 | 0.139 | 0.0496 | 0.1512 | 1 | 0.0077 | 0.151705 | low |
| TCGA-CZ-5987 | 0.5516 | 0.2667 | 0.2109 | 0.2161 | 0.870913 | 0 | 0.609634 | low |
| TCGA-CJ-4885 | 0.4296 | 0.2358 | 0.2312 | 0.1674 | 1 | 0 | 0.520179 | low |
| TCGA-DV-5574 | 0.1826 | 0.1741 | 0.0809 | 0.3721 | 0.8705 | 0.0363 | 0.977469 | high |
| TCGA-A3-3331 | 0.4146 | 0.2436 | 0.3999 | 0.1682 | 0.9421 | 0.0709 | 0.969635 | high |
| TCGA-BP-5178 | 0.3674 | 0.2833 | 0.1582 | 0.5145 | 0.851888 | 0.0159 | 1.290358 | high |
| TCGA-CJ-4871 | 0.4349 | 0.1452 | 0.1779 | 0.5914 | 0.902 | 0 | 0.859527 | low |
| TCGA-B0-4836 | 0.3091 | 0.3214 | 0.3248 | 0.4017 | 0.861278 | 0.0623 | 1.944784 | high |
| TCGA-B8-A54I | 0.3344 | 0.3582 | 0.2256 | 0.689 | 0.83735 | 0.1215 | 2.963667 | high |
| TCGA-B0-4821 | 0.3506 | 0.3817 | 0.2576 | 0.7554 | 0.586 | 0.2331 | 7.397215 | high |
| TCGA-BP-4777 | 0.5272 | 0.2884 | 0.247 | 0.3188 | 0.8499 | 0.0686 | 0.984978 | high |
| TCGA-CJ-4636 | 0.5182 | 0.2318 | 0.3755 | 0.3554 | 0.7797 | 0.086 | 1.425137 | high |
| TCGA-B0-5711 | 0.566 | 0.0749 | 0.0581 | 0.2573 | 0.7797 | 0.0146 | 0.382878 | low |
| TCGA-CZ-4857 | 0.2837 | 0.2065 | 0.2739 | 0.3726 | 0.902 | 0.0545 | 1.241068 | high |
| TCGA-B0-4852 | 0.4148 | 0.2613 | 0.4628 | 0.5456 | 0.7124 | 0.0534 | 3.055569 | high |
| TCGA-A3-3329 | 0.5737 | 0.2236 | 0.197 | 0.3915 | 0.859033 | 0.059 | 0.765953 | low |
| TCGA-CZ-5466 | 0.3326 | 0.2083 | 0.0943 | 0.4871 | 0.888078 | 0.0245 | 0.930192 | high |
| TCGA-BP-5177 | 0.5258 | 0.0856 | 0.089 | 0.5755 | 0.8762 | 0.0262 | 0.58881 | low |
| TCGA-A3-3372 | 0.431 | 0.2729 | 0.2655 | 0.2434 | 0.902 | 0.1747 | 1.143536 | high |
| TCGA-BP-4804 | 0.3309 | 0.1608 | 0.4014 | 0.634 | 1 | 0.0715 | 1.621318 | high |
| TCGA-AK-3434 | 0.6166 | 0.2251 | 0.1037 | 0.4911 | 0.804 | 0.1114 | 0.852172 | low |
| TCGA-B0-4811 | 0.3027 | 0.2684 | 0.6807 | 0.6359 | 0.788133 | 0.2832 | 8.575604 | high |
| TCGA-BP-4165 | 0.6136 | 0.1709 | 0.1424 | 0.5627 | 0.832 | 0.1152 | 0.862306 | low |
| TCGA-B0-5099 | 0.535 | 0.2749 | 0.2908 | 0.5967 | 0.6798 | 0.0603 | 2.147231 | high |
| TCGA-BP-4766 | 0.3805 | 0.2412 | 0.2214 | 0.2886 | 0.8705 | 0.1365 | 1.132754 | high |
| TCGA-BP-4335 | 0.5488 | 0.2771 | 0.4258 | 0.369 | 1 | 0.1424 | 1.179871 | high |
| TCGA-B0-4703 | 0.0774 | 0.2599 | 0.3564 | 0.6105 | 0.5482 | 0.0488 | 6.784263 | high |
| TCGA-CJ-4904 | 0.5341 | 0.1221 | 0.072 | 0.2844 | 0.8499 | 0.0375 | 0.432328 | low |
| TCGA-BP-4352 | 0.2439 | 0.4152 | 0.4216 | 0.7023 | 0.697 | 0.3457 | 11.9181 | high |
| TCGA-CJ-4635 | 0.5491 | 0.1805 | 0.2737 | 0.4694 | 1 | 0.0879 | 0.741472 | low |
| TCGA-CJ-5679 | 0.2571 | 0.3301 | 0.1483 | 0.6389 | 0.828625 | 0 | 2.066211 | high |
| TCGA-CZ-4862 | 0.4367 | 0.2832 | 0.0953 | 0.1726 | 0.8499 | 0 | 0.604964 | low |
| TCGA-B8-4622 | 0.4705 | 0.3151 | 0.1649 | 0.2868 | 1 | 0.0449 | 0.656936 | low |
| TCGA-G6-A8L8 | 0.6152 | 0.1405 | 0.2633 | 0.5587 | 0.817686 | 0.1051 | 1.019908 | high |
| TCGA-CJ-5682 | 0.4604 | 0.1633 | 0.2245 | 0.4502 | 0.761 | 0.02574 | 1.07635 | high |
| TCGA-BP-4176 | 0.6073 | 0.1956 | 0.1779 | 0.2434 | 0.8947 | 0.0416 | 0.475792 | low |
| TCGA-B8-4148 | 0.5561 | 0.2282 | 0.2899 | 0.3149 | 0.9139 | 0.1041 | 0.829405 | low |
| TCGA-CJ-4886 | 0.4277 | 0.1527 | 0.1243 | 0.3162 | 1 | 0.0449 | 0.476703 | low |
| TCGA-BP-5186 | 0.52 | 0.1779 | 0.1728 | 0.4472 | 1 | 0 | 0.523891 | low |
| TCGA-BP-4972 | 0.5576 | 0.1205 | 0.1823 | 0.3848 | 0.9396 | 0.0522 | 0.504101 | low |
| TCGA-BP-4993 | 0.6006 | 0.24 | 0.2312 | 0.4199 | 0.8947 | 0.0957 | 0.839326 | low |
| TCGA-CW-6097 | 0.3137 | 0.2799 | 0.1768 | 0.2147 | 0.855 | 0.0331 | 0.970619 | high |
| TCGA-B0-5077 | 0.5466 | 0.1463 | 0.2566 | 0.4223 | 1 | 0.0449 | 0.575882 | low |
| TCGA-A3-3383 | 0.5469 | 0.1865 | 0.1272 | 0.4418 | 0.78374 | 0 | 0.727061 | low |
| TCGA-B0-4819 | 0.2363 | 0.4327 | 0.4955 | 0.6233 | 0.6142 | 0.2439 | 12.66832 | high |
| TCGA-BP-4775 | 0.5705 | 0.1981 | 0.332 | 0.1601 | 0.9083 | 0.0631 | 0.615547 | low |
| TCGA-BP-4327 | 0.769 | 0.3263 | 0.217 | 0.4144 | 1 | 0.1466 | 0.648965 | low |
| TCGA-B0-5399 | 0.4975 | 0.2084 | 0.2196 | 0.6218 | 1 | 0.0416 | 0.885871 | high |
| TCGA-A3-3357 | 0.6643 | 0.2095 | 0.2634 | 0.1707 | 0.899833 | 0.0709 | 0.493889 | low |
| TCGA-B0-4694 | 0.5255 | 0.3151 | 0.0688 | 0.4966 | 0.832 | 0.024 | 0.916466 | high |
| TCGA-A3-3317 | 0.6646 | 0.4041 | 0.4149 | 0.2617 | 0.8814 | 0.0634 | 1.185254 | high |
| TCGA-B8-4154 | 0.6397 | 0.0906 | 0.0296 | 0.4107 | 0.9189 | 0 | 0.300138 | low |
| TCGA-B2-5636 | 0.6051 | 0.2635 | 0.0994 | 0.1702 | 0.6294 | 0 | 0.689859 | low |
| TCGA-B0-4817 | 0.3825 | 0.3543 | 0.2541 | 0.48413 | 0.7653 | 0 | 1.967776 | high |
| TCGA-BP-5010 | 0.181 | 0.37 | 0.4154 | 0.736 | 0.832 | 0.4847 | 12.22266 | high |
| TCGA-BP-4325 | 0.7361 | 0.2287 | 0.2807 | 0.4911 | 1 | 0.1308 | 0.678307 | low |
| TCGA-BP-5008 | 0.5778 | 0.1466 | 0.184 | 0.3802 | 1 | 0.0942 | 0.490603 | low |
| TCGA-B0-5707 | 0.7159 | 0.2634 | 0.0639 | 0.398 | 0.8302 | 0 | 0.486485 | low |
| TCGA-CZ-5454 | 0.5348 | 0.0748 | 0.1282 | 0.3915 | 1 | 0.0124 | 0.352796 | low |
| TCGA-BP-4807 | 0.5918 | 0.1926 | 0.1202 | 0.3915 | 1 | 0 | 0.399114 | low |
| TCGA-A3-3352 | 0.6594 | 0.1403 | 0.1792 | 0.3035 | 0.8643 | 0.059 | 0.463399 | low |
| TCGA-B0-5712 | 0.5731 | 0.0913 | 0.1151 | 0.4911 | 0.7797 | 0.0375 | 0.639481 | low |
| TCGA-B0-4843 | 0.2494 | 0.3953 | 0.2732 | 0.8283 | 0.74646 | 0.2201 | 7.249156 | high |
| TCGA-B8-A54H | 0.5132 | 0.2431 | 0.1244 | 0.3434 | 0.6995 | 0.0767 | 1.0376 | high |
| TCGA-CZ-5456 | 0.4615 | 0.1589 | 0.1396 | 0.5335 | 0.8094 | 0.0217 | 0.91392 | high |
| TCGA-B0-4828 | 0.6494 | 0.2505 | 0.352 | 0.5627 | 0.7124 | 0.0781 | 1.730788 | high |
| TCGA-AK-3444 | 0.5839 | 0.1834 | 0.0686 | 0.3565 | 0.855 | 0.0103 | 0.470728 | low |
| TCGA-CJ-6031 | 0.3453 | 0.2143 | 0.1476 | 0.6542 | 0.8094 | 0.0351 | 1.5616 | high |
| TCGA-B8-A54K | 0.7996 | 0.2125 | 0.0934 | 0.2554 | 0.88757 | 0.0721 | 0.330071 | low |
| TCGA-DV-5566 | 0.4746 | 0.2009 | 0.0362 | 0.5046 | 0.8947 | 0 | 0.619503 | low |
| TCGA-B0-5113 | 0.3584 | 0.187 | 0.4147 | 0.3514 | 0.8906 | 0.0461 | 1.340659 | high |
| TCGA-BP-4169 | 0.2333 | 0.361 | 0.2037 | 0.7023 | 1 | 0.1014 | 2.378425 | high |
| TCGA-CZ-5986 | 0.5986 | 0.0627 | 0.1435 | 0.3398 | 0.8415 | 0.03807 | 0.429785 | low |
| TCGA-CJ-4640 | 0.5413 | 0.3009 | 0.0477 | 0.3297 | 0.8004 | 0.0212 | 0.69372 | low |
| TCGA-B4-5836 | 0.459 | 0.1777 | 0.0504 | 0.4412 | 0.8094 | 0.0288 | 0.715182 | low |
| TCGA-B0-5713 | 0.5529 | 0.1624 | 0.1396 | 0.6218 | 0.9233 | 0.0179 | 0.700118 | low |
| TCGA-BP-4790 | 0.3854 | 0.2202 | 0.5055 | 0.5072 | 1 | 0 | 1.498049 | high |
| TCGA-BP-4174 | 0.4291 | 0.3838 | 0.1098 | 0.2541 | 0.934 | 0 | 0.742532 | low |
| TCGA-BP-4161 | 0.4716 | 0.2783 | 0.5188 | 0.4288 | 1 | 0.1185 | 1.682728 | high |
| TCGA-BP-5183 | 0.3832 | 0.2366 | 0.3611 | 0.2047 | 1 | 0.0449 | 0.831835 | low |
| TCGA-CW-5588 | 0.3941 | 0.1383 | 0.0862 | 0.3358 | 1 | 0 | 0.429711 | low |
| TCGA-BP-4970 | 0.4055 | 0.2473 | 0.063 | 0.3139 | 0.8947 | 0.0304 | 0.654868 | low |
| TCGA-A3-3319 | 0.545 | 0.4348 | 0.4742 | 0.2785 | 0.7124 | 0.057 | 2.511904 | high |
| TCGA-BP-4349 | 0.4831 | 0.3643 | 0.3424 | 0.3984 | 0.7797 | 0.1583 | 2.318783 | high |
| TCGA-A3-3335 | 0.6771 | 0.1399 | 0.4154 | 0.5914 | 0.7023 | 0.1522 | 1.787556 | high |
| TCGA-CZ-5464 | 0.4186 | 0.155 | 0.2235 | 0.5046 | 0.8705 | 0.0515 | 1.02399 | high |
| TCGA-AK-3436 | 0.4292 | 0.1067 | 0.2788 | 0.6218 | 0.7936 | 0 | 1.267546 | high |
| TCGA-AK-3427 | 0.8504 | 0.2914 | 0.2312 | 0.0604 | 0.8814 | 0.01872 | 0.324844 | low |
| TCGA-BP-4163 | 0.5133 | 0.2557 | 0.2908 | 0.2374 | 1 | 0.086 | 0.68657 | low |
| TCGA-BP-4170 | 0.5075 | 0.151 | 0.0699 | 0.5296 | 0.8499 | 0.0221 | 0.663872 | low |
| TCGA-B0-5088 | 0.557 | 0.2198 | 0.2604 | 0.6336 | 1 | 0.1389 | 1.083869 | high |
| TCGA-BP-4329 | 0.4991 | 0.2057 | 0.0447 | 0.4862 | 0.761 | 0.1014 | 0.950253 | high |
| TCGA-CW-5591 | 0.5599 | 0.2949 | 0.0399 | 0.2507 | 0.8575 | 0 | 0.497451 | low |
| TCGA-CW-5580 | 0.4923 | 0.2089 | 0.1705 | 0.3375 | 0.8384 | 0 | 0.700368 | low |
| TCGA-BP-4177 | 0.8025 | 0.1093 | 0.0397 | 0.229 | 1 | 0 | 0.156881 | low |
| TCGA-B0-4837 | 0.359 | 0.3046 | 0.1582 | 0.6069 | 0.856357 | 0.0686 | 1.714423 | high |
| TCGA-CJ-4907 | 0.4209 | 0.1464 | 0.184 | 0.3867 | 0.8384 | 0.0363 | 0.813435 | low |
| TCGA-CJ-4891 | 0.1981 | 0.5984 | 0.3898 | 0.4911 | 0.4694 | 0.1583 | 15.13729 | high |
| TCGA-A3-3365 | 0.4655 | 0.198 | 0.1635 | 0.4237 | 0.8415 | 0.08051 | 0.924374 | high |
| TCGA-BP-4982 | 0.4321 | 0.1501 | 0.2226 | 0.3241 | 0.9083 | 0.1172 | 0.797275 | low |
| TCGA-B8-5549 | 0.5223 | 0.1367 | 0.0388 | 0.2263 | 0.761 | 0.0173 | 0.455477 | low |
| TCGA-MM-A563 | 0.4836 | 0.2683 | 0.111 | 0.5587 | 0.821625 | 0.0441 | 1.11777 | high |
| TCGA-B0-4688 | 0.0054 | 0.4734 | 0.2336 | 0.6999 | 0.6691 | 0.2034 | 11.48041 | high |
| TCGA-BP-5194 | 0.556 | 0.1044 | 0.09 | 0.4112 | 1 | 0.0281 | 0.357524 | low |
| TCGA-CJ-4634 | 0.6287 | 0.2629 | 0.3248 | 0.5174 | 0.8762 | 0.1414 | 1.312588 | high |
| TCGA-CZ-5462 | 0.3321 | 0.1966 | 0.1977 | 0.1416 | 0.9139 | 0.0623 | 0.685406 | low |
| TCGA-CJ-4902 | 0.4817 | 0.3017 | 0.3187 | 0.6833 | 0.8643 | 0.211 | 2.697663 | high |
| TCGA-BP-4981 | 0.5875 | 0.1812 | 0.3926 | 0.6587 | 1 | 0.1114 | 1.1993 | high |
| TCGA-A3-A6NI | 0.5803 | 0.1499 | 0.1445 | 0.2158 | 0.9008 | 0.0717 | 0.424324 | low |
| TCGA-BP-5195 | 0.4159 | 0.1786 | 0.0869 | 0.476 | 0.902 | 0.0262 | 0.714065 | low |
| TCGA-BP-4782 | 0.4677 | 0.3049 | 0.1547 | 0.4313 | 0.761 | 0 | 1.17763 | high |
| TCGA-A3-3311 | 0.4779 | 0.3343 | 0.2603 | 0.3335 | 0.8384 | 0.1014 | 1.354086 | high |
| TCGA-CZ-4864 | 0.5642 | 0.1844 | 0.2334 | 0.3752 | 0.832 | 0.0555 | 0.786233 | low |
| TCGA-B2-4102 | 0.4549 | 0.2347 | 0.4302 | 0.079 | 0.9421 | 0.1137 | 0.896746 | high |
| TCGA-CJ-4878 | 0.6921 | 0.405 | 0.3338 | 0.5412 | 1 | 0.1373 | 1.300441 | high |
| TCGA-AK-3428 | 0.644 | 0.0453 | 0.05 | 0.2949 | 0.8489 | 0 | 0.274643 | low |
| TCGA-BP-4971 | 0.4619 | 0.4592 | 0.3388 | 0.7249 | 0.800125 | 0.154 | 4.47564 | high |
| TCGA-CJ-5676 | 0.2666 | 0.083 | 0.0518 | 0.5775 | 0.820583 | 0 | 0.911271 | high |
| TCGA-B8-A54J | 0.4705 | 0.2312 | 0.1105 | 0.3299 | 0.84855 | 0.094 | 0.786548 | low |
| TCGA-BP-4999 | 0.4594 | 0.0881 | 0.1705 | 0.4599 | 1 | 0.113 | 0.598069 | low |
| TCGA-BP-4332 | 0.6765 | 0.2672 | 0.3578 | 0.4694 | 0.8762 | 0 | 0.930312 | high |
| TCGA-CZ-4863 | 0.54 | 0.2222 | 0.2554 | 0.2739 | 0.8705 | 0.1404 | 0.869997 | low |
| TCGA-A3-3374 | 0.8174 | 0.5945 | 0.3182 | 0.4144 | 0.6294 | 0 | 2.152957 | high |
| TCGA-A3-3326 | 0.5055 | 0.0899 | 0.2475 | 0.6318 | 0.8195 | 0.1014 | 1.173786 | high |
| TCGA-T7-A92I | 0.3467 | 0.378 | 0.0713 | 0.0418 | 0.7774 | 0.0515 | 0.882195 | high |
| TCGA-AK-3425 | 0.6363 | 0.1186 | 0.11 | 0.3056 | 1 | 0.0321 | 0.289153 | low |
| TCGA-CJ-4894 | 0.462 | 0.1223 | 0.2159 | 0.1909 | 0.8004 | 0.0877 | 0.687703 | low |
| TCGA-AK-3458 | 0.565 | 0.0881 | 0.3583 | 0.8373 | 0.8862 | 0 | 1.262421 | high |
| TCGA-B0-5106 | 0.465 | 0.1714 | 0.2334 | 0.6291 | 0.8575 | 0.1326 | 1.4306 | high |
| TCGA-A3-3382 | 0.421 | 0.1651 | 0.1963 | 0.3602 | 0.832 | 0.1014 | 0.956997 | high |
| TCGA-BP-4353 | 0.4225 | 0.1835 | 0.3448 | 0.3525 | 0.832 | 0.0341 | 1.152598 | high |
| TCGA-B0-5698 | 0.5528 | 0.2412 | 0.0832 | 0.4237 | 0.882517 | 0 | 0.591794 | low |
| TCGA-CZ-4866 | 0.7922 | 0.2784 | 0.2608 | 0.1076 | 0.7956 | 0.0371 | 0.489128 | low |
| TCGA-BP-4799 | 0.1021 | 0.2171 | 0.21249 | 0.5335 | 0.733667 | 0.05676 | 2.770207 | high |
| TCGA-CW-5583 | 0.6418 | 0.0987 | 0.0964 | 0.1997 | 0.8575 | 0.0154 | 0.299027 | low |
| TCGA-B0-5085 | 0.5942 | 0.2225 | 0.3043 | 0.6389 | 0.573059 | 0.3608 | 4.144167 | high |
| TCGA-CJ-5683 | 0.5502 | 0.1721 | 0.1179 | 0.55 | 0.7541 | 0.0561 | 0.950133 | high |
| TCGA-CW-5590 | 0.4687 | 0.1664 | 0.1582 | 0.4862 | 0.9139 | 0.0534 | 0.758812 | low |
| TCGA-CZ-5457 | 0.532 | 0.0679 | 0.2014 | 0.2481 | 0.7797 | 0 | 0.50553 | low |
| TCGA-B8-A7U6 | 0.4625 | 0.1617 | 0.1069 | 0.6465 | 0.858167 | 0.0878 | 1.037565 | high |
| TCGA-BP-5185 | 0.6418 | 0.1382 | 0.2962 | 0.61711 | 0.564725 | 0.3156 | 2.827567 | high |
| TCGA-BP-4763 | 0.3809 | 0.2415 | 0.2527 | 0.4911 | 0.832 | 0 | 1.348796 | high |
| TCGA-BP-5180 | 0.529 | 0.1196 | 0.1074 | 0.0968 | 1 | 0.0388 | 0.259988 | low |
| TCGA-B2-5635 | 0.4481 | 0.185 | 0.1638 | 0.3067 | 1 | 0.0159 | 0.49815 | low |
| TCGA-BP-4789 | 0.4733 | 0.079 | 0.2404 | 0.4599 | 0.8094 | 0.051 | 0.866548 | low |
| TCGA-A3-A8OV | 0.6117 | 0.126 | 0.1103 | 0.5134 | 0.870771 | 0.0345 | 0.543753 | low |
| TCGA-CJ-4643 | 0.5759 | 0.3578 | 0.1911 | 0.3535 | 0.9503 | 0.0971 | 0.85326 | low |
| TCGA-BP-4326 | 0.3105 | 0.3981 | 0.2288 | 0.6654 | 0.8499 | 0.2322 | 3.935129 | high |
| TCGA-BP-4960 | 0.4035 | 0.282 | 0.2294 | 0.7433 | 0.820057 | 0.1678 | 2.741056 | high |
| TCGA-B0-5121 | 0.6248 | 0.174 | 0.546 | 0.4252 | 0.6798 | 0 | 1.679423 | high |
| TCGA-B2-4099 | 0.4543 | 0.083 | 0.3614 | 0.407 | 0.697 | 0 | 1.212904 | high |
| TCGA-BP-5168 | 0.6725 | 0.1075 | 0.1339 | 0.6002 | 0.761 | 0 | 0.656719 | low |
| TCGA-DV-A4W0 | 0.4992 | 0.2705 | 0.1114 | 0.322 | 0.871963 | 0.0524 | 0.712239 | low |
| TCGA-CJ-4905 | 0.4886 | 0.1303 | 0.076 | 0.3997 | 1 | 0.0225 | 0.402604 | low |
| TCGA-CJ-5675 | 0.5054 | 0.1096 | 0.0059 | 0.0604 | 1 | 0 | 0.192171 | low |
| TCGA-AK-3443 | 0.9209 | 0.4035 | 0.1273 | 0.065 | 0.8984 | 0.0468 | 0.309162 | low |
| TCGA-MW-A4EC | 0.6235 | 0.1347 | 0.1032 | 0.3339 | 0.7586 | 0.0878 | 0.578051 | low |
| TCGA-A3-3325 | 0.596 | 0.2037 | 0.3374 | 0.3915 | 0.9421 | 0.1114 | 0.858125 | low |
| TCGA-CZ-5469 | 0.4505 | 0.1405 | 0.3321 | 0.7605 | 0.827133 | 0.0427 | 1.79972 | high |
| TCGA-AK-3453 | 0.8997 | 0.3203 | 0.1428 | 0.2688 | 0.7797 | 0.0205 | 0.449095 | low |
| TCGA-CZ-5452 | 0.4616 | 0.1816 | 0.1376 | 0.3149 | 0.8499 | 0.0474 | 0.674205 | low |
| TCGA-CJ-4901 | 0.3912 | 0.4249 | 0.4751 | 0.5296 | 0.6026 | 0.1062 | 6.341491 | high |
| TCGA-B0-4700 | 0.1077 | 0.2324 | 0.2312 | 0.4409 | 0.7541 | 0.0781 | 2.569077 | high |
| TCGA-B2-3923 | 0.854 | 0.4858 | 0.2257 | 0.25245 | 0.8214 | 0 | 0.707191 | low |
| TCGA-BP-5184 | 0.4731 | 0.1672 | 0.0811 | 0.4248 | 0.6506 | 0.0133 | 0.955491 | high |
| TCGA-B0-4810 | 0.4607 | 0.2838 | 0.2564 | 0.4473 | 0.6798 | 0.2201 | 2.537357 | high |
| TCGA-BP-5176 | 0.5204 | 0.2422 | 0.1113 | 0.4269 | 0.8499 | 0 | 0.711621 | low |
| TCGA-CZ-5463 | 0.6461 | 0.0672 | 0.1116 | 0.1437 | 1 | 0.0116 | 0.194116 | low |
| TCGA-B2-5633 | 0.3936 | 0.2431 | 0.0966 | 0.2224 | 0.531 | 0.0488 | 1.367569 | high |
| TCGA-B8-A54E | 0.2996 | 0.5599 | 0.086 | 0.0501 | 0.7884 | 0.0356 | 1.410754 | high |
| TCGA-B0-4844 | 0.5436 | 0.2925 | 0.2898 | 0.5412 | 0.905 | 0.0659 | 1.281525 | high |
| TCGA-BP-5169 | 0.3992 | 0.398 | 0.2576 | 0.6457 | 1 | 0.192 | 2.354554 | high |
| TCGA-B0-4714 | 0.4644 | 0.3504 | 0.2871 | 0.5914 | 0.847538 | 0.0561 | 1.973608 | high |
| TCGA-CZ-4853 | 0.7704 | 0.1254 | 0.1545 | 0.1338 | 1 | 0.0348 | 0.199456 | low |

Table S5

| ID | ESAM\|19236\|AD | STRA13\|44264\|AD | PIGG\|68359\|AD | PRMT2\|60963\|AD | UQCR10\|61665\|AD | Risk score | risk |
| --- | --- | --- | --- | --- | --- | --- | --- |
| TCGA-CJ-4637 | 0.1413 | 0.2351 | 0.871 | 0.2095 | 0.1374 | 1.333168 | high |
| TCGA-CZ-4861 | 0.2315 | 0.0569 | 0.885 | 0.2778 | 0.1404 | 2.270819 | high |
| TCGA-BP-4964 | 0.1508 | 0.1054 | 0.9704 | 0.3371 | 0.1367 | 1.002263 | high |
| TCGA-CJ-4887 | 0.1819 | 0.1322 | 0.9811 | 0.2921 | 0.1039 | 0.783671 | low |
| TCGA-BP-5198 | 0.234 | 0.0402 | 0.8466 | 0.0666 | 0.1205 | 1.417765 | high |
| TCGA-BP-4967 | 0.1696 | 0.0463 | 0.9547 | 0.2322 | 0.1275 | 0.837672 | low |
| TCGA-A3-3351 | 0.1549 | 0.1142 | 0.9014 | 0.4518 | 0.124 | 1.151096 | high |
| TCGA-B0-4710 | 0.1651 | 0.1162 | 0.8941 | 0.2969 | 0.1394 | 1.384805 | high |
| TCGA-B0-5083 | 0.1931 | 0.0597 | 0.9793 | 0.0166 | 0.1124 | 0.629499 | low |
| TCGA-BP-4803 | 0.1877 | 0.0865 | 0.9508 | 0.3808 | 0.1453 | 1.691789 | high |
| TCGA-CJ-4889 | 0.1665 | 0.0812 | 0.9389 | 0.2147 | 0.1145 | 0.727515 | low |
| TCGA-B0-5080 | 0.1724 | 0.1031 | 0.9067 | 0.1159 | 0.1844 | 2.321187 | high |
| TCGA-CW-5587 | 0.1435 | 0.1587 | 0.956 | 0.0839 | 0.0717 | 0.296643 | low |
| TCGA-A3-3376 | 0.1803 | 0.0815 | 0.9188 | 0.2807 | 0.1328 | 1.237679 | high |
| TCGA-CJ-4897 | 0.1701 | 0.0551 | 0.9912 | 0.063 | 0.0737 | 0.282195 | low |
| TCGA-CW-6088 | 0.1646 | 0.0794 | 0.9778 | 0.1531 | 0.0991 | 0.479078 | low |
| TCGA-B8-5552 | 0.1332 | 0.0997 | 0.9517 | 0.2372 | 0.1337 | 0.751467 | low |
| TCGA-CW-5589 | 0.1226 | 0.0683 | 0.9347 | 0.0695 | 0.1079 | 0.367619 | low |
| TCGA-B0-4833 | 0.2038 | 0.171 | 0.795 | 0.4969 | 0.1201 | 2.551957 | high |
| TCGA-CZ-4859 | 0.1255 | 0.0493 | 0.9851 | 0.0415 | 0.0799 | 0.202323 | low |
| TCGA-A3-3320 | 0.2026 | 0.0791 | 0.8777 | 0.197 | 0.083 | 0.691732 | low |
| TCGA-MM-A564 | 0.1754 | 0.1637 | 0.9359 | 0.3552 | 0.1555 | 2.104788 | high |
| TCGA-AS-3777 | 0.2548 | 0.0568 | 0.956 | 0.0087 | 0.0817 | 0.709727 | low |
| TCGA-B0-5095 | 0.2346 | 0.1484 | 0.8213 | 0.4664 | 0.1712 | 6.541496 | high |
| TCGA-BP-4795 | 0.1668 | 0.0486 | 0.9497 | 0.062 | 0.142 | 0.863698 | low |
| TCGA-BP-4331 | 0.214 | 0.1381 | 0.877 | 0.2818 | 0.1225 | 1.779331 | high |
| TCGA-A3-A6NJ | 0.1654 | 0.103 | 0.9023 | 0.3928 | 0.1107 | 0.939137 | high |
| TCGA-B0-4701 | 0.1769 | 0.1374 | 0.8719 | 0.4314 | 0.1186 | 1.420026 | high |
| TCGA-A3-3367 | 0.1597 | 0.1275 | 0.9332 | 0.3131 | 0.1136 | 0.840337 | low |
| TCGA-B0-5402 | 0.1543 | 0.079 | 0.944 | 0.0364 | 0.1278 | 0.648251 | low |
| TCGA-B0-5691 | 0.1692 | 0.0791 | 0.9345 | 0.1574 | 0.0818 | 0.4216 | low |
| TCGA-BP-4342 | 0.2303 | 0.184 | 0.8395 | 0.3685 | 0.1229 | 2.744356 | high |
| TCGA-A3-3373 | 0.1392 | 0.1139 | 0.9795 | 0.1943 | 0.0895 | 0.366805 | low |
| TCGA-BP-4341 | 0.2161 | 0.1795 | 0.9517 | 0.4434 | 0.1065 | 1.558112 | high |
| TCGA-CW-6090 | 0.1556 | 0.2189 | 1 | 0.055 | 0.1481 | 1.095756 | high |
| TCGA-A3-3307 | 0.12 | 0.1347 | 0.9257 | 0.254 | 0.1007 | 0.462253 | low |
| TCGA-B2-3924 | 0.2625 | 0.6941 | 0.8209 | 0.569 | 0.0751 | 6.761803 | high |
| TCGA-B0-5706 | 0.1323 | 0.1521 | 1 | 0.2078 | 0.156 | 1.027565 | high |
| TCGA-BP-4963 | 0.2376 | 0.0736 | 0.8678 | 0.1758 | 0.1346 | 2.111063 | high |
| TCGA-BP-4343 | 0.1567 | 0.1255 | 0.9636 | 0.082 | 0.0732 | 0.313018 | low |
| TCGA-B0-5703 | 0.1499 | 0.0302 | 0.9544 | 0.2578 | 0.088 | 0.374561 | low |
| TCGA-CJ-6027 | 0.1692 | 0.1381 | 0.9268 | 0.1589 | 0.1244 | 0.95084 | high |
| TCGA-B0-5108 | 0.1893 | 0.1063 | 0.9383 | 0.137 | 0.1562 | 1.671238 | high |
| TCGA-BP-4965 | 0.1443 | 0.0893 | 0.9717 | 0.1347 | 0.1165 | 0.5305 | low |
| TCGA-A3-A6NL | 0.1582 | 0.1239 | 0.8599 | 0.3197 | 0.1213 | 1.102099 | high |
| TCGA-CJ-5672 | 0.1798 | 0.0885 | 0.9319 | 0.2105 | 0.0702 | 0.421371 | low |
| TCGA-B8-5158 | 0.1678 | 0.1016 | 0.9041 | 0.0731 | 0.1613 | 1.48213 | high |
| TCGA-CJ-4876 | 0.2029 | 0.1209 | 0.9297 | 0.1702 | 0.0587 | 0.447837 | low |
| TCGA-B0-5115 | 0.166 | 0.1478 | 0.9092 | 0.4465 | 0.134 | 1.564593 | high |
| TCGA-CJ-4916 | 0.1845 | 0.1284 | 0.8735 | 0.2042 | 0.0966 | 0.820673 | low |
| TCGA-B0-4848 | 0.163 | 0.1166 | 0.9804 | 0.0665 | 0.1316 | 0.769214 | low |
| TCGA-B4-5377 | 0.1516 | 0.0809 | 0.9521 | 0.052 | 0.0867 | 0.333088 | low |
| TCGA-B0-4712 | 0.1442 | 0.1957 | 1 | 0.1227 | 0.1271 | 0.728199 | low |
| TCGA-A3-3324 | 0.1863 | 0.1288 | 0.8884 | 0.2511 | 0.152 | 2.026309 | high |
| TCGA-CZ-5465 | 0.1781 | 0.0597 | 0.9804 | 0.0628 | 0.1359 | 0.832635 | low |
| TCGA-CZ-4860 | 0.2494 | 0.2495 | 0.7408 | 0.1572 | 0.2225 | 17.72596 | high |
| TCGA-B0-5102 | 0.1984 | 0.1159 | 0.8811 | 0.1132 | 0.1391 | 1.57075 | high |
| TCGA-B0-5690 | 0.1635 | 0.0781 | 0.9333 | 0.2851 | 0.1114 | 0.732907 | low |
| TCGA-BP-4160 | 0.1631 | 0.0572 | 0.9761 | 0.0844 | 0.1154 | 0.541454 | low |
| TCGA-B0-4697 | 0.1977 | 0.171 | 0.8421 | 0.6812 | 0.1182 | 2.581417 | high |
| TCGA-AK-3454 | 0.1776 | 0.0795 | 0.9514 | 0.1528 | 0.1122 | 0.702869 | low |
| TCGA-B0-5693 | 0.1692 | 0.0557 | 0.9321 | 0.1991 | 0.0816 | 0.42073 | low |
| TCGA-BP-4985 | 0.2115 | 0.1209 | 0.914 | 0.0872 | 0.1185 | 1.169487 | high |
| TCGA-B0-4718 | 0.1766 | 0.3052 | 0.7393 | 0.44 | 0.0929 | 1.844829 | high |
| TCGA-B0-5109 | 0.2509 | 0.3159 | 0.8295 | 0.1009 | 0.1286 | 3.662425 | high |
| TCGA-A3-3359 | 0.163 | 0.1684 | 0.9063 | 0.3899 | 0.0746 | 0.593062 | low |
| TCGA-DV-5569 | 0.1438 | 0.1253 | 0.896 | 0.3028 | 0.1046 | 0.675158 | low |
| TCGA-B2-A4SR | 0.1699 | 0.1259 | 0.8841 | 0.3805 | 0.1425 | 1.738929 | high |
| TCGA-B0-4815 | 0.1625 | 0.2107 | 0.8717 | 0.3195 | 0.1703 | 2.898205 | high |
| TCGA-AK-3461 | 0.1778 | 0.1672 | 0.8823 | 0.2633 | 0.1118 | 1.113959 | high |
| TCGA-CJ-4892 | 0.149 | 0.1111 | 0.9446 | 0.2489 | 0.1019 | 0.556243 | low |
| TCGA-B0-5695 | 0.1618 | 0.0978 | 0.948 | 0.1268 | 0.0901 | 0.438199 | low |
| TCGA-BP-4975 | 0.1436 | 0.0751 | 0.9633 | 0.1242 | 0.1069 | 0.443098 | low |
| TCGA-AK-3456 | 0.2129 | 0.1865 | 0.9531 | 0.1311 | 0.1167 | 1.274262 | high |
| TCGA-BP-4159 | 0.162 | 0.0433 | 0.9558 | 0.3035 | 0.1075 | 0.612302 | low |
| TCGA-CZ-5458 | 0.1545 | 0.0563 | 0.9807 | 0.1175 | 0.1206 | 0.556322 | low |
| TCGA-AK-3431 | 0.205 | 0.1347 | 1 | 0.2762 | 0.159 | 2.173678 | high |
| TCGA-B8-A8YJ | 0.2082 | 0.1369 | 0.9373 | 0.2402 | 0.1446 | 1.98551 | high |
| TCGA-BP-5182 | 0.1572 | 0.0718 | 0.9342 | 0.1219 | 0.1162 | 0.613749 | low |
| TCGA-B2-4101 | 0.2037 | 0.1209 | 0.8322 | 0.3846 | 0.1444 | 2.72439 | high |
| TCGA-CJ-5681 | 0.1825 | 0.1196 | 0.9593 | 0.0783 | 0.1255 | 0.893136 | low |
| TCGA-BP-4994 | 0.2247 | 0.0915 | 0.9318 | 0.0972 | 0.1018 | 0.926212 | high |
| TCGA-CJ-4903 | 0.1343 | 0.1028 | 0.9092 | 0.2193 | 0.0735 | 0.32081 | low |
| TCGA-BP-4760 | 0.172 | 0.0187 | 0.9871 | 0.0569 | 0.1358 | 0.70471 | low |
| TCGA-BP-5190 | 0.1516 | 0.1193 | 0.982 | 0.1191 | 0.1032 | 0.471589 | low |
| TCGA-B0-5710 | 0.1784 | 0.1119 | 0.9068 | 0.2442 | 0.1186 | 1.025627 | high |
| TCGA-CJ-4875 | 0.182 | 0.148 | 0.8753 | 0.6328 | 0.131 | 2.28955 | high |
| TCGA-CJ-4874 | 0.1389 | 0.0453 | 0.9733 | 0.1172 | 0.1178 | 0.458338 | low |
| TCGA-B2-5641 | 0.1206 | 0.0657 | 0.9908 | 0.1224 | 0.1169 | 0.386245 | low |
| TCGA-CW-5584 | 0.1441 | 0.0637 | 0.9611 | 0.0882 | 0.1315 | 0.616982 | low |
| TCGA-B8-4143 | 0.213 | 0.304 | 0.8408 | 0.8177 | 0.1811 | 12.33653 | high |
| TCGA-B0-5120 | 0.1717 | 0.1084 | 0.9683 | 0.2455 | 0.1343 | 1.068197 | high |
| TCGA-EU-5905 | 0.1486 | 0.067 | 0.9083 | 0.1567 | 0.1409 | 0.910823 | high |
| TCGA-BP-4338 | 0.1348 | 0.0725 | 0.9874 | 0.2298 | 0.1406 | 0.733715 | low |
| TCGA-A3-3358 | 0.1838 | 0.0957 | 0.9841 | 0.2572 | 0.1122 | 0.802719 | low |
| TCGA-B0-4818 | 0.2257 | 0.0723 | 0.8484 | 0.3453 | 0.1187 | 1.852456 | high |
| TCGA-BP-4976 | 0.1182 | 0.0274 | 0.9914 | 0.0766 | 0.0875 | 0.208467 | low |
| TCGA-BP-4345 | 0.2155 | 0.1091 | 0.9302 | 0.1658 | 0.1253 | 1.38332 | high |
| TCGA-CW-6093 | 0.1993 | 0.0985 | 0.9647 | 0.1283 | 0.1175 | 0.916406 | high |
| TCGA-BP-4330 | 0.2208 | 0.084 | 0.9466 | 0.3021 | 0.112 | 1.250721 | high |
| TCGA-EU-5906 | 0.1446 | 0.0892 | 0.9614 | 0.198 | 0.1053 | 0.489591 | low |
| TCGA-B0-4846 | 0.1864 | 0.0898 | 0.8721 | 0.4546 | 0.1054 | 1.165931 | high |
| TCGA-CJ-6030 | 0.223 | 0.1667 | 0.96 | 0.2401 | 0.1195 | 1.553483 | high |
| TCGA-BP-4983 | 0.1409 | 0.1193 | 0.9613 | 0.0997 | 0.1675 | 1.201587 | high |
| TCGA-B0-5697 | 0.138 | 0.084 | 1 | 0.1367 | 0.137 | 0.642176 | low |
| TCGA-BP-4346 | 0.2531 | 0.1209 | 0.9836 | 0.1957 | 0.1273 | 1.892068 | high |
| TCGA-B0-4691 | 0.1869 | 0.1011 | 0.837 | 0.3335 | 0.1312 | 1.702913 | high |
| TCGA-A3-3328 | 0.216 | 0.0696 | 0.9656 | 0.1514 | 0.1253 | 1.160619 | high |
| TCGA-BP-4765 | 0.1914 | 0.1297 | 0.8967 | 0.2491 | 0.1139 | 1.146462 | high |
| TCGA-CZ-4858 | 0.2033 | 0.2813 | 0.974 | 0.0575 | 0.1749 | 3.134307 | high |
| TCGA-B0-5117 | 0.2712 | 0.1653 | 0.8838 | 0.2012 | 0.1433 | 3.972222 | high |
| TCGA-CZ-4865 | 0.1562 | 0.1226 | 0.9889 | 0.0978 | 0.1227 | 0.646743 | low |
| TCGA-CJ-6028 | 0.21 | 0.3402 | 0.9438 | 0.1065 | 0.1293 | 2.089567 | high |
| TCGA-6D-AA2E | 0.1869 | 0.0796 | 0.9022 | 0.184 | 0.134 | 1.245032 | high |
| TCGA-CJ-4884 | 0.1982 | 0.0797 | 0.8471 | 0.2699 | 0.1325 | 1.677642 | high |
| TCGA-DV-5568 | 0.1659 | 0.0819 | 0.9696 | 0.0958 | 0.1061 | 0.520193 | low |
| TCGA-BP-4961 | 0.1555 | 0.0836 | 0.9332 | 0.1923 | 0.1462 | 1.0742 | high |
| TCGA-BP-4761 | 0.156 | 0.0433 | 0.8889 | 0.1554 | 0.1149 | 0.642718 | low |
| TCGA-CJ-5677 | 0.194 | 0.1854 | 0.8815 | 0.0954 | 0.1707 | 2.815979 | high |
| TCGA-CZ-5460 | 0.1427 | 0.0957 | 0.9599 | 0.1272 | 0.1141 | 0.519862 | low |
| TCGA-CJ-4900 | 0.1769 | 0.1801 | 0.928 | 0.2705 | 0.1598 | 2.192531 | high |
| TCGA-CZ-5451 | 0.111 | 0.0728 | 0.9774 | 0.121 | 0.0753 | 0.19257 | low |
| TCGA-B8-A54F | 0.1919 | 0.0843 | 0.8046 | 0.4441 | 0.1716 | 3.934271 | high |
| TCGA-B0-4838 | 0.1825 | 0.0918 | 0.9329 | 0.3733 | 0.1176 | 1.091328 | high |
| TCGA-B0-4699 | 0.2459 | 0.2099 | 0.9035 | 0.1622 | 0.1278 | 2.493577 | high |
| TCGA-BP-4781 | 0.1346 | 0.0563 | 1 | 0.1342 | 0.1333 | 0.552038 | low |
| TCGA-CJ-4644 | 0.1685 | 0.0627 | 1 | 0.0507 | 0.0987 | 0.404585 | low |
| TCGA-B0-5701 | 0.1397 | 0.0762 | 0.9851 | 0.4787 | 0.11 | 0.632833 | low |
| TCGA-B0-5694 | 0.1598 | 0.0802 | 0.9709 | 0.1475 | 0.119 | 0.63333 | low |
| TCGA-B8-4621 | 0.1903 | 0.082 | 0.9185 | 0.4667 | 0.1723 | 3.096222 | high |
| TCGA-BP-5006 | 0.1348 | 0.0496 | 0.9499 | 0.1455 | 0.1025 | 0.381167 | low |
| TCGA-BP-5181 | 0.154 | 0.0511 | 0.9873 | 0.0499 | 0.0958 | 0.339568 | low |
| TCGA-B0-5709 | 0.1699 | 0.0694 | 0.9776 | 0.1242 | 0.1239 | 0.703613 | low |
| TCGA-BP-4167 | 0.1648 | 0.1119 | 0.8889 | 0.1383 | 0.1269 | 0.955829 | high |
| TCGA-CJ-4638 | 0.1593 | 0.1626 | 0.8 | 0.7974 | 0.1301 | 2.68693 | high |
| TCGA-EU-5907 | 0.2159 | 0.0898 | 0.9213 | 0.0303 | 0.1114 | 0.941526 | high |
| TCGA-CJ-4869 | 0.1545 | 0.0864 | 0.9235 | 0.1212 | 0.1139 | 0.609994 | low |
| TCGA-3Z-A93Z | 0.1619 | 0.0941 | 0.8527 | 0.1084 | 0.115 | 0.780368 | low |
| TCGA-BP-5196 | 0.1935 | 0.049 | 0.9627 | 0.2782 | 0.1581 | 1.749531 | high |
| TCGA-AK-3433 | 0.2258 | 0.1209 | 0.9442 | 0.097 | 0.1228 | 1.346598 | high |
| TCGA-A3-3346 | 0.2238 | 0.0913 | 0.9728 | 0.1707 | 0.1682 | 2.571033 | high |
| TCGA-AK-3440 | 0.1845 | 0.1122 | 0.9424 | 0.08 | 0.097 | 0.59599 | low |
| TCGA-B8-A54D | 0.1891 | 0.4011 | 0.9414 | 0.5029 | 0.1115 | 2.314485 | high |
| TCGA-BP-5200 | 0.1545 | 0.0812 | 0.9721 | 0.0717 | 0.0889 | 0.346121 | low |
| TCGA-B0-5400 | 0.1709 | 0.0745 | 0.9667 | 0.169 | 0.1286 | 0.831937 | low |
| TCGA-BP-4969 | 0.1957 | 0.1171 | 0.932 | 0.3646 | 0.0582 | 0.50846 | low |
| TCGA-BP-4756 | 0.2137 | 0.1523 | 0.895 | 0.1657 | 0.0627 | 0.605569 | low |
| TCGA-BP-4776 | 0.1649 | 0.1282 | 0.7683 | 0.3034 | 0.0922 | 0.904223 | low |
| TCGA-CJ-4912 | 0.1936 | 0.2533 | 0.7746 | 0.4129 | 0.133 | 3.240853 | high |
| TCGA-CZ-5985 | 0.1656 | 0.1352 | 0.9538 | 0.1648 | 0.1165 | 0.765282 | low |
| TCGA-B0-5098 | 0.2294 | 0.278 | 0.8846 | 0.2127 | 0.1985 | 8.29216 | high |
| TCGA-B8-5545 | 0.1244 | 0.1486 | 0.9707 | 0.1161 | 0.1523 | 0.864514 | low |
| TCGA-BP-4758 | 0.1268 | 0.1028 | 1 | 0.1648 | 0.0897 | 0.296588 | low |
| TCGA-B0-4696 | 0.2467 | 0.16 | 0.7535 | 0.0916 | 0.1908 | 7.851717 | high |
| TCGA-A3-3322 | 0.1728 | 0.088 | 0.8407 | 0.4774 | 0.1196 | 1.410005 | high |
| TCGA-B0-5107 | 0.2919 | 0.1416 | 0.9147 | 0.6171 | 0.1606 | 8.819935 | high |
| TCGA-DV-5565 | 0.1344 | 0.1024 | 0.9485 | 0.0913 | 0.0991 | 0.381105 | low |
| TCGA-CZ-5989 | 0.1516 | 0.056356 | 0.9434 | 0.2306 | 0.0763 | 0.333214 | low |
| TCGA-BP-4173 | 0.1603 | 0.1077 | 0.9138 | 0.2815 | 0.1207 | 0.913152 | high |
| TCGA-CJ-4870 | 0.1225 | 0.0573 | 0.9621 | 0.0602 | 0.1209 | 0.409335 | low |
| TCGA-BP-4974 | 0.2159 | 0.2269 | 0.8501 | 0.4332 | 0.1508 | 4.283327 | high |
| TCGA-B0-5075 | 0.2182 | 0.0989 | 0.9006 | 0.3983 | 0.1139 | 1.601409 | high |
| TCGA-BP-4162 | 0.1847 | 0.1093 | 0.9745 | 0.2626 | 0.0928 | 0.631952 | low |
| TCGA-B0-4839 | 0.1451 | 0.0564 | 1 | 0.2355 | 0.1169 | 0.525401 | low |
| TCGA-B8-5553 | 0.1475 | 0.028 | 0.9819 | 0.2027 | 0.0934 | 0.351201 | low |
| TCGA-CJ-4893 | 0.1384 | 0.0541 | 0.9805 | 0.0614 | 0.1079 | 0.368399 | low |
| TCGA-CJ-6033 | 0.1845 | 0.0988 | 0.9166 | 0.0994 | 0.1001 | 0.658104 | low |
| TCGA-B0-4827 | 0.1769 | 0.0608 | 0.8852 | 0.0924 | 0.1991 | 2.844253 | high |
| TCGA-BP-4977 | 0.216 | 0.0734 | 0.9022 | 0.4453 | 0.1348 | 2.163666 | high |
| TCGA-BP-5187 | 0.1813 | 0.0885 | 1 | 0.189 | 0.0995 | 0.566732 | low |
| TCGA-CJ-4882 | 0.1954 | 0.1521 | 0.8921 | 0.391 | 0.1485 | 2.534799 | high |
| TCGA-CJ-4899 | 0.1749 | 0.1105 | 0.9579 | 0.1609 | 0.1473 | 1.263348 | high |
| TCGA-B8-4620 | 0.1851 | 0.1416 | 0.9395 | 0.3915 | 0.1358 | 1.662771 | high |
| TCGA-CJ-4868 | 0.1942 | 0.193 | 0.9828 | 0.2013 | 0.1431 | 1.664966 | high |
| TCGA-BP-5001 | 0.2195 | 0.1288 | 0.7194 | 0.4462 | 0.0872 | 1.80053 | high |
| TCGA-DV-5575 | 0.1533 | 0.0698 | 0.9837 | 0.1608 | 0.1279 | 0.661613 | low |
| TCGA-CJ-4920 | 0.1526 | 0.1665 | 0.855 | 0.3883 | 0.1267 | 1.361488 | high |
| TCGA-B0-4707 | 0.1516 | 0.159 | 0.9091 | 0.1774 | 0.1786 | 2.102906 | high |
| TCGA-AK-3426 | 0.2585 | 0.3661 | 0.625 | 0.5103 | 0.1081 | 7.875236 | high |
| TCGA-B0-4713 | 0.305 | 0.083 | 1 | 0.4021 | 0.1134 | 2.726345 | high |
| TCGA-B0-5705 | 0.1597 | 0.0469 | 0.9359 | 0.066 | 0.0835 | 0.333987 | low |
| TCGA-BP-5199 | 0.174 | 0.1391 | 0.9349 | 0.3773 | 0.1175 | 1.115778 | high |
| TCGA-CZ-5988 | 0.225 | 0.1416 | 0.9587 | 0.1729 | 0.1375 | 1.851606 | high |
| TCGA-BP-5004 | 0.177 | 0.1142 | 0.9147 | 0.1933 | 0.0977 | 0.68119 | low |
| TCGA-A3-3370 | 0.1869 | 0.1197 | 0.8994 | 0.4559 | 0.1158 | 1.384546 | high |
| TCGA-B0-5696 | 0.1712 | 0.0487 | 0.9543 | 0.0672 | 0.1159 | 0.594613 | low |
| TCGA-BP-4797 | 0.1782 | 0.0812 | 0.9796 | 0.1435 | 0.1246 | 0.800391 | low |
| TCGA-CZ-5982 | 0.1317 | 0.1131 | 0.946 | 0.1221 | 0.1027 | 0.41876 | low |
| TCGA-BP-4991 | 0.1637 | 0.1329 | 0.9564 | 0.3069 | 0.1348 | 1.159007 | high |
| TCGA-BP-4762 | 0.1361 | 0.0384 | 1 | 0.0623 | 0.0972 | 0.282572 | low |
| TCGA-A3-3362 | 0.1857 | 0.0892 | 0.9055 | 0.368 | 0.1188 | 1.203913 | high |
| TCGA-A3-A8OW | 0.1539 | 0.0785 | 0.9098 | 0.3485 | 0.0879 | 0.525493 | low |
| TCGA-CJ-4872 | 0.1687 | 0.075 | 0.8613 | 0.3919 | 0.1464 | 1.747152 | high |
| TCGA-B0-4847 | 0.1526 | 0.2676 | 1 | 0.4413 | 0.0851 | 0.674378 | low |
| TCGA-B0-4822 | 0.1623 | 0.2763 | 0.9019 | 0.5981 | 0.1248 | 2.07198 | high |
| TCGA-BP-5175 | 0.1309 | 0.1126 | 0.9516 | 0.0848 | 0.1149 | 0.476531 | low |
| TCGA-BP-4340 | 0.2055 | 0.1608 | 0.8462 | 0.4476 | 0.0615 | 0.854449 | low |
| TCGA-BP-4987 | 0.1746 | 0.0767 | 0.9825 | 0.1576 | 0.1062 | 0.580059 | low |
| TCGA-BP-5189 | 0.192 | 0.0748 | 0.9296 | 0.1833 | 0.1397 | 1.326439 | high |
| TCGA-BP-5000 | 0.1568 | 0.1086 | 0.8235 | 0.1524 | 0.1336 | 1.152666 | high |
| TCGA-AK-3451 | 0.1656 | 0.0478 | 1 | 0.1029 | 0.1181 | 0.547812 | low |
| TCGA-BP-5007 | 0.1798 | 0.0722 | 0.909 | 0.2913 | 0.1351 | 1.294913 | high |
| TCGA-A3-3363 | 0.1984 | 0.055 | 0.8853 | 0.3069 | 0.0946 | 0.841177 | low |
| TCGA-AK-3455 | 0.1609 | 0.081 | 0.9162 | 0.2127 | 0.1045 | 0.620218 | low |
| TCGA-B8-4146 | 0.1981 | 0 | 1 | 0.4141 | 0.1215 | 0.988363 | high |
| TCGA-BP-4770 | 0.2082 | 0.0935 | 1 | 0.0094 | 0.209 | 3.344974 | high |
| TCGA-A3-3378 | 0.1754 | 0.1803 | 0.9422 | 0.3935 | 0.1466 | 1.950942 | high |
| TCGA-DV-5573 | 0.1535 | 0.0865 | 0.9139 | 0.1502 | 0.1054 | 0.558224 | low |
| TCGA-BP-4962 | 0.2033 | 0.0621 | 0.8993 | 0.0254 | 0.1128 | 0.844641 | low |
| TCGA-B8-5546 | 0.1454 | 0.104 | 0.9768 | 0.0299 | 0.1263 | 0.568078 | low |
| TCGA-BP-4347 | 0.1696 | 0.1097 | 0.9295 | 0.277 | 0.1003 | 0.696579 | low |
| TCGA-A3-3343 | 0.1554 | 0.081 | 0.9405 | 0.308 | 0.1099 | 0.67502 | low |
| TCGA-CJ-4918 | 0.177 | 0.1054 | 0.9833 | 0.1695 | 0.1463 | 1.19572 | high |
| TCGA-BP-5201 | 0.1647 | 0.0972 | 0.9638 | 0.1489 | 0.0748 | 0.349633 | low |
| TCGA-CZ-5459 | 0.1376 | 0.0691 | 0.9791 | 0.2148 | 0.1563 | 0.957808 | high |
| TCGA-BP-5170 | 0.143 | 0.0463 | 0.9775 | 0.1148 | 0.1727 | 1.113993 | high |
| TCGA-B0-5094 | 0.2064 | 0.0885 | 0.922 | 0.4236 | 0.156 | 2.665648 | high |
| TCGA-BP-4973 | 0.172 | 0.0817 | 0.8899 | 0.3837 | 0.0778 | 0.579227 | low |
| TCGA-CJ-5684 | 0.1595 | 0.0537 | 0.9428 | 0.1465 | 0.1071 | 0.526829 | low |
| TCGA-BP-4771 | 0.221 | 0.1953 | 0.8428 | 0.4157 | 0.1566 | 4.579497 | high |
| TCGA-B0-5116 | 0.1811 | 0.1312 | 1 | 0.265 | 0.1246 | 0.998985 | high |
| TCGA-CW-5585 | 0.1458 | 0.0517 | 0.975 | 0.0665 | 0.0995 | 0.349997 | low |
| TCGA-CJ-4881 | 0.1516 | 0.2135 | 0.8219 | 0.3962 | 0.1283 | 1.662623 | high |
| TCGA-A3-3323 | 0.2011 | 0.2034 | 0.8911 | 0.5069 | 0.198 | 7.373933 | high |
| TCGA-A3-3380 | 0.2055 | 0.0802 | 0.9824 | 0.2335 | 0.1053 | 0.830983 | low |
| TCGA-B0-4834 | 0.1701 | 0.1837 | 0.9016 | 0.492 | 0.1361 | 1.938982 | high |
| TCGA-AK-3450 | 0.1652 | 0.0791 | 1 | 0.0617 | 0.0754 | 0.28565 | low |
| TCGA-CZ-5461 | 0.1712 | 0.1059 | 0.9366 | 0.1036 | 0.1334 | 0.957909 | high |
| TCGA-A3-3347 | 0.218 | 0.2217 | 0.9181 | 0.4567 | 0.1465 | 3.555453 | high |
| TCGA-B0-5110 | 0.1988 | 0.1209 | 0.9459 | 0.3022 | 0.1008 | 0.930522 | high |
| TCGA-AK-3460 | 0.1825 | 0.0525 | 0.8824 | 0.3388 | 0.1179 | 1.08681 | high |
| TCGA-CJ-4908 | 0.2059 | 0.1015 | 0.8888 | 0.4689 | 0.1205 | 1.771305 | high |
| TCGA-BP-4774 | 0.1992 | 0.0854 | 0.8681 | 0.3596 | 0.1294 | 1.718915 | high |
| TCGA-B0-4842 | 0.0514 | 0.4853 | 1 | 1 | 0.1538 | 2.316483 | high |
| TCGA-B0-5812 | 0.1433 | 0.0634 | 0.9515 | 0.1075 | 0.1226 | 0.555671 | low |
| TCGA-B0-4816 | 0.1925 | 0.1972 | 0.9743 | 0.3974 | 0.149 | 2.294762 | high |
| TCGA-BP-4988 | 0.2087 | 0.2789 | 0.8345 | 0.6753 | 0.1343 | 4.679973 | high |
| TCGA-B0-4945 | 0.1741 | 0.0873 | 0.8908 | 0.2978 | 0.1035 | 0.811764 | low |
| TCGA-DV-5576 | 0.1493 | 0.0676 | 0.9418 | 0.2611 | 0.136 | 0.88435 | low |
| TCGA-MM-A84U | 0.2034 | 0.1181 | 1 | 0.3784 | 0.12 | 1.255217 | high |
| TCGA-BP-4998 | 0.1863 | 0.0889 | 0.873 | 0.3101 | 0.1594 | 2.307387 | high |
| TCGA-A3-3387 | 0.1898 | 0.0969 | 0.8921 | 0.2119 | 0.1539 | 1.911896 | high |
| TCGA-BP-4968 | 0.163 | 0.1091 | 0.9408 | 0.1767 | 0.113 | 0.697792 | low |
| TCGA-BP-4995 | 0.1751 | 0.0959 | 0.9507 | 0.0693 | 0.1098 | 0.62615 | low |
| TCGA-CJ-4890 | 0.1894 | 0.1439 | 0.925 | 0.1915 | 0.1464 | 1.701264 | high |
| TCGA-B0-5092 | 0.201 | 0.1984 | 0.8072 | 0.4012 | 0.1337 | 2.8592 | high |
| TCGA-DV-A4VZ | 0.1936 | 0.1328 | 0.7976 | 0.475 | 0.1337 | 2.572682 | high |
| TCGA-A3-3385 | 0.1073 | 0.1123 | 0.9062 | 0.4093 | 0.0689 | 0.295367 | low |
| TCGA-B0-4823 | 0.1522 | 0.0147 | 0.9546 | 0.0552 | 0.1408 | 0.677081 | low |
| TCGA-BP-4992 | 0.3026 | 0.425 | 0.7 | 0.6681 | 0.0924 | 10.50778 | high |
| TCGA-B8-4151 | 0.1498 | 0.0668 | 0.9703 | 0.3001 | 0.0941 | 0.450256 | low |
| TCGA-B4-5838 | 0.1283 | 0 | 0.9694 | 0.0933 | 0.1002 | 0.281503 | low |
| TCGA-BP-4986 | 0.1781 | 0.055 | 0.9002 | 0.2893 | 0.1231 | 1.036108 | high |
| TCGA-CZ-5984 | 0.1618 | 0.071 | 0.9768 | 0.0734 | 0.1133 | 0.526049 | low |
| TCGA-BP-4344 | 0.1606 | 0.0698 | 0.871 | 0.243 | 0.1154 | 0.819768 | low |
| TCGA-CZ-4854 | 0.1966 | 0.1738 | 0.9877 | 0.2504 | 0.1286 | 1.35835 | high |
| TCGA-CJ-6032 | 0.1561 | 0.0505 | 0.9757 | 0.0582 | 0.1052 | 0.414929 | low |
| TCGA-BP-4355 | 0.2246 | 0.1746 | 0.7946 | 0.4324 | 0.1093 | 2.448007 | high |
| TCGA-B0-4814 | 0.1466 | 0.134 | 0.732 | 0.2684 | 0.128 | 1.417288 | high |
| TCGA-BP-4759 | 0.202 | 0.045 | 0.98 | 0.066 | 0.1302 | 0.921965 | high |
| TCGA-B0-5097 | 0.1991 | 0.1108 | 0.8619 | 0.3072 | 0.1786 | 3.753771 | high |
| TCGA-CJ-4923 | 0.1709 | 0.1429 | 0.8366 | 0.2232 | 0.1303 | 1.406708 | high |
| TCGA-DV-A4VX | 0.0769 | 0.1825 | 0.9139 | 0.3497 | 0.1126 | 0.474941 | low |
| TCGA-CJ-5671 | 0.1438 | 0.0669 | 0.8637 | 0.1236 | 0.1315 | 0.801659 | low |
| TCGA-BP-5191 | 0.1885 | 0.1271 | 0.7838 | 0.2934 | 0.1493 | 2.616483 | high |
| TCGA-BP-4158 | 0.1845 | 0.113 | 0.976 | 0.5471 | 0.1058 | 1.062491 | high |
| TCGA-BP-4768 | 0.2408 | 0.0471 | 0.9248 | 0.4702 | 0.1399 | 2.71437 | high |
| TCGA-B2-5639 | 0.1924 | 0.1082 | 0.9778 | 0.1157 | 0.0925 | 0.568532 | low |
| TCGA-B0-5084 | 0.209 | 0.3098 | 0.7826 | 0.2942 | 0.1803 | 7.618858 | high |
| TCGA-CJ-5680 | 0.1681 | 0.0795 | 0.9761 | 0.3021 | 0.1239 | 0.86313 | low |
| TCGA-CJ-4639 | 0.1795 | 0.0783 | 0.9626 | 0.2298 | 0.1336 | 1.058887 | high |
| TCGA-B8-5159 | 0.1556 | 0.104 | 0.9624 | 0.1094 | 0.072 | 0.3001 | low |
| TCGA-CJ-4641 | 0.2086 | 0.1495 | 0.8938 | 0.4332 | 0.1332 | 2.334815 | high |
| TCGA-B0-5692 | 0.1765 | 0.0495 | 0.972 | 0.4308 | 0.0739 | 0.464061 | low |
| TCGA-CJ-4895 | 0.181 | 0.1807 | 0.9638 | 0.2317 | 0.1737 | 2.505752 | high |
| TCGA-BP-4959 | 0.1635 | 0.0525 | 1 | 0.0495 | 0.1219 | 0.543286 | low |
| TCGA-BP-4787 | 0.1646 | 0.0778 | 0.9826 | 0.2716 | 0.1599 | 1.395606 | high |
| TCGA-AK-3465 | 0.184 | 0.0888 | 0.9182 | 0.0939 | 0.1152 | 0.804635 | low |
| TCGA-BP-5009 | 0.2151 | 0.0917 | 0.9633 | 0.2719 | 0.1411 | 1.774584 | high |
| TCGA-EU-5904 | 0.1746 | 0.041 | 0.9638 | 0.1599 | 0.1086 | 0.583293 | low |
| TCGA-A3-3306 | 0.217 | 0.0407 | 0.9816 | 0.0549 | 0.1417 | 1.234963 | high |
| TCGA-BP-5192 | 0.1495 | 0.0303 | 0.9679 | 0.1184 | 0.0885 | 0.313076 | low |
| TCGA-BP-4784 | 0.1342 | 0.0425 | 0.8455 | 0.0496 | 0.1499 | 0.892918 | low |
| TCGA-B8-4153 | 0.1519 | 0.2323 | 1 | 0.5172 | 0.0868 | 0.693548 | low |
| TCGA-B0-5081 | 0.1992 | 0.0872 | 0.8983 | 0.2831 | 0.1533 | 2.155851 | high |
| TCGA-G6-A8L6 | 0.1595 | 0.1395 | 0.9285 | 0.3461 | 0.111 | 0.865955 | low |
| TCGA-AK-3445 | 0.1553 | 0.1452 | 0.9525 | 0.2532 | 0.1631 | 1.6331 | high |
| TCGA-BP-4351 | 0.1978 | 0.1753 | 0.8092 | 0.701 | 0.1226 | 3.074464 | high |
| TCGA-B0-5100 | 0.221 | 0.221 | 1 | 0.3035 | 0.1453 | 2.51698 | high |
| TCGA-B0-5700 | 0.1237 | 0.0621 | 0.9471 | 0.0787 | 0.096 | 0.298728 | low |
| TCGA-AK-3429 | 0.131 | 0.0445 | 0.9194 | 0.0724 | 0.0652 | 0.20053 | low |
| TCGA-BP-4798 | 0.3711 | 0.1405 | 0.8162 | 0.3692 | 0.1496 | 14.50312 | high |
| TCGA-BP-4769 | 0.185 | 0.0463 | 0.9312 | 0.1373 | 0.148 | 1.262744 | high |
| TCGA-CJ-5678 | 0.1053 | 0.0434 | 1 | 0.1049 | 0.1162 | 0.304276 | low |
| TCGA-A3-3316 | 0.2108 | 0.171 | 1 | 0.2468 | 0.1289 | 1.496496 | high |
| TCGA-B8-5550 | 0.1619 | 0.0396 | 0.9693 | 0.1703 | 0.1221 | 0.639283 | low |
| TCGA-B0-5699 | 0.1368 | 0.0612 | 0.9327 | 0.1971 | 0.1165 | 0.545259 | low |
| TCGA-CJ-4888 | 0.1753 | 0.2652 | 0.9504 | 0.2976 | 0.1536 | 2.305592 | high |
| TCGA-BP-4354 | 0.2213 | 0.1859 | 0.8963 | 0.3472 | 0.1112 | 1.816615 | high |
| TCGA-BP-4989 | 0.2058 | 0.2272 | 0.8746 | 0.4054 | 0.082 | 1.220207 | high |
| TCGA-CJ-4873 | 0.1603 | 0.171 | 0.9362 | 0.8237 | 0.0961 | 1.228293 | high |
| TCGA-B0-4824 | 0.1961 | 0.1916 | 0.9441 | 0.5334 | 0.1067 | 1.50069 | high |
| TCGA-CZ-5455 | 0.1694 | 0.0812 | 0.954 | 0.1581 | 0.1092 | 0.624443 | low |
| TCGA-BP-4164 | 0.1669 | 0.148 | 0.9103 | 0.0737 | 0.0955 | 0.571276 | low |
| TCGA-CW-5581 | 0.1607 | 0.1047 | 0.9887 | 0.0721 | 0.1212 | 0.616045 | low |
| TCGA-A3-3313 | 0.1798 | 0.139 | 0.8623 | 0.1148 | 0.186 | 3.035307 | high |
| TCGA-B0-4845 | 0.1959 | 0.1461 | 0.9348 | 0.4075 | 0.1255 | 1.621789 | high |
| TCGA-B4-5378 | 0.1369 | 0.0438 | 0.9635 | 0.2547 | 0.1537 | 0.936507 | high |
| TCGA-BP-5174 | 0.1488 | 0.09 | 0.927 | 0.1264 | 0.1093 | 0.541772 | low |
| TCGA-BP-4801 | 0.1487 | 0.0574 | 1 | 0.1409 | 0.1426 | 0.733599 | low |
| TCGA-CJ-5686 | 0.1612 | 0.089 | 0.973 | 0.2783 | 0.1074 | 0.62643 | low |
| TCGA-A3-3349 | 0.191 | 0.0789 | 0.9164 | 0.3388 | 0.1222 | 1.231939 | high |
| TCGA-DV-5567 | 0.1456 | 0.0585 | 0.9581 | 0.137 | 0.1253 | 0.596236 | low |
| TCGA-CZ-5987 | 0.2419 | 0.0698 | 0.9461 | 0.1461 | 0.1214 | 1.437448 | high |
| TCGA-CJ-4885 | 0.1694 | 0.0577 | 0.8772 | 0.1613 | 0.0978 | 0.592153 | low |
| TCGA-DV-5574 | 0.1899 | 0.0741 | 0.9253 | 0.1963 | 0.1572 | 1.750822 | high |
| TCGA-A3-3331 | 0.1907 | 0.0911 | 0.8552 | 0.3207 | 0.1095 | 1.162408 | high |
| TCGA-BP-5178 | 0.151 | 0.0402 | 0.9304 | 0.0513 | 0.0968 | 0.373118 | low |
| TCGA-CJ-4871 | 0.156 | 0.0592 | 1 | 0.0944 | 0.0909 | 0.332605 | low |
| TCGA-B0-4836 | 0.1614 | 0.0572 | 0.944 | 0.1845 | 0.1357 | 0.87934 | low |
| TCGA-B8-A54I | 0.1626 | 0.1181 | 0.9141 | 0.1549 | 0.0907 | 0.518014 | low |
| TCGA-B0-4821 | 0.2078 | 0.3729 | 0.5701 | 0.8517 | 0.1183 | 9.717232 | high |
| TCGA-BP-4777 | 0.1825 | 0.1046 | 0.9352 | 0.1509 | 0.1217 | 0.931694 | high |
| TCGA-CJ-4636 | 0.1756 | 0.1502 | 0.8092 | 0.1489 | 0.1274 | 1.396809 | high |
| TCGA-B0-5711 | 0.1533 | 0.0504 | 0.9635 | 0.0732 | 0.0965 | 0.368648 | low |
| TCGA-CZ-4857 | 0.1475 | 0.1573 | 0.8245 | 0.4572 | 0.1657 | 2.712547 | high |
| TCGA-B0-4852 | 0.1841 | 0.1852 | 0.8785 | 0.2588 | 0.1064 | 1.130931 | high |
| TCGA-A3-3329 | 0.1613 | 0.0486 | 0.921 | 0.2633 | 0.1121 | 0.684192 | low |
| TCGA-CZ-5466 | 0.1658 | 0.0825 | 0.885 | 0.0983 | 0.139 | 1.056857 | high |
| TCGA-BP-5177 | 0.1895 | 0.0479 | 0.9084 | 0.2009 | 0.1238 | 1.019885 | high |
| TCGA-A3-3372 | 0.1795 | 0.1404 | 0.9037 | 0.3095 | 0.1101 | 1.042973 | high |
| TCGA-BP-4804 | 0.1878 | 0.1593 | 0.9544 | 0.2892 | 0.1212 | 1.216825 | high |
| TCGA-AK-3434 | 0.2337 | 0.135 | 0.8094 | 0.1793 | 0.1136 | 1.913881 | high |
| TCGA-B0-4811 | 0.2318 | 0.4247 | 0.8028 | 0.8233 | 0.0841 | 4.546361 | high |
| TCGA-BP-4165 | 0.1497 | 0.1157 | 0.9649 | 0.3426 | 0.0947 | 0.534918 | low |
| TCGA-B0-5099 | 0.2081 | 0.1766 | 0.933 | 0.3706 | 0.0851 | 0.99065 | high |
| TCGA-BP-4766 | 0.1765 | 0.0651 | 0.9257 | 0.2272 | 0.1166 | 0.831128 | low |
| TCGA-BP-4335 | 0.181 | 0.1101 | 0.7367 | 0.6971 | 0.1267 | 2.864972 | high |
| TCGA-B0-4703 | 0.196 | 0.1133 | 0.9415 | 0.2944 | 0.1752 | 2.868048 | high |
| TCGA-CJ-4904 | 0.1552 | 0.0711 | 0.9427 | 0.0339 | 0.092 | 0.366703 | low |
| TCGA-BP-4352 | 0.3624 | 0.2775 | 0.8789 | 0.5695 | 0.1375 | 16.09801 | high |
| TCGA-CJ-4635 | 0.2102 | 0.1402 | 0.8504 | 0.2842 | 0.1358 | 2.263444 | high |
| TCGA-CJ-5679 | 0.1806 | 0.0903 | 0.9682 | 0.1405 | 0.1007 | 0.586529 | low |
| TCGA-CZ-4862 | 0.1623 | 0.0916 | 0.9376 | 0.4593 | 0.1314 | 1.224806 | high |
| TCGA-B8-4622 | 0.1838 | 0.1142 | 0.9246 | 0.3238 | 0.104 | 0.903325 | low |
| TCGA-G6-A8L8 | 0.1704 | 0.1227 | 0.8196 | 0.4464 | 0.1158 | 1.418825 | high |
| TCGA-CJ-5682 | 0.184 | 0.1803 | 1 | 0.0655 | 0.0875 | 0.511588 | low |
| TCGA-BP-4176 | 0.1783 | 0.0979 | 1 | 0.1059 | 0.1344 | 0.887375 | low |
| TCGA-B8-4148 | 0.1416 | 0.1502 | 0.9767 | 0.2687 | 0.0877 | 0.430341 | low |
| TCGA-CJ-4886 | 0.1708 | 0.134 | 0.971 | 0.3314 | 0.109 | 0.82246 | low |
| TCGA-BP-5186 | 0.1516 | 0.0549 | 0.9358 | 0.26 | 0.1216 | 0.709929 | low |
| TCGA-BP-4972 | 0.1613 | 0.0595 | 0.9359 | 0.1765 | 0.1086 | 0.582767 | low |
| TCGA-BP-4993 | 0.1666 | 0.194 | 0.8522 | 0.3194 | 0.1572 | 2.469631 | high |
| TCGA-CW-6097 | 0.149 | 0.1074 | 0.9418 | 0.2174 | 0.162 | 1.376048 | high |
| TCGA-B0-5077 | 0.1896 | 0.0591 | 0.9007 | 0.3379 | 0.1352 | 1.479002 | high |
| TCGA-A3-3383 | 0.1676 | 0.1068 | 0.9221 | 0.1213 | 0.1313 | 0.946637 | high |
| TCGA-B0-4819 | 0.188 | 0.181 | 0.903 | 0.3186 | 0.1339 | 1.806699 | high |
| TCGA-BP-4775 | 0.1799 | 0.0942 | 0.9352 | 0.2257 | 0.077 | 0.479412 | low |
| TCGA-BP-4327 | 0.2218 | 0.2257 | 0.6621 | 0.3987 | 0.1195 | 4.054127 | high |
| TCGA-B0-5399 | 0.1633 | 0.2075 | 0.8281 | 0.3126 | 0.194 | 4.601459 | high |
| TCGA-A3-3357 | 0.1832 | 0.1252 | 0.962 | 0.2627 | 0.1334 | 1.253653 | high |
| TCGA-B0-4694 | 0.1279 | 0.0805 | 0.9175 | 0.099 | 0.1168 | 0.488945 | low |
| TCGA-A3-3317 | 0.1564 | 0.2548 | 0.9743 | 0.2688 | 0.1353 | 1.307486 | high |
| TCGA-B8-4154 | 0.1714 | 0.0861 | 1 | 0.2186 | 0.101 | 0.544768 | low |
| TCGA-B2-5636 | 0.144 | 0.0712 | 0.9209 | 0.138 | 0.1165 | 0.57248 | low |
| TCGA-B0-4817 | 0.1825 | 0.0912 | 0.8784 | 0.4077 | 0.0974 | 0.932144 | high |
| TCGA-BP-5010 | 0.1825 | 0.134 | 0.658 | 0.5773 | 0.0947 | 1.936507 | high |
| TCGA-BP-4325 | 0.1441 | 0.1271 | 0.8859 | 0.235 | 0.1247 | 0.884218 | low |
| TCGA-BP-5008 | 0.1743 | 0.0529 | 0.9508 | 0.1982 | 0.1048 | 0.603807 | low |
| TCGA-B0-5707 | 0.087 | 0.044 | 0.9659 | 0.1353 | 0.1265 | 0.338156 | low |
| TCGA-CZ-5454 | 0.1773 | 0.1153 | 0.9057 | 0.1199 | 0.1356 | 1.167339 | high |
| TCGA-BP-4807 | 0.1549 | 0.0866 | 0.9794 | 0.2199 | 0.1177 | 0.639153 | low |
| TCGA-A3-3352 | 0.2001 | 0.0743 | 0.9611 | 0.0916 | 0.1053 | 0.700866 | low |
| TCGA-B0-5712 | 0.1711 | 0.0635 | 0.9483 | 0.1591 | 0.0807 | 0.396066 | low |
| TCGA-B0-4843 | 0.2299 | 0.2157 | 0.9011 | 0.4325 | 0.0995 | 1.894901 | high |
| TCGA-B8-A54H | 0.1602 | 0.1265 | 0.9223 | 0.2632 | 0.0939 | 0.600044 | low |
| TCGA-CZ-5456 | 0.199 | 0.0702 | 0.9506 | 0.0737 | 0.1604 | 1.637716 | high |
| TCGA-B0-4828 | 0.123 | 0.1402 | 0.9016 | 0.4159 | 0.1416 | 1.15137 | high |
| TCGA-AK-3444 | 0.141 | 0.105 | 0.9649 | 0.2125 | 0.0823 | 0.344516 | low |
| TCGA-CJ-6031 | 0.1872 | 0.0979 | 0.9785 | 0.0664 | 0.1288 | 0.885928 | low |
| TCGA-B8-A54K | 0.1568 | 0.1126 | 0.9317 | 0.3739 | 0.1505 | 1.516284 | high |
| TCGA-DV-5566 | 0.1666 | 0.0459 | 0.9096 | 0.1502 | 0.1147 | 0.673702 | low |
| TCGA-B0-5113 | 0.2046 | 0.0948 | 0.8966 | 0.3549 | 0.1465 | 2.248047 | high |
| TCGA-BP-4169 | 0.1905 | 0.1747 | 0.7731 | 0.3583 | 0.114 | 1.867016 | high |
| TCGA-CZ-5986 | 0.1737 | 0.0387 | 0.962 | 0.1709 | 0.1271 | 0.781854 | low |
| TCGA-CJ-4640 | 0.1426 | 0.165 | 0.9842 | 0.1803 | 0.1296 | 0.77135 | low |
| TCGA-B4-5836 | 0.1647 | 0.0704 | 0.9434 | 0.1876 | 0.1085 | 0.611576 | low |
| TCGA-B0-5713 | 0.1789 | 0.0726 | 0.9123 | 0.1293 | 0.0809 | 0.456495 | low |
| TCGA-BP-4790 | 0.1624 | 0.2634 | 0.9079 | 0.0895 | 0.1105 | 0.90891 | high |
| TCGA-BP-4174 | 0.1757 | 0.0483 | 0.9483 | 0.0885 | 0.0767 | 0.347449 | low |
| TCGA-BP-4161 | 0.2057 | 0.1608 | 0.9353 | 0.3563 | 0.1031 | 1.216564 | high |
| TCGA-BP-5183 | 0.1962 | 0.0573 | 0.94 | 0.2081 | 0.1117 | 0.859292 | low |
| TCGA-CW-5588 | 0.1378 | 0.0308 | 0.9822 | 0.0786 | 0.1263 | 0.472115 | low |
| TCGA-BP-4970 | 0.1574 | 0.0526 | 0.935 | 0.104 | 0.132 | 0.739811 | low |
| TCGA-A3-3319 | 0.107 | 0.1429 | 0.7825 | 0.124 | 0.1322 | 0.818346 | low |
| TCGA-BP-4349 | 0.1817 | 0.1642 | 0.8673 | 0.6177 | 0.0834 | 1.122533 | high |
| TCGA-A3-3335 | 0.1945 | 0.0762 | 0.9082 | 0.1499 | 0.1163 | 0.953956 | high |
| TCGA-CZ-5464 | 0.1563 | 0.0732 | 1 | 0.1156 | 0.1363 | 0.716739 | low |
| TCGA-AK-3436 | 0.1281 | 0.0297 | 1 | 0.0262 | 0.1335 | 0.437666 | low |
| TCGA-AK-3427 | 0.1558 | 0.0454 | 0.955 | 0.0869 | 0.1034 | 0.430214 | low |
| TCGA-BP-4163 | 0.2358 | 0.0661 | 0.8977 | 0.2093 | 0.1069 | 1.284224 | high |
| TCGA-BP-4170 | 0.1881 | 0.0762 | 0.9733 | 0.26 | 0.1427 | 1.327281 | high |
| TCGA-B0-5088 | 0.1842 | 0.0753 | 0.9784 | 0.544 | 0.1178 | 1.169165 | high |
| TCGA-BP-4329 | 0.1418 | 0.0485 | 0.861 | 0.2147 | 0.1118 | 0.617967 | low |
| TCGA-CW-5591 | 0.1541 | 0.1047 | 0.9783 | 0.0937 | 0.1305 | 0.703396 | low |
| TCGA-CW-5580 | 0.1482 | 0.0773 | 0.9327 | 0.3317 | 0.1127 | 0.684373 | low |
| TCGA-BP-4177 | 0.1354 | 0.0205 | 0.9735 | 0.0592 | 0.1557 | 0.714969 | low |
| TCGA-B0-4837 | 0.1914 | 0.159 | 0.9092 | 0.2967 | 0.1418 | 1.937074 | high |
| TCGA-CJ-4907 | 0.162 | 0.0988 | 0.9772 | 0.1183 | 0.101 | 0.484234 | low |
| TCGA-CJ-4891 | 0.2018 | 0.1209 | 0.9615 | 0.6838 | 0.1502 | 3.048335 | high |
| TCGA-A3-3365 | 0.2042 | 0.1126 | 0.8879 | 0.3831 | 0.0622 | 0.652955 | low |
| TCGA-BP-4982 | 0.1896 | 0.1066 | 0.9502 | 0.1995 | 0.1112 | 0.86382 | low |
| TCGA-B8-5549 | 0.1687 | 0.0862 | 0.9525 | 0.3113 | 0.0906 | 0.556447 | low |
| TCGA-MM-A563 | 0.1645 | 0.0937 | 0.8716 | 0.2028 | 0.1005 | 0.676406 | low |
| TCGA-B0-4688 | 0.1631 | 0.2669 | 0.8256 | 0.062 | 0.1955 | 4.07706 | high |
| TCGA-BP-5194 | 0.1932 | 0.0616 | 0.9519 | 0.0874 | 0.129 | 0.943471 | high |
| TCGA-CJ-4634 | 0.1519 | 0.1427 | 0.9078 | 0.2103 | 0.1076 | 0.695778 | low |
| TCGA-CZ-5462 | 0.1983 | 0.0812 | 0.9133 | 0.0375 | 0.1195 | 0.91699 | high |
| TCGA-CJ-4902 | 0.1578 | 0.1585 | 0.8695 | 0.3194 | 0.1254 | 1.233888 | high |
| TCGA-BP-4981 | 0.1777 | 0.1193 | 0.9069 | 0.4527 | 0.1263 | 1.469135 | high |
| TCGA-A3-A6NI | 0.1523 | 0.0712 | 0.8991 | 0.2122 | 0.1356 | 0.94939 | high |
| TCGA-BP-5195 | 0.1416 | 0.0535 | 0.8845 | 0.184 | 0.1103 | 0.558503 | low |
| TCGA-BP-4782 | 0.1922 | 0.0771 | 0.9588 | 0.1232 | 0.1304 | 1.011836 | high |
| TCGA-A3-3311 | 0.1613 | 0.1098 | 0.9779 | 0.2317 | 0.1223 | 0.7781 | low |
| TCGA-CZ-4864 | 0.1529 | 0.1209 | 0.9072 | 0.2922 | 0.0964 | 0.616032 | low |
| TCGA-B2-4102 | 0.1768 | 0.1895 | 0.8968 | 0.3326 | 0.1203 | 1.382025 | high |
| TCGA-CJ-4878 | 0.204 | 0.3676 | 1 | 0.3529 | 0.0806 | 1.130238 | high |
| TCGA-AK-3428 | 0.0791 | 0.1111 | 1 | 0.1385 | 0.1542 | 0.521348 | low |
| TCGA-BP-4971 | 0.2267 | 0.214 | 0.8187 | 0.5078 | 0.1413 | 4.615552 | high |
| TCGA-CJ-5676 | 0.1387 | 0.1 | 0.8892 | 0.0447 | 0.0816 | 0.325323 | low |
| TCGA-B8-A54J | 0.1842 | 0.0943 | 0.9235 | 0.3756 | 0.0979 | 0.837894 | low |
| TCGA-BP-4999 | 0.1649 | 0.0593 | 0.9514 | 0.2698 | 0.117 | 0.734925 | low |
| TCGA-BP-4332 | 0.1662 | 0.1665 | 0.8993 | 0.4727 | 0.1137 | 1.249112 | high |
| TCGA-CZ-4863 | 0.1572 | 0.1424 | 0.9281 | 0.298 | 0.1012 | 0.694642 | low |
| TCGA-A3-3374 | 0.093 | 0.1184 | 0.9798 | 0.1462 | 0.1168 | 0.353074 | low |
| TCGA-A3-3326 | 0.1164 | 0.0691 | 0.93 | 0.2874 | 0.1034 | 0.416501 | low |
| TCGA-T7-A92I | 0.129 | 0.0679 | 0.9555 | 0.2066 | 0.1065 | 0.422782 | low |
| TCGA-AK-3425 | 0.18 | 0.0875 | 0.9144 | 0.1787 | 0.1051 | 0.731117 | low |
| TCGA-CJ-4894 | 0.1696 | 0.0911 | 0.917 | 0.2572 | 0.1079 | 0.758768 | low |
| TCGA-AK-3458 | 0.1719 | 0.0955 | 0.9505 | 0.298 | 0.0904 | 0.576795 | low |
| TCGA-B0-5106 | 0.2333 | 0.1364 | 0.9122 | 0.6444 | 0.1311 | 3.332278 | high |
| TCGA-A3-3382 | 0.1352 | 0.0729 | 0.9051 | 0.2528 | 0.1388 | 0.884107 | low |
| TCGA-BP-4353 | 0.2382 | 0.1619 | 0.859 | 0.2716 | 0.0922 | 1.495812 | high |
| TCGA-B0-5698 | 0.1511 | 0.0906 | 1 | 0.1152 | 0.0763 | 0.276879 | low |
| TCGA-CZ-4866 | 0.1834 | 0.0668 | 0.9429 | 0.1665 | 0.0943 | 0.563688 | low |
| TCGA-BP-4799 | 0.1154 | 0.3661 | 0.9331 | 0.2226 | 0.0801 | 0.500929 | low |
| TCGA-CW-5583 | 0.1276 | 0.0486 | 0.9343 | 0.1612 | 0.109 | 0.41551 | low |
| TCGA-B0-5085 | 0.2844 | 0.2226 | 0.7374 | 0.7942 | 0.0809 | 5.083788 | high |
| TCGA-CJ-5683 | 0.1742 | 0.06 | 0.9559 | 0.0821 | 0.0999 | 0.49352 | low |
| TCGA-CW-5590 | 0.1644 | 0.0643 | 0.9691 | 0.0752 | 0.0983 | 0.427915 | low |
| TCGA-CZ-5457 | 0.1332 | 0.0753 | 0.9251 | 0.1113 | 0.0969 | 0.370202 | low |
| TCGA-B8-A7U6 | 0.1829 | 0.087 | 0.9473 | 0.2708 | 0.1227 | 1.015691 | high |
| TCGA-BP-5185 | 0.1702 | 0.0478 | 1 | 0.0987 | 0.0651 | 0.247784 | low |
| TCGA-BP-4763 | 0.1659 | 0.0468 | 0.9411 | 0.1213 | 0.0851 | 0.380615 | low |
| TCGA-BP-5180 | 0.1618 | 0.0536 | 0.956 | 0.044 | 0.1476 | 0.880147 | low |
| TCGA-B2-5635 | 0.1451 | 0.1171 | 0.9148 | 0.3114 | 0.1152 | 0.767106 | low |
| TCGA-BP-4789 | 0.154 | 0.1284 | 0.9856 | 0.1335 | 0.1121 | 0.569539 | low |
| TCGA-A3-A8OV | 0.1636 | 0.0803 | 0.9013 | 0.1932 | 0.087 | 0.488302 | low |
| TCGA-CJ-4643 | 0.1486 | 0.1332 | 0.9574 | 0.2984 | 0.0953 | 0.537659 | low |
| TCGA-BP-4326 | 0.1979 | 0.1936 | 0.8904 | 0.2467 | 0.1049 | 1.22581 | high |
| TCGA-BP-4960 | 0.2107 | 0.1435 | 0.7626 | 0.2645 | 0.1277 | 2.403911 | high |
| TCGA-B0-5121 | 0.1516 | 0.0743 | 0.9381 | 0.336 | 0.1037 | 0.604656 | low |
| TCGA-B2-4099 | 0.1805 | 0.0591 | 0.9185 | 0.2141 | 0.0821 | 0.496421 | low |
| TCGA-BP-5168 | 0.1425 | 0.0773 | 0.9667 | 0.0721 | 0.1417 | 0.712651 | low |
| TCGA-DV-A4W0 | 0.1618 | 0.1319 | 0.9392 | 0.2949 | 0.1324 | 1.122845 | high |
| TCGA-CJ-4905 | 0.1611 | 0.0738 | 0.9298 | 0.1476 | 0.1328 | 0.860815 | low |
| TCGA-CJ-5675 | 0.1598 | 0.0812 | 0.9744 | 0.0403 | 0.0753 | 0.282116 | low |
| TCGA-AK-3443 | 0.1516 | 0.0994 | 0.9414 | 0.0992 | 0.1147 | 0.579967 | low |
| TCGA-MW-A4EC | 0.1843 | 0.0882 | 0.8584 | 0.2157 | 0.1143 | 1.039057 | high |
| TCGA-A3-3325 | 0.1825 | 0.1566 | 0.959 | 0.3222 | 0.1473 | 1.780911 | high |
| TCGA-CZ-5469 | 0.2025 | 0.1047 | 0.8128 | 0.4927 | 0.1274 | 2.345653 | high |
| TCGA-AK-3453 | 0.1607 | 0.071 | 0.9912 | 0.4378 | 0.1202 | 0.838918 | low |
| TCGA-CZ-5452 | 0.1762 | 0.0586 | 0.9723 | 0.1479 | 0.1194 | 0.704906 | low |
| TCGA-CJ-4901 | 0.2387 | 0.2409 | 0.9599 | 0.1785 | 0.1132 | 1.780402 | high |
| TCGA-B0-4700 | 0.1879 | 0.0935 | 0.8823 | 0.1943 | 0.125 | 1.188955 | high |
| TCGA-B2-3923 | 0.2489 | 0.0704 | 0.9039 | 0.1054 | 0.1253 | 1.714723 | high |
| TCGA-BP-5184 | 0.1322 | 0.0506 | 0.9577 | 0.145 | 0.1192 | 0.475959 | low |
| TCGA-B0-4810 | 0.1641 | 0.154 | 0.9564 | 0.3338 | 0.1655 | 2.028534 | high |
| TCGA-BP-5176 | 0.1444 | 0.0483 | 1 | 0.1008 | 0.1198 | 0.462956 | low |
| TCGA-CZ-5463 | 0.1828 | 0.0403 | 0.949 | 0.2021 | 0.1055 | 0.647549 | low |
| TCGA-B2-5633 | 0.1237 | 0.0997 | 0.9478 | 0.1554 | 0.117 | 0.488922 | low |
| TCGA-B8-A54E | 0.1047 | 0.0798 | 0.982 | 0.7937 | 0.1375 | 1.015114 | high |
| TCGA-B0-4844 | 0.1942 | 0.1854 | 0.9517 | 0.448 | 0.1013 | 1.196714 | high |
| TCGA-BP-5169 | 0.1794 | 0.1626 | 0.6824 | 0.2311 | 0.1381 | 2.556115 | high |
| TCGA-B0-4714 | 0.1921 | 0.2448 | 0.9148 | 0.519 | 0.1114 | 1.835201 | high |
| TCGA-CZ-4853 | 0.1627 | 0.064 | 0.9737 | 0.1098 | 0.0939 | 0.404041 | low |

Table S6

| ID | UACA\|31439\|AP | LIMA1\|21691\|AP | ARHGAP24\|69814\|AP | BROX\|9895\|AP | TRIM16L\|39631\|AP | Risk score | risk |
| --- | --- | --- | --- | --- | --- | --- | --- |
| TCGA-CJ-4637 | 0.5675 | 0.3493 | 0.1931 | 0.4968 | 0.2733 | 2.27624 | high |
| TCGA-CZ-4861 | 0.6957 | 0.4511 | 0.1444 | 0.2829 | 0.0597 | 1.495461 | high |
| TCGA-BP-4964 | 0.3407 | 0.4584 | 0.0121 | 0.314 | 0.0599 | 0.571839 | low |
| TCGA-CJ-4887 | 0.4185 | 0.245 | 0.0834 | 0.4892 | 0.3853 | 1.853612 | high |
| TCGA-BP-5198 | 0.6043 | 0.4544 | 0.1226 | 0.4052 | 0.0449 | 1.282691 | high |
| TCGA-BP-4967 | 0.4353 | 0.6322 | 0.075 | 0.3018 | 0.1134 | 0.622727 | low |
| TCGA-A3-3351 | 0.5431 | 0.395 | 0.1299 | 0.2427 | 0.1531 | 1.260097 | high |
| TCGA-B0-4710 | 0.462 | 0.4991 | 0.0575 | 0.3667 | 0.1071 | 0.827149 | low |
| TCGA-B0-5083 | 0.544 | 0.8307 | 0.2359 | 0.3447 | 0.1557 | 0.823566 | low |
| TCGA-BP-4803 | 0.5299 | 0.5536 | 0.0605 | 0.3434 | 0.0168 | 0.770394 | low |
| TCGA-CJ-4889 | 0.285 | 0.3505 | 0.1746 | 0.4112 | 0.0619 | 0.876272 | high |
| TCGA-B0-5080 | 0.4541 | 0.3755 | 0.07 | 0.4822 | 0.0496 | 1.038458 | high |
| TCGA-CW-5587 | 0.1586 | 0.5236 | 0.0206 | 0.3182 | 0.1114 | 0.382225 | low |
| TCGA-A3-3376 | 0.4155 | 0.3735 | 0.0683 | 0.4284 | 0.0864 | 0.959901 | high |
| TCGA-CJ-4897 | 0.2843 | 0.6881 | 0.0284 | 0.3114 | 0.0504 | 0.356592 | low |
| TCGA-CW-6088 | 0.1189 | 0.4808 | 0.0325 | 0.2938 | 0.2446 | 0.444817 | low |
| TCGA-B8-5552 | 0.1363 | 0.4307 | 0.057 | 0.314 | 0.1058 | 0.447261 | low |
| TCGA-CW-5589 | 0.2291 | 0.5976 | 0.0145 | 0.3661 | 0.0613 | 0.380065 | low |
| TCGA-B0-4833 | 0.4301 | 0.2233 | 0.0131 | 1 | 0.0496 | 1.738004 | high |
| TCGA-CZ-4859 | 0.3336 | 0.7296 | 0.0158 | 0.2756 | 0.0366 | 0.345232 | low |
| TCGA-A3-3320 | 0.3491 | 0.4737 | 0.0078 | 0.2262 | 0.0929 | 0.546473 | low |
| TCGA-MM-A564 | 0.5667 | 0.1957 | 0 | 0.55558 | 0.314 | 2.279389 | high |
| TCGA-AS-3777 | 0.2782 | 0.8111 | 0 | 0.4806 | 0.1223 | 0.344433 | low |
| TCGA-B0-5095 | 0.7163 | 0.1603 | 0.0738 | 0.4637 | 0.0892 | 2.642362 | high |
| TCGA-BP-4795 | 0.3847 | 0.3946 | 0.0945 | 0.4334 | 0.0377 | 0.861782 | high |
| TCGA-BP-4331 | 0.4169 | 0.4437 | 0.065 | 0.4328 | 0.0942 | 0.866222 | high |
| TCGA-A3-A6NJ | 0.1909 | 0.5165 | 0.0813 | 0.3803 | 0.0778 | 0.464705 | low |
| TCGA-B0-4701 | 0.5158 | 0.4572 | 0.0432 | 0.5321 | 0.2678 | 1.346278 | high |
| TCGA-A3-3367 | 0.1498 | 0.4691 | 0.0533 | 0.2647 | 0.126 | 0.423184 | low |
| TCGA-B0-5402 | 0.4052 | 0.5132 | 0.0287 | 0.2292 | 0.0967 | 0.602527 | low |
| TCGA-B0-5691 | 0.205 | 0.4402 | 0.0325 | 0.4024 | 0.0517 | 0.489263 | low |
| TCGA-BP-4342 | 0.7278 | 0.2649 | 0.0595 | 0.3345 | 0.1602 | 2.191494 | high |
| TCGA-A3-3373 | 0.3056 | 0.5492 | 0.0199 | 0.4994 | 0.0766 | 0.552617 | low |
| TCGA-BP-4341 | 0.4695 | 0.1982 | 0.0369 | 0.555 | 0.2757 | 1.886497 | high |
| TCGA-CW-6090 | 0.2751 | 0.3192 | 0.0851 | 0.2964 | 0.0262 | 0.671441 | low |
| TCGA-A3-3307 | 0.17 | 0.5584 | 0.0269 | 0.3447 | 0.0665 | 0.361802 | low |
| TCGA-B2-3924 | 0.0915 | 0.115 | 0.0308 | 0.5778 | 0.2453 | 0.951345 | high |
| TCGA-B0-5706 | 0.2978 | 0.4914 | 0 | 0.2953 | 0.0496 | 0.471958 | low |
| TCGA-BP-4963 | 0.3683 | 0.5509 | 0.1272 | 0.4129 | 0.1308 | 0.757045 | low |
| TCGA-BP-4343 | 0.6999 | 0.1882 | 0.2491 | 0.5749 | 0.0727 | 3.572254 | high |
| TCGA-B0-5703 | 0.6584 | 0.4646 | 0 | 0.4024 | 0.0151 | 1.093252 | high |
| TCGA-CJ-6027 | 0.5764 | 0.5167 | 0.0319 | 0.4214 | 0.0854 | 0.992965 | high |
| TCGA-B0-5108 | 0.6707 | 0.215 | 0.0922 | 0.4713 | 0.059 | 2.203439 | high |
| TCGA-BP-4965 | 0.1653 | 0.5223 | 0.0192 | 0.3043 | 0.1126 | 0.383669 | low |
| TCGA-A3-A6NL | 0.3964 | 0.3166 | 0.0048 | 0.3803 | 0.0922 | 0.874877 | high |
| TCGA-CJ-5672 | 0.2955 | 0.4613 | 0.142 | 0.45 | 0.4338 | 1.155154 | high |
| TCGA-B8-5158 | 0.4842 | 0.6848 | 0.1527 | 0.6008 | 0.0807 | 0.890604 | high |
| TCGA-CJ-4876 | 0.7178 | 0.386 | 0 | 0.6245 | 0.2034 | 2.122308 | high |
| TCGA-B0-5115 | 0.3922 | 0.3219 | 0.0444 | 0.3089 | 0.107 | 0.8864 | high |
| TCGA-CJ-4916 | 0.3322 | 0.5102 | 0.0777 | 0.2563 | 0.1812 | 0.6434 | low |
| TCGA-B0-4848 | 0.4912 | 0.4475 | 0.1073 | 0.3622 | 0.128 | 1.066685 | high |
| TCGA-B4-5377 | 0.5691 | 0.6713 | 0.1151 | 0.2708 | 0.031 | 0.731085 | low |
| TCGA-B0-4712 | 0.65 | 0.1781 | 0.1638 | 0.6047 | 0.1943 | 3.34839 | high |
| TCGA-A3-3324 | 0.3826 | 0.5326 | 0.0462 | 0.4373 | 0.1312 | 0.710184 | low |
| TCGA-CZ-5465 | 0.4061 | 0.6787 | 0.0409 | 0.1939 | 0.1171 | 0.470963 | low |
| TCGA-CZ-4860 | 0.8939 | 0.145 | 0.3076 | 0.4191 | 0.2998 | 7.423991 | high |
| TCGA-B0-5102 | 0.7041 | 0.3558 | 0.0279 | 0.3391 | 0.1148 | 1.617023 | high |
| TCGA-B0-5690 | 0.3103 | 0.3076 | 0.0041 | 0.3866 | 0.0651 | 0.720078 | low |
| TCGA-BP-4160 | 0.3163 | 0.4174 | 0.0651 | 0.4756 | 0.1114 | 0.774919 | low |
| TCGA-B0-4697 | 0.794 | 0.0761 | 0.3437 | 1 | 0.45736 | 13.98254 | high |
| TCGA-AK-3454 | 0.313 | 0.5245 | 0.0115 | 0.3153 | 0.0421 | 0.474719 | low |
| TCGA-B0-5693 | 0.3432 | 0.4308 | 0.0099 | 0.2114 | 0.0555 | 0.54771 | low |
| TCGA-BP-4985 | 0.6827 | 0.0771 | 0.0098 | 0.2925 | 0.2278 | 2.595815 | high |
| TCGA-B0-4718 | 0.6962 | 0.0872 | 0.0598 | 0.6291 | 0.1729 | 3.526536 | high |
| TCGA-B0-5109 | 0.5275 | 0.2259 | 0.1304 | 0.7822 | 0.0167 | 2.100003 | high |
| TCGA-A3-3359 | 0.4375 | 0.3809 | 0.0174 | 0.2723 | 0.0996 | 0.812422 | low |
| TCGA-DV-5569 | 0.2263 | 0.3913 | 0.071 | 0.3854 | 0.1223 | 0.637493 | low |
| TCGA-B2-A4SR | 0.2538 | 0.4657 | 0.0185 | 0.2815 | 0.0908 | 0.482432 | low |
| TCGA-B0-4815 | 0.5569 | 0.2668 | 0.1533 | 0.5449 | 0.542 | 3.432626 | high |
| TCGA-AK-3461 | 0.2229 | 0.3823 | 0.0828 | 0.3501 | 0.059 | 0.589355 | low |
| TCGA-CJ-4892 | 0.5443 | 0.2475 | 0.0134 | 0.553 | 0.1583 | 1.687633 | high |
| TCGA-B0-5695 | 0.1679 | 0.551 | 0.0159 | 0.3328 | 0.0533 | 0.348337 | low |
| TCGA-BP-4975 | 0.3823 | 0.6206 | 0.0211 | 0.1797 | 0.0568 | 0.435973 | low |
| TCGA-AK-3456 | 0.5134 | 0.7138 | 0.0046 | 0.5449 | 0.2088 | 0.777735 | low |
| TCGA-BP-4159 | 0.636 | 0.2658 | 0.0121 | 0.3099 | 0.0675 | 1.452406 | high |
| TCGA-CZ-5458 | 0.4206 | 0.7487 | 0.0704 | 0.2756 | 0.0932 | 0.474181 | low |
| TCGA-AK-3431 | 0.5319 | 0.2407 | 0.0368 | 0.2449 | 0.0675 | 1.206018 | high |
| TCGA-B8-A8YJ | 0.6309 | 0.2438 | 0.0651 | 0.6343 | 0.0819 | 2.16711 | high |
| TCGA-BP-5182 | 0.4133 | 0.4872 | 0.0382 | 0.3781 | 0.0741 | 0.713279 | low |
| TCGA-B2-4101 | 0.2354 | 0.3791 | 0.044 | 0.4566 | 0.3132 | 0.847066 | low |
| TCGA-CJ-5681 | 0.6507 | 0.7874 | 0 | 0.6913 | 0.6667 | 1.808659 | high |
| TCGA-BP-4994 | 0.3519 | 0.8172 | 0.0148 | 0.1818 | 0.2077 | 0.35586 | low |
| TCGA-CJ-4903 | 0.2753 | 0.5961 | 0.0392 | 0.4439 | 0.0316 | 0.450121 | low |
| TCGA-BP-4760 | 0.3921 | 0.5908 | 0.0171 | 0.2656 | 0.0346 | 0.483638 | low |
| TCGA-BP-5190 | 0.5453 | 0.545 | 0.0465 | 0.2906 | 0.1617 | 0.901397 | high |
| TCGA-B0-5710 | 0.2166 | 0.3395 | 0.0024 | 0.3279 | 0.0762 | 0.542117 | low |
| TCGA-CJ-4875 | 0.3369 | 0.2263 | 0.138 | 1 | 0.1557 | 2.0252 | high |
| TCGA-CJ-4874 | 0.5058 | 0.541 | 0.0557 | 0.2745 | 0.0856 | 0.763009 | low |
| TCGA-B2-5641 | 0.3211 | 0.719 | 0.0955 | 0.3553 | 0.0277 | 0.415805 | low |
| TCGA-CW-5584 | 0.4998 | 0.5745 | 0.0616 | 0.3977 | 0.0105 | 0.726898 | low |
| TCGA-B8-4143 | 0.7774 | 0.0999 | 0.356 | 0.5133 | 0.1778 | 6.33447 | high |
| TCGA-B0-5120 | 0.2621 | 0.3712 | 0.0599 | 0.3835 | 0.0876 | 0.665511 | low |
| TCGA-EU-5905 | 0.3984 | 0.5648 | 0.1162 | 0.3431 | 0.0204 | 0.637395 | low |
| TCGA-BP-4338 | 0.8542 | 0.55 | 0.1113 | 0.5343 | 0.2626 | 2.63825 | high |
| TCGA-A3-3358 | 0.2012 | 0.6064 | 0.083 | 0.4402 | 0.064 | 0.425349 | low |
| TCGA-B0-4818 | 0.4066 | 0.479 | 0.0462 | 0.3695 | 0.1355 | 0.77436 | low |
| TCGA-BP-4976 | 0.2437 | 0.6502 | 0.0333 | 0.2681 | 0.0351 | 0.332775 | low |
| TCGA-BP-4345 | 0.6524 | 0.1034 | 0.0468 | 0.3826 | 0.0771 | 2.228222 | high |
| TCGA-CW-6093 | 0.0864 | 0.5086 | 0.0382 | 0.329 | 0.0319 | 0.317782 | low |
| TCGA-BP-4330 | 0.4395 | 0.4481 | 0.0482 | 0.3974 | 0.0692 | 0.824536 | low |
| TCGA-EU-5906 | 0.2026 | 0.4728 | 0.034 | 0.2632 | 0.0464 | 0.410913 | low |
| TCGA-B0-4846 | 0.2044 | 0.5398 | 0.0557 | 0.3062 | 0.0946 | 0.42293 | low |
| TCGA-CJ-6030 | 0.4217 | 0.6903 | 0.1509 | 0.3984 | 0.1563 | 0.719237 | low |
| TCGA-BP-4983 | 0.6873 | 0.164 | 0.2105 | 0.6235 | 0.1067 | 3.665357 | high |
| TCGA-B0-5697 | 0.1175 | 0.623 | 0.149 | 0.4931 | 0.1143 | 0.434229 | low |
| TCGA-BP-4346 | 0.374 | 0.4111 | 0.1607 | 0.5232 | 0.1023 | 1.075069 | high |
| TCGA-B0-4691 | 0.6152 | 0.1298 | 0.1745 | 0.3745 | 0.1223 | 2.602704 | high |
| TCGA-A3-3328 | 0.3532 | 0.8789 | 0.0045 | 0.4202 | 0.1729 | 0.368924 | low |
| TCGA-BP-4765 | 0.1768 | 0.3794 | 0.0391 | 0.386 | 0.0771 | 0.524058 | low |
| TCGA-CZ-4858 | 0.8577 | 0.1883 | 0.13308 | 0.5207 | 0.1114 | 4.056883 | high |
| TCGA-B0-5117 | 0.5022 | 0.7515 | 0.2931 | 0.4994 | 0.1729 | 1.099067 | high |
| TCGA-CZ-4865 | 0.2824 | 0.4951 | 0.0671 | 0.3604 | 0.106 | 0.57837 | low |
| TCGA-CJ-6028 | 0.2586 | 0.4735 | 0.028 | 0.2852 | 0.104 | 0.498913 | low |
| TCGA-6D-AA2E | 0.4984 | 0.5783 | 0.0239 | 0.8677 | 0.4317 | 1.66041 | high |
| TCGA-CJ-4884 | 0.6126 | 0.5161 | 0.1346 | 0.5583 | 0.2263 | 1.710127 | high |
| TCGA-DV-5568 | 0.3924 | 0.2904 | 0.0673 | 0.5994 | 0.1023 | 1.223202 | high |
| TCGA-BP-4961 | 0.3578 | 0.5094 | 0.0171 | 0.2314 | 0.0274 | 0.494758 | low |
| TCGA-BP-4761 | 0.48096 | 0.1111 | 0.2166 | 1 | 0.2179 | 4.086319 | high |
| TCGA-CJ-5677 | 0.5649 | 0.1896 | 0.1173 | 0.767 | 0.2638 | 3.151054 | high |
| TCGA-CZ-5460 | 0.5113 | 0.7023 | 0.0456 | 0.408 | 0.0799 | 0.647288 | low |
| TCGA-CJ-4900 | 0.3252 | 0.2297 | 0.1374 | 0.6995 | 0.2387 | 1.703755 | high |
| TCGA-CZ-5451 | 0.3902 | 0.674 | 0.0192 | 0.3206 | 0.0442 | 0.447542 | low |
| TCGA-B8-A54F | 0.4935 | 0.4696 | 0.0937 | 0.2324 | 0.047 | 0.822189 | low |
| TCGA-B0-4838 | 0.3865 | 0.4639 | 0.1068 | 0.4878 | 0.0983 | 0.89073 | high |
| TCGA-B0-4699 | 0.5602 | 0.4999 | 0.1352 | 0.6167 | 0.2815 | 1.7682 | high |
| TCGA-BP-4781 | 0.5117 | 0.5171 | 0.043 | 0.3315 | 0.0735 | 0.80993 | low |
| TCGA-CJ-4644 | 0.3359 | 0.6057 | 0.047 | 0.371 | 0.1638 | 0.565775 | low |
| TCGA-B0-5701 | 0.4483 | 0.7231 | 0.0083 | 0.4497 | 0.3027 | 0.699676 | low |
| TCGA-B0-5694 | 0.2398 | 0.5766 | 0.0562 | 0.2784 | 0.1033 | 0.424482 | low |
| TCGA-B8-4621 | 0.5517 | 0.7937 | 0.0575 | 0.4112 | 0.203 | 0.724583 | low |
| TCGA-BP-5006 | 0.3503 | 0.4938 | 0.0445 | 0.3602 | 0.0484 | 0.597466 | low |
| TCGA-BP-5181 | 0.4294 | 0.6348 | 0.0176 | 0.1918 | 0.0678 | 0.478289 | low |
| TCGA-B0-5709 | 0.4459 | 0.4549 | 0.1148 | 0.4777 | 0.0328 | 0.949181 | high |
| TCGA-BP-4167 | 0.5904 | 0.1781 | 0.2455 | 0.4439 | 0.2417 | 3.179227 | high |
| TCGA-CJ-4638 | 1 | 0.1839 | 0.0359 | 0.58383 | 0.0651 | 4.601652 | high |
| TCGA-EU-5907 | 0.4449 | 0.7915 | 0.0648 | 0.504 | 0.1454 | 0.592056 | low |
| TCGA-CJ-4869 | 0.4865 | 0.5322 | 0.1709 | 0.3684 | 0.1903 | 1.119693 | high |
| TCGA-3Z-A93Z | 0.2544 | 0.5873 | 0.0235 | 0.395 | 0.0354 | 0.410422 | low |
| TCGA-BP-5196 | 0.6728 | 0.3627 | 0.0641 | 0.5365 | 0.0669 | 1.767726 | high |
| TCGA-AK-3433 | 0.3505 | 0.9325 | 0.0302 | 0.3612 | 0.0822 | 0.300171 | low |
| TCGA-A3-3346 | 0.8141 | 0.3031 | 0.4952 | 0.6305 | 0.5392 | 10.84248 | high |
| TCGA-AK-3440 | 0.415 | 0.7245 | 0.0271 | 0.3264 | 0.0822 | 0.463987 | low |
| TCGA-B8-A54D | 0.4292 | 0.4611 | 0.1399 | 0.8044 | 0.30751 | 1.736879 | high |
| TCGA-BP-5200 | 0.3488 | 0.6058 | 0.0332 | 0.415 | 0.0304 | 0.498528 | low |
| TCGA-B0-5400 | 0.4597 | 0.3328 | 0.0607 | 0.7317 | 0.1114 | 1.463397 | high |
| TCGA-BP-4969 | 0.2275 | 0.2715 | 0.0154 | 0.3279 | 0.3012 | 0.835749 | low |
| TCGA-BP-4756 | 0.6524 | 0.6381 | 0.0013 | 0.6225 | 0.3102 | 1.40894 | high |
| TCGA-BP-4776 | 0.3669 | 0.1264 | 0.1423 | 0.6104 | 0.111 | 1.758981 | high |
| TCGA-CJ-4912 | 0.5865 | 0.196 | 0.0573 | 0.2643 | 0.0946 | 1.582036 | high |
| TCGA-CZ-5985 | 0.3113 | 0.6697 | 0.052 | 0.3745 | 0.0332 | 0.417559 | low |
| TCGA-B0-5098 | 0.5568 | 0.3403 | 0.0482 | 0.8302 | 0.3352 | 2.472896 | high |
| TCGA-B8-5545 | 0.3833 | 0.4716 | 0.0834 | 0.3815 | 0.1114 | 0.781533 | low |
| TCGA-BP-4758 | 0.5713 | 0.2855 | 0.0074 | 0.4257 | 0.1812 | 1.54133 | high |
| TCGA-B0-4696 | 0.3291 | 0.0271 | 0.0148 | 0.6291 | 0.5881 | 2.783449 | high |
| TCGA-A3-3322 | 0.3216 | 0.3313 | 0.0291 | 0.323 | 0.1017 | 0.736972 | low |
| TCGA-B0-5107 | 0.3979 | 0 | 0.1341 | 0.8482 | 0.2118 | 3.114306 | high |
| TCGA-DV-5565 | 0.4983 | 0.4012 | 0.0294 | 0.4675 | 0 | 0.945079 | high |
| TCGA-CZ-5989 | 0 | 0.432 | 0.0078 | 0.1174 | 0.2118 | 0.299003 | low |
| TCGA-BP-4173 | 0.5156 | 0.3781 | 0.1973 | 0.5255 | 0.1763 | 1.784198 | high |
| TCGA-CJ-4870 | 0.3251 | 0.5997 | 0.0044 | 0.2461 | 0.0078 | 0.385983 | low |
| TCGA-BP-4974 | 0.3411 | 0.252 | 0.0493 | 0.392 | 0.1114 | 0.967487 | high |
| TCGA-B0-5075 | 0.476 | 0.6449 | 0.0472 | 0.4341 | 0.2299 | 0.81294 | low |
| TCGA-BP-4162 | 0.4297 | 0.5148 | 0.0335 | 0.4313 | 0.0912 | 0.74673 | low |
| TCGA-B0-4839 | 0.6176 | 0.3145 | 0.0832 | 0.4994 | 0 | 1.573349 | high |
| TCGA-B8-5553 | 0.2701 | 0.7196 | 0.1205 | 0.3504 | 0.0576 | 0.403448 | low |
| TCGA-CJ-4893 | 0.3728 | 0.6112 | 0.0435 | 0.3291 | 0.0635 | 0.514041 | low |
| TCGA-CJ-6033 | 0.5674 | 0.1255 | 0.0186 | 0.4589 | 0.1023 | 1.88034 | high |
| TCGA-B0-4827 | 0.448 | 0.5008 | 0.0435 | 0.385 | 0.458 | 1.223065 | high |
| TCGA-BP-4977 | 0.4234 | 0.6729 | 0.0511 | 0.2927 | 0.103 | 0.534434 | low |
| TCGA-BP-5187 | 0.3327 | 0.4164 | 0.0379 | 0.4866 | 0.1483 | 0.80825 | low |
| TCGA-CJ-4882 | 0.5801 | 0.1832 | 0.296 | 0.9105 | 0.1285 | 4.289096 | high |
| TCGA-CJ-4899 | 0.2582 | 0.4531 | 0.0312 | 0.5165 | 0.1355 | 0.650026 | low |
| TCGA-B8-4620 | 0.7248 | 0.3497 | 0.1399 | 0.2808 | 0.0884 | 1.919487 | high |
| TCGA-CJ-4868 | 0.6088 | 0.2412 | 0.0493 | 0.5333 | 0.1557 | 2.038934 | high |
| TCGA-BP-5001 | 0.2115 | 0.1546 | 0.0298 | 0.5828 | 0.2118 | 1.102669 | high |
| TCGA-DV-5575 | 0.2984 | 0.5339 | 0.0437 | 0.5625 | 0.0619 | 0.6016 | low |
| TCGA-CJ-4920 | 0.4723 | 0.4997 | 0.036 | 0.5781 | 0.1196 | 0.980155 | high |
| TCGA-B0-4707 | 0.8962 | 0.0852 | 0.0505 | 0.7138 | 1 | 15.70292 | high |
| TCGA-AK-3426 | 0.6633 | 0.1861 | 0.4303 | 1 | 0.4963 | 10.90891 | high |
| TCGA-B0-4713 | 0.5825 | 0.3889 | 0.1992 | 0.6662 | 0.1355 | 2.156717 | high |
| TCGA-B0-5705 | 0.2118 | 0.7913 | 0.0215 | 0.2363 | 0.0895 | 0.25321 | low |
| TCGA-BP-5199 | 0.501 | 0.1879 | 0.0923 | 0.5871 | 0.1304 | 1.938622 | high |
| TCGA-CZ-5988 | 0.1484 | 0.4444 | 0 | 0.2407 | 0.0349 | 0.350118 | low |
| TCGA-BP-5004 | 0.3925 | 0.5289 | 0.0076 | 0.3764 | 0.0759 | 0.605463 | low |
| TCGA-A3-3370 | 0.2512 | 0.4391 | 0.081 | 0.3725 | 0.0946 | 0.605242 | low |
| TCGA-B0-5696 | 0.2105 | 0.6536 | 0.0204 | 0.4439 | 0.2244 | 0.439829 | low |
| TCGA-BP-4797 | 0.5924 | 0.547 | 0.0666 | 0.3994 | 0.3093 | 1.346421 | high |
| TCGA-CZ-5982 | 0.1502 | 0.5991 | 0.0347 | 0.1468 | 0.0147 | 0.263301 | low |
| TCGA-BP-4991 | 0.3293 | 0.4343 | 0.0092 | 0.3015 | 0.1114 | 0.609173 | low |
| TCGA-BP-4762 | 0.2789 | 0.3587 | 0.0451 | 0.3441 | 0.0452 | 0.629639 | low |
| TCGA-A3-3362 | 0.4215 | 0.4602 | 0.0054 | 0.5232 | 0.1856 | 0.923224 | high |
| TCGA-A3-A8OW | 0.3998 | 0.4804 | 0 | 0.3519 | 0.1161 | 0.675526 | low |
| TCGA-CJ-4872 | 0.5873 | 0.2249 | 0.0051 | 0.491 | 0.1596 | 1.796619 | high |
| TCGA-B0-4847 | 0.3395 | 0.1514 | 0.2768 | 0.5116 | 0.5733 | 3.306151 | high |
| TCGA-B0-4822 | 0.2982 | 0.2361 | 0.1267 | 0.779 | 0.5552 | 2.465591 | high |
| TCGA-BP-5175 | 0.4028 | 0.1838 | 0.1338 | 0.3745 | 0.25163 | 1.671899 | high |
| TCGA-BP-4340 | 0.4209 | 0.5331 | 0.0141 | 0.4994 | 0.0831 | 0.719741 | low |
| TCGA-BP-4987 | 0.2849 | 0.5297 | 0.0442 | 0.2542 | 0.0829 | 0.470753 | low |
| TCGA-BP-5189 | 0.3662 | 0.5511 | 0.1225 | 0.3328 | 0.0479 | 0.632053 | low |
| TCGA-BP-5000 | 0.2833 | 0.3344 | 0.028 | 0.4439 | 0.1629 | 0.803929 | low |
| TCGA-AK-3451 | 0.4614 | 0.3345 | 0 | 0.43866 | 0.12581 | 1.055024 | high |
| TCGA-BP-5007 | 0.3763 | 0.4394 | 0.0317 | 0.3437 | 0.2295 | 0.830367 | low |
| TCGA-A3-3363 | 0.4158 | 0.7165 | 0.0825 | 0.4585 | 0.2387 | 0.701395 | low |
| TCGA-AK-3455 | 0.1771 | 0.4645 | 0.015 | 0.2958 | 0.1829 | 0.463965 | low |
| TCGA-B8-4146 | 0.0747 | 0.5142 | 0.003 | 0.1916 | 0.1223 | 0.288709 | low |
| TCGA-BP-4770 | 0.7551 | 0.0078 | 0.4451 | 0.8145 | 0.1729 | 10.4268 | high |
| TCGA-A3-3378 | 0.6169 | 0.5535 | 0.1351 | 0.4623 | 0.0989 | 1.28529 | high |
| TCGA-DV-5573 | 0.2386 | 0.5341 | 0.0908 | 0.3473 | 0.2278 | 0.594492 | low |
| TCGA-BP-4962 | 0.5363 | 0.5091 | 0.1563 | 0.3854 | 0.086 | 1.120061 | high |
| TCGA-B8-5546 | 0.4221 | 0.8717 | 0.0063 | 0.4851 | 0.1432 | 0.439326 | low |
| TCGA-BP-4347 | 0.3183 | 0.3787 | 0.0189 | 0.4052 | 0.0356 | 0.656275 | low |
| TCGA-A3-3343 | 0.4661 | 0.4299 | 0.052 | 0.2464 | 0.1114 | 0.841892 | low |
| TCGA-CJ-4918 | 0.3804 | 0.2225 | 0.0134 | 0.4494 | 0.0429 | 0.994962 | high |
| TCGA-BP-5201 | 0.4756 | 0.4579 | 0.024 | 0.4247 | 0.2459 | 1.066072 | high |
| TCGA-CZ-5459 | 0.3075 | 0.5922 | 0.0184 | 0.4479 | 0.1078 | 0.514609 | low |
| TCGA-BP-5170 | 0.1692 | 0.4957 | 0.02 | 0.3447 | 0.0153 | 0.370524 | low |
| TCGA-B0-5094 | 0.8504 | 0.5171 | 0.0453 | 0.5116 | 0.1043 | 1.982576 | high |
| TCGA-BP-4973 | 0.493 | 0.57 | 0.1114 | 0.2897 | 0.0715 | 0.778636 | low |
| TCGA-CJ-5684 | 0.2463 | 0.5206 | 0.0062 | 0.4752 | 0.0137 | 0.452774 | low |
| TCGA-BP-4771 | 0.5382 | 0.3387 | 0.2077 | 0.7548 | 0.154 | 2.37942 | high |
| TCGA-B0-5116 | 0.503 | 0.6 | 0.0243 | 0.2412 | 0.0339 | 0.595846 | low |
| TCGA-CW-5585 | 0.4735 | 0.641 | 0.0192 | 0.2184 | 0.0663 | 0.531053 | low |
| TCGA-CJ-4881 | 0.6522 | 0.1532 | 0.1969 | 0.4391 | 0.2218 | 3.357128 | high |
| TCGA-A3-3323 | 0.461 | 0.3492 | 0.1831 | 0.3891 | 0.0808 | 1.29389 | high |
| TCGA-A3-3380 | 0.4684 | 0.2469 | 0.0546 | 0.7432 | 0.1087 | 1.702932 | high |
| TCGA-B0-4834 | 0.3042 | 0.6527 | 0.0015 | 0.3114 | 0.3513 | 0.543877 | low |
| TCGA-AK-3450 | 0.3433 | 0.6202 | 0.0136 | 0.3745 | 0.0983 | 0.489383 | low |
| TCGA-CZ-5461 | 0.5456 | 0.6838 | 0.1199 | 0.4209 | 0.1023 | 0.848866 | high |
| TCGA-A3-3347 | 0.5518 | 0.2504 | 0.3061 | 0.4913 | 0.1393 | 2.662596 | high |
| TCGA-B0-5110 | 0.2555 | 0.3802 | 0.0268 | 0.3504 | 0.086 | 0.592749 | low |
| TCGA-AK-3460 | 0.3319 | 0.452 | 0.0629 | 0.5116 | 0.0429 | 0.713707 | low |
| TCGA-CJ-4908 | 0.422 | 0.3757 | 0.0605 | 0.418 | 0.1016 | 0.966164 | high |
| TCGA-BP-4774 | 0.4837 | 0.3002 | 0.0251 | 0.4618 | 0.0619 | 1.149939 | high |
| TCGA-B0-4842 | 0.3163 | 0.4241 | 0.0386 | 0.4822 | 0.1729 | 0.793363 | low |
| TCGA-B0-5812 | 0.0658 | 0.4874 | 0.0369 | 0.1943 | 0.0315 | 0.281516 | low |
| TCGA-B0-4816 | 0.5614 | 0.307 | 0.1417 | 0.3298 | 0.088 | 1.525569 | high |
| TCGA-BP-4988 | 0.5186 | 0.2259 | 0.696 | 0.7054 | 0.5704 | 10.44691 | high |
| TCGA-B0-4945 | 0.2368 | 0.5301 | 0.042 | 0.2718 | 0.1323 | 0.457007 | low |
| TCGA-DV-5576 | 0.3128 | 0.509 | 0.0085 | 0.3077 | 0.0524 | 0.487097 | low |
| TCGA-MM-A84U | 0.6652 | 0.2928 | 0.0236 | 0.4645 | 0.2515 | 2.146456 | high |
| TCGA-BP-4998 | 0.4529 | 0.5047 | 0.1337 | 0.4378 | 0.1285 | 1.001453 | high |
| TCGA-A3-3387 | 0.3452 | 0.3261 | 0.1239 | 0.4245 | 0.0702 | 0.964658 | high |
| TCGA-BP-4968 | 0.3868 | 0.2474 | 0.0581 | 0.5449 | 0.1729 | 1.330417 | high |
| TCGA-BP-4995 | 0.219 | 0.7802 | 0.0073 | 0.2852 | 0.1056 | 0.270851 | low |
| TCGA-CJ-4890 | 0.5982 | 0.1766 | 0.1553 | 0.4766 | 0.2005 | 2.69462 | high |
| TCGA-B0-5092 | 0.7162 | 0.1766 | 0.1087 | 0.5092 | 0.3542 | 3.939553 | high |
| TCGA-DV-A4VZ | 0.5528 | 0.2565 | 0 | 0.683 | 0.0724 | 1.652896 | high |
| TCGA-A3-3385 | 0.4648 | 0.5744 | 0.0083 | 0.2607 | 0.0839 | 0.602253 | low |
| TCGA-B0-4823 | 0.1734 | 0.6039 | 0.0451 | 0.2995 | 0.0604 | 0.334603 | low |
| TCGA-BP-4992 | 0.5453 | 0.1263 | 0.6593 | 0.6965 | 0.5733 | 12.10488 | high |
| TCGA-B8-4151 | 0.5453 | 0.672 | 0.0562 | 0.2995 | 0 | 0.616751 | low |
| TCGA-B4-5838 | 0.2183 | 0.3653 | 0.0172 | 0.3745 | 0.2852 | 0.720196 | low |
| TCGA-BP-4986 | 0.6099 | 0.3982 | 0.1483 | 0.3492 | 0.0746 | 1.473641 | high |
| TCGA-CZ-5984 | 0.5419 | 0.6269 | 0.0608 | 0.4719 | 0.1011 | 0.865246 | high |
| TCGA-BP-4344 | 0.2166 | 0.3214 | 0.045 | 0.3437 | 0.0983 | 0.626534 | low |
| TCGA-CZ-4854 | 0.6134 | 0.4484 | 0.0766 | 0.5449 | 0.213 | 1.677173 | high |
| TCGA-CJ-6032 | 0.2963 | 0.6905 | 0.022 | 0.3213 | 0.037 | 0.357073 | low |
| TCGA-BP-4355 | 0.4547 | 0.1849 | 0.1044 | 0.3362 | 0.2387 | 1.684209 | high |
| TCGA-B0-4814 | 0.4353 | 0.478 | 0.0238 | 0.3862 | 0.1158 | 0.782981 | low |
| TCGA-BP-4759 | 0.5252 | 0.4748 | 0.0255 | 0.3228 | 0.0057 | 0.789533 | low |
| TCGA-B0-5097 | 0.5737 | 0.1828 | 0.0841 | 0.3773 | 0.3185 | 2.38426 | high |
| TCGA-CJ-4923 | 0.4408 | 0.2559 | 0.1325 | 0.5039 | 0.3432 | 1.999946 | high |
| TCGA-DV-A4VX | 0.4359 | 0.302 | 0.1787 | 0.5693 | 0.7661 | 3.545188 | high |
| TCGA-CJ-5671 | 0.5432 | 0.3266 | 0.1371 | 0.3923 | 0.0539 | 1.423953 | high |
| TCGA-BP-5191 | 0.3979 | 0.22 | 0.0743 | 0.7218 | 0.2815 | 1.933396 | high |
| TCGA-BP-4158 | 0.4267 | 0.2275 | 0.0135 | 0.3294 | 0.0783 | 1.030257 | high |
| TCGA-BP-4768 | 0.5411 | 0.5415 | 0.0117 | 0.4009 | 0.0262 | 0.781821 | low |
| TCGA-B2-5639 | 0.1832 | 0.7974 | 0.0291 | 0.4169 | 0.3998 | 0.40664 | low |
| TCGA-B0-5084 | 0.3664 | 0.2403 | 0.076 | 0.3745 | 0.8588 | 2.701807 | high |
| TCGA-CJ-5680 | 0.2888 | 0.6143 | 0.1195 | 0.3155 | 0.0727 | 0.491273 | low |
| TCGA-CJ-4639 | 0.2565 | 0.5844 | 0.0472 | 0.3595 | 0.1432 | 0.479329 | low |
| TCGA-B8-5159 | 0.3831 | 0.7191 | 0.0389 | 0.3177 | 0.2202 | 0.526425 | low |
| TCGA-CJ-4641 | 0.5214 | 0.352 | 0.11 | 0.5647 | 0.229 | 1.777655 | high |
| TCGA-B0-5692 | 0.2403 | 0.6719 | 0.0828 | 0.4062 | 0.1114 | 0.428198 | low |
| TCGA-CJ-4895 | 0.6288 | 0.3014 | 0.1203 | 0.3087 | 0.2071 | 1.942366 | high |
| TCGA-BP-4959 | 0.303 | 0.5545 | 0.0925 | 0.2144 | 0.1067 | 0.510188 | low |
| TCGA-BP-4787 | 0.8373 | 0.5538 | 0.2082 | 0.5854 | 0.0788 | 2.497182 | high |
| TCGA-AK-3465 | 0.2698 | 0.8608 | 0.5175 | 0.2933 | 0.1199 | 0.669182 | low |
| TCGA-BP-5009 | 0.6148 | 0.3059 | 0.0635 | 0.514 | 0.1552 | 1.877016 | high |
| TCGA-EU-5904 | 0.332 | 0.6716 | 0.0062 | 0.3356 | 0.0197 | 0.381888 | low |
| TCGA-A3-3306 | 0.0628 | 0.5433 | 0.0699 | 0.2278 | 0.0512 | 0.285428 | low |
| TCGA-BP-5192 | 0.5419 | 0.5873 | 0.0057 | 0.2398 | 0.0227 | 0.62871 | low |
| TCGA-BP-4784 | 0.5197 | 0.397 | 0.0246 | 0.3398 | 0.059 | 0.956383 | high |
| TCGA-B8-4153 | 0.2624 | 0.4146 | 0.1242 | 0.5449 | 0.1114 | 0.817177 | low |
| TCGA-B0-5081 | 0.5456 | 0.288 | 0.1887 | 0.5076 | 0.1783 | 2.13663 | high |
| TCGA-G6-A8L6 | 0.4913 | 0.0683 | 0.2121 | 0.53611 | 0.1303 | 2.731573 | high |
| TCGA-AK-3445 | 0.4652 | 0.2349 | 0.1631 | 0.4298 | 0.2583 | 1.948445 | high |
| TCGA-BP-4351 | 0.6656 | 0.1564 | 0.161 | 0.6734 | 0.4553 | 5.20131 | high |
| TCGA-B0-5100 | 0.4999 | 0.1253 | 0.079 | 0.3584 | 0.2948 | 2.125029 | high |
| TCGA-B0-5700 | 0.1583 | 0.5278 | 0.0168 | 0.5994 | 0.0539 | 0.441225 | low |
| TCGA-AK-3429 | 0.4407 | 0.4801 | 0.047 | 0.555 | 0.0265 | 0.844836 | low |
| TCGA-BP-4798 | 0.8035 | 0.3468 | 0.199 | 0.6291 | 0.2784 | 4.234525 | high |
| TCGA-BP-4769 | 0.6938 | 0.3314 | 0.0084 | 0.2466 | 0.0484 | 1.358894 | high |
| TCGA-CJ-5678 | 0.4717 | 0.4108 | 0.1416 | 0.3897 | 0.0377 | 1.056147 | high |
| TCGA-A3-3316 | 0.2718 | 0.242 | 0.0847 | 0.3745 | 0.2621 | 1.075621 | high |
| TCGA-B8-5550 | 0.5968 | 0.384 | 0.0376 | 0.5022 | 0.2918 | 1.785196 | high |
| TCGA-B0-5699 | 0.2999 | 0.7306 | 0.0214 | 0.2991 | 0.1729 | 0.391314 | low |
| TCGA-CJ-4888 | 0.6012 | 0.3128 | 0.1258 | 0.6148 | 0.394 | 2.936478 | high |
| TCGA-BP-4354 | 0.5658 | 0.1538 | 0.4698 | 0.5096 | 0.1355 | 4.324036 | high |
| TCGA-BP-4989 | 0.4267 | 0.1908 | 0.1496 | 0.7391 | 0.4963 | 3.250787 | high |
| TCGA-CJ-4873 | 0.4158 | 0.1729 | 0.0265 | 0.7054 | 0.4394 | 2.385099 | high |
| TCGA-B0-4824 | 0.1629 | 0.2797 | 0.0594 | 0.5828 | 0.196 | 0.841952 | low |
| TCGA-CZ-5455 | 0.1922 | 0.5435 | 0.0391 | 0.3494 | 0.1062 | 0.418142 | low |
| TCGA-BP-4164 | 0.51 | 0.6647 | 0.0488 | 0.308 | 0.0822 | 0.637652 | low |
| TCGA-CW-5581 | 0.3571 | 0.7107 | 0.0186 | 0.3069 | 0.0314 | 0.382854 | low |
| TCGA-A3-3313 | 0.2453 | 0.3586 | 0.0301 | 0.4762 | 0.3198 | 0.893257 | high |
| TCGA-B0-4845 | 0.3418 | 0.2238 | 0.0155 | 0.7537 | 0.305 | 1.627203 | high |
| TCGA-B4-5378 | 0.4106 | 0.5726 | 0.0058 | 0.2304 | 0.0414 | 0.497192 | low |
| TCGA-BP-5174 | 0.4213 | 0.5293 | 0.0354 | 0.3447 | 0.1272 | 0.700803 | low |
| TCGA-BP-4801 | 0.336 | 0.467 | 0.0373 | 0.2375 | 0.2631 | 0.70515 | low |
| TCGA-CJ-5686 | 0.3058 | 0.5982 | 0.0747 | 0.4003 | 0.8269 | 1.31109 | high |
| TCGA-A3-3349 | 0.4847 | 0.295 | 0.0203 | 0.2686 | 0.0651 | 0.9886 | high |
| TCGA-DV-5567 | 0.4954 | 0.5551 | 0.0042 | 0.2288 | 0.0366 | 0.604266 | low |
| TCGA-CZ-5987 | 0.6066 | 0.5633 | 0.0533 | 0.4112 | 0.0356 | 0.950408 | high |
| TCGA-CJ-4885 | 0.4992 | 0.6381 | 0.0433 | 0.418 | 0.0727 | 0.69647 | low |
| TCGA-DV-5574 | 0.337 | 0.3283 | 0.0307 | 0.4402 | 0.1729 | 0.921026 | high |
| TCGA-A3-3331 | 0.3362 | 0.5293 | 0.0654 | 0.3207 | 0.1837 | 0.650686 | low |
| TCGA-BP-5178 | 0.4305 | 0.3689 | 0.1282 | 0.555 | 0.3006 | 1.601835 | high |
| TCGA-CJ-4871 | 0.5311 | 0.5898 | 0.0487 | 0.3408 | 0.1184 | 0.807146 | low |
| TCGA-B0-4836 | 0.7135 | 0.4676 | 0.0713 | 0.3072 | 0.2387 | 1.689013 | high |
| TCGA-B8-A54I | 0.726 | 0.3811 | 0.2568 | 0.6293 | 0.2171 | 3.501881 | high |
| TCGA-B0-4821 | 0.83 | 0.0892 | 0.2453 | 0.7364 | 0.4045 | 9.374222 | high |
| TCGA-BP-4777 | 0.2344 | 0.3887 | 0.0789 | 0.6804 | 0.1617 | 0.88102 | high |
| TCGA-CJ-4636 | 0.288 | 0.2642 | 0.0953 | 0.491 | 0.2387 | 1.168355 | high |
| TCGA-B0-5711 | 0.1373 | 0.5237 | 0.0318 | 0.1808 | 0.0086 | 0.293739 | low |
| TCGA-CZ-4857 | 0.7551 | 0.2821 | 0.139 | 0.4027 | 0.0491 | 2.394262 | high |
| TCGA-B0-4852 | 0.4397 | 0.4633 | 0.0299 | 0.2189 | 0.1561 | 0.750295 | low |
| TCGA-A3-3329 | 0.3154 | 0.5762 | 0.0851 | 0.3599 | 0.0539 | 0.526241 | low |
| TCGA-CZ-5466 | 0.5538 | 0.51 | 0.0674 | 0.3231 | 0.3578 | 1.317431 | high |
| TCGA-BP-5177 | 0.471 | 0.2567 | 0.0034 | 0.4731 | 0.0277 | 1.118229 | high |
| TCGA-A3-3372 | 0.3952 | 0.374 | 0.0624 | 0.3833 | 0.2088 | 1.020031 | high |
| TCGA-BP-4804 | 0.5505 | 0.2359 | 0.06 | 0.5517 | 0.2052 | 2.00233 | high |
| TCGA-AK-3434 | 0.3058 | 0.1666 | 0.0796 | 0.3416 | 0.1841 | 1.142407 | high |
| TCGA-B0-4811 | 0.676 | 0.069 | 0.0563 | 0.6423 | 0.5403 | 5.50332 | high |
| TCGA-BP-4165 | 0.6999 | 0.2594 | 0 | 0.4637 | 0.1398 | 2.033313 | high |
| TCGA-B0-5099 | 0.4992 | 0.3625 | 0.0133 | 0.2474 | 0.229 | 1.086303 | high |
| TCGA-BP-4766 | 0.4065 | 0.4601 | 0.0127 | 0.428 | 0.0927 | 0.748173 | low |
| TCGA-BP-4335 | 0.6672 | 0.1017 | 0.1326 | 0.6291 | 0.3542 | 4.614396 | high |
| TCGA-B0-4703 | 0.6621 | 0.104 | 0.2289 | 0.4385 | 0.0804 | 3.295765 | high |
| TCGA-CJ-4904 | 0.3087 | 0.6252 | 0.041 | 0.3114 | 0.03 | 0.414002 | low |
| TCGA-BP-4352 | 0.6624 | 0.0217 | 0.5051 | 1 | 0.81 | 23.85232 | high |
| TCGA-CJ-4635 | 0.6046 | 0.4702 | 0.1496 | 0.3416 | 0.1557 | 1.42906 | high |
| TCGA-CJ-5679 | 0.4135 | 0.3448 | 0.0088 | 0.701 | 0.2948 | 1.454193 | high |
| TCGA-CZ-4862 | 0.4029 | 0.5098 | 0.118 | 0.5571 | 0.0989 | 0.924566 | high |
| TCGA-B8-4622 | 0.4729 | 0.5369 | 0.148 | 0.3917 | 0.0836 | 0.926647 | high |
| TCGA-G6-A8L8 | 0.6432 | 0.2739 | 0 | 1 | 0.2034 | 2.95212 | high |
| TCGA-CJ-5682 | 0.1498 | 0.4672 | 0.0441 | 0.3513 | 0 | 0.383557 | low |
| TCGA-BP-4176 | 0.6066 | 0.5328 | 0.0422 | 0.3517 | 0.1151 | 1.028681 | high |
| TCGA-B8-4148 | 0.5081 | 0.4756 | 0.0804 | 0.4036 | 0.1114 | 1.020317 | high |
| TCGA-CJ-4886 | 0.463 | 0.4525 | 0.011 | 0.2813 | 0.0727 | 0.735678 | low |
| TCGA-BP-5186 | 0.161 | 0.4349 | 0.0133 | 0.2553 | 0.1078 | 0.41374 | low |
| TCGA-BP-4972 | 0.3299 | 0.5267 | 0.0521 | 0.3177 | 0.1924 | 0.63511 | low |
| TCGA-BP-4993 | 0.5035 | 0.4348 | 0.0172 | 0.2944 | 0.0838 | 0.8533 | high |
| TCGA-CW-6097 | 0.4311 | 0.2699 | 0.2377 | 0.4904 | 0.1264 | 1.747596 | high |
| TCGA-B0-5077 | 0.5229 | 0.357 | 0.0181 | 0.4836 | 0.179 | 1.323022 | high |
| TCGA-A3-3383 | 0.37695 | 0.191 | 0 | 1 | 0.1242 | 1.755337 | high |
| TCGA-B0-4819 | 0.4838 | 0.2297 | 0.2596 | 0.6705 | 0.5563 | 4.25716 | high |
| TCGA-BP-4775 | 0.4607 | 0.4747 | 0.0168 | 0.3709 | 0.0678 | 0.763066 | low |
| TCGA-BP-4327 | 0.4717 | 0.3884 | 0.0601 | 0.4073 | 0.2082 | 1.186462 | high |
| TCGA-B0-5399 | 0.4207 | 0.4722 | 0.037 | 0.3473 | 0.1596 | 0.802546 | low |
| TCGA-A3-3357 | 0.2179 | 0.7025 | 0.0632 | 0.1996 | 0.2136 | 0.360332 | low |
| TCGA-B0-4694 | 0.4649 | 0.4965 | 0.1773 | 0.2983 | 0.0679 | 0.931222 | high |
| TCGA-A3-3317 | 0.3383 | 0.1737 | 0.0478 | 0.3146 | 0.234 | 1.188635 | high |
| TCGA-B8-4154 | 0.3231 | 0.3213 | 0.02 | 0.3788 | 0.0274 | 0.705798 | low |
| TCGA-B2-5636 | 0.2378 | 0.5332 | 0.0351 | 0.3313 | 0.0176 | 0.410102 | low |
| TCGA-B0-4817 | 0.8605 | 0.2251 | 0.009 | 0.5583 | 0.8907 | 8.32621 | high |
| TCGA-BP-5010 | 0.6624 | 0.1205 | 0.0421 | 0.8211 | 0.1903 | 3.605332 | high |
| TCGA-BP-4325 | 0.3166 | 0.4695 | 0.0276 | 0.3142 | 0.0822 | 0.564451 | low |
| TCGA-BP-5008 | 0.3575 | 0.4101 | 0.0622 | 0.3356 | 0.1148 | 0.761817 | low |
| TCGA-B0-5707 | 0.3917 | 0.769 | 0.0698 | 0.5989 | 0.135 | 0.5911 | low |
| TCGA-CZ-5454 | 0.3545 | 0.4786 | 0.0719 | 0.2214 | 0.023 | 0.561314 | low |
| TCGA-BP-4807 | 0.4809 | 0.518 | 0.0555 | 0.3059 | 0.155 | 0.83978 | low |
| TCGA-A3-3352 | 0.3134 | 0.5372 | 0.0116 | 0.2785 | 0.1378 | 0.508503 | low |
| TCGA-B0-5712 | 0.1888 | 0.7768 | 0.0202 | 0.2407 | 0.1281 | 0.259425 | low |
| TCGA-B0-4843 | 0.8526 | 0.05 | 0.0189 | 0.8073 | 0.79 | 11.94616 | high |
| TCGA-B8-A54H | 0.3629 | 0.4552 | 0.0326 | 0.3059 | 0.1231 | 0.670518 | low |
| TCGA-CZ-5456 | 0.6783 | 0.5263 | 0.13 | 0.5059 | 0.2118 | 1.801453 | high |
| TCGA-B0-4828 | 0.5426 | 0.2752 | 0.0897 | 0.6148 | 0.2733 | 2.23121 | high |
| TCGA-AK-3444 | 0.2844 | 0.5187 | 0.0169 | 0.4518 | 0.1276 | 0.565907 | low |
| TCGA-CJ-6031 | 0.4778 | 0.3035 | 0.0526 | 0.4691 | 0.4099 | 1.833401 | high |
| TCGA-B8-A54K | 0.5108 | 0.4627 | 0 | 0.1395 | 0.0303 | 0.663162 | low |
| TCGA-DV-5566 | 0.2969 | 0.6516 | 0.0112 | 0.3018 | 0.0838 | 0.389379 | low |
| TCGA-B0-5113 | 0.4452 | 0.5286 | 0.1236 | 0.3288 | 0.0383 | 0.7627 | low |
| TCGA-BP-4169 | 0.4273 | 0.2699 | 0.1718 | 0.4219 | 0.0873 | 1.390429 | high |
| TCGA-CZ-5986 | 0.0772 | 0.6933 | 0.0136 | 0.1974 | 0.1231 | 0.222943 | low |
| TCGA-CJ-4640 | 0.5536 | 0.4586 | 0.1209 | 0.392 | 0.197 | 1.363917 | high |
| TCGA-B4-5836 | 0.1825 | 0.4919 | 0.0235 | 0.3684 | 0.0727 | 0.422033 | low |
| TCGA-B0-5713 | 0.1841 | 0.6271 | 0.0045 | 0.314 | 0.0368 | 0.30147 | low |
| TCGA-BP-4790 | 0.4252 | 0.3014 | 0 | 0.2549 | 0.0192 | 0.779068 | low |
| TCGA-BP-4174 | 0.5321 | 0.6161 | 0.0435 | 0.3317 | 0.3719 | 1.0426 | high |
| TCGA-BP-4161 | 0.4086 | 0.4188 | 0.0787 | 0.3566 | 0.1542 | 0.91897 | high |
| TCGA-BP-5183 | 0.2204 | 0.5047 | 0.0651 | 0.5133 | 0.064 | 0.536111 | low |
| TCGA-CW-5588 | 0.3537 | 0.5836 | 0.1891 | 0.456 | 0.0783 | 0.754661 | low |
| TCGA-BP-4970 | 0.1793 | 0.6015 | 0.1403 | 0.3826 | 0.0401 | 0.42002 | low |
| TCGA-A3-3319 | 0.6066 | 0.7723 | 0.1506 | 0.456 | 0.1546 | 0.968969 | high |
| TCGA-BP-4349 | 0.6208 | 0.2713 | 0.0812 | 0.6616 | 0.3552 | 2.992282 | high |
| TCGA-A3-3335 | 0.4978 | 0.6253 | 0.0681 | 0.242 | 0.0961 | 0.660785 | low |
| TCGA-CZ-5464 | 0.4534 | 0.49 | 0.0578 | 0.3085 | 0.1184 | 0.797788 | low |
| TCGA-AK-3436 | 0.5252 | 0.4868 | 0.0588 | 0.3072 | 0.146 | 0.964468 | high |
| TCGA-AK-3427 | 0.2782 | 0.5871 | 0.0598 | 0.2153 | 0.1957 | 0.484537 | low |
| TCGA-BP-4163 | 0.4153 | 0.5221 | 0.0582 | 0.2678 | 0.0822 | 0.647839 | low |
| TCGA-BP-4170 | 0.5155 | 0.5502 | 0.1219 | 0.463 | 0.0217 | 0.929267 | high |
| TCGA-B0-5088 | 0.4781 | 0.2834 | 0.0694 | 0.5232 | 0.2886 | 1.756302 | high |
| TCGA-BP-4329 | 0.3642 | 0.2862 | 0.046 | 0.2678 | 0.1199 | 0.872649 | high |
| TCGA-CW-5591 | 0.2161 | 0.6041 | 0.0201 | 0.3208 | 0.4061 | 0.545295 | low |
| TCGA-CW-5580 | 0.2754 | 0.5311 | 0.0828 | 0.2584 | 0.0398 | 0.46913 | low |
| TCGA-BP-4177 | 0.2572 | 0.6023 | 0.0528 | 0.3976 | 0.0077 | 0.411059 | low |
| TCGA-B0-4837 | 0.7079 | 0.2099 | 0.2468 | 0.3994 | 0.4755 | 4.979157 | high |
| TCGA-CJ-4907 | 0.3918 | 0.4736 | 0.0504 | 0.4135 | 0.0504 | 0.711979 | low |
| TCGA-CJ-4891 | 0.7104 | 0.155 | 0.2051 | 0.7723 | 0.899 | 11.59645 | high |
| TCGA-A3-3365 | 0.1363 | 0.2771 | 0.0204 | 0.6245 | 0.1856 | 0.762327 | low |
| TCGA-BP-4982 | 0.3763 | 0.4826 | 0.0291 | 0.256 | 0.139 | 0.642116 | low |
| TCGA-B8-5549 | 0.2095 | 0.6577 | 0.0335 | 0.3773 | 0.0082 | 0.323752 | low |
| TCGA-MM-A563 | 0.5229 | 0.2523 | 0.0322 | 0.3828 | 0.338 | 1.799726 | high |
| TCGA-B0-4688 | 0.8799 | 0.0226 | 0.6858 | 0.9341 | 0.3495 | 27.7319 | high |
| TCGA-BP-5194 | 0.2327 | 0.4798 | 0.0182 | 0.2512 | 0.0218 | 0.404161 | low |
| TCGA-CJ-4634 | 0.4052 | 0.3107 | 0.0289 | 0.3224 | 0.1132 | 0.919178 | high |
| TCGA-CZ-5462 | 0.5175 | 0.7204 | 0.0533 | 0.3648 | 0.5023 | 1.049802 | high |
| TCGA-CJ-4902 | 0.2942 | 0.2471 | 0.0528 | 0.5449 | 0.1278 | 1.028109 | high |
| TCGA-BP-4981 | 0.549 | 0.1693 | 0.065 | 0.58056 | 0.1876 | 2.245566 | high |
| TCGA-A3-A6NI | 0.5546 | 0.6319 | 0.0254 | 0.4645 | 0.1427 | 0.866154 | high |
| TCGA-BP-5195 | 0.4005 | 0.5391 | 0.1097 | 0.3288 | 0.0287 | 0.658673 | low |
| TCGA-BP-4782 | 0.3999 | 0.5572 | 0.0359 | 0.454 | 0.1693 | 0.73832 | low |
| TCGA-A3-3311 | 0.3309 | 0.6428 | 0.0739 | 0.4032 | 0.1439 | 0.554101 | low |
| TCGA-CZ-4864 | 0.4466 | 0.624 | 0.0655 | 0.2793 | 0.0489 | 0.576033 | low |
| TCGA-B2-4102 | 0.2451 | 0.4881 | 0.1059 | 0.3745 | 0.1644 | 0.630129 | low |
| TCGA-CJ-4878 | 0.5511 | 0.3313 | 0.034 | 0.4731 | 0.196 | 1.523067 | high |
| TCGA-AK-3428 | 0.2178 | 0.7342 | 0 | 0.0624 | 0.0145 | 0.214133 | low |
| TCGA-BP-4971 | 0.4876 | 0.1818 | 0.0596 | 0.5925 | 0.511 | 2.886201 | high |
| TCGA-CJ-5676 | 0.2552 | 0.4449 | 0.0808 | 0.428 | 0.0539 | 0.601354 | low |
| TCGA-B8-A54J | 0.5092 | 0.4379 | 0.0546 | 0.3261 | 0.0606 | 0.915457 | high |
| TCGA-BP-4999 | 0.3539 | 0.3936 | 0.036 | 0.2355 | 0.0607 | 0.63918 | low |
| TCGA-BP-4332 | 0.36 | 0.2395 | 0.0042 | 0.3854 | 0.1472 | 0.985506 | high |
| TCGA-CZ-4863 | 0.3489 | 0.4335 | 0.0583 | 0.4294 | 0.0539 | 0.71645 | low |
| TCGA-A3-3374 | 0.37152 | 0.7988 | 0.4161 | 0.4024 | 0.6007 | 1.510814 | high |
| TCGA-A3-3326 | 0.0176 | 0.3996 | 0.0385 | 0.1977 | 0.22 | 0.37208 | low |
| TCGA-T7-A92I | 0.3978 | 0.8 | 0.0127 | 0.6138 | 0.292 | 0.632391 | low |
| TCGA-AK-3425 | 0.1856 | 0.5017 | 0.0499 | 0.3362 | 0.0255 | 0.402677 | low |
| TCGA-CJ-4894 | 0.375 | 0.4463 | 0.03 | 0.2964 | 0.0675 | 0.643344 | low |
| TCGA-AK-3458 | 0.3912 | 0.3349 | 0 | 0.5608 | 0.0487 | 0.914475 | high |
| TCGA-B0-5106 | 0.6421 | 0.193 | 0.1385 | 0.45 | 0.1432 | 2.545597 | high |
| TCGA-A3-3382 | 0.4391 | 0.4129 | 0.0692 | 0.326 | 0.1449 | 0.937485 | high |
| TCGA-BP-4353 | 0.3644 | 0.2513 | 0.0932 | 0.5904 | 0.1253 | 1.313798 | high |
| TCGA-B0-5698 | 0.2751 | 0.5271 | 0.1484 | 0.3934 | 0.0727 | 0.616016 | low |
| TCGA-CZ-4866 | 0.3062 | 0.5549 | 0.0485 | 0.3319 | 0.2113 | 0.594343 | low |
| TCGA-BP-4799 | 0.8605 | 0.0997 | 0.0168 | 0.4016 | 0.18061 | 3.786278 | high |
| TCGA-CW-5583 | 0.227 | 0.5763 | 0.0068 | 0.2567 | 0.0152 | 0.333874 | low |
| TCGA-B0-5085 | 0.2782 | 0.0836 | 0.0437 | 0.5449 | 0.4629 | 1.924803 | high |
| TCGA-CJ-5683 | 0.3264 | 0.4659 | 0.0695 | 0.3328 | 0.0604 | 0.616805 | low |
| TCGA-CW-5590 | 0.329 | 0.5393 | 0.06 | 0.3745 | 0.0678 | 0.565701 | low |
| TCGA-CZ-5457 | 0.1684 | 0.6119 | 0.0417 | 0.3328 | 0.2296 | 0.411364 | low |
| TCGA-B8-A7U6 | 0.2464 | 0.3242 | 0.0531 | 0.5537 | 0.0724 | 0.773555 | low |
| TCGA-BP-5185 | 0.2629 | 0 | 0.1992 | 0.62982 | 0.111 | 1.949829 | high |
| TCGA-BP-4763 | 0.389 | 0.3443 | 0.0067 | 0.4334 | 0.088 | 0.858703 | high |
| TCGA-BP-5180 | 0.3311 | 0.5506 | 0.0833 | 0.4214 | 0.0375 | 0.581893 | low |
| TCGA-B2-5635 | 0.3675 | 0.463 | 0.0875 | 0.3508 | 0.0796 | 0.724291 | low |
| TCGA-BP-4789 | 0.4492 | 0.2792 | 0.0054 | 0.2383 | 0.0223 | 0.848777 | high |
| TCGA-A3-A8OV | 0.2732 | 0.5856 | 0.0504 | 0.2122 | 0.1722 | 0.45806 | low |
| TCGA-CJ-4643 | 0.3907 | 0.4776 | 0.057 | 0.365 | 0.1355 | 0.762491 | low |
| TCGA-BP-4326 | 0.6326 | 0.3285 | 0.2693 | 0.6563 | 0.0677 | 2.725917 | high |
| TCGA-BP-4960 | 0.4234 | 0.292 | 0.1542 | 0.6221 | 0.1943 | 1.732435 | high |
| TCGA-B0-5121 | 0.4324 | 0.3346 | 0.0334 | 0.3994 | 0.2005 | 1.11887 | high |
| TCGA-B2-4099 | 0.4137 | 0.4101 | 0.0849 | 0.5293 | 0.0854 | 1.006654 | high |
| TCGA-BP-5168 | 0.5487 | 0.4091 | 0.0035 | 0.2852 | 0.0915 | 0.955778 | high |
| TCGA-DV-A4W0 | 0.1257 | 0.3487 | 0.0133 | 0.2514 | 0.3485 | 0.592378 | low |
| TCGA-CJ-4905 | 0.4934 | 0.3349 | 0.0224 | 0.34 | 0.0079 | 0.935818 | high |
| TCGA-CJ-5675 | 0.2 | 0.7062 | 0.0764 | 0.2744 | 0.0551 | 0.30869 | low |
| TCGA-AK-3443 | 0.8293 | 0.9373 | 0.0256 | 0.1936 | 0.1519 | 0.762991 | low |
| TCGA-MW-A4EC | 0.3168 | 0.3746 | 0.049 | 0.3253 | 0.0561 | 0.667618 | low |
| TCGA-A3-3325 | 0.2308 | 0.3859 | 0.0561 | 0.2678 | 0.08 | 0.545344 | low |
| TCGA-CZ-5469 | 0.7662 | 0.1655 | 0.1016 | 0.3994 | 0.1617 | 3.170424 | high |
| TCGA-AK-3453 | 0.3143 | 0.7998 | 0.0081 | 0.4094 | 0.0507 | 0.331464 | low |
| TCGA-CZ-5452 | 0.6138 | 0.5047 | 0.2315 | 0.3841 | 0.1617 | 1.661568 | high |
| TCGA-CJ-4901 | 0.3971 | 0.2576 | 0.2029 | 0.6423 | 0.1783 | 1.883202 | high |
| TCGA-B0-4700 | 0.3966 | 0.1785 | 0.0537 | 0.7409 | 0.3853 | 2.294383 | high |
| TCGA-B2-3923 | 0.4795 | 0.9058 | 0.4963 | 0.319 | 0.2524 | 1.116571 | high |
| TCGA-BP-5184 | 0.1453 | 0.435 | 0.0272 | 0.5628 | 0.0819 | 0.510293 | low |
| TCGA-B0-4810 | 0.3761 | 0.2947 | 0.0479 | 0.7996 | 0.365 | 1.846146 | high |
| TCGA-BP-5176 | 0.3192 | 0.615 | 0.0085 | 0.4691 | 0.0682 | 0.483939 | low |
| TCGA-CZ-5463 | 0.2333 | 0.7127 | 0.0192 | 0.3106 | 0.0379 | 0.298335 | low |
| TCGA-B2-5633 | 0.3437 | 0.5018 | 0.0713 | 0.2483 | 0.1212 | 0.609352 | low |
| TCGA-B8-A54E | 0.1648 | 0.67 | 0.0422 | 0.8559 | 0.8923 | 1.290005 | high |
| TCGA-B0-4844 | 0.7351 | 0.2949 | 0.0727 | 0.594 | 0.1729 | 2.722561 | high |
| TCGA-BP-5169 | 0.4568 | 0.2198 | 0.1674 | 0.8835 | 0.2179 | 2.719449 | high |
| TCGA-B0-4714 | 0.6221 | 0.1925 | 0.0596 | 0.594 | 0.2638 | 2.771911 | high |
| TCGA-CZ-4853 | 0.5658 | 0.6783 | 0.0313 | 0.1808 | 0.126 | 0.646752 | low |

Table S7

| ID | EPC2\|55538\|AT | C4orf19\|69001\|AT | FAM120C\|89237\|AT | BCCIP\|13432\|AT | INPP4B\|70691\|AT | Risk score | risk |
| --- | --- | --- | --- | --- | --- | --- | --- |
| TCGA-CJ-4637 | 0.8688 | 0.5713 | 0.6039 | 0.175 | 0.9043 | 2.583289 | high |
| TCGA-CZ-4861 | 0.7438 | 0.7735 | 0.3774 | 0.2743 | 0.9625 | 1.205772 | high |
| TCGA-BP-4964 | 0.8744 | 0.792 | 0.3097 | 0.2555 | 0.9464 | 0.796937 | low |
| TCGA-CJ-4887 | 0.9122 | 0.7104 | 0.5153 | 0.2401 | 0.9371 | 1.104434 | high |
| TCGA-BP-5198 | 0.8435 | 0.876 | 0.4646 | 0.1819 | 0.9579 | 1.16865 | high |
| TCGA-BP-4967 | 0.8819 | 0.6962 | 0.1865 | 0.326 | 0.9514 | 0.605929 | low |
| TCGA-A3-3351 | 0.9311 | 0.6639 | 0.4279 | 0.2628 | 0.9503 | 0.918922 | high |
| TCGA-B0-4710 | 0.7561 | 0.5957 | 0.4084 | 0.2247 | 0.9343 | 2.218451 | high |
| TCGA-B0-5083 | 0.8828 | 0.8729 | 0.1434 | 0.2895 | 0.9178 | 0.515781 | low |
| TCGA-BP-4803 | 0.8916 | 0.7758 | 0.1763 | 0.2485 | 0.9872 | 0.642914 | low |
| TCGA-CJ-4889 | 0.8921 | 0.8626 | 0.3046 | 0.2766 | 0.9626 | 0.575658 | low |
| TCGA-B0-5080 | 0.8625 | 0.4923 | 0.2962 | 0.1848 | 0.9471 | 1.979828 | high |
| TCGA-CW-5587 | 0.9277 | 0.8867 | 0.176 | 0.2453 | 0.9797 | 0.474431 | low |
| TCGA-A3-3376 | 0.9128 | 0.7253 | 0.2756 | 0.2639 | 0.9614 | 0.722856 | low |
| TCGA-CJ-4897 | 0.9023 | 0.9022 | 0.2337 | 0.289 | 0.9638 | 0.454336 | low |
| TCGA-CW-6088 | 0.9212 | 0.8664 | 0.2109 | 0.3588 | 0.9718 | 0.326229 | low |
| TCGA-B8-5552 | 0.8956 | 0.8569 | 0.2849 | 0.2948 | 0.9815 | 0.501223 | low |
| TCGA-CW-5589 | 0.9263 | 0.8978 | 0.266 | 0.2605 | 0.9654 | 0.492834 | low |
| TCGA-B0-4833 | 0.8487 | 0.5046 | 0.6347 | 0.2244 | 0.8498 | 2.943811 | high |
| TCGA-CZ-4859 | 0.913 | 0.9165 | 0.3046 | 0.3338 | 0.9773 | 0.367138 | low |
| TCGA-A3-3320 | 0.8955 | 0.7248 | 0.4136 | 0.3012 | 0.916 | 0.824875 | high |
| TCGA-MM-A564 | 0.8788 | 0.6672 | 0.4566 | 0.1872 | 0.9097 | 1.680819 | high |
| TCGA-AS-3777 | 0.8831 | 0.9336 | 0.4945 | 0.2946 | 0.8706 | 0.703195 | low |
| TCGA-B0-5095 | 0.897 | 0.3112 | 0.2847 | 0.2377 | 0.9115 | 2.139446 | high |
| TCGA-BP-4795 | 0.8636 | 0.8415 | 0.3009 | 0.2603 | 0.9697 | 0.692634 | low |
| TCGA-BP-4331 | 0.9381 | 0.8283 | 0.601 | 0.241 | 0.9375 | 0.87484 | high |
| TCGA-A3-A6NJ | 0.9198 | 0.8122 | 0.2886 | 0.2241 | 0.9541 | 0.728276 | low |
| TCGA-B0-4701 | 0.8359 | 0.7424 | 0.6106 | 0.1782 | 0.8646 | 2.228751 | high |
| TCGA-A3-3367 | 0.8896 | 0.731 | 0.4208 | 0.3588 | 0.9397 | 0.621288 | low |
| TCGA-B0-5402 | 0.8711 | 0.797 | 0.3172 | 0.2937 | 0.9676 | 0.652012 | low |
| TCGA-B0-5691 | 0.8896 | 0.9072 | 0.1472 | 0.2773 | 0.9915 | 0.42634 | low |
| TCGA-BP-4342 | 0.8368 | 0.6159 | 0.5411 | 0.174 | 0.8947 | 2.531698 | high |
| TCGA-A3-3373 | 0.95 | 0.9299 | 0.1279 | 0.295 | 0.9258 | 0.350585 | low |
| TCGA-BP-4341 | 0.7245 | 0.4655 | 0.5552 | 0.2218 | 0.7357 | 5.678518 | high |
| TCGA-CW-6090 | 0.939 | 0.762 | 0.2853 | 0.2612 | 0.961 | 0.631771 | low |
| TCGA-A3-3307 | 0.9432 | 0.8348 | 0.4228 | 0.268 | 0.967 | 0.594821 | low |
| TCGA-B2-3924 | 0.5402 | 0.3608 | 0.2204 | 0.2786 | 0.6812 | 8.008109 | high |
| TCGA-B0-5706 | 0.9031 | 0.9698 | 0.3526 | 0.2332 | 0.9501 | 0.585175 | low |
| TCGA-BP-4963 | 0.9079 | 0.7968 | 0.3374 | 0.2304 | 0.9658 | 0.777432 | low |
| TCGA-BP-4343 | 0.8797 | 0.9286 | 0.3036 | 0.2371 | 0.9739 | 0.609014 | low |
| TCGA-B0-5703 | 0.9187 | 0.8983 | 0.4199 | 0.3382 | 0.9591 | 0.427313 | low |
| TCGA-CJ-6027 | 0.9227 | 0.3949 | 0.4781 | 0.1639 | 0.9264 | 2.694993 | high |
| TCGA-B0-5108 | 0.8594 | 0.7823 | 0.4197 | 0.209 | 0.8378 | 1.465539 | high |
| TCGA-BP-4965 | 0.9307 | 0.8333 | 0.1866 | 0.308 | 0.9848 | 0.398173 | low |
| TCGA-A3-A6NL | 0.8828 | 0.7602 | 0.4302 | 0.2851 | 0.9327 | 0.843583 | high |
| TCGA-CJ-5672 | 0.8709 | 0.8708 | 0.4823 | 0.2652 | 0.9602 | 0.764936 | low |
| TCGA-B8-5158 | 0.912 | 0.927 | 0.3693 | 0.1537 | 0.9839 | 0.821787 | high |
| TCGA-CJ-4876 | 0.9342 | 0.8089 | 0.4909 | 0.1876 | 0.8948 | 1.135915 | high |
| TCGA-B0-5115 | 0.8829 | 0.514 | 0.4019 | 0.2785 | 0.9257 | 1.38194 | high |
| TCGA-CJ-4916 | 0.9133 | 0.6732 | 0.3819 | 0.1714 | 0.9025 | 1.497633 | high |
| TCGA-B0-4848 | 0.8887 | 0.8335 | 0.346 | 0.2306 | 0.9092 | 0.876575 | high |
| TCGA-B4-5377 | 0.9149 | 0.9026 | 0.456 | 0.2869 | 0.9685 | 0.543189 | low |
| TCGA-B0-4712 | 0.8719 | 0.885 | 0.5655 | 0.1241 | 0.9096 | 1.644752 | high |
| TCGA-A3-3324 | 0.919 | 0.7926 | 0.2003 | 0.2452 | 0.9707 | 0.612402 | low |
| TCGA-CZ-5465 | 0.8195 | 0.8226 | 0.4136 | 0.2443 | 0.979 | 0.974635 | high |
| TCGA-CZ-4860 | 0.2324 | 0.2301 | 0.7408 | 0.2245 | 1 | 30.16262 | high |
| TCGA-B0-5102 | 0.8774 | 0.6888 | 0.3571 | 0.2971 | 0.921 | 0.893225 | high |
| TCGA-B0-5690 | 0.866 | 0.8045 | 0.3149 | 0.2923 | 0.9791 | 0.639835 | low |
| TCGA-BP-4160 | 0.8866 | 0.9111 | 0.2199 | 0.3158 | 0.9152 | 0.458509 | low |
| TCGA-B0-4697 | 0.7816 | 0.2145 | 0.8127 | 0.1237 | 0.8885 | 10.90461 | high |
| TCGA-AK-3454 | 0.7694 | 0.7794 | 0.4115 | 0.2349 | 0.9214 | 1.466851 | high |
| TCGA-B0-5693 | 0.9079 | 0.8915 | 0.3202 | 0.2393 | 0.9457 | 0.638746 | low |
| TCGA-BP-4985 | 0.8955 | 0.2212 | 0.1009 | 0.138 | 0.9646 | 2.919393 | high |
| TCGA-B0-4718 | 0.7156 | 0.4385 | 0.622 | 0.2046 | 0.9126 | 4.855296 | high |
| TCGA-B0-5109 | 0.8103 | 0.823 | 0.666 | 0.1111 | 0.7383 | 3.824415 | high |
| TCGA-A3-3359 | 0.9137 | 0.7652 | 0.1583 | 0.3248 | 0.9323 | 0.486294 | low |
| TCGA-DV-5569 | 0.8769 | 0.8439 | 0.3769 | 0.2682 | 0.9376 | 0.737306 | low |
| TCGA-B2-A4SR | 0.92 | 0.7485 | 0.2945 | 0.3469 | 0.9628 | 0.480305 | low |
| TCGA-B0-4815 | 0.8973 | 0.618 | 0.493 | 0.1892 | 0.884 | 1.891443 | high |
| TCGA-AK-3461 | 0.8569 | 0.593 | 0.6388 | 0.24 | 0.9281 | 1.916858 | high |
| TCGA-CJ-4892 | 0.8637 | 0.7418 | 0.5902 | 0.3002 | 0.8154 | 1.31468 | high |
| TCGA-B0-5695 | 0.8316 | 0.7962 | 0.3335 | 0.3225 | 0.9753 | 0.656298 | low |
| TCGA-BP-4975 | 0.8902 | 0.8154 | 0.2776 | 0.3699 | 0.9751 | 0.403283 | low |
| TCGA-AK-3456 | 0.9402 | 0.8139 | 0.2472 | 0.1724 | 1 | 0.737436 | low |
| TCGA-BP-4159 | 0.8203 | 0.8164 | 0.4281 | 0.2464 | 0.9421 | 1.070525 | high |
| TCGA-CZ-5458 | 0.9349 | 0.8628 | 0.2263 | 0.3129 | 0.9367 | 0.418752 | low |
| TCGA-AK-3431 | 0.8642 | 0.8777 | 0.1559 | 0.2313 | 0.969 | 0.632414 | low |
| TCGA-B8-A8YJ | 0.8238 | 0.7307 | 0.3584 | 0.1745 | 0.9626 | 1.519706 | high |
| TCGA-BP-5182 | 0.9001 | 0.8241 | 0.3663 | 0.2776 | 0.9711 | 0.62849 | low |
| TCGA-B2-4101 | 0.9193 | 0.7151 | 0.4014 | 0.2253 | 0.9509 | 0.987583 | high |
| TCGA-CJ-5681 | 0.973 | 0.805 | 0.4381 | 0.2 | 0.9446 | 0.81561 | low |
| TCGA-BP-4994 | 0.9208 | 0.8965 | 0.1057 | 0.2376 | 0.9031 | 0.540459 | low |
| TCGA-CJ-4903 | 0.8915 | 0.7591 | 0.2445 | 0.2945 | 0.9751 | 0.59905 | low |
| TCGA-BP-4760 | 0.9003 | 0.9242 | 0.1089 | 0.266 | 0.9779 | 0.414345 | low |
| TCGA-BP-5190 | 0.939 | 0.7961 | 0.3753 | 0.1865 | 0.9512 | 0.90986 | high |
| TCGA-B0-5710 | 0.8808 | 0.7672 | 0.3091 | 0.2859 | 0.9813 | 0.666788 | low |
| TCGA-CJ-4875 | 0.8181 | 0.6336 | 0.5172 | 0.2498 | 0.9244 | 1.721018 | high |
| TCGA-CJ-4874 | 0.8845 | 0.9051 | 0.2218 | 0.2922 | 0.9716 | 0.458954 | low |
| TCGA-B2-5641 | 0.9491 | 0.8628 | 0.3507 | 0.335 | 0.9548 | 0.396005 | low |
| TCGA-CW-5584 | 0.8937 | 0.9135 | 0.4542 | 0.2125 | 0.9866 | 0.752946 | low |
| TCGA-B8-4143 | 0.8139 | 0.3512 | 0.7026 | 0.2367 | 0.7482 | 5.605644 | high |
| TCGA-B0-5120 | 0.8801 | 0.7975 | 0.2509 | 0.3025 | 0.9721 | 0.564707 | low |
| TCGA-EU-5905 | 0.9382 | 0.8765 | 0.4541 | 0.2131 | 0.9787 | 0.709894 | low |
| TCGA-BP-4338 | 0.9617 | 0.8361 | 0.1693 | 0.3111 | 0.9646 | 0.362515 | low |
| TCGA-A3-3358 | 0.847 | 0.826 | 0.3416 | 0.1921 | 0.9446 | 1.109396 | high |
| TCGA-B0-4818 | 0.8434 | 0.7498 | 0.301 | 0.269 | 0.9176 | 0.952365 | high |
| TCGA-BP-4976 | 0.9346 | 0.914 | 0.1832 | 0.3367 | 0.9761 | 0.301672 | low |
| TCGA-BP-4345 | 0.8077 | 0.4911 | 0.34 | 0.2368 | 0.9286 | 2.062997 | high |
| TCGA-CW-6093 | 0.8966 | 0.8253 | 0.145 | 0.2769 | 0.9757 | 0.505239 | low |
| TCGA-BP-4330 | 0.8651 | 0.7525 | 0.3811 | 0.2935 | 0.9405 | 0.819492 | high |
| TCGA-EU-5906 | 0.9118 | 0.8714 | 0.1881 | 0.3099 | 0.9694 | 0.403709 | low |
| TCGA-B0-4846 | 0.8276 | 0.797 | 0.2803 | 0.2096 | 0.9036 | 1.191281 | high |
| TCGA-CJ-6030 | 0.9149 | 0.832 | 0.2412 | 0.2073 | 0.9569 | 0.72559 | low |
| TCGA-BP-4983 | 0.8787 | 0.3458 | 0.559 | 0.0898 | 0.8847 | 5.573482 | high |
| TCGA-B0-5697 | 0.9039 | 0.879 | 0.194 | 0.1976 | 0.9294 | 0.724042 | low |
| TCGA-BP-4346 | 0.877 | 0.7245 | 0.5313 | 0.2321 | 0.9047 | 1.358313 | high |
| TCGA-B0-4691 | 0.8306 | 0.2019 | 0.4848 | 0.2125 | 0.8618 | 4.969971 | high |
| TCGA-A3-3328 | 0.9084 | 0.8967 | 0.0838 | 0.3402 | 0.857 | 0.391174 | low |
| TCGA-BP-4765 | 0.8827 | 0.8006 | 0.2984 | 0.2857 | 0.917 | 0.70563 | low |
| TCGA-CZ-4858 | 0.942 | 0.3524 | 0.4171 | 0.0927 | 0.9213 | 3.549043 | high |
| TCGA-B0-5117 | 0.9217 | 0.8101 | 0.2232 | 0.1239 | 0.8477 | 1.314045 | high |
| TCGA-CZ-4865 | 0.8805 | 0.886 | 0.1878 | 0.3147 | 0.9405 | 0.452723 | low |
| TCGA-CJ-6028 | 0.9235 | 0.7035 | 0.1947 | 0.2184 | 0.9665 | 0.807847 | low |
| TCGA-6D-AA2E | 0.9414 | 0.82 | 0.4433 | 0.2048 | 0.9147 | 0.921813 | high |
| TCGA-CJ-4884 | 0.8984 | 0.6852 | 0.4243 | 0.2311 | 0.9092 | 1.222563 | high |
| TCGA-DV-5568 | 0.9013 | 0.7321 | 0.4228 | 0.1817 | 0.9571 | 1.231467 | high |
| TCGA-BP-4961 | 0.9194 | 0.8259 | 0.2713 | 0.2812 | 0.9665 | 0.531995 | low |
| TCGA-BP-4761 | 0.8811 | 0.7682 | 0.8549 | 0.1713 | 0.8569 | 2.443503 | high |
| TCGA-CJ-5677 | 0.8887 | 0.5405 | 0.5227 | 0.2575 | 0.9556 | 1.491119 | high |
| TCGA-CZ-5460 | 0.856 | 0.8385 | 0.5673 | 0.2102 | 0.9267 | 1.265837 | high |
| TCGA-CJ-4900 | 0.8828 | 0.6891 | 0.4719 | 0.1312 | 0.9127 | 2.039476 | high |
| TCGA-CZ-5451 | 0.9382 | 0.8267 | 0.2802 | 0.3293 | 0.9703 | 0.406751 | low |
| TCGA-B8-A54F | 0.85 | 0.7351 | 0.4456 | 0.3424 | 0.9217 | 0.801503 | low |
| TCGA-B0-4838 | 0.8568 | 0.7579 | 0.3637 | 0.2011 | 0.9335 | 1.235504 | high |
| TCGA-B0-4699 | 0.8175 | 0.8385 | 0.4722 | 0.1834 | 0.7804 | 2.002616 | high |
| TCGA-BP-4781 | 0.8923 | 0.7772 | 0.1483 | 0.3268 | 0.9691 | 0.462181 | low |
| TCGA-CJ-4644 | 0.8747 | 0.9432 | 0.47 | 0.198 | 0.9863 | 0.817912 | low |
| TCGA-B0-5701 | 0.9357 | 0.7549 | 0.3596 | 0.1785 | 0.9123 | 1.103319 | high |
| TCGA-B0-5694 | 0.9026 | 0.8269 | 0.1962 | 0.2286 | 0.9645 | 0.654931 | low |
| TCGA-B8-4621 | 0.9046 | 0.8807 | 0.2563 | 0.2737 | 0.9265 | 0.556191 | low |
| TCGA-BP-5006 | 0.8915 | 0.8329 | 0.3224 | 0.2689 | 0.9449 | 0.667721 | low |
| TCGA-BP-5181 | 0.8958 | 0.865 | 0.4347 | 0.336 | 0.958 | 0.504451 | low |
| TCGA-B0-5709 | 0.9151 | 0.837 | 0.2401 | 0.2395 | 0.9802 | 0.594391 | low |
| TCGA-BP-4167 | 0.8583 | 0.7377 | 0.6029 | 0.2187 | 0.9228 | 1.539774 | high |
| TCGA-CJ-4638 | 0.9134 | 0.2825 | 0.4181 | 0.1029 | 0.7412 | 6.286717 | high |
| TCGA-EU-5907 | 0.8979 | 0.8969 | 0.4271 | 0.3326 | 0.9453 | 0.487246 | low |
| TCGA-CJ-4869 | 0.8816 | 0.8599 | 0.406 | 0.1999 | 0.965 | 0.915907 | high |
| TCGA-3Z-A93Z | 0.9055 | 0.8124 | 0.5123 | 0.2216 | 0.9376 | 0.998099 | high |
| TCGA-BP-5196 | 0.8539 | 0.5465 | 0.2396 | 0.1837 | 0.9646 | 1.676164 | high |
| TCGA-AK-3433 | 0.9073 | 0.8456 | 0.1703 | 0.2863 | 0.9219 | 0.518359 | low |
| TCGA-A3-3346 | 0.7226 | 0.8027 | 0.7123 | 0.1787 | 0.8207 | 3.47865 | high |
| TCGA-AK-3440 | 0.922 | 0.9018 | 0.16 | 0.3047 | 0.9438 | 0.386603 | low |
| TCGA-B8-A54D | 0.8509 | 0.7734 | 0.5533 | 0.1942 | 0.8555 | 1.796768 | high |
| TCGA-BP-5200 | 0.9102 | 0.8951 | 0.2083 | 0.2888 | 0.9705 | 0.431861 | low |
| TCGA-B0-5400 | 0.8559 | 0.8143 | 0.5266 | 0.1367 | 0.8901 | 1.889211 | high |
| TCGA-BP-4969 | 0.868 | 0.6937 | 0.4159 | 0.2948 | 0.7955 | 1.277844 | high |
| TCGA-BP-4756 | 0.9548 | 0.736 | 0.3656 | 0.288 | 0.9147 | 0.672974 | low |
| TCGA-BP-4776 | 0.8158 | 0.624 | 0.5646 | 0.1942 | 0.8512 | 2.749546 | high |
| TCGA-CJ-4912 | 0.9037 | 0.6588 | 0.4236 | 0.2144 | 0.8617 | 1.503183 | high |
| TCGA-CZ-5985 | 0.9365 | 0.9068 | 0.1941 | 0.2529 | 0.9617 | 0.454086 | low |
| TCGA-B0-5098 | 0.8064 | 0.8401 | 0.673 | 0.043 | 0.7386 | 5.049633 | high |
| TCGA-B8-5545 | 0.9277 | 0.9157 | 0.2525 | 0.2771 | 0.9845 | 0.417911 | low |
| TCGA-BP-4758 | 0.8687 | 0.741 | 0.454 | 0.3034 | 0.8695 | 0.993668 | high |
| TCGA-B0-4696 | 0.9105 | 0.12 | 0.1905 | 0.2389 | 0.9109 | 2.693013 | high |
| TCGA-A3-3322 | 0.945 | 0.6315 | 0.279 | 0.2164 | 0.9782 | 0.926918 | high |
| TCGA-B0-5107 | 0.8912 | 0.0552 | 0.4338 | 0.1185 | 0.8267 | 8.320062 | high |
| TCGA-DV-5565 | 0.8208 | 0.8709 | 0.3841 | 0.258 | 0.9658 | 0.832112 | high |
| TCGA-CZ-5989 | 0.9035 | 0.9439 | 0.0228 | 0.2736 | 0.9426 | 0.378071 | low |
| TCGA-BP-4173 | 0.8301 | 0.6212 | 0.3643 | 0.2281 | 0.9206 | 1.61085 | high |
| TCGA-CJ-4870 | 0.8596 | 0.8508 | 0.404 | 0.2735 | 0.9612 | 0.734917 | low |
| TCGA-BP-4974 | 0.833 | 0.5408 | 0.3115 | 0.1889 | 0.9181 | 2.105514 | high |
| TCGA-B0-5075 | 0.9203 | 0.8117 | 0.4675 | 0.2225 | 0.9483 | 0.886755 | high |
| TCGA-BP-4162 | 0.865 | 0.7763 | 0.3249 | 0.2459 | 0.925 | 0.938298 | high |
| TCGA-B0-4839 | 0.8429 | 0.7753 | 0.3692 | 0.2136 | 0.958 | 1.130199 | high |
| TCGA-B8-5553 | 0.9022 | 0.8951 | 0.18 | 0.299 | 0.9783 | 0.40567 | low |
| TCGA-CJ-4893 | 0.8793 | 0.88 | 0.2953 | 0.2656 | 0.9459 | 0.624562 | low |
| TCGA-CJ-6033 | 0.8722 | 0.8004 | 0.3709 | 0.1262 | 0.9491 | 1.45311 | high |
| TCGA-B0-4827 | 0.9125 | 0.8257 | 0.3959 | 0.1687 | 0.9629 | 1.006224 | high |
| TCGA-BP-4977 | 0.8826 | 0.8168 | 0.336 | 0.3587 | 0.9104 | 0.526558 | low |
| TCGA-BP-5187 | 0.9242 | 0.7925 | 0.2808 | 0.1573 | 0.9573 | 0.979041 | high |
| TCGA-CJ-4882 | 0.8091 | 0.497 | 0.5057 | 0.2351 | 0.822 | 3.030152 | high |
| TCGA-CJ-4899 | 0.9258 | 0.8215 | 0.3718 | 0.2565 | 0.8941 | 0.753957 | low |
| TCGA-B8-4620 | 0.749 | 0.4018 | 0.4643 | 0.134 | 0.9217 | 5.305743 | high |
| TCGA-CJ-4868 | 0.8997 | 0.6448 | 0.3856 | 0.2641 | 0.9226 | 1.068754 | high |
| TCGA-BP-5001 | 0.8981 | 0.5749 | 0.568 | 0.2179 | 0.9007 | 1.885987 | high |
| TCGA-DV-5575 | 0.8721 | 0.8531 | 0.3137 | 0.2803 | 0.9491 | 0.639874 | low |
| TCGA-CJ-4920 | 0.8786 | 0.7578 | 0.3302 | 0.219 | 0.9428 | 1.010599 | high |
| TCGA-B0-4707 | 0.8411 | 0.6169 | 0.758 | 0.1538 | 0.9257 | 3.15627 | high |
| TCGA-AK-3426 | 0.7656 | 0.5773 | 0.7533 | 0.1438 | 0.884 | 4.95286 | high |
| TCGA-B0-4713 | 0.8828 | 0.7287 | 0.4984 | 0.1964 | 0.795 | 1.885574 | high |
| TCGA-B0-5705 | 0.9522 | 0.9114 | 0.169 | 0.318 | 0.9558 | 0.319424 | low |
| TCGA-BP-5199 | 0.8875 | 0.311 | 0.4573 | 0.2441 | 0.9414 | 2.392825 | high |
| TCGA-CZ-5988 | 0.9103 | 0.7293 | 0.2214 | 0.3293 | 0.9642 | 0.51442 | low |
| TCGA-BP-5004 | 0.9204 | 0.8364 | 0.2181 | 0.2544 | 0.9435 | 0.580628 | low |
| TCGA-A3-3370 | 0.9041 | 0.759 | 0.2783 | 0.3042 | 0.961 | 0.587988 | low |
| TCGA-B0-5696 | 0.9023 | 0.8897 | 0.2988 | 0.2552 | 0.9721 | 0.56411 | low |
| TCGA-BP-4797 | 0.9008 | 0.8896 | 0.1192 | 0.2017 | 0.9615 | 0.610045 | low |
| TCGA-CZ-5982 | 0.8988 | 0.8874 | 0.2596 | 0.3558 | 0.9716 | 0.358385 | low |
| TCGA-BP-4991 | 0.9168 | 0.6432 | 0.2981 | 0.3315 | 0.9757 | 0.621225 | low |
| TCGA-BP-4762 | 0.8754 | 0.871 | 0.2304 | 0.281 | 0.9738 | 0.532105 | low |
| TCGA-A3-3362 | 0.9012 | 0.8202 | 0.4089 | 0.275 | 0.949 | 0.697992 | low |
| TCGA-A3-A8OW | 0.9455 | 0.8416 | 0.3672 | 0.2844 | 0.9366 | 0.548303 | low |
| TCGA-CJ-4872 | 0.8371 | 0.7041 | 0.5664 | 0.1702 | 0.9396 | 2.017422 | high |
| TCGA-B0-4847 | 0.8035 | 0.6609 | 0.7114 | 0.1588 | 0.868 | 3.462392 | high |
| TCGA-B0-4822 | 0.8294 | 0.479 | 0.7545 | 0.1991 | 0.8324 | 4.295404 | high |
| TCGA-BP-5175 | 0.8915 | 0.6485 | 0.5126 | 0.1908 | 0.8718 | 1.888344 | high |
| TCGA-BP-4340 | 0.8753 | 0.7226 | 0.3093 | 0.3759 | 0.8681 | 0.64129 | low |
| TCGA-BP-4987 | 0.9118 | 0.7274 | 0.2649 | 0.326 | 0.9655 | 0.542706 | low |
| TCGA-BP-5189 | 0.9032 | 0.7753 | 0.2351 | 0.2238 | 0.9634 | 0.76865 | low |
| TCGA-BP-5000 | 0.8839 | 0.6193 | 0.5169 | 0.2318 | 0.9405 | 1.489871 | high |
| TCGA-AK-3451 | 0.951 | 0.849 | 0.3691 | 0.1714 | 0.9479 | 0.84298 | high |
| TCGA-BP-5007 | 0.9086 | 0.7243 | 0.35 | 0.3179 | 0.9515 | 0.64068 | low |
| TCGA-A3-3363 | 0.9281 | 0.8854 | 0.3337 | 0.2742 | 0.9596 | 0.512615 | low |
| TCGA-AK-3455 | 0.8766 | 0.8104 | 0.5 | 0.2406 | 0.9329 | 1.012575 | high |
| TCGA-B8-4146 | 0.8408 | 0.946 | 0.0659 | 0.3791 | 0.9251 | 0.318377 | low |
| TCGA-BP-4770 | 0.6525 | 0.2866 | 0.5676 | 0.1898 | 0.8553 | 9.143746 | high |
| TCGA-A3-3378 | 0.878 | 0.7037 | 0.3724 | 0.2058 | 0.9509 | 1.220255 | high |
| TCGA-DV-5573 | 0.9232 | 0.8429 | 0.4687 | 0.2762 | 0.9526 | 0.651372 | low |
| TCGA-BP-4962 | 0.8761 | 0.8678 | 0.3529 | 0.1821 | 0.9486 | 0.973609 | high |
| TCGA-B8-5546 | 0.9462 | 0.8918 | 0.2117 | 0.2219 | 0.9489 | 0.541173 | low |
| TCGA-BP-4347 | 0.8977 | 0.7196 | 0.3863 | 0.259 | 0.9489 | 0.899282 | high |
| TCGA-A3-3343 | 0.9186 | 0.7927 | 0.2345 | 0.3033 | 0.9622 | 0.503479 | low |
| TCGA-CJ-4918 | 0.9083 | 0.7261 | 0.2676 | 0.2258 | 0.9618 | 0.854852 | high |
| TCGA-BP-5201 | 0.928 | 0.6808 | 0.4006 | 0.2424 | 0.9499 | 0.954892 | high |
| TCGA-CZ-5459 | 0.8841 | 0.889 | 0.5216 | 0.2741 | 0.9265 | 0.760863 | low |
| TCGA-BP-5170 | 0.8696 | 0.8199 | 0.2051 | 0.1808 | 0.9582 | 0.928074 | high |
| TCGA-B0-5094 | 0.8886 | 0.7858 | 0.3984 | 0.2624 | 0.9455 | 0.818325 | low |
| TCGA-BP-4973 | 0.8824 | 0.6972 | 0.2549 | 0.3162 | 0.92 | 0.720949 | low |
| TCGA-CJ-5684 | 0.9337 | 0.8721 | 0.3949 | 0.267 | 0.9617 | 0.563696 | low |
| TCGA-BP-4771 | 0.8745 | 0.4153 | 0.4672 | 0.2181 | 0.9358 | 2.328865 | high |
| TCGA-B0-5116 | 0.9334 | 0.7972 | 0.2355 | 0.2919 | 0.9418 | 0.522219 | low |
| TCGA-CW-5585 | 0.944 | 0.8702 | 0.3395 | 0.3082 | 0.9477 | 0.447041 | low |
| TCGA-CJ-4881 | 0.8293 | 0.568 | 0.5666 | 0.1526 | 0.8981 | 3.17857 | high |
| TCGA-A3-3323 | 0.9164 | 0.6445 | 0.2162 | 0.3423 | 0.9621 | 0.56207 | low |
| TCGA-A3-3380 | 0.9247 | 0.7071 | 0.4033 | 0.2428 | 0.9239 | 0.970499 | high |
| TCGA-B0-4834 | 0.9063 | 0.8933 | 0.1979 | 0.1564 | 0.9602 | 0.783625 | low |
| TCGA-AK-3450 | 0.9186 | 0.8531 | 0.374 | 0.1763 | 0.9218 | 0.967096 | high |
| TCGA-CZ-5461 | 0.8857 | 0.8779 | 0.442 | 0.285 | 0.9785 | 0.610019 | low |
| TCGA-A3-3347 | 0.8649 | 0.4432 | 0.3744 | 0.1917 | 0.948 | 2.263883 | high |
| TCGA-B0-5110 | 0.9003 | 0.8112 | 0.2426 | 0.2797 | 0.9339 | 0.610986 | low |
| TCGA-AK-3460 | 0.9269 | 0.7677 | 0.33 | 0.2389 | 0.9105 | 0.833085 | high |
| TCGA-CJ-4908 | 0.9066 | 0.7108 | 0.265 | 0.2487 | 0.9324 | 0.852615 | high |
| TCGA-BP-4774 | 0.892 | 0.7015 | 0.468 | 0.2233 | 0.9498 | 1.197518 | high |
| TCGA-B0-4842 | 0.8267 | 0.7038 | 0.5497 | 0.0431 | 0.9717 | 3.30749 | high |
| TCGA-B0-5812 | 0.9045 | 0.8512 | 0.2418 | 0.3019 | 0.958 | 0.481063 | low |
| TCGA-B0-4816 | 0.8841 | 0.7937 | 0.3258 | 0.2259 | 0.8988 | 0.983096 | high |
| TCGA-BP-4988 | 0.8233 | 0.29 | 0.6572 | 0.2052 | 0.8047 | 5.93668 | high |
| TCGA-B0-4945 | 0.9329 | 0.7119 | 0.2027 | 0.2525 | 0.9089 | 0.759249 | low |
| TCGA-DV-5576 | 0.9191 | 0.8429 | 0.1862 | 0.2546 | 0.9521 | 0.547116 | low |
| TCGA-MM-A84U | 0.8268 | 0.6946 | 0.4658 | 0.1337 | 0.8799 | 2.554683 | high |
| TCGA-BP-4998 | 0.88 | 0.6556 | 0.2402 | 0.215 | 0.9443 | 1.137699 | high |
| TCGA-A3-3387 | 0.9125 | 0.7512 | 0.3137 | 0.2031 | 0.9734 | 0.904076 | high |
| TCGA-BP-4968 | 0.8777 | 0.7051 | 0.3975 | 0.2979 | 0.9258 | 0.887773 | high |
| TCGA-BP-4995 | 0.9176 | 0.8954 | 0.1483 | 0.2882 | 0.9063 | 0.456478 | low |
| TCGA-CJ-4890 | 0.8971 | 0.8571 | 0.2959 | 0.2291 | 0.9088 | 0.780634 | low |
| TCGA-B0-5092 | 0.838 | 0.4938 | 0.5144 | 0.1333 | 0.8292 | 4.265532 | high |
| TCGA-DV-A4VZ | 0.8183 | 0.7511 | 0.5632 | 0.196 | 0.8557 | 2.090388 | high |
| TCGA-A3-3385 | 0.9388 | 0.7526 | 0.2945 | 0.3119 | 0.9748 | 0.507563 | low |
| TCGA-B0-4823 | 0.9206 | 0.8857 | 0.2829 | 0.2887 | 0.9692 | 0.45945 | low |
| TCGA-BP-4992 | 0.7014 | 0.3043 | 0.7842 | 0.1116 | 0.7708 | 15.66323 | high |
| TCGA-B8-4151 | 0.8949 | 0.9078 | 0.5076 | 0.2961 | 0.9214 | 0.64244 | low |
| TCGA-B4-5838 | 0.9176 | 0.8038 | 0.1997 | 0.1797 | 0.9694 | 0.798968 | low |
| TCGA-BP-4986 | 0.9247 | 0.6227 | 0.2121 | 0.2601 | 0.9693 | 0.796382 | low |
| TCGA-CZ-5984 | 0.9189 | 0.845 | 0.2113 | 0.2803 | 0.9806 | 0.471076 | low |
| TCGA-BP-4344 | 0.9053 | 0.7038 | 0.2576 | 0.2255 | 0.9408 | 0.934799 | high |
| TCGA-CZ-4854 | 0.9252 | 0.5954 | 0.2825 | 0.2897 | 0.929 | 0.863463 | high |
| TCGA-CJ-6032 | 0.8811 | 0.9246 | 0.2203 | 0.2516 | 0.9713 | 0.531346 | low |
| TCGA-BP-4355 | 0.8904 | 0.4924 | 0.5485 | 0.2655 | 0.8547 | 2.003327 | high |
| TCGA-B0-4814 | 0.8948 | 0.7113 | 0.6094 | 0.2475 | 0.8924 | 1.366118 | high |
| TCGA-BP-4759 | 0.894 | 0.7874 | 0.3202 | 0.2946 | 0.9703 | 0.612429 | low |
| TCGA-B0-5097 | 0.8752 | 0.9342 | 0.4209 | 0.1777 | 0.9447 | 0.943679 | high |
| TCGA-CJ-4923 | 0.9017 | 0.6282 | 0.5323 | 0.1587 | 0.9246 | 1.986325 | high |
| TCGA-DV-A4VX | 0.7872 | 0.775 | 0.6547 | 0.1848 | 0.813 | 2.781068 | high |
| TCGA-CJ-5671 | 0.8962 | 0.7241 | 0.3367 | 0.2085 | 0.9503 | 1.055675 | high |
| TCGA-BP-5191 | 0.859 | 0.7101 | 0.6224 | 0.1878 | 0.9122 | 1.930329 | high |
| TCGA-BP-4158 | 0.8878 | 0.6306 | 0.3453 | 0.2745 | 0.9209 | 1.053134 | high |
| TCGA-BP-4768 | 0.9242 | 0.5558 | 0.3129 | 0.2759 | 0.9666 | 0.944134 | high |
| TCGA-B2-5639 | 0.9399 | 0.9525 | 0.2505 | 0.2685 | 0.9525 | 0.414591 | low |
| TCGA-B0-5084 | 0.8051 | 0.7467 | 0.5824 | 0.2017 | 0.8734 | 2.108022 | high |
| TCGA-CJ-5680 | 0.9234 | 0.7946 | 0.3414 | 0.2038 | 0.975 | 0.819054 | high |
| TCGA-CJ-4639 | 0.8995 | 0.8023 | 0.298 | 0.3354 | 0.9664 | 0.483829 | low |
| TCGA-B8-5159 | 0.9321 | 0.8889 | 0.1153 | 0.2775 | 0.9847 | 0.377732 | low |
| TCGA-CJ-4641 | 0.8605 | 0.7153 | 0.3343 | 0.1794 | 0.9199 | 1.455215 | high |
| TCGA-B0-5692 | 0.8973 | 0.8996 | 0.2686 | 0.3093 | 0.9675 | 0.437019 | low |
| TCGA-CJ-4895 | 0.9082 | 0.6542 | 0.308 | 0.1784 | 0.9082 | 1.407197 | high |
| TCGA-BP-4959 | 0.9273 | 0.8672 | 0.2433 | 0.2459 | 0.9405 | 0.572325 | low |
| TCGA-BP-4787 | 0.848 | 0.8788 | 0.3471 | 0.2367 | 0.9429 | 0.831662 | high |
| TCGA-AK-3465 | 0.9283 | 0.8909 | 0.2627 | 0.2445 | 0.8557 | 0.6699 | low |
| TCGA-BP-5009 | 0.9077 | 0.7021 | 0.33 | 0.209 | 0.9411 | 1.072874 | high |
| TCGA-EU-5904 | 0.8566 | 0.8233 | 0.2823 | 0.2978 | 0.9056 | 0.703783 | low |
| TCGA-A3-3306 | 0.926 | 0.9382 | 0.4232 | 0.2487 | 0.9211 | 0.616851 | low |
| TCGA-BP-5192 | 0.9091 | 0.8311 | 0.1982 | 0.3481 | 0.9818 | 0.367803 | low |
| TCGA-BP-4784 | 0.8948 | 0.9018 | 0.1919 | 0.2758 | 0.9491 | 0.488083 | low |
| TCGA-B8-4153 | 0.7754 | 0.7633 | 0.5748 | 0.2505 | 0.964 | 1.491132 | high |
| TCGA-B0-5081 | 0.8233 | 0.5865 | 0.5256 | 0.1892 | 0.9058 | 2.523588 | high |
| TCGA-G6-A8L6 | 0.8069 | 0.5509 | 0.7062 | 0.21 | 0.9381 | 2.915432 | high |
| TCGA-AK-3445 | 0.8752 | 0.6792 | 0.2418 | 0.2445 | 0.8894 | 1.095566 | high |
| TCGA-BP-4351 | 0.7279 | 0.4271 | 0.5084 | 0.2227 | 0.8859 | 4.172133 | high |
| TCGA-B0-5100 | 0.8549 | 0.4225 | 0.5439 | 0.1672 | 0.9444 | 3.227291 | high |
| TCGA-B0-5700 | 0.9056 | 0.9168 | 0.3544 | 0.3035 | 0.8546 | 0.584879 | low |
| TCGA-AK-3429 | 0.8943 | 0.8845 | 0.3462 | 0.3226 | 0.9788 | 0.452697 | low |
| TCGA-BP-4798 | 0.846 | 0.5477 | 0.4557 | 0.2218 | 0.9245 | 1.969217 | high |
| TCGA-BP-4769 | 0.9017 | 0.812 | 0.2358 | 0.2558 | 0.9715 | 0.616619 | low |
| TCGA-CJ-5678 | 0.9398 | 0.8721 | 0.5344 | 0.2881 | 0.9219 | 0.631619 | low |
| TCGA-A3-3316 | 0.9307 | 0.5876 | 0.1379 | 0.2117 | 0.9689 | 0.956532 | high |
| TCGA-B8-5550 | 0.9275 | 0.9135 | 0.1222 | 0.2397 | 0.9805 | 0.436743 | low |
| TCGA-B0-5699 | 0.9465 | 0.8639 | 0.4193 | 0.2654 | 0.981 | 0.54382 | low |
| TCGA-CJ-4888 | 0.8866 | 0.4983 | 0.3405 | 0.1016 | 0.9138 | 2.901017 | high |
| TCGA-BP-4354 | 0.8266 | 0.1742 | 0.3986 | 0.1754 | 0.8406 | 5.983047 | high |
| TCGA-BP-4989 | 0.8426 | 0.6547 | 0.3913 | 0.194 | 0.9357 | 1.668178 | high |
| TCGA-CJ-4873 | 0.8457 | 0.4463 | 0.5417 | 0.1547 | 0.8898 | 3.756904 | high |
| TCGA-B0-4824 | 0.744 | 0.64 | 0.4263 | 0.2817 | 0.812 | 2.192625 | high |
| TCGA-CZ-5455 | 0.92 | 0.9359 | 0.2618 | 0.2906 | 0.9645 | 0.409622 | low |
| TCGA-BP-4164 | 0.869 | 0.7934 | 0.3993 | 0.2137 | 0.8683 | 1.250562 | high |
| TCGA-CW-5581 | 0.9178 | 0.8844 | 0.2634 | 0.2978 | 0.985 | 0.423865 | low |
| TCGA-A3-3313 | 0.9736 | 0.8928 | 0.5176 | 0.3381 | 0.9824 | 0.378864 | low |
| TCGA-B0-4845 | 0.8313 | 0.533 | 0.5731 | 0.3163 | 0.9133 | 1.631883 | high |
| TCGA-B4-5378 | 0.8822 | 0.8244 | 0.1256 | 0.2808 | 0.9547 | 0.535175 | low |
| TCGA-BP-5174 | 0.9231 | 0.8388 | 0.3039 | 0.1993 | 0.9366 | 0.802121 | low |
| TCGA-BP-4801 | 0.8978 | 0.8858 | 0.2322 | 0.2755 | 0.9778 | 0.488756 | low |
| TCGA-CJ-5686 | 0.9086 | 0.7965 | 0.2505 | 0.2148 | 0.9361 | 0.810916 | low |
| TCGA-A3-3349 | 0.8666 | 0.6556 | 0.4551 | 0.2765 | 0.949 | 1.119603 | high |
| TCGA-DV-5567 | 0.9085 | 0.8771 | 0.1527 | 0.3095 | 0.9402 | 0.415417 | low |
| TCGA-CZ-5987 | 0.8548 | 0.7952 | 0.3174 | 0.2088 | 0.9346 | 1.066023 | high |
| TCGA-CJ-4885 | 0.9253 | 0.9048 | 0.2266 | 0.3108 | 0.9559 | 0.385722 | low |
| TCGA-DV-5574 | 0.8724 | 0.8766 | 0.2146 | 0.2026 | 0.973 | 0.733626 | low |
| TCGA-A3-3331 | 0.8742 | 0.7417 | 0.2538 | 0.2688 | 0.9482 | 0.78262 | low |
| TCGA-BP-5178 | 0.8932 | 0.816 | 0.5007 | 0.1374 | 0.9497 | 1.426076 | high |
| TCGA-CJ-4871 | 0.9277 | 0.8701 | 0.2227 | 0.2229 | 0.9542 | 0.596801 | low |
| TCGA-B0-4836 | 0.8598 | 0.624 | 0.3833 | 0.3756 | 0.934 | 0.764944 | low |
| TCGA-B8-A54I | 0.8304 | 0.7436 | 0.6224 | 0.224 | 0.8818 | 1.813997 | high |
| TCGA-B0-4821 | 0.652 | 0.3999 | 0.8165 | 0.0853 | 0.8442 | 15.09534 | high |
| TCGA-BP-4777 | 0.8916 | 0.8448 | 0.3131 | 0.2909 | 0.9641 | 0.564355 | low |
| TCGA-CJ-4636 | 0.826 | 0.7367 | 0.4877 | 0.2219 | 0.9424 | 1.445445 | high |
| TCGA-B0-5711 | 0.9283 | 0.8495 | 0.2618 | 0.2854 | 0.9744 | 0.472169 | low |
| TCGA-CZ-4857 | 0.8576 | 0.2315 | 0.368 | 0.3211 | 0.9343 | 2.055398 | high |
| TCGA-B0-4852 | 0.7797 | 0.7004 | 0.3173 | 0.2227 | 0.9263 | 1.570136 | high |
| TCGA-A3-3329 | 0.926 | 0.844 | 0.3966 | 0.2287 | 0.9893 | 0.679392 | low |
| TCGA-CZ-5466 | 0.8825 | 0.8236 | 0.5089 | 0.2163 | 0.9721 | 0.996829 | high |
| TCGA-BP-5177 | 0.9026 | 0.606 | 0.2046 | 0.3149 | 0.967 | 0.697136 | low |
| TCGA-A3-3372 | 0.9195 | 0.7399 | 0.1989 | 0.2839 | 0.9593 | 0.587128 | low |
| TCGA-BP-4804 | 0.9138 | 0.6957 | 0.3448 | 0.1746 | 0.9662 | 1.187094 | high |
| TCGA-AK-3434 | 0.8121 | 0.6774 | 0.4783 | 0.2696 | 0.9031 | 1.490581 | high |
| TCGA-B0-4811 | 0.7881 | 0.4389 | 0.6601 | 0.1556 | 0.8974 | 5.071436 | high |
| TCGA-BP-4165 | 0.852 | 0.6607 | 0.5038 | 0.3774 | 0.9408 | 0.80566 | low |
| TCGA-B0-5099 | 0.8602 | 0.7553 | 0.4442 | 0.2716 | 0.936 | 0.978149 | high |
| TCGA-BP-4766 | 0.9032 | 0.8705 | 0.2832 | 0.2849 | 0.9742 | 0.503819 | low |
| TCGA-BP-4335 | 0.7722 | 0.4693 | 0.3593 | 0.1944 | 0.7696 | 4.151994 | high |
| TCGA-B0-4703 | 0.8723 | 0.1203 | 0.3279 | 0.1567 | 0.9361 | 4.712466 | high |
| TCGA-CJ-4904 | 0.9041 | 0.8716 | 0.2158 | 0.3046 | 0.9801 | 0.425324 | low |
| TCGA-BP-4352 | 0.8781 | 0.0821 | 0.6537 | 0.2006 | 0.7797 | 7.977102 | high |
| TCGA-CJ-4635 | 0.9053 | 0.4889 | 0.2951 | 0.2725 | 0.9711 | 1.129139 | high |
| TCGA-CJ-5679 | 0.9483 | 0.401 | 0.2194 | 0.1463 | 1 | 1.744218 | high |
| TCGA-CZ-4862 | 0.8706 | 0.8187 | 0.2685 | 0.2812 | 0.9577 | 0.642753 | low |
| TCGA-B8-4622 | 0.882 | 0.7578 | 0.279 | 0.2129 | 0.8891 | 1.093627 | high |
| TCGA-G6-A8L8 | 0.9069 | 0.6948 | 0.5792 | 0.2508 | 0.7349 | 1.8173 | high |
| TCGA-CJ-5682 | 0.8733 | 0.8656 | 0.4453 | 0.2621 | 0.9688 | 0.735174 | low |
| TCGA-BP-4176 | 0.8856 | 0.6573 | 0.284 | 0.2483 | 0.9389 | 1.020001 | high |
| TCGA-B8-4148 | 0.9066 | 0.8171 | 0.4211 | 0.2625 | 0.9456 | 0.742249 | low |
| TCGA-CJ-4886 | 0.8824 | 0.7879 | 0.2549 | 0.266 | 0.9588 | 0.689861 | low |
| TCGA-BP-5186 | 0.8774 | 0.7916 | 0.2608 | 0.2867 | 0.9757 | 0.61802 | low |
| TCGA-BP-4972 | 0.8877 | 0.8486 | 0.2793 | 0.2714 | 0.9743 | 0.58356 | low |
| TCGA-BP-4993 | 0.8547 | 0.7925 | 0.4677 | 0.2514 | 0.9245 | 1.059885 | high |
| TCGA-CW-6097 | 0.9126 | 0.7652 | 0.1481 | 0.2356 | 0.9857 | 0.631824 | low |
| TCGA-B0-5077 | 0.8973 | 0.7603 | 0.347 | 0.2417 | 0.9254 | 0.90603 | high |
| TCGA-A3-3383 | 0.9063 | 0.791 | 0.6188 | 0.2746 | 0.8384 | 1.136707 | high |
| TCGA-B0-4819 | 0.8311 | 0.4153 | 0.6752 | 0.1226 | 0.8164 | 6.418947 | high |
| TCGA-BP-4775 | 0.8901 | 0.8142 | 0.3506 | 0.3065 | 0.9478 | 0.605111 | low |
| TCGA-BP-4327 | 0.8467 | 0.5971 | 0.5246 | 0.2605 | 0.9203 | 1.633813 | high |
| TCGA-B0-5399 | 0.8989 | 0.6946 | 0.4695 | 0.2077 | 0.9099 | 1.383973 | high |
| TCGA-A3-3357 | 0.9442 | 0.8285 | 0.3404 | 0.2983 | 0.9293 | 0.526278 | low |
| TCGA-B0-4694 | 0.8423 | 0.8104 | 0.4888 | 0.2659 | 0.9146 | 1.044752 | high |
| TCGA-A3-3317 | 0.9281 | 0.7685 | 0.1376 | 0.1657 | 0.9729 | 0.819282 | high |
| TCGA-B8-4154 | 0.8723 | 0.8571 | 0.4709 | 0.299 | 0.9779 | 0.643971 | low |
| TCGA-B2-5636 | 0.9141 | 0.8713 | 0.1779 | 0.239 | 0.9691 | 0.538076 | low |
| TCGA-B0-4817 | 0.8372 | 0.785 | 0.4944 | 0.1114 | 0.8521 | 2.488916 | high |
| TCGA-BP-5010 | 0.775 | 0.682 | 0.5584 | 0.1356 | 0.8869 | 3.321601 | high |
| TCGA-BP-4325 | 0.8393 | 0.6931 | 0.2463 | 0.3075 | 0.8883 | 0.921147 | high |
| TCGA-BP-5008 | 0.8742 | 0.7388 | 0.2602 | 0.2732 | 0.9342 | 0.800907 | low |
| TCGA-B0-5707 | 0.9123 | 0.8991 | 0.2118 | 0.1745 | 0.9057 | 0.801038 | low |
| TCGA-CZ-5454 | 0.8331 | 0.8968 | 0.1671 | 0.2294 | 0.9134 | 0.774372 | low |
| TCGA-BP-4807 | 0.8786 | 0.86 | 0.3032 | 0.303 | 0.9756 | 0.524333 | low |
| TCGA-A3-3352 | 0.8958 | 0.7615 | 0.3634 | 0.2777 | 0.9551 | 0.742483 | low |
| TCGA-B0-5712 | 0.9206 | 0.8245 | 0.2403 | 0.188 | 0.9339 | 0.823936 | high |
| TCGA-B0-4843 | 0.8109 | 0.6009 | 0.672 | 0.1727 | 0.8527 | 3.555075 | high |
| TCGA-B8-A54H | 0.8852 | 0.8379 | 0.3154 | 0.2836 | 0.9233 | 0.659094 | low |
| TCGA-CZ-5456 | 0.8754 | 0.8349 | 0.3884 | 0.2346 | 0.9897 | 0.788013 | low |
| TCGA-B0-4828 | 0.8371 | 0.6646 | 0.6017 | 0.2458 | 0.8654 | 1.912257 | high |
| TCGA-AK-3444 | 0.8485 | 0.8592 | 0.1925 | 0.2666 | 0.9623 | 0.623963 | low |
| TCGA-CJ-6031 | 0.9189 | 0.8427 | 0.2677 | 0.2481 | 0.9699 | 0.587992 | low |
| TCGA-B8-A54K | 0.8908 | 0.7616 | 0.2029 | 0.3002 | 0.9744 | 0.560236 | low |
| TCGA-DV-5566 | 0.8865 | 0.9054 | 0.3739 | 0.2588 | 0.9464 | 0.645873 | low |
| TCGA-B0-5113 | 0.846 | 0.7597 | 0.3013 | 0.2179 | 0.9533 | 1.068842 | high |
| TCGA-BP-4169 | 0.8142 | 0.607 | 0.373 | 0.2323 | 0.8916 | 1.838432 | high |
| TCGA-CZ-5986 | 0.9289 | 0.8616 | 0.2081 | 0.3051 | 0.974 | 0.401291 | low |
| TCGA-CJ-4640 | 0.9059 | 0.9285 | 0.1478 | 0.2554 | 0.9672 | 0.449083 | low |
| TCGA-B4-5836 | 0.854 | 0.8827 | 0.317 | 0.1954 | 0.92 | 0.984804 | high |
| TCGA-B0-5713 | 0.9089 | 0.855 | 0.1186 | 0.3305 | 0.9568 | 0.369216 | low |
| TCGA-BP-4790 | 0.9095 | 0.7484 | 0.237 | 0.2775 | 0.951 | 0.648388 | low |
| TCGA-BP-4174 | 0.9089 | 0.8857 | 0.2407 | 0.267 | 0.9588 | 0.513605 | low |
| TCGA-BP-4161 | 0.8527 | 0.7836 | 0.4587 | 0.2576 | 0.8996 | 1.10484 | high |
| TCGA-BP-5183 | 0.8992 | 0.6666 | 0.3634 | 0.209 | 0.9603 | 1.172685 | high |
| TCGA-CW-5588 | 0.8977 | 0.9259 | 0.1529 | 0.3546 | 0.9783 | 0.297318 | low |
| TCGA-BP-4970 | 0.9058 | 0.8512 | 0.1711 | 0.2781 | 0.9626 | 0.489585 | low |
| TCGA-A3-3319 | 0.8727 | 0.8179 | 0.3704 | 0.2646 | 0.8808 | 0.895834 | high |
| TCGA-BP-4349 | 0.7566 | 0.522 | 0.439 | 0.2367 | 0.9006 | 2.690258 | high |
| TCGA-A3-3335 | 0.8379 | 0.7088 | 0.5721 | 0.1402 | 0.9047 | 2.457415 | high |
| TCGA-CZ-5464 | 0.9078 | 0.8715 | 0.3534 | 0.2813 | 0.9423 | 0.577626 | low |
| TCGA-AK-3436 | 0.9178 | 0.8016 | 0.6434 | 0.1787 | 0.9773 | 1.232108 | high |
| TCGA-AK-3427 | 0.8395 | 0.9245 | 0.0928 | 0.1733 | 0.9401 | 0.801748 | low |
| TCGA-BP-4163 | 0.9164 | 0.8213 | 0.3771 | 0.2243 | 0.9433 | 0.807954 | low |
| TCGA-BP-4170 | 0.8295 | 0.8646 | 0.3053 | 0.2746 | 0.9609 | 0.71238 | low |
| TCGA-B0-5088 | 0.8535 | 0.7092 | 0.5088 | 0.1926 | 0.8697 | 1.886099 | high |
| TCGA-BP-4329 | 0.8179 | 0.7613 | 0.2745 | 0.3018 | 0.9584 | 0.784707 | low |
| TCGA-CW-5591 | 0.9211 | 0.9324 | 0.1736 | 0.205 | 0.9516 | 0.558555 | low |
| TCGA-CW-5580 | 0.8807 | 0.8218 | 0.3281 | 0.2303 | 0.9684 | 0.797629 | low |
| TCGA-BP-4177 | 0.9055 | 0.8748 | 0.2335 | 0.2414 | 0.9628 | 0.582785 | low |
| TCGA-B0-4837 | 0.8473 | 0.7356 | 0.6146 | 0.196 | 0.8293 | 2.182917 | high |
| TCGA-CJ-4907 | 0.911 | 0.813 | 0.2541 | 0.2978 | 0.9764 | 0.502491 | low |
| TCGA-CJ-4891 | 0.8048 | 0.4445 | 0.5807 | 0.101 | 0.7889 | 6.997301 | high |
| TCGA-A3-3365 | 0.886 | 0.7233 | 0.3453 | 0.262 | 0.9701 | 0.840047 | high |
| TCGA-BP-4982 | 0.9375 | 0.652 | 0.1862 | 0.2776 | 0.9641 | 0.659588 | low |
| TCGA-B8-5549 | 0.8805 | 0.8816 | 0.1965 | 0.4006 | 0.9689 | 0.299846 | low |
| TCGA-MM-A563 | 0.8745 | 0.7235 | 0.5959 | 0.2068 | 0.8934 | 1.670989 | high |
| TCGA-B0-4688 | 0.6716 | 0.4729 | 0.4707 | 0.2069 | 0.6392 | 8.011907 | high |
| TCGA-BP-5194 | 0.9073 | 0.8054 | 0.3272 | 0.331 | 0.9782 | 0.479623 | low |
| TCGA-CJ-4634 | 0.8835 | 0.8435 | 0.4003 | 0.3132 | 0.9278 | 0.622361 | low |
| TCGA-CZ-5462 | 0.9289 | 0.8719 | 0.1854 | 0.1123 | 0.8619 | 1.117569 | high |
| TCGA-CJ-4902 | 0.9131 | 0.5961 | 0.387 | 0.2493 | 0.9185 | 1.210675 | high |
| TCGA-BP-4981 | 0.8257 | 0.6022 | 0.5093 | 0.1504 | 0.8481 | 3.193028 | high |
| TCGA-A3-A6NI | 0.9359 | 0.7301 | 0.4706 | 0.2349 | 0.9336 | 0.969485 | high |
| TCGA-BP-5195 | 0.8832 | 0.8608 | 0.2042 | 0.2931 | 0.9719 | 0.491422 | low |
| TCGA-BP-4782 | 0.9081 | 0.8599 | 0.2417 | 0.2707 | 0.9578 | 0.534593 | low |
| TCGA-A3-3311 | 0.905 | 0.8417 | 0.3526 | 0.281 | 0.9577 | 0.597976 | low |
| TCGA-CZ-4864 | 0.9266 | 0.8418 | 0.2961 | 0.3357 | 0.927 | 0.444827 | low |
| TCGA-B2-4102 | 0.9228 | 0.7949 | 0.2816 | 0.2467 | 0.9589 | 0.665379 | low |
| TCGA-CJ-4878 | 0.8291 | 0.5438 | 0.4931 | 0.2504 | 0.6695 | 3.321758 | high |
| TCGA-AK-3428 | 0.8092 | 0.8969 | 0.0808 | 0.3494 | 0.9334 | 0.439762 | low |
| TCGA-BP-4971 | 0.7689 | 0.4285 | 0.4401 | 0.1692 | 0.8916 | 4.226113 | high |
| TCGA-CJ-5676 | 0.9161 | 0.8113 | 0.5191 | 0.2424 | 0.8326 | 1.113925 | high |
| TCGA-B8-A54J | 0.878 | 0.8054 | 0.3721 | 0.2883 | 0.8739 | 0.828471 | high |
| TCGA-BP-4999 | 0.9049 | 0.7438 | 0.2766 | 0.2871 | 0.9584 | 0.652545 | low |
| TCGA-BP-4332 | 0.9111 | 0.7748 | 0.3835 | 0.2746 | 0.9518 | 0.716691 | low |
| TCGA-CZ-4863 | 0.8727 | 0.8552 | 0.3224 | 0.2387 | 0.9465 | 0.77117 | low |
| TCGA-A3-3374 | 0.857 | 0.909 | 0.1704 | 0.3035 | 0.6647 | 0.869534 | high |
| TCGA-A3-3326 | 0.9358 | 0.7516 | 0.3352 | 0.2718 | 0.9496 | 0.670371 | low |
| TCGA-T7-A92I | 0.9617 | 0.592 | 0.2656 | 0.2015 | 0.9623 | 1.031559 | high |
| TCGA-AK-3425 | 0.8286 | 0.699 | 0.3802 | 0.2122 | 0.8993 | 1.584016 | high |
| TCGA-CJ-4894 | 0.9331 | 0.6809 | 0.266 | 0.2265 | 0.984 | 0.817208 | low |
| TCGA-AK-3458 | 0.9193 | 0.4484 | 0.7144 | 0.2893 | 0.9577 | 1.697271 | high |
| TCGA-B0-5106 | 0.8349 | 0.5562 | 0.4598 | 0.244 | 0.8763 | 2.032517 | high |
| TCGA-A3-3382 | 0.9197 | 0.2667 | 0.1967 | 0.248 | 0.92 | 1.864572 | high |
| TCGA-BP-4353 | 0.8854 | 0.8092 | 0.2915 | 0.2464 | 0.9213 | 0.801165 | low |
| TCGA-B0-5698 | 0.9028 | 0.8518 | 0.2919 | 0.2742 | 0.9281 | 0.609777 | low |
| TCGA-CZ-4866 | 0.9293 | 0.7882 | 0.2102 | 0.2786 | 0.9304 | 0.569744 | low |
| TCGA-BP-4799 | 0.8147 | 0.7051 | 0.4354 | 0.1238 | 0.9566 | 2.236741 | high |
| TCGA-CW-5583 | 0.9295 | 0.854 | 0.3044 | 0.2681 | 0.9737 | 0.524702 | low |
| TCGA-B0-5085 | 0.7884 | 0.5892 | 0.5611 | 0.2194 | 0.7137 | 3.861464 | high |
| TCGA-CJ-5683 | 0.8838 | 0.8445 | 0.3157 | 0.2981 | 0.9458 | 0.585552 | low |
| TCGA-CW-5590 | 0.8961 | 0.7475 | 0.2681 | 0.268 | 0.9613 | 0.713104 | low |
| TCGA-CZ-5457 | 0.8945 | 0.8534 | 0.2843 | 0.2443 | 0.9512 | 0.670684 | low |
| TCGA-B8-A7U6 | 0.8444 | 0.7835 | 0.5772 | 0.218 | 0.7479 | 2.094844 | high |
| TCGA-BP-5185 | 0.8771 | 0.8009 | 0.6138 | 0.2571 | 0.8837 | 1.19391 | high |
| TCGA-BP-4763 | 0.9109 | 0.8835 | 0.409 | 0.1868 | 0.962 | 0.849036 | high |
| TCGA-BP-5180 | 0.9262 | 0.8007 | 0.4068 | 0.255 | 0.9318 | 0.753805 | low |
| TCGA-B2-5635 | 0.9055 | 0.8309 | 0.2315 | 0.2567 | 0.959 | 0.598068 | low |
| TCGA-BP-4789 | 0.9128 | 0.7961 | 0.2017 | 0.2648 | 0.9817 | 0.558122 | low |
| TCGA-A3-A8OV | 0.9408 | 0.8283 | 0.3859 | 0.3868 | 0.9916 | 0.333526 | low |
| TCGA-CJ-4643 | 0.821 | 0.7659 | 0.3552 | 0.2803 | 0.9354 | 0.961132 | high |
| TCGA-BP-4326 | 0.7728 | 0.4996 | 0.4998 | 0.2502 | 0.943 | 2.440861 | high |
| TCGA-BP-4960 | 0.8089 | 0.6493 | 0.5416 | 0.2151 | 0.893 | 2.18741 | high |
| TCGA-B0-5121 | 0.8507 | 0.7716 | 0.3975 | 0.2371 | 0.9188 | 1.122355 | high |
| TCGA-B2-4099 | 0.8958 | 0.8745 | 0.3534 | 0.2313 | 0.9876 | 0.671669 | low |
| TCGA-BP-5168 | 0.9451 | 0.8152 | 0.378 | 0.3445 | 0.9717 | 0.418776 | low |
| TCGA-DV-A4W0 | 0.9153 | 0.7603 | 0.3548 | 0.2782 | 0.9392 | 0.714848 | low |
| TCGA-CJ-4905 | 0.8663 | 0.8463 | 0.2888 | 0.3277 | 0.9893 | 0.482632 | low |
| TCGA-CJ-5675 | 0.9302 | 0.8517 | 0.3695 | 0.2241 | 0.9698 | 0.683332 | low |
| TCGA-AK-3443 | 0.8802 | 0.883 | 0.2369 | 0.2573 | 0.9399 | 0.613099 | low |
| TCGA-MW-A4EC | 0.8778 | 0.8744 | 0.3213 | 0.3771 | 0.9534 | 0.397187 | low |
| TCGA-A3-3325 | 0.8813 | 0.7511 | 0.2601 | 0.305 | 0.9453 | 0.650942 | low |
| TCGA-CZ-5469 | 0.7967 | 0.2576 | 0.6182 | 0.2153 | 0.9522 | 4.632523 | high |
| TCGA-AK-3453 | 0.9214 | 0.883 | 0.3162 | 0.2558 | 1 | 0.513416 | low |
| TCGA-CZ-5452 | 0.937 | 0.8362 | 0.3716 | 0.1309 | 0.8826 | 1.240674 | high |
| TCGA-CJ-4901 | 0.8088 | 0.6608 | 0.7359 | 0.1672 | 0.8827 | 3.260259 | high |
| TCGA-B0-4700 | 0.8449 | 0.8237 | 0.5943 | 0.1583 | 0.9316 | 1.715583 | high |
| TCGA-B2-3923 | 0.9535 | 0.8794 | 0.197 | 0.263 | 0.8804 | 0.517841 | low |
| TCGA-BP-5184 | 0.8677 | 0.7595 | 0.2781 | 0.3024 | 0.9473 | 0.686241 | low |
| TCGA-B0-4810 | 0.8044 | 0.5717 | 0.5497 | 0.2199 | 0.951 | 2.251722 | high |
| TCGA-BP-5176 | 0.9297 | 0.8438 | 0.3549 | 0.2515 | 0.9772 | 0.599478 | low |
| TCGA-CZ-5463 | 0.9643 | 0.9122 | 0.343 | 0.3418 | 0.975 | 0.315998 | low |
| TCGA-B2-5633 | 0.929 | 0.7999 | 0.2492 | 0.1723 | 0.9802 | 0.821971 | high |
| TCGA-B8-A54E | 0.8129 | 0.9164 | 0.2269 | 0.0566 | 0.9798 | 1.538472 | high |
| TCGA-B0-4844 | 0.8155 | 0.7524 | 0.6328 | 0.2278 | 0.9085 | 1.757562 | high |
| TCGA-BP-5169 | 0.8899 | 0.5166 | 0.8917 | 0.1252 | 0.9077 | 4.392074 | high |
| TCGA-B0-4714 | 0.7968 | 0.503 | 0.4764 | 0.2592 | 0.8313 | 2.676652 | high |
| TCGA-CZ-4853 | 0.9405 | 0.8239 | 0.4771 | 0.2463 | 0.9226 | 0.780905 | low |

Table S8

| ID | GK\|88735\|ES | SIPA1L1\|28199\|ES | MGRN1\|33783\|ES | CLEC1A\|20304\|ES | C16orf13\|32919\|ES | KLHL42\|20900\|ES | SEC31A\|100881\|ES | Risk score | risk |
| --- | --- | --- | --- | --- | --- | --- | --- | --- | --- |
| TCGA-CJ-4637 | 0.4551 | 0.1111 | 0.9945 | 0.5253 | 0.9748 | 0.229 | 0.5174 | 2.009745 | high |
| TCGA-CZ-4861 | 0.1696 | 0.0275 | 0.9782 | 0.3262 | 0.9596 | 0.1909 | 0.4188 | 1.208691 | high |
| TCGA-BP-4964 | 0.1866 | 0.0491 | 0.9834 | 0.2017 | 0.9822 | 0.1427 | 0.7604 | 0.599342 | low |
| TCGA-CJ-4887 | 0.1741 | 0.1061 | 0.9792 | 0.3584 | 0.9863 | 0.5509 | 0.9205 | 0.887611 | high |
| TCGA-BP-5198 | 0.1868 | 0.1024 | 0.9891 | 0.1167 | 0.9736 | 0.1869 | 0.4415 | 1.014585 | high |
| TCGA-BP-4967 | 0.0975 | 0.0707 | 0.9957 | 0.3089 | 0.9879 | 0.1909 | 0.8375 | 0.497779 | low |
| TCGA-A3-3351 | 0.4037 | 0.0782 | 0.9881 | 0.2591 | 0.993 | 0.3206 | 0.6817 | 1.167481 | high |
| TCGA-B0-4710 | 0.4088 | 0.1541 | 0.9582 | 0.3898 | 0.9458 | 0.3949 | 0.7328 | 2.234315 | high |
| TCGA-B0-5083 | 0.1603 | 0 | 1 | 0.0511 | 0.9886 | 0.4855 | 0.7208 | 0.608137 | low |
| TCGA-BP-4803 | 0.2914 | 0.1094 | 0.9924 | 0.1335 | 0.973 | 0.1817 | 0.6125 | 0.949555 | high |
| TCGA-CJ-4889 | 0.1751 | 0.0916 | 0.9961 | 0.3526 | 0.9904 | 0.1846 | 0.7255 | 0.698609 | low |
| TCGA-B0-5080 | 0.6728 | 0.1393 | 0.9616 | 0.108 | 0.9929 | 0.1277 | 0.1877 | 3.515541 | high |
| TCGA-CW-5587 | 0.2438 | 0.0608 | 0.9913 | 0.0334 | 0.9862 | 0.1909 | 0.8062 | 0.540114 | low |
| TCGA-A3-3376 | 0.296 | 0.0641 | 0.9875 | 0.2709 | 0.988 | 0.1682 | 0.5431 | 1.033267 | high |
| TCGA-CJ-4897 | 0.078 | 0.0186 | 0.994 | 0.2933 | 0.9935 | 0.1475 | 0.8974 | 0.376149 | low |
| TCGA-CW-6088 | 0.1074 | 0.0837 | 1 | 0.139 | 0.9843 | 0.1503 | 0.879 | 0.404862 | low |
| TCGA-B8-5552 | 0.233 | 0.0728 | 0.9904 | 0.1856 | 0.9897 | 0.1022 | 0.7078 | 0.638012 | low |
| TCGA-CW-5589 | 0.0693 | 0.0125 | 0.9855 | 0.0693 | 0.9896 | 0.1989 | 0.8895 | 0.349625 | low |
| TCGA-B0-4833 | 0.311 | 0.2051 | 0.9899 | 0.8582 | 0.9894 | 0.2123 | 0.78 | 1.594686 | high |
| TCGA-CZ-4859 | 0.1021 | 0 | 0.9929 | 0.0747 | 0.9926 | 0.1248 | 0.9696 | 0.283406 | low |
| TCGA-A3-3320 | 0.2461 | 0.1867 | 0.9965 | 0.2823 | 0.9868 | 0.2105 | 0.8771 | 0.737227 | low |
| TCGA-MM-A564 | 0.5187 | 0.1147 | 1 | 0.1913 | 0.9651 | 0.36686 | 0.2546 | 2.888782 | high |
| TCGA-AS-3777 | 0.1272 | 0.0358 | 0.9811 | 0.3675 | 0.9683 | 0.407 | 0.876 | 0.723912 | low |
| TCGA-B0-5095 | 0.6533 | 0.1919 | 0.9659 | 0.2276 | 0.9855 | 0.2981 | 0.1461 | 5.208462 | high |
| TCGA-BP-4795 | 0.4353 | 0.0578 | 0.9863 | 0.108 | 0.9955 | 0.0895 | 0.5454 | 1.01216 | high |
| TCGA-BP-4331 | 0.2009 | 0.0902 | 0.9851 | 0.3637 | 0.9856 | 0.3337 | 0.6929 | 0.97489 | high |
| TCGA-A3-A6NJ | 0.0937 | 0.0593 | 0.9949 | 0.132 | 0.9388 | 0.1451 | 0.9122 | 0.454668 | low |
| TCGA-B0-4701 | 0.2714 | 0.1137 | 0.9812 | 0.4207 | 0.9806 | 0.3019 | 0.6047 | 1.375352 | high |
| TCGA-A3-3367 | 0.1412 | 0.0191 | 1 | 0.1326 | 0.9753 | 0.225 | 0.8612 | 0.437406 | low |
| TCGA-B0-5402 | 0.3342 | 0.1888 | 0.9914 | 0.0616 | 0.955 | 0.1804 | 0.8038 | 0.920896 | high |
| TCGA-B0-5691 | 0.0403 | 0.0182 | 1 | 0.017 | 1 | 0.1359 | 0.763 | 0.316334 | low |
| TCGA-BP-4342 | 0.4396 | 0.1016 | 0.9921 | 0.492 | 0.9238 | 0.3891 | 0.776 | 1.954085 | high |
| TCGA-A3-3373 | 0.0345 | 0.0401 | 0.9962 | 0.1306 | 0.9915 | 0.2185 | 0.8109 | 0.385285 | low |
| TCGA-BP-4341 | 0.0879 | 0.1983 | 0.9736 | 0.3958 | 0.985 | 0.4471 | 0.8897 | 0.915818 | high |
| TCGA-CW-6090 | 0.4551 | 0.0381 | 0.9773 | 0.139 | 0.9916 | 0.434 | 0.2301 | 2.454199 | high |
| TCGA-A3-3307 | 0.1634 | 0.0246 | 0.995 | 0.252 | 0.9846 | 0.1523 | 0.7792 | 0.520808 | low |
| TCGA-B2-3924 | 0.3462 | 0.3845 | 0.9772 | 0.7771 | 1 | 0.7023 | 0.661022 | 4.316442 | high |
| TCGA-B0-5706 | 0.1463 | 0 | 1 | 0.18368 | 0.9818 | 0.15258 | 0.5222 | 0.648868 | low |
| TCGA-BP-4963 | 0.3657 | 0.1258 | 0.9858 | 0.2999 | 0.9616 | 0.1953 | 0.6367 | 1.357962 | high |
| TCGA-BP-4343 | 0.229 | 0.0471 | 1 | 0.0747 | 0.9711 | 0.2955 | 0.581 | 0.824961 | low |
| TCGA-B0-5703 | 0.0291 | 0.0714 | 0.9901 | 0.1827 | 0.995 | 0.2206 | 0.8941 | 0.382967 | low |
| TCGA-CJ-6027 | 0.2522 | 0 | 0.9928 | 0.1537 | 0.9719 | 0.3398 | 0.7651 | 0.704708 | low |
| TCGA-B0-5108 | 0.1772 | 0.0918 | 0.9863 | 0.1238 | 0.9797 | 0.2798 | 0.338 | 1.23862 | high |
| TCGA-BP-4965 | 0.1567 | 0.0271 | 0.9974 | 0.1175 | 0.9946 | 0.1179 | 0.7491 | 0.440678 | low |
| TCGA-A3-A6NL | 0.1715 | 0.2343 | 0.9878 | 0.1913 | 0.9878 | 0.4653 | 0.6891 | 1.163676 | high |
| TCGA-CJ-5672 | 0.1308 | 0.0275 | 0.994 | 0.4657 | 0.9928 | 0.1733 | 0.7836 | 0.579382 | low |
| TCGA-B8-5158 | 0.4076 | 0.0471 | 0.9601 | 0.2498 | 0.9599 | 0.2123 | 0.6273 | 1.479375 | high |
| TCGA-CJ-4876 | 0.3584 | 0.0232 | 0.963 | 0.6115 | 0.9816 | 0.3206 | 0.9736 | 1.06096 | high |
| TCGA-B0-5115 | 0.2926 | 0.2609 | 0.9738 | 0.2306 | 0.9725 | 0.3206 | 0.7841 | 1.360382 | high |
| TCGA-CJ-4916 | 0.3635 | 0.0724 | 0.9885 | 0.3651 | 0.9746 | 0.4144 | 0.8328 | 1.132621 | high |
| TCGA-B0-4848 | 0.3649 | 0.0158 | 0.9865 | 0.2664 | 0.8972 | 0.0557 | 0.7269 | 1.112999 | high |
| TCGA-B4-5377 | 0.1266 | 0.0552 | 0.9787 | 0.1804 | 0.983 | 0.2221 | 0.8783 | 0.506457 | low |
| TCGA-B0-4712 | 0.3521 | 0.0303 | 0.9424 | 0.1215 | 0.8679 | 0.1652 | 0.2601 | 3.22303 | high |
| TCGA-A3-3324 | 0.1562 | 0.0986 | 0.9827 | 0.2202 | 0.9924 | 0.1846 | 0.8211 | 0.578973 | low |
| TCGA-CZ-5465 | 0.2181 | 0.0572 | 0.9952 | 0.1497 | 0.9466 | 0.2419 | 0.8907 | 0.617067 | low |
| TCGA-CZ-4860 | 0.472 | 0.0974 | 0.9897 | 0.20627 | 0.6793 | 0.5412 | 0.088 | 15.59014 | high |
| TCGA-B0-5102 | 0.1199 | 0.104 | 0.9934 | 0.3923 | 0.9752 | 0.4433 | 0.8785 | 0.764018 | low |
| TCGA-B0-5690 | 0.2088 | 0.0512 | 0.9809 | 0.087 | 0.9906 | 0.1312 | 0.6437 | 0.648525 | low |
| TCGA-BP-4160 | 0.1336 | 0.1165 | 0.9868 | 0.1005 | 0.9958 | 0.0949 | 0.8384 | 0.44792 | low |
| TCGA-B0-4697 | 0.1616 | 0.25226 | 1 | 0.6449 | 0.9041 | 0.739 | 0.658 | 3.18056 | high |
| TCGA-AK-3454 | 0.2561 | 0.0928 | 0.9892 | 0.257 | 0.9922 | 0.121 | 0.8519 | 0.601726 | low |
| TCGA-B0-5693 | 0.1011 | 0.0797 | 0.9712 | 0.0819 | 0.9885 | 0.0949 | 0.7745 | 0.488161 | low |
| TCGA-BP-4985 | 0.3979 | 0.1527 | 0.9804 | 0.244 | 0.9684 | 0.1485 | 0.1205 | 2.853982 | high |
| TCGA-B0-4718 | 0.21 | 0.1749 | 1 | 0.4296 | 0.9678 | 0.6536 | 0.7683 | 1.482841 | high |
| TCGA-B0-5109 | 0.2658 | 0.1001 | 0.9379 | 0.3675 | 0.9646 | 0.6016 | 0.1491 | 4.598249 | high |
| TCGA-A3-3359 | 0.1876 | 0.1091 | 0.9952 | 0.263 | 0.9892 | 0.1427 | 0.8643 | 0.545199 | low |
| TCGA-DV-5569 | 0.102 | 0.0194 | 0.9767 | 0.139 | 0.988 | 0.2238 | 0.8244 | 0.472307 | low |
| TCGA-B2-A4SR | 0.2678 | 0.0518 | 0.9905 | 0.1763 | 1 | 0.177 | 0.7999 | 0.582554 | low |
| TCGA-B0-4815 | 0.2552 | 0.0691 | 0.9588 | 0.4735 | 0.9569 | 0.502 | 0.522 | 2.266068 | high |
| TCGA-AK-3461 | 0.2886 | 0.148 | 0.9786 | 0.1357 | 0.9689 | 0.2077 | 0.7448 | 0.956484 | high |
| TCGA-CJ-4892 | 0.1403 | 0.1989 | 1 | 0.3262 | 0.9792 | 0.3861 | 0.8968 | 0.76902 | low |
| TCGA-B0-5695 | 0.1221 | 0.094 | 0.9871 | 0.1173 | 0.9589 | 0.2277 | 0.8823 | 0.542643 | low |
| TCGA-BP-4975 | 0.1326 | 0.052 | 0.9968 | 0.1015 | 0.9768 | 0.1909 | 0.79 | 0.48275 | low |
| TCGA-AK-3456 | 0.074 | 0.09 | 0.9868 | 0.28645 | 0.9658 | 0.2026 | 0.5265 | 0.893882 | high |
| TCGA-BP-4159 | 0.1705 | 0.145 | 1 | 0.1856 | 0.9817 | 0.1359 | 0.4695 | 0.926683 | high |
| TCGA-CZ-5458 | 0.1822 | 0.1372 | 0.9959 | 0.212 | 0.9741 | 0.0498 | 0.7653 | 0.610025 | low |
| TCGA-AK-3431 | 0.4926 | 0.05262 | 1 | 0.1449 | 0.9748 | 0.0631 | 0.5159 | 1.165953 | high |
| TCGA-B8-A8YJ | 0.322 | 0.1775 | 0.9854 | 0.1869 | 0.9534 | 0.259 | 0.2888 | 2.294663 | high |
| TCGA-BP-5182 | 0.0746 | 0.0808 | 0.9927 | 0.4133 | 0.9856 | 0.1955 | 0.7988 | 0.579751 | low |
| TCGA-B2-4101 | 0.2569 | 0.1253 | 0.9957 | 0.3376 | 0.9876 | 0.4325 | 0.5529 | 1.39187 | high |
| TCGA-CJ-5681 | 0.0446 | 0.0765 | 0.9862 | 0.27847 | 0.9509 | 0.1909 | 0.7876 | 0.606036 | low |
| TCGA-BP-4994 | 0.0951 | 0.0139 | 0.9974 | 0.3397 | 0.9972 | 0.2229 | 0.9075 | 0.407573 | low |
| TCGA-CJ-4903 | 0.1361 | 0.0709 | 0.991 | 0.128 | 0.977 | 0.2035 | 0.8283 | 0.509276 | low |
| TCGA-BP-4760 | 0.5363 | 0.0512 | 1 | 0.113 | 0.992 | 0.0117 | 0.4976 | 1.098105 | high |
| TCGA-BP-5190 | 0.0816 | 0.1802 | 0.9875 | 0.31671 | 0.9745 | 0.114 | 0.8931 | 0.566845 | low |
| TCGA-B0-5710 | 0.2483 | 0.0963 | 0.9689 | 0.1162 | 0.997 | 0.0574 | 0.5922 | 0.802011 | low |
| TCGA-CJ-4875 | 0.1904 | 0.1821 | 0.9401 | 0.4587 | 0.9896 | 0.586 | 0.6844 | 2.048937 | high |
| TCGA-CJ-4874 | 0.1689 | 0.0753 | 0.9941 | 0.0852 | 0.9897 | 0.1909 | 0.8221 | 0.482787 | low |
| TCGA-B2-5641 | 0.2552 | 0.0298 | 0.9625 | 0.1144 | 0.9889 | 0.1317 | 0.6602 | 0.760144 | low |
| TCGA-CW-5584 | 0.2243 | 0 | 0.9857 | 0.0262 | 0.9773 | 0.1682 | 0.7502 | 0.532823 | low |
| TCGA-B8-4143 | 0.4213 | 0.09 | 0.9548 | 0.3837 | 0.9589 | 0.3935 | 0.63309 | 2.241592 | high |
| TCGA-B0-5120 | 0.2414 | 0.1042 | 0.9945 | 0.1466 | 0.9937 | 0.1975 | 0.6818 | 0.723826 | low |
| TCGA-EU-5905 | 0.2131 | 0.0204 | 1 | 0.1591 | 0.9162 | 0.1435 | 0.6404 | 0.833582 | low |
| TCGA-BP-4338 | 0.1336 | 0.0525 | 0.9674 | 0.21373 | 0.9772 | 0.0949 | 0.7325 | 0.62455 | low |
| TCGA-A3-3358 | 0.1651 | 0.1292 | 0.9859 | 0.2103 | 0.9967 | 0.3331 | 0.8182 | 0.688602 | low |
| TCGA-B0-4818 | 0.1479 | 0.1602 | 0.9787 | 0.1833 | 0.9895 | 0.3776 | 0.7579 | 0.850832 | high |
| TCGA-BP-4976 | 0.0645 | 0.0225 | 0.9978 | 0.0373 | 0.9921 | 0.0584 | 0.9461 | 0.253105 | low |
| TCGA-BP-4345 | 0.4005 | 0.2461 | 1 | 0.0771 | 0.99 | 0.2453 | 0.4572 | 1.621988 | high |
| TCGA-CW-6093 | 0.0807 | 0.0996 | 0.9755 | 0.0925 | 0.9797 | 0.0825 | 0.8571 | 0.439277 | low |
| TCGA-BP-4330 | 0.1927 | 0.1308 | 0.9945 | 0.3543 | 0.9783 | 0.2912 | 0.8276 | 0.792728 | low |
| TCGA-EU-5906 | 0.1299 | 0.0283 | 0.99 | 0.1826 | 0.976 | 0.1107 | 0.8003 | 0.468455 | low |
| TCGA-B0-4846 | 0.2243 | 0.0812 | 0.9648 | 0.3035 | 0.991 | 0.2047 | 0.9454 | 0.645682 | low |
| TCGA-CJ-6030 | 0.2481 | 0.0653 | 0.9797 | 0.1552 | 0.9893 | 0.2097 | 0.5672 | 0.915402 | high |
| TCGA-BP-4983 | 0.3108 | 0.0665 | 0.9408 | 0.8134 | 0.9627 | 0.3844 | 0.1386 | 5.415653 | high |
| TCGA-B0-5697 | 0.1755 | 0.054 | 0.9821 | 0.4365 | 0.9955 | 0.1682 | 0.553 | 0.935982 | high |
| TCGA-BP-4346 | 0.2512 | 0.1678 | 0.9914 | 0.727 | 0.9955 | 0.2922 | 0.7865 | 1.257894 | high |
| TCGA-B0-4691 | 0.3567 | 0.2169 | 0.9723 | 0.6397 | 0.9894 | 0.3816 | 0.5241 | 2.790882 | high |
| TCGA-A3-3328 | 0.0377 | 0.1652 | 0.9863 | 0.6397 | 1 | 0.3761 | 0.9007 | 0.766482 | low |
| TCGA-BP-4765 | 0.1824 | 0.2087 | 0.9871 | 0.3441 | 0.9863 | 0.2393 | 0.8957 | 0.771685 | low |
| TCGA-CZ-4858 | 0.3092 | 0.0603 | 1 | 0.26608 | 0.9301 | 0.2822 | 0.59141 | 1.319581 | high |
| TCGA-B0-5117 | 0.1569 | 0.10338 | 1 | 0.2664 | 0.9919 | 0.3802 | 0.7336 | 0.752061 | low |
| TCGA-CZ-4865 | 0.1899 | 0.1402 | 1 | 0.204 | 0.9836 | 0.2393 | 0.859 | 0.60926 | low |
| TCGA-CJ-6028 | 0.2987 | 0.0453 | 0.9903 | 0.2251 | 0.9743 | 0.2327 | 0.4685 | 1.200257 | high |
| TCGA-6D-AA2E | 0.0211 | 0.0721 | 1 | 0.5578 | 0.9936 | 0.4244 | 0.7195 | 0.760451 | low |
| TCGA-CJ-4884 | 0.2234 | 0.1253 | 0.985 | 0.5848 | 0.9697 | 0.1909 | 0.7998 | 1.032127 | high |
| TCGA-DV-5568 | 0.3082 | 0.0867 | 0.9696 | 0.2906 | 0.9848 | 0.1285 | 0.686 | 0.991275 | high |
| TCGA-BP-4961 | 0.2044 | 0.09 | 0.9787 | 0.1247 | 0.9875 | 0.2134 | 0.7574 | 0.675031 | low |
| TCGA-BP-4761 | 0.106 | 0.1061 | 0.9568 | 1 | 0.9766 | 0.2614 | 0.5059 | 2.151597 | high |
| TCGA-CJ-5677 | 0.1545 | 0.0407 | 0.9779 | 0.492 | 0.9735 | 0.4522 | 0.5916 | 1.316288 | high |
| TCGA-CZ-5460 | 0.1463 | 0.1393 | 0.9938 | 0.139 | 0.9663 | 0.4144 | 0.7314 | 0.86148 | high |
| TCGA-CJ-4900 | 0.273 | 0.1691 | 0.9897 | 0.4822 | 0.9616 | 0.3206 | 0.4466 | 2.109118 | high |
| TCGA-CZ-5451 | 0.1511 | 0.0231 | 0.9909 | 0.1058 | 0.978 | 0.0844 | 0.8891 | 0.382505 | low |
| TCGA-B8-A54F | 0.1675 | 0.1185 | 0.9887 | 0.2081 | 0.9922 | 0.27706 | 0.4188 | 1.131829 | high |
| TCGA-B0-4838 | 0.3285 | 0.0523 | 0.9822 | 0.4466 | 0.9918 | 0.4451 | 0.8468 | 1.069753 | high |
| TCGA-B0-4699 | 0.2947 | 0.1274 | 1 | 0.6854 | 0.9108 | 0.4891 | 0.5841 | 2.757346 | high |
| TCGA-BP-4781 | 0.2301 | 0.056 | 0.9949 | 0.139 | 0.9845 | 0.1417 | 0.8421 | 0.508526 | low |
| TCGA-CJ-4644 | 0.1034 | 0.1775 | 0.9776 | 0.0539 | 0.9515 | 0.1643 | 0.7969 | 0.673321 | low |
| TCGA-B0-5701 | 0.2974 | 0.0619 | 0.9876 | 0.1949 | 1 | 0.0862 | 0.808 | 0.581218 | low |
| TCGA-B0-5694 | 0.1505 | 0.0691 | 1 | 0.1694 | 0.9949 | 0.2206 | 0.3942 | 0.881767 | high |
| TCGA-B8-4621 | 0.0592 | 0.0988 | 0.9784 | 0.30325 | 0.9876 | 0.3105 | 0.7299 | 0.714157 | low |
| TCGA-BP-5006 | 0.1956 | 0.0484 | 0.9921 | 0.219 | 0.9654 | 0.0777 | 0.8573 | 0.51537 | low |
| TCGA-BP-5181 | 0.0581 | 0.0518 | 0.9949 | 0.0511 | 0.9895 | 0.1909 | 0.8286 | 0.370273 | low |
| TCGA-B0-5709 | 0.1952 | 0.055 | 0.9865 | 0.1856 | 0.9899 | 0.1085 | 0.6819 | 0.620437 | low |
| TCGA-BP-4167 | 0.2552 | 0.08116 | 0.9832 | 0.38169 | 0.9605 | 0.274 | 0.62687 | 1.250535 | high |
| TCGA-CJ-4638 | 0.312 | 0.4418 | 0.9341 | 1 | 0.996 | 0.58658 | 0.7393 | 5.717438 | high |
| TCGA-EU-5907 | 0.3881 | 0.1876 | 0.9892 | 0.3557 | 0.9718 | 0.2038 | 0.6408 | 1.540711 | high |
| TCGA-CJ-4869 | 0.2218 | 0 | 0.9874 | 0.2664 | 0.992 | 0.0631 | 0.6241 | 0.641504 | low |
| TCGA-3Z-A93Z | 0.1849 | 0.0503 | 0.9915 | 0.3557 | 0.9659 | 0.20293 | 0.7724 | 0.725595 | low |
| TCGA-BP-5196 | 0.2357 | 0.0211 | 0.989 | 0.139 | 0.9716 | 0.2393 | 0.2344 | 1.383995 | high |
| TCGA-AK-3433 | 0.19 | 0 | 0.9912 | 0.4365 | 0.9928 | 0.1359 | 0.9584 | 0.454835 | low |
| TCGA-A3-3346 | 0.241 | 0.0843 | 0.9815 | 0.6679 | 0.9014 | 0.3032 | 0.5889 | 2.225627 | high |
| TCGA-AK-3440 | 0.1146 | 0.0115 | 0.9915 | 0.27349 | 0.9953 | 0.4855 | 0.8578 | 0.579191 | low |
| TCGA-B8-A54D | 0.221 | 0.2939 | 0.9711 | 0.818 | 0.965 | 0.4464 | 0.8705 | 2.169369 | high |
| TCGA-BP-5200 | 0.1463 | 0.0402 | 0.9805 | 0.0781 | 0.9153 | 0.1339 | 0.7696 | 0.676075 | low |
| TCGA-B0-5400 | 0.1691 | 0.2289 | 0.9584 | 1 | 0.9928 | 0.3206 | 0.7219 | 2.130544 | high |
| TCGA-BP-4969 | 0.0726 | 0.2163 | 1 | 0.5666 | 0.9936 | 0.4402 | 0.9197 | 0.823047 | low |
| TCGA-BP-4756 | 0.1488 | 0.0782 | 0.9885 | 0.6221 | 0.9865 | 0.1219 | 0.8902 | 0.643972 | low |
| TCGA-BP-4776 | 0.1563 | 0.189 | 0.9664 | 0.3922 | 0.9799 | 0.3414 | 0.7918 | 1.11279 | high |
| TCGA-CJ-4912 | 0.4568 | 0.2223 | 0.9441 | 0.0787 | 0.9213 | 0.4357 | 0.5919 | 3.29485 | high |
| TCGA-CZ-5985 | 0.3605 | 0.0396 | 0.9867 | 0.2251 | 0.9802 | 0.1359 | 0.7246 | 0.827298 | low |
| TCGA-B0-5098 | 0.4645 | 0.3191 | 0.9707 | 0.9356 | 0.9245 | 0.51 | 0.53543 | 7.707402 | high |
| TCGA-B8-5545 | 0.1705 | 0.0328 | 1 | 0.0911 | 0.9699 | 0.0631 | 0.6137 | 0.563637 | low |
| TCGA-BP-4758 | 0.1126 | 0.1556 | 1 | 0.2167 | 0.9746 | 0.3615 | 0.7864 | 0.726078 | low |
| TCGA-B0-4696 | 0.6533 | 0.2446 | 0.9899 | 0.39967 | 0.8091 | 0.4026 | 0.1641 | 13.74729 | high |
| TCGA-A3-3322 | 0.1043 | 0.1189 | 0.9916 | 0.3262 | 0.9874 | 0.3657 | 0.8449 | 0.672826 | low |
| TCGA-B0-5107 | 0.402 | 0.0691 | 0.9882 | 1 | 0.9873 | 0.6536 | 0.3603 | 4.687755 | high |
| TCGA-DV-5565 | 0.1851 | 0.0808 | 0.9888 | 0.0826 | 0.9647 | 0.2798 | 0.4889 | 1.014018 | high |
| TCGA-CZ-5989 | 0.1814 | 0.1821 | 1 | 0.273433 | 1 | 0.274 | 0.5181 | 1.064989 | high |
| TCGA-BP-4173 | 0.4539 | 0.1367 | 0.9836 | 0.3675 | 0.965 | 0.4233 | 0.4585 | 2.726119 | high |
| TCGA-CJ-4870 | 0.1234 | 0.0885 | 0.9934 | 0.1016 | 0.9729 | 0.235 | 0.8883 | 0.479245 | low |
| TCGA-BP-4974 | 0.3651 | 0.1935 | 0.9467 | 0.2766 | 0.9854 | 0.4263 | 0.5403 | 2.468337 | high |
| TCGA-B0-5075 | 0.1593 | 0.1578 | 0.9758 | 0.6932 | 0.9813 | 0.3564 | 0.7243 | 1.412712 | high |
| TCGA-BP-4162 | 0.2447 | 0.0675 | 0.9925 | 0.3456 | 0.9973 | 0.2968 | 0.823 | 0.720472 | low |
| TCGA-B0-4839 | 0.1384 | 0.0733 | 1 | 0.1537 | 0.9892 | 0.1359 | 0.8279 | 0.43873 | low |
| TCGA-B8-5553 | 0.195 | 0.0337 | 0.9848 | 0.1115 | 0.9872 | 0.0599 | 0.7724 | 0.482486 | low |
| TCGA-CJ-4893 | 0.2053 | 0.0837 | 0.9907 | 0.094 | 0.9827 | 0.1297 | 0.827 | 0.516327 | low |
| TCGA-CJ-6033 | 0.1816 | 0.0172 | 0.9858 | 0.0747 | 0.9741 | 0.0862 | 0.459 | 0.75157 | low |
| TCGA-B0-4827 | 0.0999 | 0.0675 | 0.9818 | 0.0916 | 0.9861 | 0.1324 | 0.7406 | 0.495802 | low |
| TCGA-BP-4977 | 0.2077 | 0.0257 | 0.9897 | 0.4269 | 0.987 | 0.2353 | 0.9042 | 0.600884 | low |
| TCGA-BP-5187 | 0.3517 | 0.1028 | 0.9841 | 0.1623 | 0.9953 | 0.2922 | 0.6363 | 1.088216 | high |
| TCGA-CJ-4882 | 0.3779 | 0.0588 | 0.9504 | 0.6704 | 0.9666 | 0.2483 | 0.5247 | 2.489307 | high |
| TCGA-CJ-4899 | 0.1917 | 0.0627 | 0.9671 | 0.2007 | 0.9788 | 0.1771 | 0.825 | 0.655791 | low |
| TCGA-B8-4620 | 0.4492 | 0.1061 | 0.9809 | 0.108 | 0.9079 | 0.2939 | 0.2984 | 3.028866 | high |
| TCGA-CJ-4868 | 0.4249 | 0.0857 | 0.9754 | 0.139 | 0.938 | 0.2541 | 0.3828 | 2.211461 | high |
| TCGA-BP-5001 | 0.0628 | 0.3893 | 0.9807 | 0.6094 | 0.9625 | 0.586 | 0.9435 | 1.651393 | high |
| TCGA-DV-5575 | 0.1841 | 0.0424 | 0.9962 | 0.0647 | 0.9947 | 0.1909 | 0.6651 | 0.553765 | low |
| TCGA-CJ-4920 | 0.1634 | 0.1341 | 0.9851 | 0.2933 | 0.9872 | 0.2393 | 0.6542 | 0.893199 | high |
| TCGA-B0-4707 | 0.7094 | 0.2572 | 0.9919 | 1 | 0.9036 | 0.8251 | 0.7159 | 11.16519 | high |
| TCGA-AK-3426 | 0.2489 | 0.4259 | 0.9085 | 0.8878 | 0.9484 | 0.4712 | 0.61199 | 7.109978 | high |
| TCGA-B0-4713 | 0.1235 | 0.1512 | 0.9414 | 1 | 0.9959 | 0.36236 | 0.8302 | 1.685307 | high |
| TCGA-B0-5705 | 0.0703 | 0.0437 | 0.9973 | 0.1215 | 0.9846 | 0.1481 | 0.8828 | 0.352505 | low |
| TCGA-BP-5199 | 0.2117 | 0.1765 | 0.9778 | 0.361 | 0.9395 | 0.5174 | 0.4853 | 2.400661 | high |
| TCGA-CZ-5988 | 0.227 | 0.0167 | 1 | 0.108 | 0.9959 | 0.3206 | 0.7089 | 0.611114 | low |
| TCGA-BP-5004 | 0.0952 | 0.056 | 0.9949 | 0.3151 | 0.9797 | 0.2047 | 0.9424 | 0.442703 | low |
| TCGA-A3-3370 | 0.1025 | 0.0849 | 0.9968 | 0.2745 | 0.9854 | 0.201 | 0.9058 | 0.461173 | low |
| TCGA-B0-5696 | 0.249 | 0.0241 | 0.9874 | 0.2498 | 0.9788 | 0.2158 | 0.7214 | 0.744375 | low |
| TCGA-BP-4797 | 0.2247 | 0 | 0.9917 | 0.139 | 0.9472 | 0.0763 | 0.748 | 0.59129 | low |
| TCGA-CZ-5982 | 0.1956 | 0.0403 | 0.9868 | 0.1258 | 0.9926 | 0.2704 | 0.8347 | 0.537727 | low |
| TCGA-BP-4991 | 0.1505 | 0.0953 | 0.9873 | 0.2766 | 0.9767 | 0.2355 | 0.7747 | 0.700279 | low |
| TCGA-BP-4762 | 0.2097 | 0.0498 | 0.9841 | 0.0832 | 0.9882 | 0.0748 | 0.6452 | 0.604095 | low |
| TCGA-A3-3362 | 0.0354 | 0.1393 | 0.9765 | 0.2498 | 0.9968 | 0.2798 | 0.8568 | 0.555274 | low |
| TCGA-A3-A8OW | 0.1773 | 0.0692 | 0.9953 | 0.1813 | 0.9693 | 0.2525 | 0.8816 | 0.557293 | low |
| TCGA-CJ-4872 | 0.2634 | 0.0757 | 0.9841 | 0.0904 | 0.9535 | 0.1219 | 0.5506 | 0.976422 | high |
| TCGA-B0-4847 | 0.3395 | 0.2028 | 0.9591 | 0.673 | 0.9929 | 0.6228 | 0.8385 | 2.361676 | high |
| TCGA-B0-4822 | 0.2923 | 0.3377 | 0.9426 | 0.5175 | 0.8716 | 0.6619 | 0.4154 | 8.933795 | high |
| TCGA-BP-5175 | 0.199 | 0.1094 | 1 | 0.7177 | 0.9178 | 0.3776 | 0.8611 | 1.369852 | high |
| TCGA-BP-4340 | 0.0994 | 0.1517 | 0.9919 | 0.3151 | 0.987 | 0.409 | 0.9467 | 0.633493 | low |
| TCGA-BP-4987 | 0.1449 | 0.1512 | 0.9941 | 0.2323 | 0.9888 | 0.1528 | 0.8108 | 0.586825 | low |
| TCGA-BP-5189 | 0.3258 | 0.0983 | 0.9908 | 0.0626 | 0.9588 | 0.1427 | 0.5559 | 1.045631 | high |
| TCGA-BP-5000 | 0.2357 | 0.1652 | 1 | 0.2141 | 0.9707 | 0.0999 | 0.6684 | 0.837726 | low |
| TCGA-AK-3451 | 0.1309 | 0.03344 | 1 | 0 | 0.985 | 0.19609 | 0.6691 | 0.483696 | low |
| TCGA-BP-5007 | 0.1882 | 0.1253 | 0.9749 | 0.1809 | 0.9954 | 0.2777 | 0.8814 | 0.646172 | low |
| TCGA-A3-3363 | 0.0774 | 0.1253 | 0.9846 | 0.3262 | 1 | 0.2798 | 0.5813 | 0.855502 | high |
| TCGA-AK-3455 | 0.2055 | 0.0381 | 1 | 0.404 | 0.9941 | 0.3206 | 0.8746 | 0.619765 | low |
| TCGA-B8-4146 | 0.1068 | 0.1544 | 0.9749 | 0.17235 | 1 | 0.4402 | 0.9074 | 0.655183 | low |
| TCGA-BP-4770 | 0.5766 | 0.1425 | 1 | 0.0883 | 0.7946 | 0.5136 | 0.0709 | 10.07261 | high |
| TCGA-A3-3378 | 0.2398 | 0.1972 | 0.9752 | 0.5923 | 0.9875 | 0.2136 | 0.4923 | 1.875539 | high |
| TCGA-DV-5573 | 0.2914 | 0.0393 | 0.9817 | 0.492 | 0.9944 | 0.1682 | 0.6926 | 0.954818 | high |
| TCGA-BP-4962 | 0.2281 | 0.0215 | 0.9916 | 0.0883 | 0.956 | 0.206 | 0.696 | 0.697996 | low |
| TCGA-B8-5546 | 0.1403 | 0.0588 | 1 | 0.18467 | 1 | 0.0752 | 0.287 | 0.848103 | high |
| TCGA-BP-4347 | 0.2711 | 0.1393 | 0.9841 | 0.1552 | 0.98 | 0.1941 | 0.8793 | 0.696399 | low |
| TCGA-A3-3343 | 0.0835 | 0.1199 | 1 | 0.1804 | 0.995 | 0.586 | 0.8879 | 0.622308 | low |
| TCGA-CJ-4918 | 0.2685 | 0.0714 | 0.9778 | 0.0665 | 0.9689 | 0.1277 | 0.4477 | 1.080573 | high |
| TCGA-BP-5201 | 0.3483 | 0.0795 | 0.9955 | 0.3675 | 0.9815 | 0.358 | 0.7019 | 1.183748 | high |
| TCGA-CZ-5459 | 0.1409 | 0.0322 | 0.969 | 0.1288 | 0.9864 | 0.2693 | 0.7341 | 0.64175 | low |
| TCGA-BP-5170 | 0.3563 | 0.0215 | 0.9801 | 0.1949 | 0.9852 | 0.4638 | 0.2998 | 2.000049 | high |
| TCGA-B0-5094 | 0.3796 | 0.1038 | 0.9823 | 0.7385 | 0.9583 | 0.2172 | 0.6693 | 1.925997 | high |
| TCGA-BP-4973 | 0.1289 | 0.1149 | 0.9899 | 0.2269 | 0.9891 | 0.3569 | 0.7771 | 0.702816 | low |
| TCGA-CJ-5684 | 0.1444 | 0.0825 | 0.9954 | 0.1268 | 0.9886 | 0.1804 | 0.9006 | 0.428659 | low |
| TCGA-BP-4771 | 0.3141 | 0.1695 | 0.9821 | 0.318 | 0.9762 | 0.3206 | 0.466 | 1.887044 | high |
| TCGA-B0-5116 | 0.33 | 0.0232 | 0.981 | 0.1268 | 0.9867 | 0.0557 | 0.4765 | 0.931335 | high |
| TCGA-CW-5585 | 0.0622 | 0.0577 | 0.9929 | 0.1233 | 0.9942 | 0.1321 | 0.9621 | 0.308779 | low |
| TCGA-CJ-4881 | 0.3664 | 0.1821 | 0.9857 | 0.5784 | 0.9699 | 0.257 | 0.6782 | 1.815066 | high |
| TCGA-A3-3323 | 0.2044 | 0.1121 | 1 | 0.3397 | 0.9452 | 0.3657 | 0.707 | 1.110826 | high |
| TCGA-A3-3380 | 0.1291 | 0.0916 | 0.9964 | 0.4413 | 0.9907 | 0.3417 | 0.934 | 0.603148 | low |
| TCGA-B0-4834 | 0.0389 | 0.1821 | 1 | 0.0883 | 0.993 | 0.2158 | 0.7939 | 0.477896 | low |
| TCGA-AK-3450 | 0.1705 | 0.1652 | 0.993 | 0.1537 | 0.9722 | 0.1804 | 0.907 | 0.573868 | low |
| TCGA-CZ-5461 | 0.3302 | 0.0603 | 0.9754 | 0.0422 | 0.798 | 0.074 | 0.6063 | 1.933511 | high |
| TCGA-A3-3347 | 0.3502 | 0.0719 | 0.9762 | 0.7948 | 0.9621 | 0.2663 | 0.5701 | 2.237389 | high |
| TCGA-B0-5110 | 0.1647 | 0.1118 | 1 | 0.2766 | 0.9929 | 0.3002 | 0.7515 | 0.698707 | low |
| TCGA-AK-3460 | 0.1463 | 0.11 | 1 | 0.2323 | 0.9908 | 0.2393 | 0.7084 | 0.658553 | low |
| TCGA-CJ-4908 | 0.3672 | 0.1042 | 0.9845 | 0.1523 | 0.9748 | 0.1452 | 0.5738 | 1.148259 | high |
| TCGA-BP-4774 | 0.1407 | 0.1292 | 0.9641 | 0.3262 | 0.9758 | 0.218 | 0.8413 | 0.78833 | low |
| TCGA-B0-4842 | 0.1298 | 0.09407 | 1 | 0.38299 | 0.9646 | 0.35865 | 0.6341 | 0.992286 | high |
| TCGA-B0-5812 | 0.1917 | 0.0828 | 0.994 | 0.0511 | 0.9777 | 0.1118 | 0.7506 | 0.534436 | low |
| TCGA-B0-4816 | 0.3802 | 0.1313 | 0.9778 | 0.1827 | 0.9847 | 0.3484 | 0.6186 | 1.44295 | high |
| TCGA-BP-4988 | 0.3635 | 0.4057 | 0.954 | 0.6241 | 0.9971 | 0.6479 | 0.2653 | 7.906494 | high |
| TCGA-B0-4945 | 0.3018 | 0.1891 | 0.9786 | 0.2949 | 0.9965 | 0.5006 | 0.7179 | 1.464403 | high |
| TCGA-DV-5576 | 0.227 | 0.1578 | 0.9883 | 0.204 | 1 | 0.1804 | 0.5493 | 0.974269 | high |
| TCGA-MM-A84U | 0.0882 | 0.1817 | 0.9797 | 0.6883 | 0.955 | 0.5589 | 0.4459 | 2.608741 | high |
| TCGA-BP-4998 | 0.2999 | 0.0683 | 0.9889 | 0.467 | 0.9754 | 0.2644 | 0.5729 | 1.359501 | high |
| TCGA-A3-3387 | 0.1847 | 0.0588 | 0.9629 | 0.2266 | 0.9915 | 0.1055 | 0.4951 | 0.945888 | high |
| TCGA-BP-4968 | 0.1727 | 0.0974 | 0.9911 | 0.3262 | 0.9877 | 0.409 | 0.9156 | 0.686179 | low |
| TCGA-BP-4995 | 0.1497 | 0.0308 | 0.9976 | 0.2108 | 0.9929 | 0.1382 | 0.8541 | 0.418373 | low |
| TCGA-CJ-4890 | 0.1958 | 0.0928 | 0.9925 | 0.2792 | 0.965 | 0.2636 | 0.3533 | 1.437739 | high |
| TCGA-B0-5092 | 0.3567 | 0.2626 | 0.9744 | 0.601 | 0.9691 | 0.4026 | 0.5791 | 3.00113 | high |
| TCGA-DV-A4VZ | 0.4853 | 0.21461 | 0.9826 | 0.6755 | 1 | 0.5698 | 0.5085 | 3.901411 | high |
| TCGA-A3-3385 | 0.0928 | 0.1494 | 0.9921 | 0.5305 | 0.9828 | 0.3206 | 0.9143 | 0.724622 | low |
| TCGA-B0-4823 | 0.1637 | 0.0757 | 0.9946 | 0.1167 | 0.9903 | 0.2521 | 0.8375 | 0.507921 | low |
| TCGA-BP-4992 | 0.2765 | 0.3372 | 0.9776 | 1 | 0.9522 | 0.8222 | 0.5216 | 7.217066 | high |
| TCGA-B8-4151 | 0.0293 | 0.1354 | 1 | 0.2511 | 0.9858 | 0.1733 | 0.9774 | 0.377167 | low |
| TCGA-B4-5838 | 0.2838 | 0 | 0.9895 | 0.15991 | 0.982 | 0.111 | 0.8261 | 0.531045 | low |
| TCGA-BP-4986 | 0.445 | 0.056 | 0.9888 | 0.2695 | 0.9798 | 0.1458 | 0.4124 | 1.577811 | high |
| TCGA-CZ-5984 | 0.3849 | 0.0426 | 0.9798 | 0.2068 | 0.9981 | 0.2358 | 0.6158 | 1.056893 | high |
| TCGA-BP-4344 | 0.2235 | 0.1258 | 0.9916 | 0.1449 | 0.9807 | 0.226 | 0.3991 | 1.209673 | high |
| TCGA-CZ-4854 | 0.3089 | 0.0803 | 0.9802 | 0.4626 | 0.9576 | 0.1953 | 0.664 | 1.318776 | high |
| TCGA-CJ-6032 | 0.1139 | 0.0648 | 0.9926 | 0.1552 | 0.9772 | 0.1846 | 0.8872 | 0.443366 | low |
| TCGA-BP-4355 | 0.4205 | 0.3389 | 0.9583 | 0.2489 | 0.9973 | 0.444 | 0.7218 | 2.373816 | high |
| TCGA-B0-4814 | 0.2185 | 0.2339 | 0.9658 | 0.2736 | 0.9561 | 0.2614 | 0.7787 | 1.277796 | high |
| TCGA-BP-4759 | 0.219 | 0.0619 | 1 | 0.1441 | 0.9758 | 0.0693 | 0.7372 | 0.5516 | low |
| TCGA-B0-5097 | 0.2902 | 0.0358 | 0.964 | 0.1537 | 0.9677 | 0.2521 | 0.5909 | 1.140837 | high |
| TCGA-CJ-4923 | 0.5615 | 0.153 | 0.9665 | 0.5784 | 0.9479 | 0.304 | 0.4197 | 4.476725 | high |
| TCGA-DV-A4VX | 0.1725 | 0.3682 | 0.9798 | 1 | 0.9753 | 0.6282 | 0.8706 | 2.86448 | high |
| TCGA-CJ-5671 | 0.2248 | 0.0818 | 0.9532 | 0.0844 | 0.9521 | 0.1558 | 0.5428 | 1.172613 | high |
| TCGA-BP-5191 | 0.2243 | 0.2706 | 1 | 0.5371 | 0.9576 | 0.3861 | 0.6443 | 1.876651 | high |
| TCGA-BP-4158 | 0.2407 | 0.1794 | 0.9936 | 0.212 | 0.9979 | 0.3206 | 0.8536 | 0.760771 | low |
| TCGA-BP-4768 | 0.4456 | 0.0679 | 0.9829 | 0.404 | 0.9808 | 0.2843 | 0.7697 | 1.283412 | high |
| TCGA-B2-5639 | 0.1446 | 0.1024 | 0.9879 | 0.492 | 1 | 0.1733 | 0.7909 | 0.687102 | low |
| TCGA-B0-5084 | 0.5497 | 0.1367 | 1 | 0.8288 | 0.8733 | 0.6768 | 0.1743 | 12.26715 | high |
| TCGA-CJ-5680 | 0.1083 | 0.1182 | 0.9682 | 0.1771 | 0.9922 | 0.101 | 0.703 | 0.636594 | low |
| TCGA-CJ-4639 | 0.1114 | 0.0578 | 0.9922 | 0.5071 | 0.9902 | 0.206 | 0.8803 | 0.564115 | low |
| TCGA-B8-5159 | 0.1903 | 0.1128 | 0.9887 | 0.1856 | 0.9905 | 0.1077 | 0.8796 | 0.50637 | low |
| TCGA-CJ-4641 | 0.2159 | 0.056 | 0.9762 | 0.4116 | 0.9925 | 0.3034 | 0.6861 | 0.980211 | high |
| TCGA-B0-5692 | 0.2905 | 0.0849 | 0.9883 | 0.1474 | 0.9953 | 0.2123 | 0.8265 | 0.647194 | low |
| TCGA-CJ-4895 | 0.3635 | 0.1351 | 0.9882 | 0.1949 | 0.9552 | 0.0982 | 0.3593 | 1.725087 | high |
| TCGA-BP-4959 | 0.083 | 0.0547 | 1 | 0.0809 | 0.9859 | 0.11 | 0.8789 | 0.336258 | low |
| TCGA-BP-4787 | 0.2375 | 0.1544 | 0.9749 | 0.4777 | 0.9843 | 0.3369 | 0.5228 | 1.738742 | high |
| TCGA-AK-3465 | 0.0526 | 0.0337 | 1 | 0.5476 | 1 | 0.2663 | 0.8686 | 0.498658 | low |
| TCGA-BP-5009 | 0.315 | 0.2563 | 0.971 | 0.5375 | 0.9658 | 0.2347 | 0.3438 | 3.222393 | high |
| TCGA-EU-5904 | 0.1333 | 0.0503 | 0.9906 | 0.1642 | 0.9866 | 0.2008 | 0.834 | 0.477629 | low |
| TCGA-A3-3306 | 0.4353 | 0.0711 | 0.9843 | 0.3837 | 0.9726 | 0.3978 | 0.7283 | 1.525621 | high |
| TCGA-BP-5192 | 0.0692 | 0.0252 | 0.9912 | 0.146 | 0.9978 | 0.1682 | 0.9196 | 0.328699 | low |
| TCGA-BP-4784 | 0.4005 | 0 | 1 | 0.1199 | 0.9891 | 0.0999 | 0.4989 | 0.889957 | high |
| TCGA-B8-4153 | 0.0577 | 0.0619 | 1 | 0.2906 | 0.9872 | 0.2123 | 0.9213 | 0.400786 | low |
| TCGA-B0-5081 | 0.2762 | 0.0936 | 0.9795 | 0.4614 | 0.9825 | 0.3503 | 0.5049 | 1.674028 | high |
| TCGA-G6-A8L6 | 0.2985 | 0.2798 | 1 | 0.5867 | 0.9453 | 0.36076 | 0.5373 | 2.69409 | high |
| TCGA-AK-3445 | 0.2651 | 0.0512 | 0.9373 | 0.5305 | 0.9625 | 0.2309 | 0.5728 | 1.846351 | high |
| TCGA-BP-4351 | 0.4037 | 0.3961 | 0.9838 | 0.6397 | 0.9461 | 0.5055 | 0.7835 | 3.684572 | high |
| TCGA-B0-5100 | 0.3858 | 0.6091 | 1 | 0.5375 | 0.9866 | 0.3802 | 0.3852 | 5.604594 | high |
| TCGA-B0-5700 | 0.1051 | 0.0407 | 0.9913 | 0.2167 | 0.9653 | 0.2277 | 0.8678 | 0.502521 | low |
| TCGA-AK-3429 | 0.1805 | 0 | 1 | 0.0571 | 0.9765 | 0.0592 | 0.9006 | 0.338628 | low |
| TCGA-BP-4798 | 0.3184 | 0.2404 | 0.9873 | 0.6932 | 0.9658 | 0.3453 | 0.5287 | 2.775913 | high |
| TCGA-BP-4769 | 0.3723 | 0.0471 | 0.9884 | 0.3526 | 1 | 0.0481 | 0.4369 | 1.179463 | high |
| TCGA-CJ-5678 | 0.2257 | 0.06338 | 0.9484 | 0.1949 | 0.99 | 0.0557 | 0.9406 | 0.554159 | low |
| TCGA-A3-3316 | 0.4926 | 0.0857 | 0.9883 | 0.2906 | 0.9647 | 0.2704 | 0.4718 | 2.046747 | high |
| TCGA-B8-5550 | 0.1034 | 0.09 | 0.9748 | 0.139 | 0.9944 | 0.214 | 0.7443 | 0.584557 | low |
| TCGA-B0-5699 | 0.0855 | 0.0198 | 0.9877 | 0.1552 | 0.988 | 0.1643 | 0.9255 | 0.355693 | low |
| TCGA-CJ-4888 | 0.2573 | 0.2433 | 0.9938 | 0.1719 | 0.9601 | 0.2555 | 0.3863 | 1.829357 | high |
| TCGA-BP-4354 | 0.3941 | 0.0857 | 0.9415 | 0.5755 | 0.9914 | 0.288 | 0.1079 | 4.398174 | high |
| TCGA-BP-4989 | 0.2406 | 0.1578 | 0.978 | 0.7017 | 0.9954 | 0.2498 | 0.5031 | 1.851559 | high |
| TCGA-CJ-4873 | 0.2606 | 0.1292 | 1 | 0.6832 | 0.9928 | 0.3861 | 0.3449 | 2.27121 | high |
| TCGA-B0-4824 | 0.1189 | 0.1784 | 0.9894 | 0.3584 | 0.9939 | 0.7905 | 0.8591 | 1.156512 | high |
| TCGA-CZ-5455 | 0.1954 | 0.0266 | 0.9821 | 0.1771 | 0.9932 | 0.1407 | 0.786 | 0.528553 | low |
| TCGA-BP-4164 | 0.1107 | 0.216 | 0.9885 | 0.3997 | 0.9883 | 0.3105 | 0.88 | 0.782981 | low |
| TCGA-CW-5581 | 0.1631 | 0.0549 | 0.9832 | 0.1552 | 0.9851 | 0.0918 | 0.8299 | 0.477515 | low |
| TCGA-A3-3313 | 0.0564 | 0.1351 | 1 | 0.9142 | 1 | 0.296 | 0.8368 | 0.868923 | high |
| TCGA-B0-4845 | 0.2782 | 0.2413 | 0.9815 | 0.1623 | 0.9618 | 0.3369 | 0.8026 | 1.204476 | high |
| TCGA-B4-5378 | 0.4703 | 0.0252 | 0.9956 | 0.2136 | 1 | 0.1588 | 0.4501 | 1.250654 | high |
| TCGA-BP-5174 | 0.1218 | 0.054 | 0.9758 | 0.3262 | 0.961 | 0.1588 | 0.9013 | 0.576935 | low |
| TCGA-BP-4801 | 0.1034 | 0.0709 | 1 | 0.1789 | 0.9779 | 0.1251 | 0.8334 | 0.434973 | low |
| TCGA-CJ-5686 | 0.1911 | 0.1393 | 0.9811 | 0.2023 | 0.9737 | 0.2393 | 0.7435 | 0.842465 | high |
| TCGA-A3-3349 | 0.1205 | 0.0808 | 0.9945 | 0.1996 | 0.9948 | 0.3206 | 0.7694 | 0.589568 | low |
| TCGA-DV-5567 | 0.2168 | 0.0275 | 0.9935 | 0.0952 | 0.9904 | 0.121 | 0.6587 | 0.569508 | low |
| TCGA-CZ-5987 | 0.3179 | 0 | 0.9774 | 0.25462 | 0.9924 | 0.3206 | 0.7707 | 0.829882 | low |
| TCGA-CJ-4885 | 0.1289 | 0.2316 | 1 | 0.1497 | 0.9838 | 0.079 | 0.841 | 0.541474 | low |
| TCGA-DV-5574 | 0.1517 | 0.0912 | 0.9918 | 0.0986 | 0.9968 | 0.0677 | 0.3728 | 0.800914 | low |
| TCGA-A3-3331 | 0.0808 | 0.0566 | 0.9934 | 0.3361 | 0.9751 | 0.1268 | 0.8514 | 0.477596 | low |
| TCGA-BP-5178 | 0.1217 | 0.0678 | 0.9776 | 0.3262 | 0.9734 | 0.2243 | 0.754 | 0.726376 | low |
| TCGA-CJ-4871 | 0.1822 | 0.0347 | 0.9906 | 0.1041 | 0.9512 | 0.0862 | 0.6291 | 0.67323 | low |
| TCGA-B0-4836 | 0.4386 | 0.11 | 0.9918 | 0.139 | 0.9637 | 0.1841 | 0.5515 | 1.393924 | high |
| TCGA-B8-A54I | 0.1644 | 0.1044 | 0.9674 | 0.5965 | 0.9536 | 0.5802 | 0.3542 | 3.026323 | high |
| TCGA-B0-4821 | 0.6614 | 0.3553 | 0.9744 | 0.9237 | 0.9045 | 0.5182 | 0.6139 | 10.88992 | high |
| TCGA-BP-4777 | 0.1891 | 0.1565 | 1 | 0.4735 | 0.9946 | 0.0982 | 0.8108 | 0.688309 | low |
| TCGA-CJ-4636 | 0.1568 | 0.1205 | 0.9625 | 0.361 | 0.9908 | 0.586 | 0.6337 | 1.496377 | high |
| TCGA-B0-5711 | 0.1924 | 0.0117 | 0.9922 | 0.0434 | 0.9843 | 0.0729 | 0.725 | 0.458925 | low |
| TCGA-CZ-4857 | 0.326 | 0.0619 | 0.9847 | 0.3262 | 0.9857 | 0.2992 | 0.6952 | 1.067709 | high |
| TCGA-B0-4852 | 0.2583 | 0.1139 | 0.9801 | 0.1891 | 0.9754 | 0.4352 | 0.7661 | 1.046486 | high |
| TCGA-A3-3329 | 0.0848 | 0.1044 | 0.9767 | 0.244 | 0.9727 | 0.1733 | 0.9313 | 0.50892 | low |
| TCGA-CZ-5466 | 0.1851 | 0.0445 | 0.9633 | 0.19129 | 0.968 | 0.1944 | 0.7394 | 0.770084 | low |
| TCGA-BP-5177 | 0.1779 | 0.1035 | 0.9775 | 0.1326 | 0.9837 | 0.0592 | 0.8111 | 0.542597 | low |
| TCGA-A3-3372 | 0.1948 | 0.0612 | 0.987 | 0.3262 | 0.9876 | 0.1797 | 0.7448 | 0.694456 | low |
| TCGA-BP-4804 | 0.3517 | 0.1061 | 0.9906 | 0.139 | 0.9713 | 0.1104 | 0.427 | 1.293366 | high |
| TCGA-AK-3434 | 0.2602 | 0.0803 | 1 | 0.1835 | 0.9755 | 0.274 | 0.8013 | 0.705883 | low |
| TCGA-B0-4811 | 0.2021 | 0.3899 | 1 | 0.8582 | 0.9823 | 0.8251 | 0.7566 | 3.410773 | high |
| TCGA-BP-4165 | 0.183 | 0.248 | 0.9895 | 0.2604 | 0.984 | 0.3861 | 0.9096 | 0.874673 | high |
| TCGA-B0-5099 | 0.2619 | 0.1932 | 0.9878 | 0.2103 | 0.9711 | 0.4276 | 0.8842 | 1.006124 | high |
| TCGA-BP-4766 | 0.096 | 0.1802 | 0.9909 | 0.3131 | 0.9803 | 0.3124 | 0.8523 | 0.712544 | low |
| TCGA-BP-4335 | 0.2576 | 0.2662 | 0.9803 | 0.6221 | 0.9839 | 0.5721 | 0.7946 | 2.050062 | high |
| TCGA-B0-4703 | 0.7825 | 0.1024 | 0.953 | 0.1585 | 0.977 | 0.2277 | 0.59255 | 2.901875 | high |
| TCGA-CJ-4904 | 0.1969 | 0.0498 | 0.991 | 0.108 | 0.9783 | 0.0927 | 0.8323 | 0.472565 | low |
| TCGA-BP-4352 | 0.5286 | 0.6128 | 0.8454 | 1 | 0.9576 | 0.752 | 0.4208 | 42.10896 | high |
| TCGA-CJ-4635 | 0.2726 | 0.2495 | 1 | 0.4321 | 0.9671 | 0.274 | 0.922 | 1.039618 | high |
| TCGA-CJ-5679 | 0.3179 | 0.1143 | 0.965 | 0.27644 | 1 | 0.1251 | 0.7447 | 0.9201 | high |
| TCGA-CZ-4862 | 0.2229 | 0.0724 | 1 | 0.4587 | 0.9347 | 0.1989 | 0.8261 | 0.882144 | high |
| TCGA-B8-4622 | 0.2221 | 0.1652 | 0.9863 | 0.2149 | 0.9829 | 0.3067 | 0.8227 | 0.832379 | low |
| TCGA-G6-A8L8 | 0.0773 | 0.2057 | 0.9895 | 0.2358 | 0.9744 | 0.5513 | 0.9056 | 0.827683 | low |
| TCGA-CJ-5682 | 0.2458 | 0.1367 | 0.9843 | 0.0809 | 0.9867 | 0.1285 | 0.7703 | 0.663117 | low |
| TCGA-BP-4176 | 0.298 | 0.1607 | 0.9862 | 0.0925 | 0.9808 | 0.1359 | 0.6971 | 0.863886 | high |
| TCGA-B8-4148 | 0.167 | 0.0922 | 0.9874 | 0.3023 | 0.9789 | 0.4402 | 0.7869 | 0.871083 | high |
| TCGA-CJ-4886 | 0.1302 | 0.0702 | 1 | 0.2078 | 0.9834 | 0.2714 | 0.7558 | 0.585238 | low |
| TCGA-BP-5186 | 0.0556 | 0.0732 | 1 | 0.2619 | 0.9951 | 0.2774 | 0.9404 | 0.398767 | low |
| TCGA-BP-4972 | 0.1067 | 0.0798 | 0.9928 | 0.1537 | 0.9988 | 0.1879 | 0.8289 | 0.443002 | low |
| TCGA-BP-4993 | 0.2104 | 0.0704 | 0.9878 | 0.1679 | 0.9586 | 0.1503 | 0.8402 | 0.614692 | low |
| TCGA-CW-6097 | 0.3252 | 0.0319 | 0.9859 | 0.0294 | 0.9858 | 0.1822 | 0.2353 | 1.349376 | high |
| TCGA-B0-5077 | 0.2465 | 0.1535 | 0.9833 | 0.2792 | 0.9936 | 0.2704 | 0.8323 | 0.825016 | low |
| TCGA-A3-3383 | 0.1436 | 0 | 1 | 0.3212 | 0.9769 | 0.5181 | 0.9174 | 0.606496 | low |
| TCGA-B0-4819 | 0.2089 | 0.471 | 1 | 0.5743 | 0.9803 | 0.5572 | 0.1871 | 5.48764 | high |
| TCGA-BP-4775 | 0.1568 | 0.1133 | 1 | 0.3795 | 0.9341 | 0.3082 | 0.9334 | 0.76594 | low |
| TCGA-BP-4327 | 0.2044 | 0.1919 | 1 | 0.3795 | 0.9844 | 0.3206 | 0.8145 | 0.906175 | high |
| TCGA-B0-5399 | 0.2142 | 0.11 | 0.9946 | 0.2017 | 0.9797 | 0.3439 | 0.908 | 0.653841 | low |
| TCGA-A3-3357 | 0.1247 | 0.0772 | 0.9931 | 0.3863 | 0.9977 | 0.3019 | 0.9036 | 0.553813 | low |
| TCGA-B0-4694 | 0.26 | 0.1205 | 0.9871 | 0.1949 | 0.9619 | 0.1588 | 0.7238 | 0.876558 | high |
| TCGA-A3-3317 | 0.3231 | 0.1061 | 0.9854 | 0.5476 | 0.9948 | 0.288 | 0.7804 | 1.149434 | high |
| TCGA-B8-4154 | 0.1275 | 0.0822 | 0.9899 | 0.1268 | 0.9852 | 0.16599 | 0.824 | 0.480943 | low |
| TCGA-B2-5636 | 0.5069 | 0.0691 | 1 | 0.1297 | 0.9835 | 0.1188 | 0.4576 | 1.33949 | high |
| TCGA-B0-4817 | 0.2321 | 0.056 | 0.9619 | 0.50647 | 0.9392 | 0.4263 | 0.926 | 1.218222 | high |
| TCGA-BP-5010 | 0.4353 | 0.2835 | 0.9641 | 0.7516 | 0.9721 | 0.2843 | 0.5438 | 3.930993 | high |
| TCGA-BP-4325 | 0.1067 | 0.2119 | 0.9955 | 0.3526 | 0.9943 | 0.3915 | 0.8843 | 0.746502 | low |
| TCGA-BP-5008 | 0.2477 | 0.09 | 0.9833 | 0.2007 | 0.9964 | 0.2415 | 0.7745 | 0.72217 | low |
| TCGA-B0-5707 | 0.2009 | 0.0429 | 1 | 0.28447 | 0.9902 | 0.235 | 0.3855 | 1.05338 | high |
| TCGA-CZ-5454 | 0.1634 | 0.0365 | 0.9962 | 0.1268 | 0.9868 | 0.1464 | 0.7262 | 0.506435 | low |
| TCGA-BP-4807 | 0.0635 | 0.0578 | 0.9894 | 0.0462 | 0.9858 | 0.0315 | 0.847 | 0.328917 | low |
| TCGA-A3-3352 | 0.2475 | 0.0747 | 0.9855 | 0.2604 | 0.9608 | 0.3484 | 0.8839 | 0.815518 | low |
| TCGA-B0-5712 | 0.1514 | 0.0849 | 0.9946 | 0.1167 | 0.984 | 0.2853 | 0.8794 | 0.507132 | low |
| TCGA-B0-4843 | 0.3511 | 0.3372 | 0.9827 | 0.4296 | 0.9856 | 0.3741 | 0.4652 | 2.9746 | high |
| TCGA-B8-A54H | 0.1099 | 0.0645 | 0.9962 | 0.256 | 0.941 | 0.3536 | 0.7888 | 0.749745 | low |
| TCGA-CZ-5456 | 0.2311 | 0.0288 | 0.9824 | 0.108 | 0.996 | 0.2369 | 0.8939 | 0.495504 | low |
| TCGA-B0-4828 | 0.2805 | 0.2061 | 0.9658 | 1 | 0.9117 | 0.8598 | 0.8457 | 4.890802 | high |
| TCGA-AK-3444 | 0.0844 | 0.1285 | 0.995 | 0.1891 | 0.9768 | 0.274 | 0.9115 | 0.506321 | low |
| TCGA-CJ-6031 | 0.2178 | 0.015 | 0.9924 | 0.139 | 0.9662 | 0.101 | 0.6867 | 0.612516 | low |
| TCGA-B8-A54K | 0.1675 | 0.0769 | 0.9906 | 0.2746 | 0.9936 | 0.3154 | 0.5965 | 0.879948 | high |
| TCGA-DV-5566 | 0.0821 | 0.0311 | 1 | 0.2251 | 0.9742 | 0.1188 | 0.8276 | 0.413981 | low |
| TCGA-B0-5113 | 0.3559 | 0.1097 | 0.9918 | 0.139 | 0.9782 | 0.3095 | 0.645 | 1.128427 | high |
| TCGA-BP-4169 | 0.5407 | 0.1582 | 0.9711 | 0.1518 | 0.975 | 0.2721 | 0.5091 | 2.26918 | high |
| TCGA-CZ-5986 | 0.1463 | 0.0087 | 1 | 0.0511 | 0.9901 | 0.1909 | 0.8473 | 0.373632 | low |
| TCGA-CJ-4640 | 0.1363 | 0.0448 | 0.9829 | 0.2531 | 0.9979 | 0.3206 | 0.6905 | 0.701905 | low |
| TCGA-B4-5836 | 0.1848 | 0.1205 | 0.9822 | 0.1427 | 0.9911 | 0.121 | 0.8244 | 0.558846 | low |
| TCGA-B0-5713 | 0.1463 | 0.0521 | 0.9526 | 0.2664 | 0.9969 | 0.155 | 0.9134 | 0.546676 | low |
| TCGA-BP-4790 | 0.1687 | 0.1749 | 0.9819 | 0.1949 | 0.974 | 0 | 0.7357 | 0.68052 | low |
| TCGA-BP-4174 | 0.2457 | 0.1512 | 0.9914 | 0.1949 | 0.999 | 0.3034 | 0.7546 | 0.821089 | low |
| TCGA-BP-4161 | 0.227 | 0.1589 | 0.9797 | 0.3009 | 0.9254 | 0.2296 | 0.7706 | 1.201593 | high |
| TCGA-BP-5183 | 0.3312 | 0.0462 | 0.9835 | 0.6772 | 0.9521 | 0.371 | 0.5137 | 2.267982 | high |
| TCGA-CW-5588 | 0.2869 | 0 | 0.9896 | 0.1268 | 0.9848 | 0.1487 | 0.533 | 0.806551 | low |
| TCGA-BP-4970 | 0.1505 | 0.0539 | 0.9941 | 0.1771 | 0.9824 | 0.1226 | 0.7139 | 0.546818 | low |
| TCGA-A3-3319 | 0.2139 | 0.0578 | 0.9398 | 0.7329 | 0.9829 | 0.4017 | 0.7561 | 1.656509 | high |
| TCGA-BP-4349 | 0.2357 | 0.28 | 0.9937 | 0.2211 | 0.9855 | 0.3791 | 0.754 | 1.172337 | high |
| TCGA-A3-3335 | 0.2368 | 0.0543 | 0.9858 | 0.3262 | 0.9909 | 0.2922 | 0.5806 | 1.029966 | high |
| TCGA-CZ-5464 | 0.317 | 0.0484 | 0.9625 | 0.1719 | 0.8843 | 0.1719 | 0.6242 | 1.611321 | high |
| TCGA-AK-3436 | 0.4851 | 0.1367 | 0.9918 | 0.0571 | 0.9457 | 0.2327 | 0.7274 | 1.312247 | high |
| TCGA-AK-3427 | 0.0618 | 0.03652 | 1 | 0.1771 | 0.9882 | 0.4302 | 0.8698 | 0.468363 | low |
| TCGA-BP-4163 | 0.3275 | 0.1544 | 0.9906 | 0.2792 | 0.996 | 0.2185 | 0.5551 | 1.256686 | high |
| TCGA-BP-4170 | 0.1993 | 0.1123 | 0.9724 | 0.0832 | 0.9804 | 0.1082 | 0.754 | 0.654342 | low |
| TCGA-B0-5088 | 0.1705 | 0.0782 | 0.9845 | 0.3456 | 0.971 | 0.3206 | 0.7865 | 0.829767 | low |
| TCGA-BP-4329 | 0.3653 | 0.2742 | 0.9912 | 0.2167 | 0.9639 | 0.0394 | 0.6337 | 1.358016 | high |
| TCGA-CW-5591 | 0.062 | 0.0246 | 0.9956 | 0.1569 | 0.9929 | 0.1202 | 0.7288 | 0.407074 | low |
| TCGA-CW-5580 | 0.3927 | 0.0691 | 0.9843 | 0.022 | 0.9818 | 0.1251 | 0.5304 | 1.024398 | high |
| TCGA-BP-4177 | 0.3563 | 0.0182 | 0.9882 | 0.139 | 0.9957 | 0.1055 | 0.4856 | 0.927345 | high |
| TCGA-B0-4837 | 0.054 | 0.1061 | 0.9529 | 0.2167 | 0.949 | 0.1817 | 0.5841 | 1.009754 | high |
| TCGA-CJ-4907 | 0.2325 | 0.0837 | 0.9963 | 0.1233 | 0.9553 | 0.1682 | 0.7387 | 0.713242 | low |
| TCGA-CJ-4891 | 0.5151 | 0.2835 | 0.9614 | 0.8058 | 0.9175 | 0.472 | 0.61543 | 6.661406 | high |
| TCGA-A3-3365 | 0.1139 | 0.1182 | 0.9774 | 0.1284 | 1 | 0.0557 | 0.7795 | 0.483217 | low |
| TCGA-BP-4982 | 0.1716 | 0.0928 | 0.9846 | 0.2541 | 0.9863 | 0.1711 | 0.7676 | 0.653538 | low |
| TCGA-B8-5549 | 0.1216 | 0.0488 | 0.9946 | 0.1023 | 0.9963 | 0.0394 | 0.8436 | 0.348546 | low |
| TCGA-MM-A563 | 0.3346 | 0.2256 | 0.9888 | 0.3212 | 0.9592 | 0.3154 | 0.7817 | 1.42584 | high |
| TCGA-B0-4688 | 0.7069 | 0.1842 | 0.9697 | 0.37237 | 0.8684 | 0.4937 | 0.1353 | 12.99657 | high |
| TCGA-BP-5194 | 0.0436 | 0.0633 | 0.9908 | 0.0448 | 0.9908 | 0.0702 | 0.8205 | 0.335658 | low |
| TCGA-CJ-4634 | 0.1356 | 0.1735 | 0.9866 | 0.4207 | 0.9795 | 0.2339 | 0.8561 | 0.777154 | low |
| TCGA-CZ-5462 | 0.2932 | 0.118 | 0.9916 | 0.244 | 0.9912 | 0.226 | 0.6357 | 0.988254 | high |
| TCGA-CJ-4902 | 0.2677 | 0.0759 | 0.9929 | 0.2174 | 0.9848 | 0.2725 | 0.6273 | 0.932127 | high |
| TCGA-BP-4981 | 0.3337 | 0.1565 | 0.9729 | 0.5476 | 0.9917 | 0.2521 | 0.4215 | 2.24725 | high |
| TCGA-A3-A6NI | 0.3233 | 0.0744 | 0.9829 | 0.3798 | 0.9873 | 0.2629 | 0.8318 | 0.905126 | high |
| TCGA-BP-5195 | 0.1889 | 0.0881 | 0.9913 | 0.1005 | 0.955 | 0.0473 | 0.7678 | 0.576903 | low |
| TCGA-BP-4782 | 0.1659 | 0.0702 | 0.9915 | 0.2251 | 0.992 | 0.1359 | 0.7329 | 0.575565 | low |
| TCGA-A3-3311 | 0.2044 | 0.0177 | 0.9796 | 0.3526 | 0.987 | 0.1332 | 0.8067 | 0.612497 | low |
| TCGA-CZ-4864 | 0.2123 | 0.1139 | 0.9926 | 0.3131 | 0.9934 | 0.2498 | 0.8302 | 0.693992 | low |
| TCGA-B2-4102 | 0.227 | 0.0878 | 0.9661 | 0.3577 | 0.997 | 0.1909 | 0.8013 | 0.800391 | low |
| TCGA-CJ-4878 | 0.2974 | 0.2503 | 0.9809 | 0.3262 | 0.9627 | 0.4471 | 0.92 | 1.361435 | high |
| TCGA-AK-3428 | 0.186 | 0.0619 | 0.9896 | 0.0883 | 0.9652 | 0.12169 | 0.8372 | 0.510901 | low |
| TCGA-BP-4971 | 0.3723 | 0.3333 | 0.9635 | 0.8667 | 0.9955 | 0.4105 | 0.471 | 4.810459 | high |
| TCGA-CJ-5676 | 0.445 | 0.1061 | 0.9846 | 0.0619 | 0.9781 | 0.4144 | 0.6094 | 1.490594 | high |
| TCGA-B8-A54J | 0.158 | 0.0949 | 0.9864 | 0.1828 | 0.9864 | 0.235 | 0.8184 | 0.593669 | low |
| TCGA-BP-4999 | 0.2139 | 0.1128 | 0.99 | 0.2197 | 0.9849 | 0.2316 | 0.7864 | 0.711087 | low |
| TCGA-BP-4332 | 0.1616 | 0.1652 | 1 | 0.3113 | 0.9932 | 0.3752 | 0.6897 | 0.922692 | high |
| TCGA-CZ-4863 | 0.1348 | 0.0523 | 0.9551 | 0.257 | 0.9843 | 0.3124 | 0.799 | 0.764152 | low |
| TCGA-A3-3374 | 0.0982 | 0 | 1 | 0.244 | 0.9884 | 0.3517 | 0.7157 | 0.565695 | low |
| TCGA-A3-3326 | 0.227 | 0.0543 | 0.9927 | 0.5923 | 0.993 | 0.2206 | 0.9129 | 0.693106 | low |
| TCGA-T7-A92I | 0.0556 | 0.13 | 0.9966 | 0.4862 | 0.9911 | 0.2568 | 0.7601 | 0.694776 | low |
| TCGA-AK-3425 | 0.2044 | 0.0182 | 0.9663 | 0.1335 | 0.9855 | 0.2902 | 0.6272 | 0.846564 | high |
| TCGA-CJ-4894 | 0.29 | 0.0691 | 0.9846 | 0.5476 | 0.987 | 0.2934 | 0.8364 | 0.985904 | high |
| TCGA-AK-3458 | 0.151 | 0.1393 | 1 | 0.2225 | 0.9676 | 0.524 | 0.6463 | 1.11803 | high |
| TCGA-B0-5106 | 0.3477 | 0.1247 | 0.9792 | 0.2289 | 0.9855 | 0.4206 | 0.6941 | 1.337923 | high |
| TCGA-A3-3382 | 0.4614 | 0.0412 | 0.993 | 0.1314 | 0.9888 | 0.2047 | 0.4996 | 1.238417 | high |
| TCGA-BP-4353 | 0.4781 | 0.1401 | 0.9922 | 0.176 | 0.9829 | 0.1855 | 0.4856 | 1.623881 | high |
| TCGA-B0-5698 | 0.2346 | 0.0381 | 0.9912 | 0.0539 | 0.972 | 0.1682 | 0.7408 | 0.59507 | low |
| TCGA-CZ-4866 | 0.1477 | 0.0271 | 0.9804 | 0.5137 | 0.9909 | 0.2041 | 0.719 | 0.766819 | low |
| TCGA-BP-4799 | 0.3912 | 0.2289 | 0.9282 | 0.3675 | 0.7345 | 0.4855 | 0.569 | 10.62423 | high |
| TCGA-CW-5583 | 0.1242 | 0.0675 | 0.9923 | 0.0971 | 0.9883 | 0.1248 | 0.8586 | 0.404008 | low |
| TCGA-B0-5085 | 0.128 | 0.5743 | 0.9584 | 0.9394 | 0.9752 | 0.5027 | 0.8552 | 3.755065 | high |
| TCGA-CJ-5683 | 0.1441 | 0.0842 | 0.9941 | 0.113 | 0.9909 | 0.2026 | 0.7674 | 0.523413 | low |
| TCGA-CW-5590 | 0.3123 | 0.108 | 0.9799 | 0.2149 | 0.9729 | 0.1766 | 0.6768 | 1.027229 | high |
| TCGA-CZ-5457 | 0.0754 | 0.0943 | 1 | 0.094 | 0.9884 | 0.1167 | 0.7845 | 0.40947 | low |
| TCGA-B8-A7U6 | 0.1563 | 0.1147 | 0.993 | 0.2265 | 0.9862 | 0.6663 | 0.835 | 0.915855 | high |
| TCGA-BP-5185 | 0.0646 | 0.2591 | 1 | 0.5867 | 0.9012 | 0.46234 | 0.9229 | 1.383288 | high |
| TCGA-BP-4763 | 0.4551 | 0.0133 | 0.97 | 0.4089 | 0.9608 | 0.1643 | 0.3962 | 2.124024 | high |
| TCGA-BP-5180 | 0.3677 | 0.0843 | 0.9833 | 0.2374 | 0.9824 | 0.2327 | 0.6815 | 1.0827 | high |
| TCGA-B2-5635 | 0.3612 | 0.0619 | 0.9855 | 0.3009 | 0.9792 | 0.4855 | 0.7565 | 1.254497 | high |
| TCGA-BP-4789 | 0.2006 | 0.0471 | 1 | 0.1949 | 0.9823 | 0.1297 | 0.7225 | 0.571546 | low |
| TCGA-A3-A8OV | 0.0705 | 0.1018 | 1 | 0.5867 | 0.9808 | 0.1331 | 0.9013 | 0.545855 | low |
| TCGA-CJ-4643 | 0.1189 | 0.1393 | 0.9842 | 0.3629 | 0.9861 | 0.1955 | 0.9023 | 0.602969 | low |
| TCGA-BP-4326 | 0.4416 | 0.0878 | 0.9682 | 0.212 | 0.9857 | 0.1635 | 0.6444 | 1.282281 | high |
| TCGA-BP-4960 | 0.1971 | 0.1695 | 0.9832 | 0.6289 | 0.9532 | 0.3816 | 0.5438 | 2.098031 | high |
| TCGA-B0-5121 | 0.2396 | 0.0704 | 1 | 0.1794 | 0.9856 | 0.2158 | 0.7122 | 0.683136 | low |
| TCGA-B2-4099 | 0.1889 | 0.1496 | 0.9725 | 0.2531 | 0.9915 | 0.2614 | 0.8385 | 0.773154 | low |
| TCGA-BP-5168 | 0.1868 | 0.0782 | 0.9931 | 0.0777 | 0.9661 | 0.1359 | 0.8625 | 0.497926 | low |
| TCGA-DV-A4W0 | 0.3068 | 0.0769 | 0.9765 | 0.2844 | 0.9955 | 0.3603 | 0.6885 | 1.107854 | high |
| TCGA-CJ-4905 | 0.0584 | 0.061 | 0.9862 | 0.0428 | 0.9979 | 0.0411 | 0.634 | 0.430931 | low |
| TCGA-CJ-5675 | 0.3206 | 0 | 1 | 0.1949 | 0.9925 | 0.0949 | 0.6731 | 0.634028 | low |
| TCGA-AK-3443 | 0.0867 | 0 | 0.9927 | 0.5636 | 0.9958 | 0.1874 | 0.9618 | 0.434867 | low |
| TCGA-MW-A4EC | 0.165 | 0.04 | 0.9865 | 0.4645 | 0.9781 | 0.1872 | 0.8715 | 0.627815 | low |
| TCGA-A3-3325 | 0.2357 | 0.0462 | 0.9793 | 0.3718 | 0.9851 | 0.3427 | 0.6676 | 1.044513 | high |
| TCGA-CZ-5469 | 0.5754 | 0.0665 | 0.9255 | 0.5158 | 0.9933 | 0.2981 | 0.0903 | 6.192712 | high |
| TCGA-AK-3453 | 0.0659 | 0.1572 | 0.99 | 0.6289 | 1 | 0.4932 | 0.8604 | 0.912812 | high |
| TCGA-CZ-5452 | 0.4628 | 0.0393 | 0.9959 | 0.3102 | 0.9844 | 0.2555 | 0.555 | 1.392579 | high |
| TCGA-CJ-4901 | 0.2693 | 0.0512 | 0.9837 | 0.4524 | 0.9751 | 0.3062 | 0.1588 | 2.402027 | high |
| TCGA-B0-4700 | 0.2827 | 0.09 | 0.9834 | 0.4515 | 0.9689 | 0.449 | 0.441 | 2.093346 | high |
| TCGA-B2-3923 | 0.1587 | 0.0471 | 0.9929 | 0.7722 | 1 | 0.3299 | 0.7757 | 0.925537 | high |
| TCGA-BP-5184 | 0.0914 | 0.0699 | 0.9937 | 0.3212 | 0.9929 | 0.1259 | 0.9308 | 0.403845 | low |
| TCGA-B0-4810 | 0.3128 | 0.2289 | 0.9851 | 0.4657 | 0.984 | 0.5466 | 0.6151 | 2.262122 | high |
| TCGA-BP-5176 | 0.0803 | 0.0703 | 0.995 | 0.1111 | 0.9777 | 0.114 | 0.9095 | 0.362916 | low |
| TCGA-CZ-5463 | 0.1247 | 0.0241 | 0.9941 | 0.1537 | 0.9961 | 0.1946 | 0.8943 | 0.381167 | low |
| TCGA-B2-5633 | 0.3979 | 0.0437 | 0.9838 | 0.071 | 0.9839 | 0.0465 | 0.5778 | 0.882767 | high |
| TCGA-B8-A54E | 0.0642 | 0.2498 | 0.9809 | 0.2668 | 1 | 0.25344 | 0.9239 | 0.609762 | low |
| TCGA-B0-4844 | 0.3464 | 0.1578 | 0.9784 | 0.8288 | 0.9418 | 0.4522 | 0.5891 | 3.372968 | high |
| TCGA-BP-5169 | 0.4244 | 0.2289 | 0.9443 | 0.4466 | 0.8802 | 0.4081 | 0.5042 | 5.643441 | high |
| TCGA-B0-4714 | 0.228 | 0.4661 | 1 | 0.5691 | 0.9843 | 0.3002 | 0.9179 | 1.508992 | high |
| TCGA-CZ-4853 | 0.148 | 0.0894 | 0.9884 | 0.1557 | 0.965 | 0.2853 | 0.8977 | 0.579198 | low |

Table S9

| ID | SERP2\|25779\|ME | ANKRD13A\|250011\|ME | TPM2\|86278\|ME | P4HA1\|12122\|ME | STEAP3\|95656\|ME | ZNF611\|101327\|ME | ERBB2IP\|102012\|ME | SLC39A14\|140283\|ME | ADAM28\|83091\|ME | Risk score | risk |
| --- | --- | --- | --- | --- | --- | --- | --- | --- | --- | --- | --- |
| TCGA-CJ-4637 | 0.8463 | 0.0331 | 0.9762 | 0.3901 | 0.6624 | 0.2878 | 0.3733 | 0.1333 | 0.9919 | 2.669883 | high |
| TCGA-CZ-4861 | 0.9643 | 0.0509 | 0.9825 | 0.4314 | 0.6094 | 0.4239 | 0.484 | 0.1443 | 0.9732 | 1.009064 | high |
| TCGA-BP-4964 | 0.9587 | 0 | 0.9826 | 0.4485 | 0.2224 | 0.2902 | 0.5266 | 0.3041 | 0.9283 | 0.651859 | low |
| TCGA-CJ-4887 | 0.8118 | 0.1622 | 0.9866 | 0.584 | 0.0444 | 0.1985 | 0.7863 | 0.3474 | 0.9612 | 0.885093 | low |
| TCGA-BP-5198 | 0.9753 | 0.0072 | 0.9803 | 0.3966 | 0.4387 | 0.3686 | 0.435 | 0.1736 | 1 | 0.86022 | low |
| TCGA-BP-4967 | 0.9703 | 0.0217 | 0.9777 | 0.5793 | 0.6141 | 0.2504 | 0.8802 | 0.4443 | 0.9061 | 0.396641 | low |
| TCGA-A3-3351 | 0.9224 | 0.0747 | 0.993 | 0.4417 | 0.1542 | 0.0783 | 0.5141 | 0.2545 | 0.9915 | 1.127935 | high |
| TCGA-B0-4710 | 0.9208 | 0.0116 | 0.9679 | 0.545 | 0.4287 | 0.0805 | 0.4431 | 0.2218 | 0.9879 | 1.131663 | high |
| TCGA-B0-5083 | 1 | 0.0235 | 0.9865 | 0.5586 | 0.9045 | 0.2015 | 0.7504 | 0.1856 | 1 | 0.632355 | low |
| TCGA-BP-4803 | 1 | 0.0094 | 0.9886 | 0.5072 | 0.6473 | 0.101 | 0.469 | 0.2501 | 1 | 0.808452 | low |
| TCGA-CJ-4889 | 0.9499 | 0.0211 | 0.9898 | 0.4802 | 0.3033 | 0.1813 | 0.5138 | 0.3828 | 0.9685 | 0.649075 | low |
| TCGA-B0-5080 | 0.9929 | 0.0132 | 0.9857 | 0.4684 | 1 | 0.20076 | 0.4271 | 0.0405 | 1 | 1.533925 | high |
| TCGA-CW-5587 | 0.9772 | 0.0098 | 0.9897 | 0.4394 | 0.1813 | 0.6142 | 0.8429 | 0.268 | 0.9879 | 0.239275 | low |
| TCGA-A3-3376 | 0.9135 | 0.0379 | 0.9914 | 0.4591 | 0.1615 | 0.237 | 0.1777 | 0.1626 | 0.9941 | 1.267988 | high |
| TCGA-CJ-4897 | 0.9781 | 0.0204 | 0.9946 | 0.5194 | 0.1406 | 0.4809 | 0.815 | 0.2686 | 1 | 0.223023 | low |
| TCGA-CW-6088 | 0.9623 | 0.0283 | 0.9949 | 0.6409 | 0.0905 | 0.4538 | 0.7648 | 0.5152 | 0.9723 | 0.13346 | low |
| TCGA-B8-5552 | 0.9749 | 0.0151 | 0.9919 | 0.4661 | 0.2678 | 0.4181 | 0.483 | 0.2162 | 0.9916 | 0.494951 | low |
| TCGA-CW-5589 | 0.9908 | 0.0123 | 0.9881 | 0.5338 | 0.1626 | 0.4592 | 0.2622 | 0.393 | 1 | 0.313154 | low |
| TCGA-B0-4833 | 0.9013 | 0.2801 | 0.9797 | 0.4458 | 0.0116 | 0.0333 | 0.50333 | 0.4454 | 1 | 1.745442 | high |
| TCGA-CZ-4859 | 0.9962 | 0.008 | 0.9874 | 0.3938 | 0 | 0.3058 | 0.6063 | 0.5989 | 0.9213 | 0.324484 | low |
| TCGA-A3-3320 | 0.9019 | 0.0968 | 0.9893 | 0.3551 | 0.1029 | 0.0974 | 0.6958 | 0.3975 | 0.9219 | 1.298043 | high |
| TCGA-MM-A564 | 0.9305 | 0.1313 | 0.9845 | 0.4802 | 0.3175 | 0.04314 | 0.35473 | 0.0776 | 0.560723 | 5.912183 | high |
| TCGA-AS-3777 | 1 | 0.0287 | 0.9795 | 0.552 | 0.7159 | 0.18059 | 0.7851 | 0.5746 | 1 | 0.335169 | low |
| TCGA-B0-5095 | 0.8752 | 0.0423 | 0.9817 | 0.5036 | 0.9411 | 0.203 | 0.518 | 0.0842 | 0.9018 | 2.672473 | high |
| TCGA-BP-4795 | 0.9856 | 0.0098 | 0.9926 | 0.4139 | 0.4424 | 0.2359 | 0.2105 | 0.0569 | 1 | 1.328618 | high |
| TCGA-BP-4331 | 0.9522 | 0.0365 | 0.9866 | 0.4991 | 0.4367 | 0 | 0.4055 | 0.1988 | 1 | 1.283772 | high |
| TCGA-A3-A6NJ | 0.9421 | 0.0281 | 0.9881 | 0.5882 | 0.2029 | 0.2587 | 0.3232 | 0.3995 | 0.9735 | 0.481764 | low |
| TCGA-B0-4701 | 0.9507 | 0.1061 | 0.9782 | 0.4648 | 0.182 | 0.0737 | 0.6307 | 0.3162 | 0.9718 | 0.97529 | high |
| TCGA-A3-3367 | 0.9532 | 0.0388 | 0.9919 | 0.4953 | 0.2361 | 0.3494 | 0.4573 | 0.4501 | 0.9894 | 0.426147 | low |
| TCGA-B0-5402 | 0.9693 | 0.0202 | 0.9784 | 0.4696 | 0.8018 | 0.2097 | 0.7104 | 0.0652 | 0.9838 | 1.159838 | high |
| TCGA-B0-5691 | 1 | 0.022 | 0.9861 | 0.4757 | 0.1085 | 0.2765 | 0.7125 | 0.2788 | 1 | 0.355963 | low |
| TCGA-BP-4342 | 0.8782 | 0.0256 | 0.9755 | 0.3801 | 0.6116 | 0.1266 | 0.1318 | 0.3891 | 0.9822 | 2.59126 | high |
| TCGA-A3-3373 | 0.9661 | 0.0117 | 0.9908 | 0.7174 | 0.0966 | 0.496 | 0.61 | 0.6572 | 0.992 | 0.087128 | low |
| TCGA-BP-4341 | 0.9274 | 0.1051 | 0.9936 | 0.6217 | 0.1264 | 0.0305 | 0.5515 | 0.4658 | 1 | 0.512291 | low |
| TCGA-CW-6090 | 0.9552 | 0.0389 | 0.9908 | 0.4345 | 0.5529 | 0 | 0.4043 | 0.155 | 1 | 1.758508 | high |
| TCGA-A3-3307 | 0.9307 | 0.0567 | 0.9968 | 0.4959 | 0.097 | 0.4855 | 0.6123 | 0.3036 | 1 | 0.362779 | low |
| TCGA-B2-3924 | 0.6468 | 0.0813 | 0.9738 | 0.5433 | 0 | 0 | 0.3447 | 0.3987 | 0.8241 | 4.65607 | high |
| TCGA-B0-5706 | 1 | 0.0136 | 0.9717 | 0.4455 | 0.4842 | 0.238422 | 0.52285 | 0.3943 | 0.9756 | 0.635124 | low |
| TCGA-BP-4963 | 0.8578 | 0.059 | 0.9866 | 0.4107 | 0.7121 | 0.288 | 0.7798 | 0.2792 | 0.975 | 1.400273 | high |
| TCGA-BP-4343 | 1 | 0.0182 | 0.9643 | 0.3661 | 0.727 | 0.26179 | 0.3399 | 0.3161 | 1 | 1.263133 | high |
| TCGA-B0-5703 | 1 | 0 | 0.9946 | 0.6054 | 0.3447 | 0.16904 | 0.8047 | 0.3281 | 1 | 0.257459 | low |
| TCGA-CJ-6027 | 0.9763 | 0.0317 | 0.9321 | 0.4338 | 0.9541 | 0.2847 | 0.5496 | 0.1205 | 0.9507 | 2.020563 | high |
| TCGA-B0-5108 | 0.9888 | 0.0281 | 0.9767 | 0.389 | 0.6417 | 0.3127 | 0.1768 | 0.062 | 0.975 | 1.849219 | high |
| TCGA-BP-4965 | 0.9604 | 0.0223 | 0.9852 | 0.4813 | 0.1624 | 0.3628 | 0.8375 | 0.3127 | 1 | 0.322455 | low |
| TCGA-A3-A6NL | 0.9542 | 0.0341 | 0.9906 | 0.5195 | 0.1648 | 0.4056 | 0.5366 | 0.256 | 0.974 | 0.432778 | low |
| TCGA-CJ-5672 | 0.9884 | 0.0248 | 0.9926 | 0.4721 | 1 | 0.14705 | 0.6456 | 0.137 | 1 | 1.162942 | high |
| TCGA-B8-5158 | 1 | 0 | 0.9588 | 0.4548 | 0.9023 | 0.386 | 0.6177 | 0.1189 | 0.9928 | 0.921051 | high |
| TCGA-CJ-4876 | 0.9148 | 0.0674 | 0.9918 | 0.5499 | 0.1109 | 0 | 1 | 0.3797 | 1 | 0.4562 | low |
| TCGA-B0-5115 | 0.9234 | 0.1279 | 0.9722 | 0.4846 | 0.0518 | 0 | 0.432 | 0.2139 | 0.9612 | 1.690555 | high |
| TCGA-CJ-4916 | 0.9023 | 0 | 0.9905 | 0.486 | 0.1803 | 0 | 0.3448 | 0.3996 | 0.9845 | 0.990394 | high |
| TCGA-B0-4848 | 0.993 | 0.0133 | 0.9948 | 0.4348 | 0.8024 | 0.20047 | 0.7277 | 0.0605 | 1 | 0.991093 | high |
| TCGA-B4-5377 | 0.982 | 0.0095 | 0.9938 | 0.5631 | 0.2002 | 0.4332 | 0.8124 | 0.4111 | 1 | 0.171747 | low |
| TCGA-B0-4712 | 1 | 0.0714 | 0.92 | 0.4082 | 0.7385 | 0.14264 | 0.6456 | 0.0446 | 0.9471 | 2.562454 | high |
| TCGA-A3-3324 | 0.8918 | 0 | 0.9953 | 0.4187 | 0.0898 | 0.2884 | 0.2074 | 0.3756 | 1 | 0.846785 | low |
| TCGA-CZ-5465 | 0.9759 | 0.0245 | 0.9833 | 0.4572 | 0.6152 | 0.3375 | 0.5522 | 0.1939 | 1 | 0.765412 | low |
| TCGA-CZ-4860 | 1 | 0.0527 | 0.9817 | 0.3075 | 0.7591 | 0.28398 | 0.2808 | 0.0164 | 1 | 2.429232 | high |
| TCGA-B0-5102 | 0.9639 | 0.0538 | 0.9843 | 0.5235 | 0.1099 | 0.0617 | 0.5701 | 0.0795 | 1 | 0.863697 | low |
| TCGA-B0-5690 | 0.981 | 0 | 0.9884 | 0.4828 | 0.4034 | 0.21292 | 0.1684 | 0.2183 | 1 | 0.912635 | high |
| TCGA-BP-4160 | 0.9758 | 0 | 0.9896 | 0.5235 | 0 | 0.23657 | 0.3197 | 0.3079 | 0.9688 | 0.471453 | low |
| TCGA-B0-4697 | 0.7893 | 0.1657 | 0.9938 | 0.3125 | 0.431 | 0.0918 | 0.40823 | 0.0933 | 0.6967 | 13.14314 | high |
| TCGA-AK-3454 | 0.9553 | 0.0685 | 0.9815 | 0.4351 | 0.0845 | 0.188 | 0.6551 | 0.2975 | 0.9747 | 0.711268 | low |
| TCGA-B0-5693 | 0.9707 | 0 | 0.9857 | 0.6021 | 0.141 | 0.2163 | 0.4551 | 0.317 | 1 | 0.359767 | low |
| TCGA-BP-4985 | 0.9772 | 0.0208 | 0.9793 | 0.5268 | 0.5809 | 0.0244 | 0.2329 | 0.0435 | 1 | 1.692435 | high |
| TCGA-B0-4718 | 0.7489 | 0.2214 | 0.9956 | 0.4628 | 0.1738 | 0 | 0.43281 | 0.1634 | 1 | 4.833369 | high |
| TCGA-B0-5109 | 0.8749 | 0 | 0.9797 | 0.3329 | 0.5025 | 0 | 0.2731 | 0.04 | 0.8972 | 5.375855 | high |
| TCGA-A3-3359 | 0.9161 | 0.1097 | 0.9846 | 0.547 | 0.0215 | 0.1784 | 0.6098 | 0.3935 | 0.9813 | 0.581309 | low |
| TCGA-DV-5569 | 0.9699 | 0.0715 | 0.9894 | 0.4511 | 0.1498 | 0.113 | 0.387 | 0.4015 | 1 | 0.762898 | low |
| TCGA-B2-A4SR | 0.9002 | 0.0733 | 0.9937 | 0.4894 | 0.3648 | 0.4343 | 0.6424 | 0.3622 | 0.9433 | 0.611652 | low |
| TCGA-B0-4815 | 0.8233 | 0.0899 | 0.9721 | 0.3945 | 1 | 0.26 | 0.6075 | 0.1089 | 0.9712 | 4.104912 | high |
| TCGA-AK-3461 | 0.8982 | 0.0421 | 0.9853 | 0.5045 | 0.1222 | 0 | 0.7156 | 0.2231 | 0.9213 | 1.100161 | high |
| TCGA-CJ-4892 | 0.8509 | 0 | 0.9929 | 0.5306 | 0.0155 | 0 | 0.4767 | 0.4054 | 1 | 0.794496 | low |
| TCGA-B0-5695 | 0.9948 | 0.0628 | 0.9862 | 0.4945 | 0.102 | 0 | 0 | 0.28 | 0.9539 | 1.337054 | high |
| TCGA-BP-4975 | 0.9663 | 0.0571 | 0.9876 | 0.5182 | 0.2933 | 0.4019 | 0.4461 | 0.2017 | 0.9912 | 0.566458 | low |
| TCGA-AK-3456 | 1 | 0.0259 | 0.989 | 0.5657 | 0 | 0.5928 | 0.7723 | 0.0594 | 0.949 | 0.223003 | low |
| TCGA-BP-4159 | 0.9836 | 0.1283 | 0.9907 | 0.4661 | 0.1058 | 0.09038 | 0.42586 | 0.1623 | 1 | 1.095317 | high |
| TCGA-CZ-5458 | 0.9724 | 0 | 0.9874 | 0.5677 | 0.0559 | 0.5664 | 0.6193 | 0.2862 | 1 | 0.190149 | low |
| TCGA-AK-3431 | 0.9694 | 0.0297 | 0.9866 | 0.4991 | 0.6701 | 0.18555 | 0.4767 | 0.1063 | 0.965 | 1.209652 | high |
| TCGA-B8-A8YJ | 0.9524 | 0.0238 | 0.9841 | 0.517 | 0.674 | 0.2622 | 0.5218 | 0.105 | 1 | 0.972759 | high |
| TCGA-BP-5182 | 0.9834 | 0 | 0.9915 | 0.5534 | 0.2288 | 0.5219 | 0.4514 | 0.3048 | 0.9853 | 0.268089 | low |
| TCGA-B2-4101 | 0.9349 | 0.0918 | 0.9692 | 0.4415 | 0.1933 | 0.2765 | 0.5324 | 0.2884 | 0.975 | 0.959286 | high |
| TCGA-CJ-5681 | 0.9291 | 0.0106 | 0.9656 | 0.568 | 0.6944 | 0.2765 | 1 | 0.018 | 1 | 0.699006 | low |
| TCGA-BP-4994 | 0.9577 | 0.0232 | 0.9869 | 0.4487 | 0.3149 | 0.4529 | 0.88 | 0.2821 | 0.9813 | 0.365394 | low |
| TCGA-CJ-4903 | 0.9729 | 0 | 0.9871 | 0.5005 | 0.3239 | 0.259 | 0.3921 | 0.2923 | 0.9834 | 0.591505 | low |
| TCGA-BP-4760 | 0.9857 | 0.0133 | 0.987 | 0.5115 | 0.1914 | 0.4045 | 0.4062 | 0.0748 | 1 | 0.529172 | low |
| TCGA-BP-5190 | 0.9712 | 0.0577 | 0.9823 | 0.502 | 0.0209 | 0.344 | 0.4364 | 0.4108 | 1 | 0.390099 | low |
| TCGA-B0-5710 | 0.9866 | 0 | 0.9878 | 0.408 | 0.0688 | 0.3891 | 0.4059 | 0.0717 | 1 | 0.644423 | low |
| TCGA-CJ-4875 | 0.958 | 0.1981 | 0.9794 | 0.4724 | 0.1363 | 0.0525 | 0.0277 | 0.1681 | 1 | 2.540304 | high |
| TCGA-CJ-4874 | 0.9962 | 0.0177 | 0.9744 | 0.543 | 0.0399 | 0.24483 | 0.6873 | 0.4085 | 1 | 0.26589 | low |
| TCGA-B2-5641 | 0.9297 | 0.0167 | 0.9831 | 0.4011 | 0.7483 | 0.3117 | 0.6709 | 0.4547 | 0.9776 | 0.802913 | low |
| TCGA-CW-5584 | 0.9942 | 0.0085 | 0.988 | 0.4833 | 0.3911 | 0.6407 | 0.5654 | 0.2326 | 1 | 0.298226 | low |
| TCGA-B8-4143 | 0.8332 | 0 | 0.9669 | 0.2684 | 0.9713 | 0 | 0.6995 | 0.0678 | 0.9323 | 7.035311 | high |
| TCGA-B0-5120 | 0.9497 | 0 | 0.9899 | 0.4181 | 0.3984 | 0.3013 | 0.1706 | 0.2642 | 0.9867 | 1.056875 | high |
| TCGA-EU-5905 | 1 | 0.0141 | 0.9862 | 0.4898 | 0.8309 | 0.4795 | 0.5581 | 0.073 | 0.9652 | 0.723126 | low |
| TCGA-BP-4338 | 1 | 0.011 | 0.9696 | 0.5514 | 0.9466 | 0.1038 | 0.4461 | 0.1229 | 1 | 1.233957 | high |
| TCGA-A3-3358 | 0.9353 | 0.0822 | 0.9822 | 0.443 | 0.3503 | 0.1516 | 0.5594 | 0.4691 | 0.9856 | 0.852594 | low |
| TCGA-B0-4818 | 0.9281 | 0.0859 | 0.9941 | 0.551 | 0.0606 | 0.1281 | 0.4462 | 0.496 | 1 | 0.51787 | low |
| TCGA-BP-4976 | 0.9766 | 0.0098 | 0.9867 | 0.4884 | 0.1198 | 0.586 | 0.7797 | 0.3928 | 1 | 0.181664 | low |
| TCGA-BP-4345 | 0.9349 | 0.034 | 0.9783 | 0.3877 | 0.3455 | 0 | 0.1479 | 0.1832 | 0.9507 | 2.801401 | high |
| TCGA-CW-6093 | 0.9945 | 0 | 0.9914 | 0.5075 | 1 | 0.1452 | 0.3571 | 0.2959 | 1 | 1.013287 | high |
| TCGA-BP-4330 | 0.8843 | 0.0281 | 0.9942 | 0.4763 | 0.074 | 0 | 0.4468 | 0.4685 | 0.9768 | 0.898515 | high |
| TCGA-EU-5906 | 0.9919 | 0.0133 | 0.9743 | 0.5359 | 0.3009 | 0.6305 | 0.7027 | 0.4143 | 1 | 0.180255 | low |
| TCGA-B0-4846 | 0.9533 | 0.0448 | 0.9875 | 0.5571 | 0 | 0.113 | 0.6801 | 0.4372 | 1 | 0.353576 | low |
| TCGA-CJ-6030 | 0.9828 | 0 | 0.971 | 0.4965 | 0.4958 | 0.203 | 0.7439 | 0.132 | 0.9857 | 0.694686 | low |
| TCGA-BP-4983 | 0.8839 | 0.018 | 0.889 | 0.2938 | 0.7582 | 0.1823 | 0.9216 | 0.0116 | 0.9931 | 4.307628 | high |
| TCGA-B0-5697 | 0.9605 | 0.0125 | 0.9839 | 0.5183 | 0.1975 | 0 | 0.6573 | 0.1903 | 0.9523 | 0.810232 | low |
| TCGA-BP-4346 | 0.7741 | 0.0864 | 0.9786 | 0.513 | 0.2086 | 0.0839 | 0.5324 | 0.3358 | 0.987 | 1.824881 | high |
| TCGA-B0-4691 | 0.8992 | 0.0892 | 0.9728 | 0.4615 | 0.3511 | 0 | 0.49895 | 0.1219 | 0.8348 | 3.233475 | high |
| TCGA-A3-3328 | 0.9871 | 0.0303 | 0.9148 | 0.5625 | 0.1966 | 0.188 | 0.9161 | 0.037 | 1 | 0.659452 | low |
| TCGA-BP-4765 | 0.9459 | 0.0843 | 0.9891 | 0.489 | 0 | 0.2928 | 0.2677 | 0.4106 | 1 | 0.593412 | low |
| TCGA-CZ-4858 | 0.9882 | 0.0701 | 0.9864 | 0.3219 | 0.9589 | 0.3531 | 0.4856 | 0.0194 | 1 | 2.129155 | high |
| TCGA-B0-5117 | 0.9291 | 0.0424 | 0.9726 | 0.5527 | 0.4131 | 0 | 0.7123 | 0.12 | 1 | 1.090259 | high |
| TCGA-CZ-4865 | 0.9752 | 0 | 0.9832 | 0.5648 | 0.2301 | 0.2144 | 0.7651 | 0.2095 | 1 | 0.36855 | low |
| TCGA-CJ-6028 | 0.9736 | 0.0201 | 0.975 | 0.4029 | 0.2158 | 0.3891 | 0.4436 | 0.0523 | 1 | 0.88249 | low |
| TCGA-6D-AA2E | 0.9486 | 0.0178 | 0.9903 | 0.6017 | 0.3957 | 0 | 0.4904 | 0.1677 | 1 | 0.827076 | low |
| TCGA-CJ-4884 | 0.9581 | 0.0887 | 0.986 | 0.6052 | 0.5239 | 0.1618 | 0.4501 | 0.3094 | 0.943 | 0.823061 | low |
| TCGA-DV-5568 | 0.9074 | 0.1776 | 0.9764 | 0.4481 | 0.905 | 0 | 0.4216 | 0.1787 | 0.752 | 7.546498 | high |
| TCGA-BP-4961 | 0.9704 | 0.0198 | 0.9865 | 0.4718 | 0.3258 | 0.4174 | 0.6966 | 0.2769 | 1 | 0.394822 | low |
| TCGA-BP-4761 | 0.909 | 0.2995 | 0.9732 | 0.5635 | 0.6085 | 0.023 | 0.53842 | 0.0318 | 1 | 3.691008 | high |
| TCGA-CJ-5677 | 1 | 0.0106 | 0.9569 | 0.4454 | 0.5127 | 0.10868 | 0.3942 | 0.0665 | 1 | 1.492764 | high |
| TCGA-CZ-5460 | 0.9482 | 0 | 0.9947 | 0.4732 | 0.5042 | 0.4115 | 0.313 | 0.2069 | 0.9029 | 0.916666 | high |
| TCGA-CJ-4900 | 0.9654 | 0 | 0.9825 | 0.3657 | 0.4732 | 0 | 0.7147 | 0.0192 | 0.9738 | 1.732473 | high |
| TCGA-CZ-5451 | 0.9813 | 0.0095 | 0.9895 | 0.4908 | 0.1252 | 0.6153 | 0.575 | 0.2935 | 1 | 0.236133 | low |
| TCGA-B8-A54F | 0.9363 | 0.142 | 0.9707 | 0.4666 | 0.8724 | 0.5443 | 0.49152 | 0.3647 | 0.9866 | 1.024521 | high |
| TCGA-B0-4838 | 0.9512 | 0 | 0.9937 | 0.52 | 0.0608 | 0.1942 | 0.3892 | 0.3892 | 0.99 | 0.457431 | low |
| TCGA-B0-4699 | 0.8425 | 0.1284 | 0.8056 | 0.3779 | 1 | 0.1159 | 0.9051 | 0.1136 | 0.876 | 14.28065 | high |
| TCGA-BP-4781 | 0.9919 | 0.0495 | 0.9742 | 0.5027 | 0.824 | 0.4421 | 0.6033 | 0.1987 | 0.99 | 0.682342 | low |
| TCGA-CJ-4644 | 0.9823 | 0.0079 | 0.9878 | 0.2511 | 0.015 | 0.18877 | 0.302 | 0.1843 | 1 | 1.322504 | high |
| TCGA-B0-5701 | 0.9816 | 0.0187 | 0.9733 | 0.4736 | 0.7856 | 0.1566 | 0.8198 | 0.4296 | 1 | 0.606549 | low |
| TCGA-B0-5694 | 1 | 0.0314 | 0.9758 | 0.4665 | 0.4576 | 0.16134 | 0.1022 | 0.0851 | 1 | 1.526497 | high |
| TCGA-B8-4621 | 0.9897 | 0.0209 | 0.9289 | 0.5262 | 0.3328 | 0.2415 | 0.5867 | 0.1722 | 0.9907 | 0.77263 | low |
| TCGA-BP-5006 | 0.9578 | 0 | 0.969 | 0.5918 | 0.0702 | 0 | 0.5295 | 0.3325 | 1 | 0.522708 | low |
| TCGA-BP-5181 | 0.9713 | 0 | 0.9912 | 0.4235 | 0.0928 | 0 | 0.6949 | 0.38 | 1 | 0.570792 | low |
| TCGA-B0-5709 | 0.9578 | 0.0046 | 0.9777 | 0.5658 | 0.8612 | 0.5866 | 0.5638 | 0.2091 | 1 | 0.466166 | low |
| TCGA-BP-4167 | 1 | 0.1536 | 0.9936 | 0.4364 | 0.2798 | 0 | 0.37643 | 0.124 | 1 | 1.714703 | high |
| TCGA-CJ-4638 | 0.9756 | 0.1847 | 0.9902 | 0.5472 | 1 | 0 | 0 | 0.2 | 1 | 3.472681 | high |
| TCGA-EU-5907 | 0.9759 | 0 | 0.9799 | 0.507 | 0.8383 | 0.2424 | 0.5622 | 0.0915 | 1 | 0.988973 | high |
| TCGA-CJ-4869 | 0.9677 | 0.045 | 0.9924 | 0.5689 | 1 | 0.11714 | 0.2983 | 0.1201 | 1 | 1.540827 | high |
| TCGA-3Z-A93Z | 0.9642 | 0.0767 | 0.9894 | 0.5094 | 0.2214 | 0.10297 | 0.2543 | 0.2132 | 1 | 1.075424 | high |
| TCGA-BP-5196 | 0.9564 | 0 | 0.9757 | 0.4485 | 0.3293 | 0 | 0.6456 | 0.072 | 1 | 1.228845 | high |
| TCGA-AK-3433 | 0.95914 | 0.0281 | 0.959 | 0.4131 | 0.882 | 0.1638 | 0.8027 | 0.1905 | 1 | 1.385441 | high |
| TCGA-A3-3346 | 0.977 | 0.036 | 0.9463 | 0.4315 | 0.8291 | 0.1935 | 0.48875 | 0.0456 | 0.9708 | 2.224836 | high |
| TCGA-AK-3440 | 0.9183 | 0.0263 | 0.9879 | 0.5714 | 0.2331 | 0.1182 | 0.8323 | 0.0671 | 1 | 0.653503 | low |
| TCGA-B8-A54D | 0.7766 | 0.1802 | 0.9887 | 0.5364 | 0.5235 | 0.0343 | 0.7443 | 0.3929 | 0.9749 | 2.181454 | high |
| TCGA-BP-5200 | 0.9851 | 0.0095 | 0.9717 | 0.5275 | 0.5527 | 0.1337 | 0.4893 | 0.1268 | 1 | 0.93743 | high |
| TCGA-B0-5400 | 0.9727 | 0.1411 | 0.9918 | 0.4368 | 0.8396 | 0.06493 | 0.47946 | 0.053 | 1 | 2.620009 | high |
| TCGA-BP-4969 | 0.8986 | 0.0809 | 0.9832 | 0.5788 | 0.05 | 0.0661 | 0.3905 | 0.5933 | 1 | 0.574245 | low |
| TCGA-BP-4756 | 0.8839 | 0.0322 | 0.9657 | 0.6016 | 0.4478 | 0.2494 | 0.8933 | 0.1083 | 0.9354 | 0.805638 | low |
| TCGA-BP-4776 | 0.9056 | 0.2726 | 0.9943 | 0.4834 | 0.1173 | 0.0296 | 0.43867 | 0.2297 | 1 | 2.176885 | high |
| TCGA-CJ-4912 | 0.9589 | 0.0941 | 0.9888 | 0.3701 | 0.8728 | 0.1452 | 0.1318 | 0 | 1 | 4.195158 | high |
| TCGA-CZ-5985 | 0.9054 | 0.048 | 0.9728 | 0.4308 | 0.8054 | 0.3435 | 0.7579 | 0.2175 | 0.9908 | 1.195082 | high |
| TCGA-B0-5098 | 0.9515 | 0.0803 | 0.9954 | 0.2625 | 0.4328 | 0.071 | 0.2152 | 0.0669 | 0.99648 | 3.71801 | high |
| TCGA-B8-5545 | 0.9813 | 0.0082 | 0.9838 | 0.5477 | 0.445 | 0.3531 | 0.4141 | 0.3492 | 1 | 0.42529 | low |
| TCGA-BP-4758 | 0.9147 | 0.027 | 0.9798 | 0.4964 | 0.0395 | 0.0675 | 0.7641 | 0.204 | 1 | 0.716519 | low |
| TCGA-B0-4696 | 0.8973 | 0.0656 | 0.5975 | 0.5561 | 0.3769 | 0.0834 | 0.432433 | 0.0231 | 0.991444 | 18.22122 | high |
| TCGA-A3-3322 | 0.9267 | 0.0481 | 0.9949 | 0.4867 | 0.1222 | 0.1666 | 0.5931 | 0.3996 | 0.9707 | 0.591274 | low |
| TCGA-B0-5107 | 0.9335 | 0.2017 | 1 | 0.3851 | 0.6097 | 0.101 | 0.4216 | 0.0789 | 1 | 3.348271 | high |
| TCGA-DV-5565 | 0.9652 | 0.0255 | 0.989 | 0.4929 | 0.2837 | 0 | 0.2992 | 0.1481 | 1 | 1.254338 | high |
| TCGA-CZ-5989 | 0.927 | 0.0277 | 0.9583 | 0.5119 | 0 | 0.1695 | 0.2174 | 0.2534 | 1 | 0.989563 | high |
| TCGA-BP-4173 | 0.9211 | 0.0632 | 0.9876 | 0.4555 | 0.1091 | 0.19 | 0.3779 | 0.0696 | 0.9802 | 1.364344 | high |
| TCGA-CJ-4870 | 0.9765 | 0.0189 | 0.9889 | 0.4982 | 0.2367 | 0.23936 | 0.5732 | 0.4238 | 1 | 0.391364 | low |
| TCGA-BP-4974 | 0.8615 | 0.0798 | 0.9901 | 0.4694 | 0.6197 | 0.1425 | 0.2257 | 0.3183 | 0.9808 | 2.272221 | high |
| TCGA-B0-5075 | 0.9019 | 0 | 0.9722 | 0.5092 | 0.5341 | 0.0892 | 0.6876 | 0.2757 | 0.9562 | 1.074662 | high |
| TCGA-BP-4162 | 0.904 | 0.0281 | 0.9866 | 0.5407 | 0.1647 | 0.2351 | 0.1491 | 0.2846 | 1 | 0.886574 | high |
| TCGA-B0-4839 | 0.9788 | 0.2243 | 0.9901 | 0.4157 | 0.339 | 0 | 0.47979 | 0.0195 | 1 | 2.767146 | high |
| TCGA-B8-5553 | 0.9846 | 0.0059 | 0.9846 | 0.5557 | 0.2052 | 0.3508 | 0.6205 | 0.2179 | 1 | 0.332692 | low |
| TCGA-CJ-4893 | 0.9722 | 0.0203 | 0.9841 | 0.4413 | 1 | 0.2127 | 0.6599 | 0.1857 | 0.9738 | 1.258427 | high |
| TCGA-CJ-6033 | 0.9945 | 0.0612 | 0.9906 | 0.4107 | 0.6829 | 0.08916 | 0.8574 | 0.035 | 1 | 1.232582 | high |
| TCGA-B0-4827 | 0.992 | 0.0093 | 0.9895 | 0.4362 | 0.7818 | 0.4976 | 0.313 | 0.3588 | 1 | 0.619592 | low |
| TCGA-BP-4977 | 0.9105 | 0.0645 | 0.9722 | 0.4393 | 0.4219 | 0.2073 | 0.6429 | 0.4904 | 0.9937 | 0.826188 | low |
| TCGA-BP-5187 | 0.9475 | 0.0781 | 0.9787 | 0.4088 | 0.7078 | 0.15037 | 0.1022 | 0.2106 | 1 | 2.649096 | high |
| TCGA-CJ-4882 | 0.872 | 0.123 | 0.9804 | 0.3107 | 0.7788 | 0.023 | 0.7804 | 0.294 | 1 | 3.240417 | high |
| TCGA-CJ-4899 | 0.8954 | 0.0257 | 0.9868 | 0.4889 | 0.2016 | 0.238 | 0.6371 | 0.2803 | 0.9896 | 0.690912 | low |
| TCGA-B8-4620 | 0.9229 | 0.0831 | 0.9487 | 0.3973 | 1 | 0.2168 | 0.2845 | 0.0461 | 0.9854 | 4.625987 | high |
| TCGA-CJ-4868 | 0.9624 | 0.0376 | 0.9822 | 0.3925 | 1 | 0 | 0.4167 | 0.131 | 1 | 2.92028 | high |
| TCGA-BP-5001 | 0.864 | 0.0627 | 0.9918 | 0.5873 | 0 | 0.0048 | 0.7321 | 0.4267 | 0.570134 | 1.517849 | high |
| TCGA-DV-5575 | 0.9782 | 0.0247 | 0.9849 | 0.4829 | 0.2178 | 0.6776 | 0.6473 | 0.2372 | 1 | 0.259668 | low |
| TCGA-CJ-4920 | 0.9863 | 0.0275 | 0.9906 | 0.4902 | 1 | 0.4538 | 0.6349 | 0.2717 | 1 | 0.588433 | low |
| TCGA-B0-4707 | 0.9649 | 0.1257 | 0.986 | 0.4413 | 0.8486 | 0.0536 | 0.41872 | 0.0182 | 1 | 3.039313 | high |
| TCGA-AK-3426 | 0.8301 | 0.1338 | 0.9876 | 0.4708 | 1 | 0.1656 | 0.0919 | 0.3636 | 1 | 4.091083 | high |
| TCGA-B0-4713 | 0.9663 | 0.0747 | 0.9759 | 0.4931 | 0.3407 | 0.1452 | 0.52609 | 0.3876 | 0.558337 | 2.03366 | high |
| TCGA-B0-5705 | 0.9765 | 0.0157 | 0.9775 | 0.5648 | 0.0631 | 0.1928 | 0.7308 | 0.3285 | 1 | 0.314687 | low |
| TCGA-BP-5199 | 0.8478 | 0.1058 | 0.9858 | 0.3982 | 0.0899 | 0.1952 | 0.32 | 0.1111 | 0.8982 | 3.034965 | high |
| TCGA-CZ-5988 | 0.9504 | 0.0361 | 0.987 | 0.5601 | 0.3355 | 0.25706 | 0.4568 | 0.5995 | 1 | 0.354989 | low |
| TCGA-BP-5004 | 0.9054 | 0.0236 | 0.9837 | 0.5955 | 0.2399 | 0.3279 | 0.6249 | 0.5257 | 0.9838 | 0.313077 | low |
| TCGA-A3-3370 | 0.8963 | 0.059 | 0.9818 | 0.4918 | 0.0802 | 0.1128 | 0.4767 | 0.443 | 0.9947 | 0.784404 | low |
| TCGA-B0-5696 | 0.9921 | 0.0213 | 0.9832 | 0.5087 | 0.2921 | 0.13201 | 0.6839 | 0.1785 | 1 | 0.580755 | low |
| TCGA-BP-4797 | 1 | 0.0324 | 0.9746 | 0.5529 | 0.4358 | 0.203 | 0.5268 | 0.1286 | 1 | 0.673885 | low |
| TCGA-CZ-5982 | 0.9868 | 0.0658 | 0.9816 | 0.4705 | 0.4012 | 0.26894 | 0.7068 | 0.2315 | 0.9822 | 0.636497 | low |
| TCGA-BP-4991 | 0.8894 | 0.0692 | 0.984 | 0.4374 | 0.2848 | 0.3021 | 0.4943 | 0.419 | 0.949 | 0.956302 | high |
| TCGA-BP-4762 | 0.9927 | 0.0147 | 0.9886 | 0.4667 | 0.2962 | 0 | 0.49233 | 0.2131 | 1 | 0.881895 | low |
| TCGA-A3-3362 | 0.8903 | 0.0848 | 0.9946 | 0.4754 | 0.1013 | 0 | 0.8123 | 0.391 | 1 | 0.775355 | low |
| TCGA-A3-A8OW | 0.9502 | 0.0728 | 0.9899 | 0.5738 | 0.072 | 0 | 0.648 | 0.3779 | 1 | 0.515816 | low |
| TCGA-CJ-4872 | 0.9525 | 0.1259 | 0.9906 | 0.4212 | 0.6717 | 0.1665 | 0.1444 | 0.1535 | 0.9728 | 2.804744 | high |
| TCGA-B0-4847 | 0.7071 | 0.1249 | 0.991 | 0.5313 | 0.0256 | 0.0596 | 0.4673 | 0.3467 | 0.572171 | 5.969036 | high |
| TCGA-B0-4822 | 0.8704 | 0.1963 | 0.9784 | 0.3143 | 1 | 0.1887 | 0.41957 | 0.2721 | 1 | 5.505438 | high |
| TCGA-BP-5175 | 0.9581 | 0.0992 | 0.9797 | 0.4319 | 0.9164 | 0.0572 | 0.7736 | 0.2174 | 1 | 1.703854 | high |
| TCGA-BP-4340 | 0.887 | 0.0556 | 1 | 0.6405 | 0.1179 | 0.113 | 0.7645 | 0.5774 | 0.9539 | 0.322773 | low |
| TCGA-BP-4987 | 0.9248 | 0.019 | 0.9873 | 0.4126 | 0.0861 | 0.2607 | 0.7381 | 0.4227 | 1 | 0.471772 | low |
| TCGA-BP-5189 | 0.9921 | 0.0136 | 0.992 | 0.4369 | 0.715 | 0.3467 | 0.2625 | 0.055 | 0.9891 | 1.239576 | high |
| TCGA-BP-5000 | 0.9321 | 0.0454 | 0.9846 | 0.4273 | 0.3412 | 0 | 0.3968 | 0.1677 | 1 | 1.775902 | high |
| TCGA-AK-3451 | 1 | 0.3252 | 0.9916 | 0.3537 | 0.42808 | 0.11567 | 0.63275 | 0.6 | 1 | 1.329494 | high |
| TCGA-BP-5007 | 0.9303 | 0.088 | 0.9881 | 0.4361 | 0.0799 | 0.2851 | 0.4767 | 0.3687 | 0.9632 | 0.765638 | low |
| TCGA-A3-3363 | 0.9272 | 0.0322 | 0.9907 | 0.574 | 0.0815 | 0.3531 | 0.4928 | 0.1176 | 1 | 0.504858 | low |
| TCGA-AK-3455 | 0.9692 | 0 | 0.9901 | 0.5148 | 0 | 0 | 0.6349 | 0.6017 | 0.9539 | 0.351638 | low |
| TCGA-B8-4146 | 0.8921 | 0.0627 | 0.9396 | 0.4702 | 0 | 0.15225 | 0.6567 | 0.4419 | 1 | 0.819463 | low |
| TCGA-BP-4770 | 0.9772 | 0.0435 | 0.7671 | 0.49 | 0.448 | 0.0765 | 0.35446 | 0.0365 | 1 | 5.62938 | high |
| TCGA-A3-3378 | 0.9438 | 0.0498 | 0.9902 | 0.451 | 0.8471 | 0.0776 | 0.503 | 0.0627 | 1 | 2.101551 | high |
| TCGA-DV-5573 | 0.9483 | 0.0474 | 0.9762 | 0.3963 | 0.1654 | 0.1965 | 0.6375 | 0.177 | 1 | 0.962496 | high |
| TCGA-BP-4962 | 0.9907 | 0.0466 | 0.9922 | 0.5043 | 0.2151 | 0 | 1 | 0.2645 | 0.9785 | 0.470283 | low |
| TCGA-B8-5546 | 1 | 0.0067 | 0.9686 | 0.5192 | 0.8016 | 0.24143 | 0.503 | 0.0255 | 1 | 1.06503 | high |
| TCGA-BP-4347 | 0.8999 | 0.0364 | 0.9716 | 0.5127 | 0.3215 | 0.0608 | 0.4685 | 0.2714 | 0.9756 | 1.274333 | high |
| TCGA-A3-3343 | 0.9537 | 0.024 | 0.9858 | 0.382 | 0.0242 | 0.1587 | 0.5281 | 0.4979 | 0.9658 | 0.621249 | low |
| TCGA-CJ-4918 | 0.9397 | 0.017 | 0.979 | 0.4336 | 0.4947 | 0.5047 | 0.327 | 0.0619 | 1 | 1.040075 | high |
| TCGA-BP-5201 | 0.9849 | 0.0374 | 0.984 | 0.4766 | 0.2586 | 0.0336 | 0.3434 | 0.1927 | 0.9168 | 1.303362 | high |
| TCGA-CZ-5459 | 0.9646 | 0.0477 | 0.9806 | 0.4562 | 0.3392 | 0.3786 | 0.178 | 0.4228 | 0.9804 | 0.728597 | low |
| TCGA-BP-5170 | 0.9958 | 0.0666 | 0.9852 | 0.3877 | 0.7315 | 0.6903 | 0.1318 | 0.0267 | 1 | 1.189881 | high |
| TCGA-B0-5094 | 0.9312 | 0.0563 | 0.9636 | 0.483 | 0.7874 | 0.0407 | 0.4989 | 0.1829 | 1 | 2.070242 | high |
| TCGA-BP-4973 | 0.8713 | 0.0648 | 0.9926 | 0.4448 | 0.0856 | 0.155 | 0.8455 | 0.38 | 0.9694 | 0.725993 | low |
| TCGA-CJ-5684 | 0.9776 | 0 | 0.9727 | 0.4494 | 0.0765 | 0 | 0.5301 | 0.4319 | 1 | 0.622172 | low |
| TCGA-BP-4771 | 0.7796 | 0.07 | 0.9824 | 0.463 | 0.4951 | 0.1038 | 0.2474 | 0.305 | 0.9771 | 3.309028 | high |
| TCGA-B0-5116 | 0.982 | 0.0117 | 0.9849 | 0.5117 | 0.7408 | 0.4592 | 0.3302 | 0.4034 | 1 | 0.507164 | low |
| TCGA-CW-5585 | 0.9737 | 0.0111 | 0.9774 | 0.5223 | 0.0365 | 0 | 0.88 | 0.3994 | 1 | 0.365702 | low |
| TCGA-CJ-4881 | 0.9058 | 0.0542 | 0.9793 | 0.5195 | 0.8446 | 0.0892 | 0.7223 | 0.1416 | 0.9323 | 1.804618 | high |
| TCGA-A3-3323 | 0.8877 | 0.0431 | 0.9935 | 0.4242 | 0.3094 | 0.095 | 0.6485 | 0.39 | 0.9605 | 1.067813 | high |
| TCGA-A3-3380 | 0.9315 | 0.072 | 0.9909 | 0.5261 | 0.0952 | 0 | 0.842 | 0.2322 | 1 | 0.664778 | low |
| TCGA-B0-4834 | 0.9649 | 0.0541 | 0.9888 | 0.5531 | 0.1545 | 0.3375 | 0.4576 | 0.0377 | 0.99223 | 0.628187 | low |
| TCGA-AK-3450 | 0.9564 | 0.0304 | 0.9892 | 0.5138 | 0.141 | 0 | 0.784 | 0.3878 | 1 | 0.483261 | low |
| TCGA-CZ-5461 | 1 | 0.0136 | 0.9829 | 0.4088 | 0.3289 | 0.4477 | 0.6212 | 0.2151 | 1 | 0.47349 | low |
| TCGA-A3-3347 | 0.8773 | 0.0465 | 0.9724 | 0.373 | 0.7697 | 0.1952 | 0.8808 | 0.0815 | 0.9692 | 2.227216 | high |
| TCGA-B0-5110 | 0.9749 | 0.0663 | 0.986 | 0.4994 | 0.1797 | 0.203 | 0.7506 | 0.3651 | 0.943 | 0.483494 | low |
| TCGA-AK-3460 | 0.9746 | 0.0485 | 0.961 | 0.5325 | 0 | 0 | 0.6193 | 0.2873 | 1 | 0.648462 | low |
| TCGA-CJ-4908 | 0.9431 | 0 | 0.9777 | 0.4787 | 0.2738 | 0 | 0.5676 | 0.183 | 0.9802 | 1.086086 | high |
| TCGA-BP-4774 | 0.92 | 0.1393 | 0.9854 | 0.5643 | 0.2776 | 0.0239 | 0.6657 | 0.3086 | 0.943 | 1.060313 | high |
| TCGA-B0-4842 | 1 | 0.1563 | 0.983 | 0.5062 | 0.1183 | 0 | 0.47713 | 0.0133 | 1 | 1.406271 | high |
| TCGA-B0-5812 | 0.9662 | 0.0169 | 0.9878 | 0.4533 | 0.1114 | 0.124 | 0.3779 | 0.2779 | 1 | 0.765781 | low |
| TCGA-B0-4816 | 0.9409 | 0.0303 | 0.9815 | 0.6207 | 0.3255 | 0.0648 | 0.1482 | 0.2301 | 0.9813 | 1.020918 | high |
| TCGA-BP-4988 | 0.6539 | 0.2234 | 0.9466 | 0.4964 | 0.4045 | 0.0809 | 0.5852 | 0.4715 | 0.918 | 6.364743 | high |
| TCGA-B0-4945 | 0.8557 | 0.0313 | 0.9933 | 0.5081 | 0.0834 | 0 | 0.4924 | 0.3296 | 1 | 1.052384 | high |
| TCGA-DV-5576 | 0.9627 | 0.0537 | 0.9794 | 0.477 | 0.2069 | 0 | 0.5875 | 0.1569 | 0.9254 | 1.27025 | high |
| TCGA-MM-A84U | 0.9175 | 0.0918 | 0.9914 | 0.5354 | 0.3686 | 0 | 0 | 0 | 1 | 2.91978 | high |
| TCGA-BP-4998 | 0.917 | 0.0355 | 0.9872 | 0.4732 | 0.5664 | 0.3099 | 0.6836 | 0.183 | 0.9804 | 0.896826 | high |
| TCGA-A3-3387 | 0.9766 | 0.0863 | 0.9886 | 0.4306 | 0.6659 | 0.3261 | 0.6931 | 0.2021 | 1 | 0.868882 | low |
| TCGA-BP-4968 | 0.9483 | 0.1027 | 0.9837 | 0.5053 | 0.1121 | 0 | 0.4651 | 0.4036 | 1 | 0.863877 | low |
| TCGA-BP-4995 | 0.9669 | 0.0315 | 0.9615 | 0.4369 | 0.2454 | 0.2485 | 0.7813 | 0.3166 | 0.9857 | 0.587873 | low |
| TCGA-CJ-4890 | 0.967 | 0.0204 | 0.9809 | 0.4512 | 0.3665 | 0.1618 | 0.3675 | 0.1621 | 0.975 | 1.176423 | high |
| TCGA-B0-5092 | 0.7816 | 0.1977 | 0.9863 | 0.4604 | 0.2328 | 0.1097 | 0.1855 | 0.2871 | 0.7338 | 7.268811 | high |
| TCGA-DV-A4VZ | 0.8776 | 0.2523 | 0.9941 | 0.4525 | 0.3452 | 0.0896 | 0.3485 | 0.1067 | 0.9385 | 4.184243 | high |
| TCGA-A3-3385 | 0.9241 | 0.0469 | 0.9729 | 0.5129 | 0.2518 | 0.1452 | 0.9296 | 0.4294 | 1 | 0.46057 | low |
| TCGA-B0-4823 | 1 | 0.0235 | 0.9891 | 0.481 | 0.2577 | 0.24009 | 0.3501 | 0.2155 | 1 | 0.645326 | low |
| TCGA-BP-4992 | 0.5442 | 0.147 | 0.9923 | 0.5046 | 0.5568 | 0 | 0.8555 | 0.3664 | 0.9566 | 6.328416 | high |
| TCGA-B8-4151 | 0.9701 | 0.0365 | 0.9606 | 0.5289 | 0.0176 | 0.298 | 0.8318 | 0.5359 | 1 | 0.233639 | low |
| TCGA-B4-5838 | 0.9827 | 0.0101 | 0.9804 | 0.5614 | 0.6769 | 0.20157 | 0.8117 | 0.1968 | 1 | 0.526255 | low |
| TCGA-BP-4986 | 0.8943 | 0.0298 | 0.9856 | 0.4229 | 0.7298 | 0.1503 | 0.9173 | 0.1539 | 0.9946 | 1.326218 | high |
| TCGA-CZ-5984 | 1 | 0.0302 | 0.9814 | 0.4537 | 0.5515 | 0 | 0.9251 | 0.0602 | 0.992 | 0.973486 | high |
| TCGA-BP-4344 | 0.9353 | 0.0638 | 0.9743 | 0.4033 | 0.1779 | 0 | 0.1803 | 0.177 | 1 | 2.288785 | high |
| TCGA-CZ-4854 | 0.8931 | 0.0891 | 0.9814 | 0.4561 | 0.8699 | 0.0752 | 0.7469 | 0.1833 | 0.9712 | 2.206482 | high |
| TCGA-CJ-6032 | 0.9649 | 0 | 0.9706 | 0.5323 | 0.3285 | 0 | 0.7253 | 0.3409 | 1 | 0.592533 | low |
| TCGA-BP-4355 | 0.8355 | 0.102 | 0.9934 | 0.4022 | 0.1203 | 0.2668 | 0.0919 | 0.2818 | 1 | 2.123558 | high |
| TCGA-B0-4814 | 0.9358 | 0.1095 | 0.9925 | 0.4342 | 0.1284 | 0.1592 | 0.3338 | 0.3804 | 1 | 1.021118 | high |
| TCGA-BP-4759 | 0.9823 | 0.0399 | 0.9819 | 0.4127 | 0.2607 | 0.3891 | 0 | 0.286 | 1 | 0.960512 | high |
| TCGA-B0-5097 | 0.9743 | 0 | 0.9793 | 0.3742 | 0.8758 | 0 | 0.1163 | 0.156 | 1 | 3.162162 | high |
| TCGA-CJ-4923 | 0.9187 | 0.1083 | 0.9557 | 0.3395 | 1 | 0.0239 | 0.42889 | 0.2443 | 1 | 4.869055 | high |
| TCGA-DV-A4VX | 0.9354 | 0.0974 | 0.9794 | 0.5641 | 0.40379 | 0 | 0.542356 | 0.1586 | 1 | 1.282616 | high |
| TCGA-CJ-5671 | 0.9917 | 0.0413 | 0.9774 | 0.4522 | 0.9038 | 0.6903 | 0.6103 | 0.1574 | 1 | 0.568823 | low |
| TCGA-BP-5191 | 0.9405 | 0 | 0.9792 | 0.4623 | 0.3812 | 0 | 0.57429 | 0.3164 | 1 | 0.969828 | high |
| TCGA-BP-4158 | 0.9118 | 0.1144 | 0.9892 | 0.5257 | 0.0163 | 0 | 0.6777 | 0.4214 | 1 | 0.700468 | low |
| TCGA-BP-4768 | 0.9523 | 0.0794 | 0.9901 | 0.4176 | 0.4881 | 0.2359 | 0.9249 | 0.3365 | 0.986 | 0.660356 | low |
| TCGA-B2-5639 | 0.9707 | 0.0174 | 0.9702 | 0.5423 | 0.1073 | 0.4764 | 0.7831 | 0.2358 | 1 | 0.263996 | low |
| TCGA-B0-5084 | 0.9019 | 0.1019 | 0.9867 | 0.3965 | 0.8818 | 0.045 | 0.5634 | 0.1208 | 1 | 3.315579 | high |
| TCGA-CJ-5680 | 1 | 0.0184 | 0.9896 | 0.4325 | 0.819 | 0.1539 | 0.9484 | 0.1844 | 1 | 0.738209 | low |
| TCGA-CJ-4639 | 0.9199 | 0.0888 | 0.9942 | 0.5429 | 0.0408 | 0.1326 | 0.367 | 0.2134 | 1 | 0.886623 | high |
| TCGA-B8-5159 | 0.988 | 0.0096 | 0.9906 | 0.5481 | 0.0624 | 0.3702 | 0.755 | 0.1829 | 1 | 0.261286 | low |
| TCGA-CJ-4641 | 0.8701 | 0.0421 | 0.9713 | 0.5231 | 0.2935 | 0.0845 | 0.4633 | 0.3546 | 0.9904 | 1.168055 | high |
| TCGA-B0-5692 | 0.9842 | 0.0231 | 0.9866 | 0.5185 | 0 | 0.3578 | 0.596 | 0.3799 | 1 | 0.264861 | low |
| TCGA-CJ-4895 | 0.9164 | 0.0309 | 0.982 | 0.4587 | 0.8799 | 0 | 0.2455 | 0.0914 | 0.9865 | 3.420684 | high |
| TCGA-BP-4959 | 0.9811 | 0.0324 | 0.988 | 0.5385 | 0.4281 | 0.4669 | 0.8299 | 0.3042 | 0.9946 | 0.270266 | low |
| TCGA-BP-4787 | 0.8509 | 0.0812 | 0.9738 | 0.3865 | 1 | 0 | 0.6383 | 0.1512 | 1 | 4.558896 | high |
| TCGA-AK-3465 | 0.9321 | 0.0451 | 0.9781 | 0.5624 | 0.0952 | 0 | 0.9002 | 0.2865 | 1 | 0.518974 | low |
| TCGA-BP-5009 | 0.9313 | 0.0388 | 0.9817 | 0.4917 | 0.8829 | 0.0954 | 0.2818 | 0.1476 | 0.9794 | 2.323402 | high |
| TCGA-EU-5904 | 0.9872 | 0.0584 | 0.996 | 0.4684 | 0.2574 | 0.161 | 0.5609 | 0.4316 | 1 | 0.502226 | low |
| TCGA-A3-3306 | 0.9622 | 0.0527 | 0.9946 | 0.4225 | 0.2445 | 0.0783 | 1 | 0.1186 | 1 | 0.730607 | low |
| TCGA-BP-5192 | 0.9858 | 0.057 | 0.9839 | 0.4559 | 0.0747 | 0.124 | 0.4385 | 0.5261 | 0.9718 | 0.545244 | low |
| TCGA-BP-4784 | 0.9701 | 0 | 0.9868 | 0.3983 | 0.2931 | 0.096 | 0.51501 | 0.0598 | 1 | 1.220765 | high |
| TCGA-B8-4153 | 0.9393 | 0.1128 | 0.9694 | 0.466 | 0 | 0.05968 | 0.5394 | 0.3773 | 1 | 0.919935 | high |
| TCGA-B0-5081 | 0.8385 | 0.0827 | 0.9692 | 0.5003 | 0.2187 | 0.0547 | 0.1622 | 0.2829 | 0.9666 | 2.575738 | high |
| TCGA-G6-A8L6 | 0.8961 | 0.3436 | 0.9933 | 0.4269 | 0.3441 | 0 | 0.1852 | 0.2826 | 1 | 4.944416 | high |
| TCGA-AK-3445 | 0.7217 | 0 | 0.9864 | 0.4313 | 0.3891 | 0 | 0.44507 | 0.1069 | 1 | 4.214012 | high |
| TCGA-BP-4351 | 0.8557 | 0.2471 | 0.9886 | 0.4604 | 0.1869 | 0.0685 | 0.2183 | 0.4661 | 0.9381 | 2.841204 | high |
| TCGA-B0-5100 | 0.9855 | 0.0715 | 0.996 | 0.4656 | 0 | 0 | 0.39219 | 0.0278 | 0.554924 | 3.322759 | high |
| TCGA-B0-5700 | 0.9947 | 0.0407 | 0.9842 | 0.4806 | 0.1093 | 0.08909 | 0.57502 | 0.4442 | 1 | 0.461986 | low |
| TCGA-AK-3429 | 1 | 0 | 0.9736 | 0.4517 | 0.0731 | 0.2144 | 0 | 0.3008 | 1 | 0.820783 | low |
| TCGA-BP-4798 | 0.8903 | 0.1125 | 0.9277 | 0.3348 | 0.0878 | 0.1274 | 0.5868 | 0.1226 | 0.9762 | 3.091647 | high |
| TCGA-BP-4769 | 0.9617 | 0.0054 | 0.9881 | 0.4169 | 0.6523 | 0.1624 | 0.3442 | 0.1016 | 0.965 | 1.737461 | high |
| TCGA-CJ-5678 | 0.9891 | 0.1021 | 0.9905 | 0.5144 | 0.1029 | 0.07516 | 0.52449 | 0.2673 | 0.9323 | 0.79646 | low |
| TCGA-A3-3316 | 0.958 | 0.0416 | 0.9507 | 0.4386 | 0.8482 | 0 | 0 | 0.1697 | 0.9872 | 4.201058 | high |
| TCGA-B8-5550 | 0.9848 | 0.0297 | 0.9862 | 0.586 | 0.6468 | 0 | 0.2845 | 0.0616 | 1 | 1.36023 | high |
| TCGA-B0-5699 | 0.9881 | 0.0123 | 0.9587 | 0.517 | 0.1712 | 0.298 | 0.6584 | 0.4278 | 1 | 0.329808 | low |
| TCGA-CJ-4888 | 0.944 | 0.0245 | 0.9728 | 0.4359 | 0.4143 | 0 | 0.4059 | 0.087 | 0.9741 | 2.078652 | high |
| TCGA-BP-4354 | 0.8708 | 0.0337 | 0.9528 | 0.338 | 0.8778 | 0 | 0.2301 | 0.0533 | 0.9943 | 7.664043 | high |
| TCGA-BP-4989 | 0.866 | 0.1812 | 0.977 | 0.4498 | 0.3506 | 0.1431 | 0.2918 | 0.1003 | 0.9213 | 4.129981 | high |
| TCGA-CJ-4873 | 0.8743 | 0 | 0.9776 | 0.5478 | 0.479 | 0.012 | 0.5774 | 0.2346 | 0.943 | 1.3823 | high |
| TCGA-B0-4824 | 0.853 | 0.1048 | 0.9811 | 0.5278 | 0 | 0 | 0.6675 | 0.477 | 1 | 0.852422 | low |
| TCGA-CZ-5455 | 0.9725 | 0.0116 | 0.9689 | 0.5035 | 0.3318 | 0.3643 | 0.7908 | 0.3524 | 0.9938 | 0.349233 | low |
| TCGA-BP-4164 | 0.8984 | 0 | 0.9813 | 0.5266 | 0.0067 | 0 | 0.4873 | 0.2059 | 1 | 0.917957 | high |
| TCGA-CW-5581 | 0.9663 | 0.005 | 0.98 | 0.4346 | 0.339 | 0.6443 | 0.7859 | 0.2851 | 1 | 0.288756 | low |
| TCGA-A3-3313 | 0.9424 | 0.0197 | 0.9804 | 0.5809 | 0.5755 | 0.113 | 0.8908 | 0.202 | 0.965 | 0.64455 | low |
| TCGA-B0-4845 | 0.9395 | 0.1468 | 0.9808 | 0.4635 | 0.2527 | 0.1344 | 0.4826 | 0.3391 | 0.9018 | 1.445996 | high |
| TCGA-B4-5378 | 0.9732 | 0.0284 | 0.9739 | 0.402 | 0.1585 | 0.5124 | 0.4299 | 0.0666 | 1 | 0.719058 | low |
| TCGA-BP-5174 | 0.9741 | 0.0527 | 0.9885 | 0.461 | 0.3655 | 0.1055 | 0.8766 | 0.4582 | 1 | 0.465354 | low |
| TCGA-BP-4801 | 0.9907 | 0.014 | 0.9822 | 0.4463 | 0.2897 | 0 | 0.5913 | 0.3212 | 0.9934 | 0.767827 | low |
| TCGA-CJ-5686 | 0.9782 | 0.0245 | 0.9824 | 0.4702 | 0.2627 | 0.2479 | 0.4613 | 0.1928 | 0.9921 | 0.71771 | low |
| TCGA-A3-3349 | 0.9261 | 0 | 0.9821 | 0.4641 | 0.6415 | 0.298 | 0.2715 | 0.2219 | 1 | 1.212113 | high |
| TCGA-DV-5567 | 0.9707 | 0.0226 | 0.9907 | 0.4752 | 0.3035 | 0.5696 | 0.3864 | 0.2163 | 1 | 0.444546 | low |
| TCGA-CZ-5987 | 0.9856 | 0.0221 | 0.9869 | 0.4444 | 0.2932 | 0 | 0.686 | 0.2369 | 1 | 0.797799 | low |
| TCGA-CJ-4885 | 0.9763 | 0.0178 | 0.9985 | 0.5862 | 0.1121 | 0.2272 | 0.3561 | 0.1278 | 0.9694 | 0.537447 | low |
| TCGA-DV-5574 | 0.9816 | 0.0233 | 0.9843 | 0.5066 | 0.5033 | 0.7977 | 0.4005 | 0.0765 | 1 | 0.398471 | low |
| TCGA-A3-3331 | 0.9115 | 0 | 0.9809 | 0.6055 | 0.1245 | 0.122 | 0.5737 | 0.2498 | 0.9931 | 0.543292 | low |
| TCGA-BP-5178 | 1 | 0.0187 | 0.9772 | 0.4238 | 0.6204 | 0.1751 | 0.503 | 0.4791 | 1 | 0.692879 | low |
| TCGA-CJ-4871 | 1 | 0.0356 | 0.9779 | 0.5013 | 0.8582 | 0.589 | 0.4551 | 0.1031 | 1 | 0.661797 | low |
| TCGA-B0-4836 | 0.9574 | 0.0118 | 0.9546 | 0.4761 | 0.8967 | 0 | 0.3942 | 0.0769 | 0.943 | 2.98644 | high |
| TCGA-B8-A54I | 0.9809 | 0.099 | 0.9802 | 0.5258 | 1 | 0.0866 | 0.5264 | 0.109 | 0.9863 | 1.82507 | high |
| TCGA-B0-4821 | 0.8806 | 0 | 0.8651 | 0.4934 | 0.5807 | 0 | 0.38103 | 0.33 | 1 | 3.25367 | high |
| TCGA-BP-4777 | 0.9291 | 0.0319 | 0.9891 | 0.4641 | 0.1469 | 0.2847 | 0.4436 | 0.2777 | 0.9778 | 0.717923 | low |
| TCGA-CJ-4636 | 0.8658 | 0.0926 | 0.986 | 0.5176 | 0.6121 | 0.0862 | 0.107 | 0.386 | 0.9771 | 2.292021 | high |
| TCGA-B0-5711 | 0.9772 | 0.012 | 0.9809 | 0.509 | 0.0731 | 0.4144 | 0.5308 | 0.2996 | 0.9797 | 0.343813 | low |
| TCGA-CZ-4857 | 0.9047 | 0 | 0.9654 | 0.3828 | 0.764 | 0.298 | 0.4356 | 0.0931 | 1 | 2.121858 | high |
| TCGA-B0-4852 | 0.9101 | 0.0731 | 0.9784 | 0.542 | 0.3094 | 0.3249 | 0.6366 | 0.3114 | 0.9896 | 0.606339 | low |
| TCGA-A3-3329 | 0.9529 | 0.0704 | 0.9842 | 0.5564 | 0.36016 | 0.1392 | 0.5931 | 0.2393 | 0.9894 | 0.736381 | low |
| TCGA-CZ-5466 | 0.9804 | 0.0115 | 0.9797 | 0.546 | 0.5994 | 0.3902 | 0.6025 | 0.1166 | 1 | 0.548933 | low |
| TCGA-BP-5177 | 0.9749 | 0.0687 | 0.989 | 0.3946 | 0.2651 | 0.0783 | 0.1202 | 0.1582 | 0.7861 | 3.08721 | high |
| TCGA-A3-3372 | 0.9188 | 0.0482 | 0.9847 | 0.4916 | 0 | 0.1326 | 0.72 | 0.2563 | 0.9585 | 0.689067 | low |
| TCGA-BP-4804 | 0.8936 | 0.065 | 0.9858 | 0.5206 | 0.1647 | 0.2535 | 0.4252 | 0.1417 | 1 | 1.002179 | high |
| TCGA-AK-3434 | 0.9462 | 0.2207 | 0.9861 | 0.4545 | 0.0621 | 0.124 | 0.46038 | 0.1189 | 1 | 1.706969 | high |
| TCGA-B0-4811 | 0.8927 | 0.1685 | 0.9825 | 0.4717 | 0.2399 | 0 | 0.1955 | 0.2262 | 1 | 2.797099 | high |
| TCGA-BP-4165 | 0.9565 | 0.1897 | 0.9878 | 0.4041 | 0.0316 | 0.1038 | 0.7636 | 0.3889 | 0.988667 | 0.883028 | low |
| TCGA-B0-5099 | 0.9364 | 0.0432 | 0.9947 | 0.4973 | 0.7528 | 0 | 0.4294 | 0.5179 | 1 | 1.049109 | high |
| TCGA-BP-4766 | 0.9321 | 0.0264 | 0.9875 | 0.5909 | 0.4315 | 0.3615 | 0.3059 | 0.6117 | 0.9848 | 0.369376 | low |
| TCGA-BP-4335 | 0.8345 | 0.1819 | 0.9837 | 0.514 | 0.0952 | 0 | 0 | 0.3429 | 0.8464 | 4.324037 | high |
| TCGA-B0-4703 | 0.9086 | 0.0345 | 0.9816 | 0.3902 | 0.8975 | 0.203 | 0.0752 | 0.0358 | 0.9414 | 4.665216 | high |
| TCGA-CJ-4904 | 0.9868 | 0 | 0.9873 | 0.4634 | 0.1452 | 0.4432 | 0.4952 | 0.3051 | 1 | 0.347417 | low |
| TCGA-BP-4352 | 0.79473 | 0.2943 | 0.9787 | 0.3062 | 0.3153 | 0 | 0.2872 | 0.2459 | 1 | 9.87823 | high |
| TCGA-CJ-4635 | 0.9076 | 0.0778 | 0.972 | 0.4636 | 0.6864 | 0.1425 | 0.5308 | 0.2606 | 0.9747 | 1.789057 | high |
| TCGA-CJ-5679 | 0.9203 | 0.0607 | 0.956 | 0.5005 | 0.4878 | 0.15262 | 0.5283 | 0.1071 | 1 | 1.590893 | high |
| TCGA-CZ-4862 | 0.8609 | 0.0558 | 0.9736 | 0.4845 | 0.1459 | 0.298 | 0.4908 | 0.3832 | 1 | 0.82338 | low |
| TCGA-B8-4622 | 0.9321 | 0.0368 | 0.9937 | 0.4973 | 0.4431 | 0.1519 | 0.6122 | 0.4821 | 0.9822 | 0.603497 | low |
| TCGA-G6-A8L8 | 0.9117 | 0.1088 | 0.9891 | 0.5911 | 0.35368 | 0 | 0.5612 | 0.2838 | 1 | 1.007707 | high |
| TCGA-CJ-5682 | 0.9731 | 0.0595 | 0.9947 | 0.4533 | 0.2148 | 0.13011 | 0.5968 | 0.2942 | 1 | 0.677089 | low |
| TCGA-BP-4176 | 0.9623 | 0.0324 | 0.9796 | 0.5187 | 0.8724 | 0.4713 | 0.7384 | 0.0993 | 1 | 0.668705 | low |
| TCGA-B8-4148 | 0.9154 | 0.0228 | 0.9894 | 0.5156 | 0.0193 | 0.0792 | 0.7601 | 0.3481 | 0.952 | 0.551933 | low |
| TCGA-CJ-4886 | 0.958 | 0.0292 | 0.9866 | 0.5317 | 0 | 0.3375 | 0.5534 | 0.3645 | 0.9368 | 0.371306 | low |
| TCGA-BP-5186 | 0.9713 | 0.0296 | 0.9765 | 0.4959 | 0.307 | 0.1494 | 0.6304 | 0.5196 | 1 | 0.447261 | low |
| TCGA-BP-4972 | 0.9603 | 0.0462 | 0.9859 | 0.4237 | 0.2763 | 0.2544 | 0.494 | 0.4575 | 0.993 | 0.612197 | low |
| TCGA-BP-4993 | 0.988 | 0.0574 | 0.9918 | 0.4492 | 0.264 | 0.1503 | 0.5365 | 0.2627 | 1 | 0.726368 | low |
| TCGA-CW-6097 | 1 | 0.0153 | 0.9841 | 0.3554 | 0.7288 | 0.27151 | 0.1717 | 0.0587 | 0.982 | 2.024857 | high |
| TCGA-B0-5077 | 0.9178 | 0.0344 | 0.9679 | 0.4387 | 0.2262 | 0.4002 | 0.2728 | 0.3223 | 1 | 0.88013 | low |
| TCGA-A3-3383 | 0.9444 | 0.1433 | 0.9897 | 0.5257 | 0.38465 | 0.01948 | 0.567489 | 0.1481 | 1 | 1.403339 | high |
| TCGA-B0-4819 | 0.8809 | 0.0964 | 0.9882 | 0.4892 | 0.2604 | 0 | 0.2882 | 0.1605 | 1 | 2.230186 | high |
| TCGA-BP-4775 | 0.9854 | 0 | 0.9705 | 0.5628 | 0.1738 | 0.1038 | 0.7417 | 0.3409 | 1 | 0.367884 | low |
| TCGA-BP-4327 | 0.8648 | 0.0823 | 0.9847 | 0.3818 | 0.5569 | 0 | 0.3026 | 0.2902 | 1 | 3.319102 | high |
| TCGA-B0-5399 | 0.9371 | 0.1075 | 0.9594 | 0.4943 | 0.0506 | 0 | 0.2216 | 0.3271 | 0.9507 | 1.688651 | high |
| TCGA-A3-3357 | 0.8676 | 0.0431 | 0.986 | 0.4808 | 0.6624 | 0.4212 | 0.6147 | 0.4757 | 1 | 0.68324 | low |
| TCGA-B0-4694 | 0.9219 | 0.0705 | 0.9757 | 0.4363 | 0.8318 | 0.1618 | 0.4059 | 0.2461 | 0.9913 | 2.105217 | high |
| TCGA-A3-3317 | 0.856 | 0.081 | 0.9856 | 0.426 | 0.7075 | 0.2367 | 0.8996 | 0.1076 | 1 | 1.676533 | high |
| TCGA-B8-4154 | 0.9714 | 0.0209 | 0.9911 | 0.5819 | 0.0101 | 0 | 0.4865 | 0.3871 | 1 | 0.432219 | low |
| TCGA-B2-5636 | 0.9868 | 0.0321 | 0.9842 | 0.4147 | 0.5108 | 0.1928 | 0.3552 | 0.0656 | 0.9785 | 1.497838 | high |
| TCGA-B0-4817 | 0.966 | 0.0468 | 0.9915 | 0.4682 | 0.4141 | 0 | 0.46483 | 0.2964 | 1 | 1.063146 | high |
| TCGA-BP-5010 | 0.9321 | 0.1773 | 0.9739 | 0.5039 | 0.4381 | 0.0661 | 0.5605 | 0.2737 | 1 | 1.567932 | high |
| TCGA-BP-4325 | 0.9566 | 0.093 | 0.9794 | 0.5444 | 0.0463 | 0.0591 | 0.8955 | 0.5518 | 0.9718 | 0.358784 | low |
| TCGA-BP-5008 | 0.9436 | 0.0732 | 0.9938 | 0.4066 | 0.1117 | 0.4188 | 0.6314 | 0.3934 | 0.9805 | 0.482482 | low |
| TCGA-B0-5707 | 0.9456 | 0.0092 | 0.9748 | 0.511 | 0.2486 | 0.16493 | 0.8144 | 0.0635 | 0.9141 | 0.840524 | low |
| TCGA-CZ-5454 | 0.9808 | 0.0443 | 0.9791 | 0.3328 | 0.4212 | 0.4491 | 0.7475 | 0.1339 | 0.9929 | 0.783138 | low |
| TCGA-BP-4807 | 0.981 | 0.0076 | 0.9835 | 0.5267 | 0.2892 | 0.5149 | 0.6733 | 0.5336 | 1 | 0.185185 | low |
| TCGA-A3-3352 | 0.8854 | 0.0539 | 0.9841 | 0.4534 | 0.1188 | 0 | 0.5411 | 0.2075 | 1 | 1.434606 | high |
| TCGA-B0-5712 | 0.9716 | 0.0338 | 0.9937 | 0.5773 | 0.2266 | 0.113 | 0.7203 | 0.1919 | 1 | 0.46643 | low |
| TCGA-B0-4843 | 0.8755 | 0.2557 | 0.9785 | 0.3619 | 0.0452 | 0 | 0.2146 | 0.363 | 0.563191 | 10.53501 | high |
| TCGA-B8-A54H | 0.8937 | 0.078 | 0.9854 | 0.5159 | 0.2905 | 0 | 0.6211 | 0.4646 | 0.9758 | 0.933422 | high |
| TCGA-CZ-5456 | 1 | 0.0104 | 0.9842 | 0.4096 | 0.7174 | 0.4332 | 0.6119 | 0.2193 | 1 | 0.640697 | low |
| TCGA-B0-4828 | 0.8633 | 0.0645 | 0.9954 | 0.4658 | 0.1267 | 0 | 0 | 0.1779 | 0.9539 | 2.947564 | high |
| TCGA-AK-3444 | 0.9764 | 0.0125 | 0.9846 | 0.4633 | 0.1425 | 0.5928 | 0.5476 | 0.4281 | 1 | 0.241553 | low |
| TCGA-CJ-6031 | 0.9918 | 0.0646 | 0.9857 | 0.4541 | 0.4019 | 0.6903 | 0.1173 | 0.1934 | 0.9681 | 0.655483 | low |
| TCGA-B8-A54K | 0.946 | 0.0665 | 0.9855 | 0.4933 | 0.2182 | 0.2776 | 0 | 0.1347 | 0.951 | 1.504436 | high |
| TCGA-DV-5566 | 0.9829 | 0.014 | 0.9739 | 0.5813 | 0.2198 | 0.13951 | 0.7852 | 0.3237 | 0.9254 | 0.410427 | low |
| TCGA-B0-5113 | 0.9523 | 0.0827 | 0.9956 | 0.4755 | 0.3338 | 0.1452 | 0.539 | 0.1244 | 0.9918 | 1.094816 | high |
| TCGA-BP-4169 | 0.9141 | 0 | 0.9852 | 0.3991 | 0.66 | 0.1097 | 0 | 0.1939 | 0.9785 | 2.966289 | high |
| TCGA-CZ-5986 | 0.9619 | 0.0292 | 0.9695 | 0.552 | 0.0148 | 0 | 0.5408 | 0.3652 | 1 | 0.567105 | low |
| TCGA-CJ-4640 | 0.9671 | 0 | 0.9892 | 0.5641 | 1 | 0.5271 | 0.6858 | 0.2782 | 0.9591 | 0.44236 | low |
| TCGA-B4-5836 | 0.9757 | 0.0087 | 0.9921 | 0.5121 | 0.2143 | 0.3232 | 0.513 | 0.3712 | 0.9658 | 0.384497 | low |
| TCGA-B0-5713 | 0.9631 | 0.0396 | 0.991 | 0.5822 | 0.0399 | 0.1965 | 0.7198 | 0.4415 | 0.965 | 0.284031 | low |
| TCGA-BP-4790 | 0.8644 | 0.0212 | 0.9594 | 0.4615 | 0.0151 | 0.0167 | 1 | 0.1667 | 1 | 0.984001 | high |
| TCGA-BP-4174 | 0.9881 | 0.0486 | 0.9927 | 0.4276 | 0.0472 | 0.2855 | 0.313 | 0.2523 | 0.9797 | 0.687886 | low |
| TCGA-BP-4161 | 0.8875 | 0.044 | 0.9943 | 0.5253 | 0.3077 | 0 | 0.5374 | 0.2525 | 0.9832 | 1.179595 | high |
| TCGA-BP-5183 | 0.9563 | 0.0236 | 0.9864 | 0.4924 | 0.0799 | 0 | 0.2607 | 0.2472 | 1 | 1.017318 | high |
| TCGA-CW-5588 | 0.9947 | 0.011 | 0.9792 | 0.5016 | 0.6212 | 0.27132 | 0.064 | 0.1653 | 1 | 1.14855 | high |
| TCGA-BP-4970 | 0.981 | 0.0133 | 0.9918 | 0.5708 | 0.2333 | 0.3847 | 0.5635 | 0.2614 | 0.9948 | 0.307989 | low |
| TCGA-A3-3319 | 0.8762 | 0.0644 | 0.9614 | 0.4371 | 0.9145 | 0.1349 | 0.7916 | 0.0742 | 0.9865 | 2.711482 | high |
| TCGA-BP-4349 | 0.9075 | 0.1095 | 0.982 | 0.4692 | 0.2866 | 0 | 0.49948 | 0.4585 | 1 | 1.213936 | high |
| TCGA-A3-3335 | 0.909 | 0.0315 | 0.9849 | 0.3925 | 0.2999 | 0.3347 | 0.5667 | 0.0603 | 1 | 1.197396 | high |
| TCGA-CZ-5464 | 0.9751 | 0.0642 | 0.9914 | 0.4248 | 0.7327 | 0.0572 | 0.7841 | 0.1551 | 0.9897 | 1.306043 | high |
| TCGA-AK-3436 | 1 | 0.09 | 0.9727 | 0.386 | 0.6938 | 0.0783 | 0.7063 | 0.2121 | 1 | 1.450621 | high |
| TCGA-AK-3427 | 0.9712 | 0.0249 | 0.9883 | 0.6646 | 0.1244 | 0.14204 | 0.56034 | 0.0171 | 1 | 0.485511 | low |
| TCGA-BP-4163 | 0.9199 | 0.0194 | 0.9836 | 0.4239 | 0.1914 | 0 | 0.1272 | 0.1334 | 0.9694 | 2.32993 | high |
| TCGA-BP-4170 | 0.9622 | 0.0115 | 0.9889 | 0.469 | 0.1363 | 0.3615 | 0.5674 | 0.3528 | 0.9774 | 0.40208 | low |
| TCGA-B0-5088 | 0.9139 | 0.1707 | 0.9797 | 0.4335 | 0.2604 | 0 | 0.5152 | 0.3702 | 1 | 1.746343 | high |
| TCGA-BP-4329 | 0.9861 | 0.0343 | 0.9875 | 0.497 | 0.4436 | 0.2816 | 1 | 0.1558 | 1 | 0.419787 | low |
| TCGA-CW-5591 | 0.9627 | 0.0068 | 0.9803 | 0.5283 | 0.7083 | 0.30261 | 0.7575 | 0.1364 | 1 | 0.636853 | low |
| TCGA-CW-5580 | 0.9907 | 0.0152 | 0.9827 | 0.4338 | 0.5115 | 0.4119 | 0.3337 | 0.2658 | 0.969 | 0.744715 | low |
| TCGA-BP-4177 | 0.9817 | 0.0352 | 0.991 | 0.4406 | 0.1391 | 0.15585 | 0.3894 | 0.1048 | 1 | 0.949289 | high |
| TCGA-B0-4837 | 1 | 0.1643 | 0.9849 | 0.3857 | 0.756 | 0 | 0.2992 | 0.149 | 1 | 3.257583 | high |
| TCGA-CJ-4907 | 0.9778 | 0.0393 | 0.9805 | 0.4412 | 0.2253 | 0.2643 | 0.6089 | 0.1754 | 1 | 0.682068 | low |
| TCGA-CJ-4891 | 0.9258 | 0.18 | 0.9895 | 0.2547 | 0.8913 | 0.1735 | 0.1684 | 0.1432 | 1 | 6.680311 | high |
| TCGA-A3-3365 | 0.9227 | 0 | 0.974 | 0.5892 | 0.0973 | 0.1751 | 0.328 | 0.3438 | 1 | 0.56119 | low |
| TCGA-BP-4982 | 0.9374 | 0.0223 | 0.9915 | 0.4846 | 0.2334 | 0.4198 | 0.313 | 0.4103 | 0.9756 | 0.510901 | low |
| TCGA-B8-5549 | 0.9885 | 0.0158 | 0.9954 | 0.5403 | 0.5522 | 0.5698 | 0.6117 | 0.6176 | 1 | 0.172926 | low |
| TCGA-MM-A563 | 0.9414 | 0.032 | 0.9966 | 0.4098 | 0.4242 | 0.1245 | 0.5218 | 0.3492 | 1 | 0.955065 | high |
| TCGA-B0-4688 | 0.8557 | 0.0575 | 0.6083 | 0.4322 | 0.2651 | 0 | 0.2671 | 0.0224 | 1 | 35.12578 | high |
| TCGA-BP-5194 | 0.9794 | 0.011 | 0.9882 | 0.4606 | 0.217 | 0.3891 | 0.4372 | 0.3855 | 1 | 0.402413 | low |
| TCGA-CJ-4634 | 0.927 | 0 | 0.9771 | 0.4428 | 0.2909 | 0.119 | 0.5372 | 0.3309 | 0.949 | 0.983409 | high |
| TCGA-CZ-5462 | 1 | 0 | 1 | 0.5468 | 0.0815 | 0.2905 | 0.3561 | 0.0254 | 1 | 0.490451 | low |
| TCGA-CJ-4902 | 0.9093 | 0.0946 | 0.9808 | 0.4864 | 0.2626 | 0.1231 | 0.3576 | 0.3528 | 0.9348 | 1.401378 | high |
| TCGA-BP-4981 | 0.921 | 0.1734 | 0.9912 | 0.442 | 0.047 | 0.0396 | 0.3779 | 0.2832 | 1 | 1.611309 | high |
| TCGA-A3-A6NI | 0.9467 | 0.0503 | 0.979 | 0.5127 | 0.369 | 0.103 | 0.7662 | 0.3031 | 0.9831 | 0.695601 | low |
| TCGA-BP-5195 | 0.9852 | 0.0223 | 0.9829 | 0.4777 | 0.7597 | 0.1503 | 0.553 | 0.1477 | 1 | 1.094325 | high |
| TCGA-BP-4782 | 0.9624 | 0 | 0.9807 | 0.4873 | 0.2933 | 0.3891 | 0.5236 | 0.332 | 1 | 0.427481 | low |
| TCGA-A3-3311 | 0.9362 | 0.0203 | 0.9753 | 0.4804 | 0.2714 | 0 | 0.6239 | 0.3231 | 1 | 0.882038 | low |
| TCGA-CZ-4864 | 0.9246 | 0.0325 | 1 | 0.501 | 0.0206 | 0.0543 | 0.3768 | 0.3451 | 0.9708 | 0.782464 | low |
| TCGA-B2-4102 | 0.9217 | 0.0542 | 0.9823 | 0.4321 | 0.0731 | 0.1634 | 0.6343 | 0.334 | 0.9825 | 0.779545 | low |
| TCGA-CJ-4878 | 0.8465 | 0.0993 | 0.9856 | 0.6502 | 0.0572 | 0 | 1 | 0.2562 | 1 | 0.601177 | low |
| TCGA-AK-3428 | 1 | 0.027 | 0.9845 | 0.4315 | 0.0206 | 0.2408 | 0.7613 | 0.2774 | 1 | 0.392514 | low |
| TCGA-BP-4971 | 0.8233 | 0.2417 | 0.9921 | 0.4953 | 0.5116 | 0 | 0.2008 | 0.2058 | 0.8865 | 6.796548 | high |
| TCGA-CJ-5676 | 0.9864 | 0.0291 | 0.978 | 0.4069 | 0.3707 | 0.07687 | 0.5744 | 0.1446 | 1 | 1.156986 | high |
| TCGA-B8-A54J | 0.936 | 0.0543 | 0.9826 | 0.5347 | 0.1492 | 0.0574 | 0.6106 | 0.3752 | 1 | 0.620245 | low |
| TCGA-BP-4999 | 0.9402 | 0.0592 | 0.9825 | 0.4391 | 0.1534 | 0.2255 | 0.3725 | 0.3447 | 0.9788 | 0.888275 | high |
| TCGA-BP-4332 | 0.9016 | 0.0602 | 0.9839 | 0.428 | 0.3329 | 0.2107 | 0.3627 | 0.4323 | 1 | 1.077964 | high |
| TCGA-CZ-4863 | 0.9032 | 0.0378 | 0.9952 | 0.4718 | 0.1953 | 0.3101 | 0.5566 | 0.3374 | 0.9297 | 0.704505 | low |
| TCGA-A3-3374 | 1 | 0.0381 | 0.9858 | 0.4287 | 0.3303 | 0.124 | 0.8998 | 0.2329 | 1 | 0.570953 | low |
| TCGA-A3-3326 | 0.8822 | 0.0252 | 0.9926 | 0.4651 | 0.1002 | 0.2144 | 0.53485 | 0.3982 | 1 | 0.662776 | low |
| TCGA-T7-A92I | 0.9841 | 0.0111 | 0.9771 | 0.6937 | 0.1624 | 0.3849 | 0.6853 | 0.0431 | 1 | 0.260479 | low |
| TCGA-AK-3425 | 0.9349 | 0.0224 | 0.9883 | 0.4163 | 0.0511 | 0.1171 | 0.8108 | 0.2743 | 1 | 0.627067 | low |
| TCGA-CJ-4894 | 0.9407 | 0.0221 | 0.9918 | 0.4042 | 0.6395 | 0.1751 | 0.0964 | 0.2767 | 0.9764 | 1.911303 | high |
| TCGA-AK-3458 | 0.9703 | 0.1931 | 0.996 | 0.4583 | 0.0098 | 0 | 0.52016 | 0.1907 | 1 | 1.296346 | high |
| TCGA-B0-5106 | 0.9462 | 0.0458 | 0.9877 | 0.3698 | 0.1005 | 0 | 0.2706 | 0.3536 | 0.9348 | 1.628337 | high |
| TCGA-A3-3382 | 1 | 0.0799 | 0.9907 | 0.4563 | 0.8324 | 0.269 | 0.2908 | 0.0414 | 0.9826 | 1.712593 | high |
| TCGA-BP-4353 | 0.9049 | 0.0418 | 0.9778 | 0.4746 | 0.4557 | 0.4099 | 0.3004 | 0.2464 | 0.9557 | 1.136785 | high |
| TCGA-B0-5698 | 0.9605 | 0.071 | 0.981 | 0.5716 | 0.038 | 0.589 | 0.7301 | 0.3126 | 0.9822 | 0.216875 | low |
| TCGA-CZ-4866 | 0.9859 | 0.0238 | 0.9811 | 0.5596 | 0.2188 | 0.3117 | 0.8058 | 0.2173 | 0.9817 | 0.327534 | low |
| TCGA-BP-4799 | 0.9287 | 0.148 | 0.9741 | 0.284 | 0.421 | 0.08814 | 0.42207 | 0.0849 | 0.9168 | 4.931902 | high |
| TCGA-CW-5583 | 0.9697 | 0.0253 | 0.9867 | 0.442 | 0.1255 | 0.3829 | 0.6456 | 0.4602 | 0.9756 | 0.339021 | low |
| TCGA-B0-5085 | 0.8138 | 0.1371 | 0.9845 | 0.5323 | 0.0669 | 0.0481 | 0.1246 | 0.5157 | 0.573059 | 4.598427 | high |
| TCGA-CJ-5683 | 0.9655 | 0.0328 | 0.9771 | 0.4891 | 0.2518 | 0.13452 | 0.6671 | 0.3768 | 1 | 0.548399 | low |
| TCGA-CW-5590 | 0.9633 | 0.0117 | 0.9832 | 0.4447 | 0.3492 | 0.4502 | 0.5995 | 0.1125 | 1 | 0.59734 | low |
| TCGA-CZ-5457 | 1 | 0.0175 | 0.9866 | 0.5618 | 0.1713 | 0.4727 | 0.8582 | 0.2684 | 1 | 0.184288 | low |
| TCGA-B8-A7U6 | 0.9534 | 0.0358 | 0.9725 | 0.4323 | 0.2367 | 0.11577 | 0.4212 | 0.2654 | 1 | 1.090573 | high |
| TCGA-BP-5185 | 0.9859 | 0.1304 | 0.9873 | 0.4318 | 0.1343 | 0.4603 | 0.581656 | 0.129 | 1 | 0.641845 | low |
| TCGA-BP-4763 | 0.9748 | 0.0155 | 0.9797 | 0.422 | 0.387 | 0.9386 | 0.3291 | 0.06 | 1 | 0.431628 | low |
| TCGA-BP-5180 | 0.989 | 0.008 | 0.989 | 0.437 | 0.7752 | 0 | 0.7312 | 0.1335 | 0.9911 | 1.247241 | high |
| TCGA-B2-5635 | 0.9514 | 0.0374 | 0.9794 | 0.4595 | 0.3701 | 0.298 | 0.3612 | 0.4238 | 0.986 | 0.71151 | low |
| TCGA-BP-4789 | 0.9537 | 0.0221 | 0.9774 | 0.5254 | 0.2071 | 0.3508 | 0.5241 | 0.2577 | 0.9707 | 0.510826 | low |
| TCGA-A3-A8OV | 0.9408 | 0.0247 | 0.9864 | 0.5591 | 0.1784 | 0 | 0.57387 | 0.6565 | 0.9475 | 0.438995 | low |
| TCGA-CJ-4643 | 0.9015 | 0.0567 | 0.9877 | 0.4433 | 0.117 | 0.1751 | 0.4869 | 0.387 | 0.9791 | 0.882941 | low |
| TCGA-BP-4326 | 0.9328 | 0.083 | 0.9788 | 0.3399 | 0.5911 | 0.148 | 0.336 | 0.1003 | 0.949 | 3.383082 | high |
| TCGA-BP-4960 | 0.8877 | 0.1233 | 0.9804 | 0.4867 | 1 | 0.0181 | 0.426 | 0.3832 | 0.8527 | 3.746232 | high |
| TCGA-B0-5121 | 0.9446 | 0.1881 | 0.9836 | 0.4643 | 0.1647 | 0 | 0.1684 | 0.2807 | 1 | 2.135686 | high |
| TCGA-B2-4099 | 0.9754 | 0 | 0.9861 | 0.5113 | 0.0343 | 0.4886 | 0.3779 | 0.2423 | 0.9822 | 0.351144 | low |
| TCGA-BP-5168 | 1 | 0.1475 | 0.9929 | 0.5045 | 0.003 | 0.13145 | 0 | 0.5094 | 1 | 0.758986 | low |
| TCGA-DV-A4W0 | 0.9826 | 0.0517 | 0.9931 | 0.4485 | 0.4879 | 0.2774 | 0.4389 | 0.459 | 0.9683 | 0.632994 | low |
| TCGA-CJ-4905 | 0.9806 | 0.0075 | 0.9832 | 0.4123 | 0.0952 | 0.3891 | 0.528 | 0.265 | 0.9808 | 0.48954 | low |
| TCGA-CJ-5675 | 0.9868 | 0.0338 | 0.9668 | 0.4092 | 0.0373 | 0.1861 | 0.8932 | 0.1604 | 1 | 0.580205 | low |
| TCGA-AK-3443 | 0.9139 | 0.0524 | 0.9941 | 0.5528 | 0.8647 | 0.2255 | 0.9502 | 0.198 | 1 | 0.738128 | low |
| TCGA-MW-A4EC | 0.9599 | 0.0393 | 0.9869 | 0.5063 | 0.539 | 0.2424 | 0.4661 | 0.3137 | 0.974 | 0.769311 | low |
| TCGA-A3-3325 | 0.9237 | 0.031 | 0.9846 | 0.4939 | 0.1333 | 0.2494 | 0.801 | 0.3643 | 0.9872 | 0.434719 | low |
| TCGA-CZ-5469 | 0.9574 | 0.1304 | 0.9861 | 0.3765 | 0.7608 | 0 | 0.5561 | 0.1234 | 0.9507 | 3.289239 | high |
| TCGA-AK-3453 | 0.9625 | 0.0077 | 0.9585 | 0.6319 | 0.5691 | 0.2306 | 0.9545 | 0.0729 | 1 | 0.485285 | low |
| TCGA-CZ-5452 | 0.9887 | 0.0327 | 0.9818 | 0.4092 | 0.214 | 0.2415 | 0.6957 | 0.1145 | 0.9817 | 0.746369 | low |
| TCGA-CJ-4901 | 0.9301 | 0.0937 | 0.9597 | 0.4004 | 0.6303 | 0.2168 | 0.4243 | 0.1269 | 0.9302 | 2.829267 | high |
| TCGA-B0-4700 | 0.8797 | 0.1089 | 0.9564 | 0.4153 | 0.8283 | 0.4332 | 0.42906 | 0.1774 | 0.8921 | 3.047791 | high |
| TCGA-B2-3923 | 0.9574 | 0.0457 | 0.9509 | 0.4373 | 0.4757 | 0 | 0.9344 | 0.0291 | 1 | 1.502925 | high |
| TCGA-BP-5184 | 0.9625 | 0.038 | 0.9806 | 0.521 | 0.0937 | 0.3902 | 0.53196 | 0.4219 | 1 | 0.321952 | low |
| TCGA-B0-4810 | 0.9331 | 0.0587 | 0.9416 | 0.4296 | 0.3725 | 0.0705 | 0.546 | 0.1534 | 0.9694 | 2.067853 | high |
| TCGA-BP-5176 | 0.9822 | 0.0178 | 0.9837 | 0.5447 | 0.1289 | 0.12867 | 0.4084 | 0.2851 | 1 | 0.538473 | low |
| TCGA-CZ-5463 | 0.9784 | 0 | 1 | 0.5369 | 0.5583 | 0.2044 | 0.3071 | 0.5014 | 1 | 0.480132 | low |
| TCGA-B2-5633 | 0.9641 | 0.0215 | 0.9856 | 0.4058 | 0.5704 | 0.2157 | 0.5695 | 0.1482 | 0.9817 | 1.174473 | high |
| TCGA-B8-A54E | 0.9934 | 0.0098 | 1 | 0.6031 | 0.9687 | 0.2677 | 0.61537 | 0.1704 | 1 | 0.559291 | low |
| TCGA-B0-4844 | 0.9476 | 0.1968 | 0.9845 | 0.4226 | 0.5258 | 0 | 0.6029 | 0.2664 | 0.9254 | 2.518465 | high |
| TCGA-BP-5169 | 0.8557 | 0.0993 | 0.968 | 0.3511 | 0.9741 | 0.0198 | 0.53559 | 0.0409 | 0.8834 | 8.783335 | high |
| TCGA-B0-4714 | 0.8122 | 0.2929 | 0.9661 | 0.428 | 0.0237 | 0.0267 | 0.8096 | 0.2476 | 0.9254 | 3.684976 | high |
| TCGA-CZ-4853 | 0.9688 | 0.0516 | 0.9925 | 0.4538 | 0 | 0.1604 | 0.4356 | 0.2676 | 1 | 0.675749 | low |

Table S10

| ID | QSOX1\|9130\|RI | DHRS1\|26954\|RI | TAF1D\|18313\|RI | Risk score | risk |
| --- | --- | --- | --- | --- | --- |
| TCGA-CJ-4637 | 0.8024 | 0.4712 | 0.2567 | 1.915213 | high |
| TCGA-CZ-4861 | 0.8958 | 0.2876 | 0.09 | 0.564316 | low |
| TCGA-BP-4964 | 0.8512 | 0.2549 | 0.1675 | 0.808157 | low |
| TCGA-CJ-4887 | 0.8587 | 0.2672 | 0.2816 | 0.964294 | high |
| TCGA-BP-5198 | 0.807 | 0.166 | 0.0937 | 0.797188 | low |
| TCGA-BP-4967 | 0.8652 | 0.2602 | 0.2245 | 0.825985 | low |
| TCGA-A3-3351 | 0.8859 | 0.6605 | 0.249 | 1.57605 | high |
| TCGA-B0-4710 | 0.9051 | 0.2241 | 0.2443 | 0.62199 | low |
| TCGA-B0-5083 | 0.8396 | 0.2926 | 0.068 | 0.781725 | low |
| TCGA-BP-4803 | 0.8397 | 0.1879 | 0.0972 | 0.678933 | low |
| TCGA-CJ-4889 | 0.8665 | 0.2258 | 0.2248 | 0.769563 | low |
| TCGA-B0-5080 | 0.8922 | 0.3082 | 0.194 | 0.720438 | low |
| TCGA-CW-5587 | 0.8971 | 0.2012 | 0.0883 | 0.47628 | low |
| TCGA-A3-3376 | 0.8833 | 0.3259 | 0.2036 | 0.80077 | low |
| TCGA-CJ-4897 | 0.8387 | 0.1335 | 0.0559 | 0.574888 | low |
| TCGA-CW-6088 | 0.8999 | 0.2285 | 0.1819 | 0.580371 | low |
| TCGA-B8-5552 | 0.904 | 0.1785 | 0.1517 | 0.489178 | low |
| TCGA-CW-5589 | 0.8785 | 0.2353 | 0.0749 | 0.55698 | low |
| TCGA-B0-4833 | 0.8529 | 0.4894 | 0.3716 | 1.76232 | high |
| TCGA-CZ-4859 | 0.8949 | 0.0976 | 0.0521 | 0.374679 | low |
| TCGA-A3-3320 | 0.8956 | 0.3412 | 0.3448 | 0.977709 | high |
| TCGA-MM-A564 | 0.8349 | 0.571 | 0.357 | 2.235158 | high |
| TCGA-AS-3777 | 0.8494 | 0.1046 | 0.1813 | 0.635877 | low |
| TCGA-B0-5095 | 0.8735 | 0.4757 | 0.3397 | 1.42594 | high |
| TCGA-BP-4795 | 0.8272 | 0.2443 | 0.0822 | 0.793539 | low |
| TCGA-BP-4331 | 0.8756 | 0.4177 | 0.2528 | 1.085156 | high |
| TCGA-A3-A6NJ | 0.8864 | 0.1916 | 0.1535 | 0.561873 | low |
| TCGA-B0-4701 | 0.8288 | 0.4642 | 0.4401 | 2.212047 | high |
| TCGA-A3-3367 | 0.8389 | 0.2847 | 0.1965 | 0.971102 | high |
| TCGA-B0-5402 | 0.8249 | 0.2761 | 0.0761 | 0.844355 | low |
| TCGA-B0-5691 | 0.8831 | 0.2434 | 0.1222 | 0.596944 | low |
| TCGA-BP-4342 | 0.8763 | 0.6879 | 0.2228 | 1.681371 | high |
| TCGA-A3-3373 | 0.9004 | 0.1702 | 0.1886 | 0.526088 | low |
| TCGA-BP-4341 | 0.8621 | 0.4496 | 0.42 | 1.683639 | high |
| TCGA-CW-6090 | 0.9079 | 0.233 | 0.0549 | 0.444508 | low |
| TCGA-A3-3307 | 0.8332 | 0.2189 | 0.2298 | 0.946432 | high |
| TCGA-B2-3924 | 0.9583 | 0.7505 | 0.2855 | 1.253942 | high |
| TCGA-B0-5706 | 0.8678 | 0.2353 | 0.0696 | 0.590439 | low |
| TCGA-BP-4963 | 0.8818 | 0.5259 | 0.2469 | 1.259124 | high |
| TCGA-BP-4343 | 0.7714 | 0.1545 | 0.0456 | 0.898109 | low |
| TCGA-B0-5703 | 0.8767 | 0.2697 | 0.102 | 0.62945 | low |
| TCGA-CJ-6027 | 0.7901 | 0.193 | 0.2987 | 1.338928 | high |
| TCGA-B0-5108 | 0.8963 | 0.3934 | 0.286 | 0.965463 | high |
| TCGA-BP-4965 | 0.9003 | 0.1932 | 0.1762 | 0.537194 | low |
| TCGA-A3-A6NL | 0.9076 | 0.3262 | 0.1694 | 0.646753 | low |
| TCGA-CJ-5672 | 0.9077 | 0.147 | 0.2134 | 0.502985 | low |
| TCGA-B8-5158 | 0.8638 | 0.3082 | 0.1101 | 0.743485 | low |
| TCGA-CJ-4876 | 0.8734 | 0.3346 | 0.3703 | 1.162838 | high |
| TCGA-B0-5115 | 0.8156 | 0.3394 | 0.181 | 1.210448 | high |
| TCGA-CJ-4916 | 0.8907 | 0.3727 | 0.3333 | 1.046958 | high |
| TCGA-B0-4848 | 0.7984 | 0.1465 | 0.2034 | 0.985877 | high |
| TCGA-B4-5377 | 0.8928 | 0.0655 | 0.1032 | 0.39181 | low |
| TCGA-B0-4712 | 0.7134 | 0.1364 | 0.22 | 1.706405 | high |
| TCGA-A3-3324 | 0.8901 | 0.3779 | 0.1832 | 0.813899 | low |
| TCGA-CZ-5465 | 0.8325 | 0.2603 | 0.1355 | 0.868222 | low |
| TCGA-CZ-4860 | 0.6242 | 0.3271 | 0.0055 | 2.913775 | high |
| TCGA-B0-5102 | 0.8232 | 0.3157 | 0.2304 | 1.205289 | high |
| TCGA-B0-5690 | 0.8955 | 0.179 | 0.1312 | 0.498305 | low |
| TCGA-BP-4160 | 0.8939 | 0.1929 | 0.0861 | 0.476826 | low |
| TCGA-B0-4697 | 0.7891 | 0.8503 | 0.5204 | 6.650624 | high |
| TCGA-AK-3454 | 0.9123 | 0.3774 | 0.2305 | 0.768168 | low |
| TCGA-B0-5693 | 0.8929 | 0.3112 | 0.0708 | 0.580163 | low |
| TCGA-BP-4985 | 0.7763 | 0.2554 | 0.0984 | 1.149998 | high |
| TCGA-B0-4718 | 0.7596 | 0.5204 | 0.3735 | 3.377492 | high |
| TCGA-B0-5109 | 0.4829 | 0.3269 | 0.2851 | 11.66961 | high |
| TCGA-A3-3359 | 0.8681 | 0.4993 | 0.3276 | 1.508153 | high |
| TCGA-DV-5569 | 0.863 | 0.1689 | 0.0886 | 0.557287 | low |
| TCGA-B2-A4SR | 0.8856 | 0.3838 | 0.2728 | 0.991673 | high |
| TCGA-B0-4815 | 0.8077 | 0.6071 | 0.3838 | 2.974048 | high |
| TCGA-AK-3461 | 0.8445 | 0.3438 | 0.4425 | 1.613032 | high |
| TCGA-CJ-4892 | 0.7867 | 0.3289 | 0.2881 | 1.722501 | high |
| TCGA-B0-5695 | 0.9102 | 0.1215 | 0.0526 | 0.355655 | low |
| TCGA-BP-4975 | 0.8999 | 0.2086 | 0.0679 | 0.45752 | low |
| TCGA-AK-3456 | 0.9129 | 0.266 | 0.1761 | 0.566746 | low |
| TCGA-BP-4159 | 0.85 | 0.3209 | 0.0826 | 0.791055 | low |
| TCGA-CZ-5458 | 0.8961 | 0.1006 | 0.1337 | 0.431866 | low |
| TCGA-AK-3431 | 0.8904 | 0.2758 | 0.0379 | 0.521193 | low |
| TCGA-B8-A8YJ | 0.84 | 0.3947 | 0.247 | 1.289897 | high |
| TCGA-BP-5182 | 0.8987 | 0.179 | 0.0448 | 0.4192 | low |
| TCGA-B2-4101 | 0.8964 | 0.3766 | 0.347 | 1.042033 | high |
| TCGA-CJ-5681 | 0.8639 | 0.2373 | 0.1015 | 0.642622 | low |
| TCGA-BP-4994 | 0.8591 | 0.2865 | 0.1623 | 0.807153 | low |
| TCGA-CJ-4903 | 0.8958 | 0.3645 | 0.1494 | 0.721624 | low |
| TCGA-BP-4760 | 0.8407 | 0.1443 | 0.075 | 0.598878 | low |
| TCGA-BP-5190 | 0.8394 | 0.2219 | 0.1314 | 0.76905 | low |
| TCGA-B0-5710 | 0.8963 | 0.1853 | 0.1253 | 0.496348 | low |
| TCGA-CJ-4875 | 0.79 | 0.6037 | 0.4292 | 3.581836 | high |
| TCGA-CJ-4874 | 0.8863 | 0.0946 | 0.0669 | 0.403884 | low |
| TCGA-B2-5641 | 0.9001 | 0.1784 | 0.1467 | 0.496899 | low |
| TCGA-CW-5584 | 0.8728 | 0.1628 | 0.1197 | 0.547207 | low |
| TCGA-B8-4143 | 0.8069 | 0.6759 | 0.297 | 2.908916 | high |
| TCGA-B0-5120 | 0.8381 | 0.5091 | 0.091 | 1.222299 | high |
| TCGA-EU-5905 | 0.8925 | 0.0916 | 0.0751 | 0.391852 | low |
| TCGA-BP-4338 | 0.8372 | 0.2256 | 0.1077 | 0.752947 | low |
| TCGA-A3-3358 | 0.8907 | 0.4214 | 0.2228 | 0.941741 | high |
| TCGA-B0-4818 | 0.8516 | 0.3121 | 0.3537 | 1.24387 | high |
| TCGA-BP-4976 | 0.9058 | 0.1092 | 0.0445 | 0.352463 | low |
| TCGA-BP-4345 | 0.8531 | 0.3906 | 0.3271 | 1.357507 | high |
| TCGA-CW-6093 | 0.8732 | 0.199 | 0.1819 | 0.65101 | low |
| TCGA-BP-4330 | 0.896 | 0.3904 | 0.1798 | 0.797447 | low |
| TCGA-EU-5906 | 0.8856 | 0.1598 | 0.0897 | 0.475967 | low |
| TCGA-B0-4846 | 0.8059 | 0.1822 | 0.3013 | 1.193268 | high |
| TCGA-CJ-6030 | 0.9128 | 0.2569 | 0.1409 | 0.524102 | low |
| TCGA-BP-4983 | 0.7576 | 0.4006 | 0.2053 | 2.040251 | high |
| TCGA-B0-5697 | 0.8898 | 0.1673 | 0.1295 | 0.504126 | low |
| TCGA-BP-4346 | 0.8556 | 0.5644 | 0.308 | 1.776577 | high |
| TCGA-B0-4691 | 0.8156 | 0.3795 | 0.3481 | 1.750083 | high |
| TCGA-A3-3328 | 0.7851 | 0.2943 | 0.2195 | 1.446722 | high |
| TCGA-BP-4765 | 0.8646 | 0.3349 | 0.2169 | 0.938124 | high |
| TCGA-CZ-4858 | 0.7087 | 0.2794 | 0.2248 | 2.304215 | high |
| TCGA-B0-5117 | 0.7917 | 0.3775 | 0.2526 | 1.713476 | high |
| TCGA-CZ-4865 | 0.8763 | 0.12 | 0.1135 | 0.489446 | low |
| TCGA-CJ-6028 | 0.8357 | 0.1733 | 0.1357 | 0.725654 | low |
| TCGA-6D-AA2E | 0.8876 | 0.2266 | 0.3145 | 0.790158 | low |
| TCGA-CJ-4884 | 0.8319 | 0.3937 | 0.4137 | 1.819273 | high |
| TCGA-DV-5568 | 0.8616 | 0.2144 | 0.2641 | 0.833261 | low |
| TCGA-BP-4961 | 0.8762 | 0.265 | 0.151 | 0.682626 | low |
| TCGA-BP-4761 | 0.7712 | 0.4621 | 0.2069 | 2.101364 | high |
| TCGA-CJ-5677 | 0.7879 | 0.5075 | 0.2331 | 2.152063 | high |
| TCGA-CZ-5460 | 0.8134 | 0.2637 | 0.3201 | 1.365949 | high |
| TCGA-CJ-4900 | 0.8218 | 0.5214 | 0.408 | 2.426284 | high |
| TCGA-CZ-5451 | 0.9084 | 0.1085 | 0.064 | 0.358402 | low |
| TCGA-B8-A54F | 0.8538 | 0.5784 | 0.2572 | 1.685408 | high |
| TCGA-B0-4838 | 0.8653 | 0.3263 | 0.3571 | 1.177722 | high |
| TCGA-B0-4699 | 0.7616 | 0.4993 | 0.2895 | 2.766058 | high |
| TCGA-BP-4781 | 0.8403 | 0.2556 | 0.0605 | 0.717663 | low |
| TCGA-CJ-4644 | 0.862 | 0.0844 | 0.0722 | 0.466632 | low |
| TCGA-B0-5701 | 0.9234 | 0.3938 | 0.1094 | 0.595815 | low |
| TCGA-B0-5694 | 0.841 | 0.2047 | 0.0915 | 0.687474 | low |
| TCGA-B8-4621 | 0.8515 | 0.1968 | 0.1972 | 0.76419 | low |
| TCGA-BP-5006 | 0.8887 | 0.2581 | 0.1571 | 0.629537 | low |
| TCGA-BP-5181 | 0.8266 | 0.1106 | 0.028 | 0.566498 | low |
| TCGA-B0-5709 | 0.8551 | 0.1771 | 0.088 | 0.594093 | low |
| TCGA-BP-4167 | 0.7329 | 0.37 | 0.1869 | 2.183125 | high |
| TCGA-CJ-4638 | 0.8541 | 0.5232 | 0.4064 | 1.978756 | high |
| TCGA-EU-5907 | 0.9092 | 0.3078 | 0.0458 | 0.497594 | low |
| TCGA-CJ-4869 | 0.8399 | 0.3058 | 0.1409 | 0.909206 | low |
| TCGA-3Z-A93Z | 0.8301 | 0.2224 | 0.1648 | 0.866024 | low |
| TCGA-BP-5196 | 0.8118 | 0.3355 | 0.2678 | 1.435061 | high |
| TCGA-AK-3433 | 0.8655 | 0.5122 | 0.1269 | 1.101274 | high |
| TCGA-A3-3346 | 0.7393 | 0.3876 | 0.3328 | 2.80175 | high |
| TCGA-AK-3440 | 0.794 | 0.3313 | 0.1722 | 1.34617 | high |
| TCGA-B8-A54D | 0.8546 | 0.5124 | 0.4474 | 2.079088 | high |
| TCGA-BP-5200 | 0.9001 | 0.0585 | 0.0854 | 0.357922 | low |
| TCGA-B0-5400 | 0.8482 | 0.2624 | 0.3336 | 1.119777 | high |
| TCGA-BP-4969 | 0.8664 | 0.4871 | 0.3546 | 1.563568 | high |
| TCGA-BP-4756 | 0.8897 | 0.4677 | 0.2783 | 1.137929 | high |
| TCGA-BP-4776 | 0.8699 | 0.4237 | 0.3235 | 1.288748 | high |
| TCGA-CJ-4912 | 0.8901 | 0.6034 | 0.4253 | 1.887197 | high |
| TCGA-CZ-5985 | 0.8268 | 0.2229 | 0.1045 | 0.795679 | low |
| TCGA-B0-5098 | 0.755 | 0.6484 | 0.2951 | 3.828185 | high |
| TCGA-B8-5545 | 0.8683 | 0.2198 | 0.1085 | 0.612778 | low |
| TCGA-BP-4758 | 0.7926 | 0.2952 | 0.241 | 1.435444 | high |
| TCGA-B0-4696 | 0.6317 | 0.4561 | 0.3434 | 6.393828 | high |
| TCGA-A3-3322 | 0.8952 | 0.3807 | 0.1662 | 0.768678 | low |
| TCGA-B0-5107 | 0.8469 | 0.5369 | 0.4477 | 2.284368 | high |
| TCGA-DV-5565 | 0.8543 | 0.2561 | 0.2523 | 0.922542 | high |
| TCGA-CZ-5989 | 1 | 0.251456 | 0.1612 | 0.309773 | low |
| TCGA-BP-4173 | 0.8743 | 0.4226 | 0.2628 | 1.123664 | high |
| TCGA-CJ-4870 | 0.8644 | 0.1455 | 0.0396 | 0.485303 | low |
| TCGA-BP-4974 | 0.8724 | 0.398 | 0.2622 | 1.085958 | high |
| TCGA-B0-5075 | 0.8498 | 0.4676 | 0.2058 | 1.288418 | high |
| TCGA-BP-4162 | 0.8827 | 0.2749 | 0.1877 | 0.711799 | low |
| TCGA-B0-4839 | 0.8357 | 0.2164 | 0.0849 | 0.717912 | low |
| TCGA-B8-5553 | 0.8923 | 0.1029 | 0.0773 | 0.40212 | low |
| TCGA-CJ-4893 | 0.8779 | 0.1776 | 0.1855 | 0.611502 | low |
| TCGA-CJ-6033 | 0.7472 | 0.2874 | 0.2532 | 1.927024 | high |
| TCGA-B0-4827 | 0.9177 | 0.2431 | 0.0925 | 0.454816 | low |
| TCGA-BP-4977 | 0.8738 | 0.3352 | 0.2643 | 0.9629 | high |
| TCGA-BP-5187 | 0.8589 | 0.2016 | 0.0215 | 0.53939 | low |
| TCGA-CJ-4882 | 0.8009 | 0.6247 | 0.5372 | 4.204588 | high |
| TCGA-CJ-4899 | 0.8679 | 0.4028 | 0.3365 | 1.28528 | high |
| TCGA-B8-4620 | 0.7859 | 0.3815 | 0.1997 | 1.630866 | high |
| TCGA-CJ-4868 | 0.7887 | 0.2471 | 0.2313 | 1.324246 | high |
| TCGA-BP-5001 | 0.7859 | 0.5033 | 0.402 | 2.914582 | high |
| TCGA-DV-5575 | 0.8896 | 0.1657 | 0.0913 | 0.470446 | low |
| TCGA-CJ-4920 | 0.7931 | 0.246 | 0.272 | 1.3811 | high |
| TCGA-B0-4707 | 0.8535 | 0.4667 | 0.3867 | 1.72961 | high |
| TCGA-AK-3426 | 0.8011 | 0.7508 | 0.5951 | 5.86112 | high |
| TCGA-B0-4713 | 0.8651 | 0.4488 | 0.2714 | 1.268739 | high |
| TCGA-B0-5705 | 0.8391 | 0.0769 | 0.0719 | 0.531739 | low |
| TCGA-BP-5199 | 0.9253 | 0.4971 | 0.2818 | 0.96468 | high |
| TCGA-CZ-5988 | 0.8153 | 0.3015 | 0.1127 | 1.002773 | high |
| TCGA-BP-5004 | 0.8872 | 0.3803 | 0.1117 | 0.733837 | low |
| TCGA-A3-3370 | 0.8824 | 0.3394 | 0.2548 | 0.903661 | low |
| TCGA-B0-5696 | 0.8586 | 0.1393 | 0.0561 | 0.512475 | low |
| TCGA-BP-4797 | 0.8571 | 0.2191 | 0.1067 | 0.654849 | low |
| TCGA-CZ-5982 | 0.8726 | 0.1197 | 0.1803 | 0.563479 | low |
| TCGA-BP-4991 | 0.8792 | 0.3918 | 0.1982 | 0.918534 | high |
| TCGA-BP-4762 | 0.8704 | 0.1302 | 0.0223 | 0.44063 | low |
| TCGA-A3-3362 | 0.9026 | 0.3474 | 0.1467 | 0.666716 | low |
| TCGA-A3-A8OW | 0.8756 | 0.3751 | 0.2437 | 0.987636 | high |
| TCGA-CJ-4872 | 0.7608 | 0.376 | 0.1954 | 1.878069 | high |
| TCGA-B0-4847 | 0.7552 | 0.5391 | 0.3741 | 3.597761 | high |
| TCGA-B0-4822 | 0.8121 | 0.6211 | 0.5216 | 3.785423 | high |
| TCGA-BP-5175 | 0.7636 | 0.3624 | 0.3277 | 2.273436 | high |
| TCGA-BP-4340 | 0.922 | 0.4029 | 0.3026 | 0.859832 | low |
| TCGA-BP-4987 | 0.9402 | 0.2619 | 0.1518 | 0.453385 | low |
| TCGA-BP-5189 | 0.8714 | 0.2056 | 0.1528 | 0.633088 | low |
| TCGA-BP-5000 | 0.7968 | 0.4074 | 0.3695 | 2.154498 | high |
| TCGA-AK-3451 | 0.8888 | 0.5113 | 0.1226 | 0.941576 | high |
| TCGA-BP-5007 | 0.863 | 0.3148 | 0.2264 | 0.928844 | high |
| TCGA-A3-3363 | 0.8972 | 0.3683 | 0.3242 | 0.98081 | high |
| TCGA-AK-3455 | 0.8901 | 0.3316 | 0.3323 | 0.972951 | high |
| TCGA-B8-4146 | 0.9455 | 0.3374 | 0.1063 | 0.464628 | low |
| TCGA-BP-4770 | 0.7489 | 0.2918 | 0.1045 | 1.477902 | high |
| TCGA-A3-3378 | 0.9019 | 0.3732 | 0.3511 | 1.007411 | high |
| TCGA-DV-5573 | 0.8394 | 0.2359 | 0.2479 | 0.96933 | high |
| TCGA-BP-4962 | 0.8621 | 0.2361 | 0.1082 | 0.656276 | low |
| TCGA-B8-5546 | 0.8918 | 0.1217 | 0.2374 | 0.554006 | low |
| TCGA-BP-4347 | 0.8578 | 0.3684 | 0.2277 | 1.061452 | high |
| TCGA-A3-3343 | 0.8921 | 0.3713 | 0.1742 | 0.781478 | low |
| TCGA-CJ-4918 | 0.8657 | 0.1573 | 0.1304 | 0.577419 | low |
| TCGA-BP-5201 | 0.8354 | 0.3804 | 0.1952 | 1.180519 | high |
| TCGA-CZ-5459 | 0.8906 | 0.3288 | 0.1367 | 0.683024 | low |
| TCGA-BP-5170 | 0.822 | 0.209 | 0.0217 | 0.690784 | low |
| TCGA-B0-5094 | 0.8286 | 0.4253 | 0.2752 | 1.541255 | high |
| TCGA-BP-4973 | 0.8762 | 0.4689 | 0.2304 | 1.141354 | high |
| TCGA-CJ-5684 | 0.8678 | 0.1548 | 0.0527 | 0.494452 | low |
| TCGA-BP-4771 | 0.8426 | 0.5469 | 0.3879 | 2.151137 | high |
| TCGA-B0-5116 | 0.6439 | 0.3543 | 0.0735 | 3.048901 | high |
| TCGA-CW-5585 | 0.8972 | 0.1216 | 0.1059 | 0.424344 | low |
| TCGA-CJ-4881 | 0.7514 | 0.5321 | 0.4599 | 4.233714 | high |
| TCGA-A3-3323 | 0.8767 | 0.4651 | 0.1946 | 1.060615 | high |
| TCGA-A3-3380 | 0.8707 | 0.3595 | 0.1419 | 0.82708 | low |
| TCGA-B0-4834 | 0.8641 | 0.2505 | 0.2009 | 0.783733 | low |
| TCGA-AK-3450 | 0.9018 | 0.3484 | 0.2294 | 0.776922 | low |
| TCGA-CZ-5461 | 0.8468 | 0.1022 | 0.0577 | 0.517355 | low |
| TCGA-A3-3347 | 0.8202 | 0.3986 | 0.278 | 1.555421 | high |
| TCGA-B0-5110 | 0.8597 | 0.2324 | 0.21 | 0.792191 | low |
| TCGA-AK-3460 | 0.8641 | 0.2842 | 0.1273 | 0.732042 | low |
| TCGA-CJ-4908 | 0.8549 | 0.4216 | 0.2945 | 1.341117 | high |
| TCGA-BP-4774 | 0.8553 | 0.3841 | 0.281 | 1.21943 | high |
| TCGA-B0-4842 | 0.87 | 0.5691 | 0.3242 | 1.68339 | high |
| TCGA-B0-5812 | 0.8481 | 0.156 | 0.0388 | 0.547707 | low |
| TCGA-B0-4816 | 0.8604 | 0.3675 | 0.3479 | 1.288989 | high |
| TCGA-BP-4988 | 0.8811 | 0.6867 | 0.5839 | 3.079865 | high |
| TCGA-B0-4945 | 0.9211 | 0.3519 | 0.35 | 0.856343 | low |
| TCGA-DV-5576 | 0.8588 | 0.2849 | 0.2477 | 0.937605 | high |
| TCGA-MM-A84U | 0.8416 | 0.474 | 0.3248 | 1.694233 | high |
| TCGA-BP-4998 | 0.9056 | 0.3957 | 0.3822 | 1.083454 | high |
| TCGA-A3-3387 | 0.8889 | 0.2967 | 0.2047 | 0.734036 | low |
| TCGA-BP-4968 | 0.86 | 0.2952 | 0.3074 | 1.053708 | high |
| TCGA-BP-4995 | 0.8588 | 0.2707 | 0.1298 | 0.741771 | low |
| TCGA-CJ-4890 | 0.8082 | 0.3181 | 0.3925 | 1.772443 | high |
| TCGA-B0-5092 | 0.8505 | 0.5816 | 0.4272 | 2.337404 | high |
| TCGA-DV-A4VZ | 0.7852 | 0.6188 | 0.3819 | 3.491653 | high |
| TCGA-A3-3385 | 0.8943 | 0.4224 | 0.1416 | 0.798995 | low |
| TCGA-B0-4823 | 0.8766 | 0.2109 | 0.0209 | 0.490015 | low |
| TCGA-BP-4992 | 0.8006 | 0.9256 | 0.6621 | 9.118276 | high |
| TCGA-B8-4151 | 0.8774 | 0.2805 | 0.1234 | 0.663823 | low |
| TCGA-B4-5838 | 0.8657 | 0.1466 | 0.0961 | 0.532917 | low |
| TCGA-BP-4986 | 0.929 | 0.4211 | 0.161 | 0.66228 | low |
| TCGA-CZ-5984 | 0.806 | 0.1921 | 0.1458 | 0.922688 | high |
| TCGA-BP-4344 | 0.88 | 0.2856 | 0.2757 | 0.862587 | low |
| TCGA-CZ-4854 | 0.8499 | 0.4823 | 0.2453 | 1.41836 | high |
| TCGA-CJ-6032 | 0.8838 | 0.1215 | 0.203 | 0.548212 | low |
| TCGA-BP-4355 | 0.742 | 0.558 | 0.2267 | 3.120859 | high |
| TCGA-B0-4814 | 0.8787 | 0.3097 | 0.242 | 0.856464 | low |
| TCGA-BP-4759 | 0.8493 | 0.3205 | 0.0176 | 0.707861 | low |
| TCGA-B0-5097 | 0.7532 | 0.3205 | 0.2441 | 1.93986 | high |
| TCGA-CJ-4923 | 0.7996 | 0.4947 | 0.3833 | 2.5453 | high |
| TCGA-DV-A4VX | 0.8458 | 0.4603 | 0.4992 | 2.189345 | high |
| TCGA-CJ-5671 | 0.846 | 0.1966 | 0.1008 | 0.667119 | low |
| TCGA-BP-5191 | 0.7775 | 0.6522 | 0.1794 | 2.725425 | high |
| TCGA-BP-4158 | 0.8478 | 0.3306 | 0.1312 | 0.889727 | low |
| TCGA-BP-4768 | 0.9047 | 0.449 | 0.1299 | 0.769427 | low |
| TCGA-B2-5639 | 0.8449 | 0.0672 | 0.0934 | 0.523037 | low |
| TCGA-B0-5084 | 0.884 | 0.4423 | 0.2421 | 1.056298 | high |
| TCGA-CJ-5680 | 0.8733 | 0.2781 | 0.1019 | 0.653002 | low |
| TCGA-CJ-4639 | 0.8743 | 0.288 | 0.1092 | 0.669342 | low |
| TCGA-B8-5159 | 0.9581 | 0.0901 | 0.0842 | 0.262211 | low |
| TCGA-CJ-4641 | 0.8785 | 0.2873 | 0.3501 | 0.996215 | high |
| TCGA-B0-5692 | 0.8584 | 0.185 | 0.1673 | 0.679063 | low |
| TCGA-CJ-4895 | 0.8147 | 0.3693 | 0.304 | 1.598014 | high |
| TCGA-BP-4959 | 0.8839 | 0.1706 | 0.1076 | 0.506498 | low |
| TCGA-BP-4787 | 0.804 | 0.3108 | 0.3385 | 1.632586 | high |
| TCGA-AK-3465 | 0.8416 | 0.3097 | 0.3415 | 1.29117 | high |
| TCGA-BP-5009 | 0.8435 | 0.3773 | 0.3377 | 1.434381 | high |
| TCGA-EU-5904 | 0.8728 | 0.2561 | 0.1189 | 0.648358 | low |
| TCGA-A3-3306 | 0.8389 | 0.2593 | 0.1721 | 0.887822 | low |
| TCGA-BP-5192 | 0.8591 | 0.1021 | 0.0378 | 0.462003 | low |
| TCGA-BP-4784 | 0.7758 | 0.2476 | 0.0853 | 1.11125 | high |
| TCGA-B8-4153 | 0.8356 | 0.2477 | 0.2087 | 0.946751 | high |
| TCGA-B0-5081 | 0.8494 | 0.4683 | 0.3958 | 1.809127 | high |
| TCGA-G6-A8L6 | 0.8629 | 0.4253 | 0.2385 | 1.162706 | high |
| TCGA-AK-3445 | 0.8759 | 0.398 | 0.3262 | 1.189302 | high |
| TCGA-BP-4351 | 0.88 | 0.5994 | 0.3197 | 1.657206 | high |
| TCGA-B0-5100 | 0.7716 | 0.602 | 0.2624 | 2.987817 | high |
| TCGA-B0-5700 | 0.9098 | 0.0844 | 0.0332 | 0.321892 | low |
| TCGA-AK-3429 | 0.8991 | 0.229 | 0.0961 | 0.501744 | low |
| TCGA-BP-4798 | 0.8226 | 0.382 | 0.3862 | 1.799045 | high |
| TCGA-BP-4769 | 0.8368 | 0.3276 | 0.219 | 1.107769 | high |
| TCGA-CJ-5678 | 0.7684 | 0.2601 | 0.1577 | 1.35406 | high |
| TCGA-A3-3316 | 0.8827 | 0.3082 | 0.2389 | 0.828219 | low |
| TCGA-B8-5550 | 0.9141 | 0.1978 | 0.1164 | 0.446682 | low |
| TCGA-B0-5699 | 0.8895 | 0.2681 | 0.063 | 0.540252 | low |
| TCGA-CJ-4888 | 0.8295 | 0.4501 | 0.3903 | 1.965361 | high |
| TCGA-BP-4354 | 0.7755 | 0.4957 | 0.3811 | 2.95849 | high |
| TCGA-BP-4989 | 0.8815 | 0.5817 | 0.5119 | 2.231552 | high |
| TCGA-CJ-4873 | 0.831 | 0.5383 | 0.4257 | 2.436095 | high |
| TCGA-B0-4824 | 0.8142 | 0.3867 | 0.3236 | 1.713337 | high |
| TCGA-CZ-5455 | 0.8356 | 0.2079 | 0.0954 | 0.7205 | low |
| TCGA-BP-4164 | 0.7935 | 0.2202 | 0.1753 | 1.107654 | high |
| TCGA-CW-5581 | 0.8794 | 0.1577 | 0.0528 | 0.461992 | low |
| TCGA-A3-3313 | 0.7725 | 0.4612 | 0.2988 | 2.447442 | high |
| TCGA-B0-4845 | 0.8508 | 0.4112 | 0.3373 | 1.45642 | high |
| TCGA-B4-5378 | 0.839 | 0.2843 | 0.0872 | 0.79951 | low |
| TCGA-BP-5174 | 0.8574 | 0.173 | 0.1417 | 0.638948 | low |
| TCGA-BP-4801 | 0.8569 | 0.1789 | 0.0393 | 0.540732 | low |
| TCGA-CJ-5686 | 0.8982 | 0.2639 | 0.1125 | 0.553758 | low |
| TCGA-A3-3349 | 0.8745 | 0.4824 | 0.2527 | 1.230117 | high |
| TCGA-DV-5567 | 0.8796 | 0.2424 | 0.0937 | 0.579284 | low |
| TCGA-CZ-5987 | 0.844 | 0.2246 | 0.3587 | 1.121591 | high |
| TCGA-CJ-4885 | 0.8536 | 0.1346 | 0.0382 | 0.508087 | low |
| TCGA-DV-5574 | 0.891 | 0.2494 | 0.0787 | 0.531657 | low |
| TCGA-A3-3331 | 0.9088 | 0.2491 | 0.2833 | 0.681456 | low |
| TCGA-BP-5178 | 0.7229 | 0.134 | 0.1204 | 1.341715 | high |
| TCGA-CJ-4871 | 0.9101 | 0.1237 | 0.0823 | 0.376564 | low |
| TCGA-B0-4836 | 0.8654 | 0.4346 | 0.3913 | 1.524834 | high |
| TCGA-B8-A54I | 0.8776 | 0.401 | 0.3546 | 1.24393 | high |
| TCGA-B0-4821 | 0.6528 | 0.5523 | 0.5861 | 10.24593 | high |
| TCGA-BP-4777 | 0.8333 | 0.2992 | 0.2388 | 1.113388 | high |
| TCGA-CJ-4636 | 0.8463 | 0.4761 | 0.3295 | 1.664699 | high |
| TCGA-B0-5711 | 0.9004 | 0.1305 | 0.0781 | 0.402424 | low |
| TCGA-CZ-4857 | 0.7515 | 0.4647 | 0.1139 | 2.029363 | high |
| TCGA-B0-4852 | 0.8404 | 0.3425 | 0.2634 | 1.203594 | high |
| TCGA-A3-3329 | 0.9255 | 0.1486 | 0.2486 | 0.479645 | low |
| TCGA-CZ-5466 | 0.8914 | 0.3971 | 0.1017 | 0.72404 | low |
| TCGA-BP-5177 | 0.7791 | 0.2606 | 0.0479 | 1.043284 | high |
| TCGA-A3-3372 | 0.8725 | 0.4006 | 0.3212 | 1.210233 | high |
| TCGA-BP-4804 | 0.8601 | 0.4036 | 0.1292 | 0.937649 | high |
| TCGA-AK-3434 | 0.9502 | 0.1822 | 0.2108 | 0.408132 | low |
| TCGA-B0-4811 | 0.753 | 0.6616 | 0.3944 | 4.733407 | high |
| TCGA-BP-4165 | 0.8338 | 0.4081 | 0.1572 | 1.173196 | high |
| TCGA-B0-5099 | 0.8768 | 0.3241 | 0.2986 | 0.983589 | high |
| TCGA-BP-4766 | 0.8753 | 0.3333 | 0.124 | 0.741853 | low |
| TCGA-BP-4335 | 0.7971 | 0.5035 | 0.3686 | 2.560575 | high |
| TCGA-B0-4703 | 0.8839 | 0.3764 | 0.303 | 1.043076 | high |
| TCGA-CJ-4904 | 0.8447 | 0.1518 | 0.0325 | 0.549179 | low |
| TCGA-BP-4352 | 0.7971 | 0.888 | 0.3952 | 5.430598 | high |
| TCGA-CJ-4635 | 0.8882 | 0.4372 | 0.1514 | 0.868173 | low |
| TCGA-CJ-5679 | 0.8833 | 0.2813 | 0.1862 | 0.715572 | low |
| TCGA-CZ-4862 | 0.88 | 0.3435 | 0.2858 | 0.976441 | high |
| TCGA-B8-4622 | 0.893 | 0.2865 | 0.2753 | 0.795226 | low |
| TCGA-G6-A8L8 | 0.768 | 0.5661 | 0.309 | 3.107524 | high |
| TCGA-CJ-5682 | 0.8537 | 0.1045 | 0.1031 | 0.538874 | low |
| TCGA-BP-4176 | 0.8124 | 0.3577 | 0.2491 | 1.440622 | high |
| TCGA-B8-4148 | 0.8716 | 0.3105 | 0.2754 | 0.951651 | high |
| TCGA-CJ-4886 | 0.8662 | 0.1186 | 0.1994 | 0.605676 | low |
| TCGA-BP-5186 | 0.8875 | 0.1817 | 0.0709 | 0.473553 | low |
| TCGA-BP-4972 | 0.8983 | 0.224 | 0.0926 | 0.496609 | low |
| TCGA-BP-4993 | 0.8377 | 0.2008 | 0.1333 | 0.750388 | low |
| TCGA-CW-6097 | 0.8075 | 0.2604 | 0.1734 | 1.087628 | high |
| TCGA-B0-5077 | 0.884 | 0.3095 | 0.1718 | 0.731363 | low |
| TCGA-A3-3383 | 0.8378 | 0.2945 | 0.1804 | 0.967695 | high |
| TCGA-B0-4819 | 0.8568 | 0.4559 | 0.5803 | 2.33776 | high |
| TCGA-BP-4775 | 0.9187 | 0.2392 | 0.1595 | 0.505104 | low |
| TCGA-BP-4327 | 0.8346 | 0.5154 | 0.3509 | 2.000733 | high |
| TCGA-B0-5399 | 0.8155 | 0.2534 | 0.146 | 0.972536 | high |
| TCGA-A3-3357 | 0.9339 | 0.2933 | 0.2362 | 0.580127 | low |
| TCGA-B0-4694 | 0.8872 | 0.1997 | 0.1404 | 0.55442 | low |
| TCGA-A3-3317 | 0.9044 | 0.3564 | 0.3214 | 0.912386 | high |
| TCGA-B8-4154 | 0.9036 | 0.3682 | 0.0775 | 0.609069 | low |
| TCGA-B2-5636 | 0.8867 | 0.2098 | 0.0967 | 0.524481 | low |
| TCGA-B0-4817 | 0.8744 | 0.6319 | 0.4409 | 2.257432 | high |
| TCGA-BP-5010 | 0.7723 | 0.5006 | 0.3751 | 3.014125 | high |
| TCGA-BP-4325 | 0.8752 | 0.256 | 0.2675 | 0.830113 | low |
| TCGA-BP-5008 | 0.8901 | 0.2107 | 0.1493 | 0.564229 | low |
| TCGA-B0-5707 | 0.9079 | 0.2427 | 0.1453 | 0.530824 | low |
| TCGA-CZ-5454 | 0.8558 | 0.2605 | 0.0235 | 0.614959 | low |
| TCGA-BP-4807 | 0.894 | 0.1209 | 0.1275 | 0.449288 | low |
| TCGA-A3-3352 | 0.8955 | 0.2793 | 0.166 | 0.636856 | low |
| TCGA-B0-5712 | 0.8917 | 0.1137 | 0.1628 | 0.478842 | low |
| TCGA-B0-4843 | 0.7511 | 0.5976 | 0.3906 | 4.231833 | high |
| TCGA-B8-A54H | 0.9097 | 0.3674 | 0.294 | 0.857734 | low |
| TCGA-CZ-5456 | 0.8029 | 0.2226 | 0.0664 | 0.864856 | low |
| TCGA-B0-4828 | 0.857 | 0.4257 | 0.3576 | 1.490619 | high |
| TCGA-AK-3444 | 0.8892 | 0.1159 | 0.1917 | 0.514024 | low |
| TCGA-CJ-6031 | 0.8846 | 0.2309 | 0.0525 | 0.510965 | low |
| TCGA-B8-A54K | 0.8528 | 0.4196 | 0.1992 | 1.144292 | high |
| TCGA-DV-5566 | 0.8592 | 0.1132 | 0.0799 | 0.507578 | low |
| TCGA-B0-5113 | 0.872 | 0.541 | 0.1561 | 1.173175 | high |
| TCGA-BP-4169 | 0.8205 | 0.33 | 0.3106 | 1.450171 | high |
| TCGA-CZ-5986 | 0.8818 | 0.2016 | 0.0561 | 0.496044 | low |
| TCGA-CJ-4640 | 0.8109 | 0.1649 | 0.1688 | 0.8863 | low |
| TCGA-B4-5836 | 0.8851 | 0.1944 | 0.1844 | 0.601373 | low |
| TCGA-B0-5713 | 0.8974 | 0.1531 | 0.1487 | 0.484261 | low |
| TCGA-BP-4790 | 0.8541 | 0.2634 | 0.113 | 0.731956 | low |
| TCGA-BP-4174 | 0.8918 | 0.1717 | 0.2142 | 0.582804 | low |
| TCGA-BP-4161 | 0.8229 | 0.2697 | 0.2723 | 1.195192 | high |
| TCGA-BP-5183 | 0.8499 | 0.27 | 0.0866 | 0.726152 | low |
| TCGA-CW-5588 | 0.9189 | 0.2732 | 0.0581 | 0.448861 | low |
| TCGA-BP-4970 | 0.9 | 0.1287 | 0.2327 | 0.528384 | low |
| TCGA-A3-3319 | 0.6914 | 0.3002 | 0.3518 | 3.342304 | high |
| TCGA-BP-4349 | 0.8614 | 0.583 | 0.3236 | 1.821468 | high |
| TCGA-A3-3335 | 0.8375 | 0.3339 | 0.1409 | 0.971914 | high |
| TCGA-CZ-5464 | 0.8463 | 0.2812 | 0.217 | 0.954709 | high |
| TCGA-AK-3436 | 0.8433 | 0.2681 | 0.0496 | 0.706752 | low |
| TCGA-AK-3427 | 0.7718 | 0.3655 | 0.1321 | 1.53662 | high |
| TCGA-BP-4163 | 0.8541 | 0.3916 | 0.2873 | 1.259674 | high |
| TCGA-BP-4170 | 0.8303 | 0.294 | 0.062 | 0.822471 | low |
| TCGA-B0-5088 | 0.8522 | 0.4186 | 0.3256 | 1.43336 | high |
| TCGA-BP-4329 | 0.7546 | 0.2617 | 0.1236 | 1.395315 | high |
| TCGA-CW-5591 | 0.9141 | 0.0971 | 0.0687 | 0.34138 | low |
| TCGA-CW-5580 | 0.83 | 0.2134 | 0.1489 | 0.828786 | low |
| TCGA-BP-4177 | 0.8589 | 0.1995 | 0.0547 | 0.56977 | low |
| TCGA-B0-4837 | 0.8284 | 0.4144 | 0.2371 | 1.414225 | high |
| TCGA-CJ-4907 | 0.8824 | 0.2021 | 0.1501 | 0.583957 | low |
| TCGA-CJ-4891 | 0.693 | 0.4054 | 0.4349 | 4.646749 | high |
| TCGA-A3-3365 | 0.8156 | 0.2634 | 0.2702 | 1.232737 | high |
| TCGA-BP-4982 | 0.8794 | 0.3226 | 0.164 | 0.760709 | low |
| TCGA-B8-5549 | 0.8318 | 0.2145 | 0.0628 | 0.705215 | low |
| TCGA-MM-A563 | 0.8819 | 0.3609 | 0.3769 | 1.169939 | high |
| TCGA-B0-4688 | 0.7458 | 0.5269 | 0.2857 | 3.193997 | high |
| TCGA-BP-5194 | 0.8654 | 0.1822 | 0.0273 | 0.504716 | low |
| TCGA-CJ-4634 | 0.8635 | 0.2857 | 0.2418 | 0.902019 | low |
| TCGA-CZ-5462 | 0.879 | 0.2724 | 0.1119 | 0.634432 | low |
| TCGA-CJ-4902 | 0.8196 | 0.5086 | 0.3756 | 2.269559 | high |
| TCGA-BP-4981 | 0.9178 | 0.5623 | 0.4542 | 1.545797 | high |
| TCGA-A3-A6NI | 0.8536 | 0.376 | 0.2348 | 1.119278 | high |
| TCGA-BP-5195 | 0.8748 | 0.1552 | 0.0818 | 0.498346 | low |
| TCGA-BP-4782 | 0.8626 | 0.1993 | 0.2619 | 0.802277 | low |
| TCGA-A3-3311 | 0.8417 | 0.2544 | 0.3581 | 1.200655 | high |
| TCGA-CZ-4864 | 0.8851 | 0.3907 | 0.2491 | 0.966172 | high |
| TCGA-B2-4102 | 0.8528 | 0.4146 | 0.3177 | 1.397854 | high |
| TCGA-CJ-4878 | 0.9127 | 0.4544 | 0.5886 | 1.660999 | high |
| TCGA-AK-3428 | 0.8817 | 0.198 | 0.0192 | 0.461979 | low |
| TCGA-BP-4971 | 0.8503 | 0.602 | 0.624 | 3.439509 | high |
| TCGA-CJ-5676 | 0.9225 | 0.147 | 0.2903 | 0.524662 | low |
| TCGA-B8-A54J | 0.8656 | 0.2481 | 0.2921 | 0.908027 | low |
| TCGA-BP-4999 | 0.8642 | 0.207 | 0.1366 | 0.645559 | low |
| TCGA-BP-4332 | 0.852 | 0.3823 | 0.1767 | 1.032237 | high |
| TCGA-CZ-4863 | 0.9148 | 0.2787 | 0.2281 | 0.628304 | low |
| TCGA-A3-3374 | 0.8187 | 0.2977 | 0.3965 | 1.60897 | high |
| TCGA-A3-3326 | 0.8517 | 0.4223 | 0.1719 | 1.103484 | high |
| TCGA-T7-A92I | 0.8992 | 0.3595 | 0.2146 | 0.785245 | low |
| TCGA-AK-3425 | 0.9049 | 0.3738 | 0.203 | 0.761777 | low |
| TCGA-CJ-4894 | 0.8545 | 0.4115 | 0.2228 | 1.162844 | high |
| TCGA-AK-3458 | 0.8524 | 0.7469 | 0.3373 | 2.667745 | high |
| TCGA-B0-5106 | 0.8523 | 0.4656 | 0.2728 | 1.422306 | high |
| TCGA-A3-3382 | 0.8951 | 0.4279 | 0.23 | 0.938727 | high |
| TCGA-BP-4353 | 0.8225 | 0.4682 | 0.1572 | 1.406913 | high |
| TCGA-B0-5698 | 0.8831 | 0.1293 | 0.2264 | 0.582147 | low |
| TCGA-CZ-4866 | 0.9088 | 0.4143 | 0.301 | 0.951773 | high |
| TCGA-BP-4799 | 0.7265 | 0.4199 | 0.0924 | 2.108125 | high |
| TCGA-CW-5583 | 0.891 | 0.2871 | 0.07 | 0.561011 | low |
| TCGA-B0-5085 | 0.8408 | 0.6074 | 0.429 | 2.614016 | high |
| TCGA-CJ-5683 | 0.8371 | 0.1488 | 0.1519 | 0.707629 | low |
| TCGA-CW-5590 | 0.8522 | 0.1986 | 0.2153 | 0.788127 | low |
| TCGA-CZ-5457 | 0.8979 | 0.1689 | 0.0445 | 0.413379 | low |
| TCGA-B8-A7U6 | 0.8654 | 0.344 | 0.2266 | 0.96549 | high |
| TCGA-BP-5185 | 0.7079 | 0.2678 | 0.1082 | 1.845178 | high |
| TCGA-BP-4763 | 0.9132 | 0.2995 | 0.0853 | 0.512364 | low |
| TCGA-BP-5180 | 0.8133 | 0.2788 | 0.0717 | 0.906095 | low |
| TCGA-B2-5635 | 0.8886 | 0.2877 | 0.2114 | 0.732009 | low |
| TCGA-BP-4789 | 0.8712 | 0.2383 | 0.068 | 0.57942 | low |
| TCGA-A3-A8OV | 0.9064 | 0.3448 | 0.1666 | 0.670958 | low |
| TCGA-CJ-4643 | 0.8422 | 0.4276 | 0.2959 | 1.473041 | high |
| TCGA-BP-4326 | 0.7498 | 0.381 | 0.4577 | 3.229521 | high |
| TCGA-BP-4960 | 0.7909 | 0.4959 | 0.5205 | 3.434405 | high |
| TCGA-B0-5121 | 0.8381 | 0.2209 | 0.1271 | 0.768126 | low |
| TCGA-B2-4099 | 0.8326 | 0.3122 | 0.1913 | 1.053126 | high |
| TCGA-BP-5168 | 0.8634 | 0.1396 | 0.071 | 0.510684 | low |
| TCGA-DV-A4W0 | 0.8436 | 0.4296 | 0.251 | 1.353679 | high |
| TCGA-CJ-4905 | 0.903 | 0.1389 | 0.0445 | 0.378836 | low |
| TCGA-CJ-5675 | 0.8597 | 0.1277 | 0.0908 | 0.529706 | low |
| TCGA-AK-3443 | 0.9227 | 0.2735 | 0.2828 | 0.652055 | low |
| TCGA-MW-A4EC | 0.8773 | 0.2822 | 0.1526 | 0.701586 | low |
| TCGA-A3-3325 | 0.8241 | 0.4874 | 0.3095 | 1.887952 | high |
| TCGA-CZ-5469 | 0.6779 | 0.4006 | 0.3122 | 4.080303 | high |
| TCGA-AK-3453 | 0.82 | 0.2268 | 0.2005 | 0.991226 | high |
| TCGA-CZ-5452 | 0.8745 | 0.2263 | 0.3123 | 0.854641 | low |
| TCGA-CJ-4901 | 0.6397 | 0.3111 | 0.3096 | 4.389481 | high |
| TCGA-B0-4700 | 0.741 | 0.3199 | 0.2627 | 2.163142 | high |
| TCGA-B2-3923 | 0.8537 | 0.4686 | 0.1644 | 1.170514 | high |
| TCGA-BP-5184 | 0.904 | 0.1448 | 0.0471 | 0.382292 | low |
| TCGA-B0-4810 | 0.7276 | 0.5514 | 0.4532 | 5.039007 | high |
| TCGA-BP-5176 | 0.8561 | 0.0914 | 0.0861 | 0.502831 | low |
| TCGA-CZ-5463 | 0.858 | 0.2138 | 0.0691 | 0.603398 | low |
| TCGA-B2-5633 | 0.8941 | 0.1719 | 0.1871 | 0.547738 | low |
| TCGA-B8-A54E | 0.9179 | 0.1426 | 0.214 | 0.468257 | low |
| TCGA-B0-4844 | 0.8472 | 0.3138 | 0.354 | 1.283657 | high |
| TCGA-BP-5169 | 0.5853 | 0.5269 | 0.3152 | 9.289485 | high |
| TCGA-B0-4714 | 0.7288 | 0.601 | 0.3348 | 4.443301 | high |
| TCGA-CZ-4853 | 0.8706 | 0.449 | 0.0945 | 0.896834 | low |
