# Supplementary figures and images for: Systematic Identification of Survival-Associated Alternative Splicing Events in Kidney Renal Clear Cell Carcinoma
Source: Comput Math Methods Med. 2021 Apr 19;2021:5576933. doi: 10.1155/2021/5576933 (PMC8075682; doi:10.1155/2021/5576933)

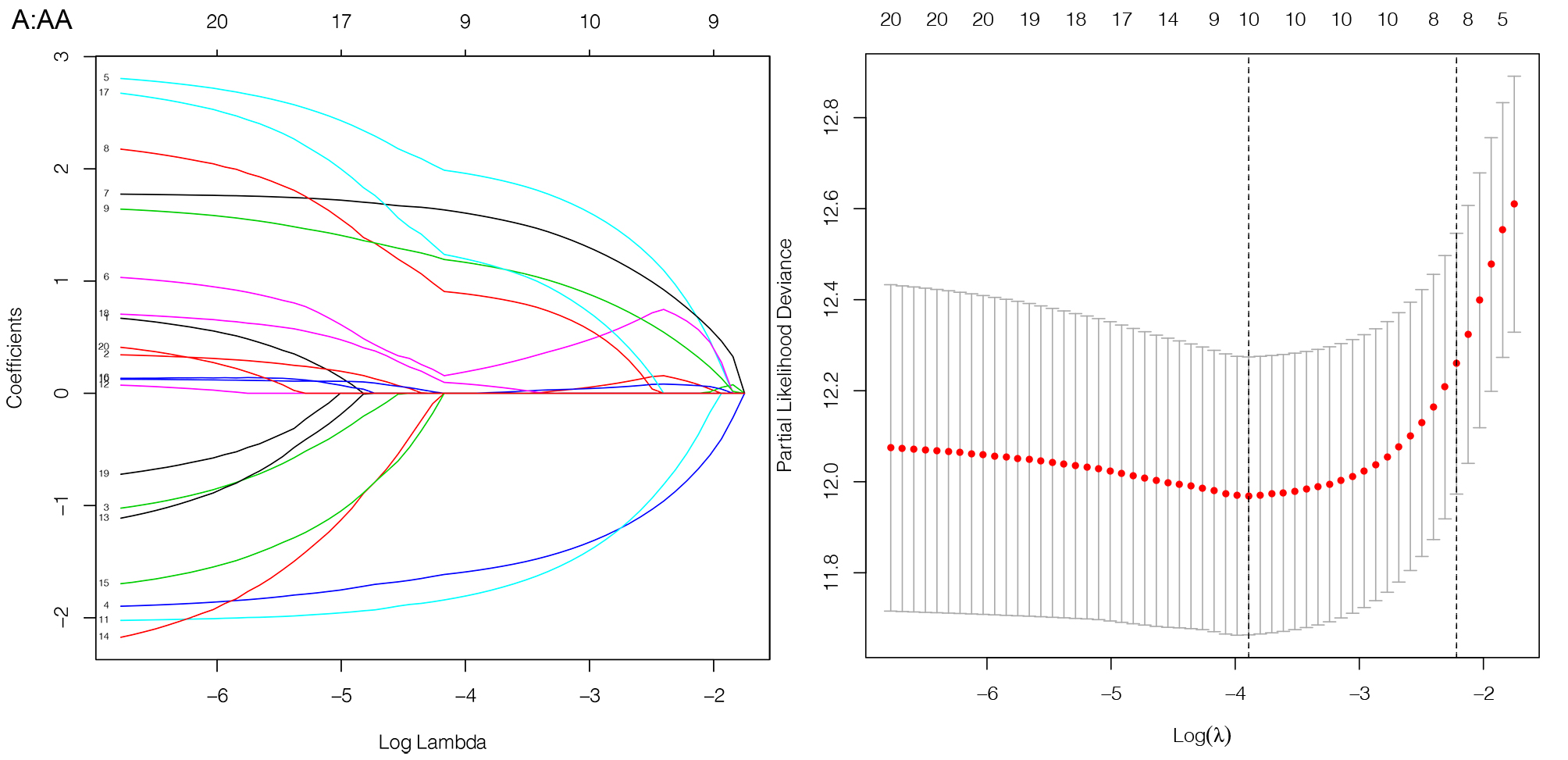

Supplement: Supplementary Materials — Figure S1: the tenfold cross-validation penalized LASSO logistic regression for AA. LASSO: least absolute shrinkage and selection operator; AA: alternate acceptor site. Figure S2: the tenfold cross-validation penalized LASSO logistic regression for AD. LASSO: least absolute shrinkage and selection operator; AD: alternative donor site. Figure S3: the tenfold cross-validation penalized LASSO logistic regression for AP. LASSO: least absolute shrinkage and selection operator; AP: alternate promoter. Figure S4: The tenfold cross-validation penalized LASSO logistic regression for AT. LASSO: least absolute shrinkage and selection operator; AT: alternative terminator. Figure S5: the tenfold cross-validation penalized LASSO logistic regression for ES. LASSO: least absolute shrinkage and selection operator; ES: exon skip. Figure S6: the tenfold cross-validation penalized LASSO logistic regression for ME. LASSO: least absolute shrinkage and selection operator; ME: mutually exclusive exon. Figure S7: the tenfold cross-validation penalized LASSO logistic regression for RI. LASSO: least absolute shrinkage and selection operator; RI: retained intron exons. Figure S8: details of prognosis signatures of AA. (A) The risk scores of KIRC patients' distribution basing on the median value. (B) The green dots mean survivors, and the red dots mean death cases. (C) The heat map shows the alteration of the percent spliced in value from low risk score to high risk score. AA: alternate acceptor site; KIRC: kidney renal clear cell carcinoma. Figure S9: details of prognosis signatures of AD. (A) The risk scores of KIRC patients' distribution basing on the median value. (B) The green dots mean survivors, and the red dots mean death cases. (C) The heat map shows the alteration of the percent spliced in value from low risk score to high risk score. AD: alternative donor site; KIRC: kidney renal clear cell carcinoma. Figure S10: details of prognosis signatures of AP. (A) The risk scores of KIRC pati [file 5576933.f1.zip › 5576933.f1/Figure S1 (1).jpg]

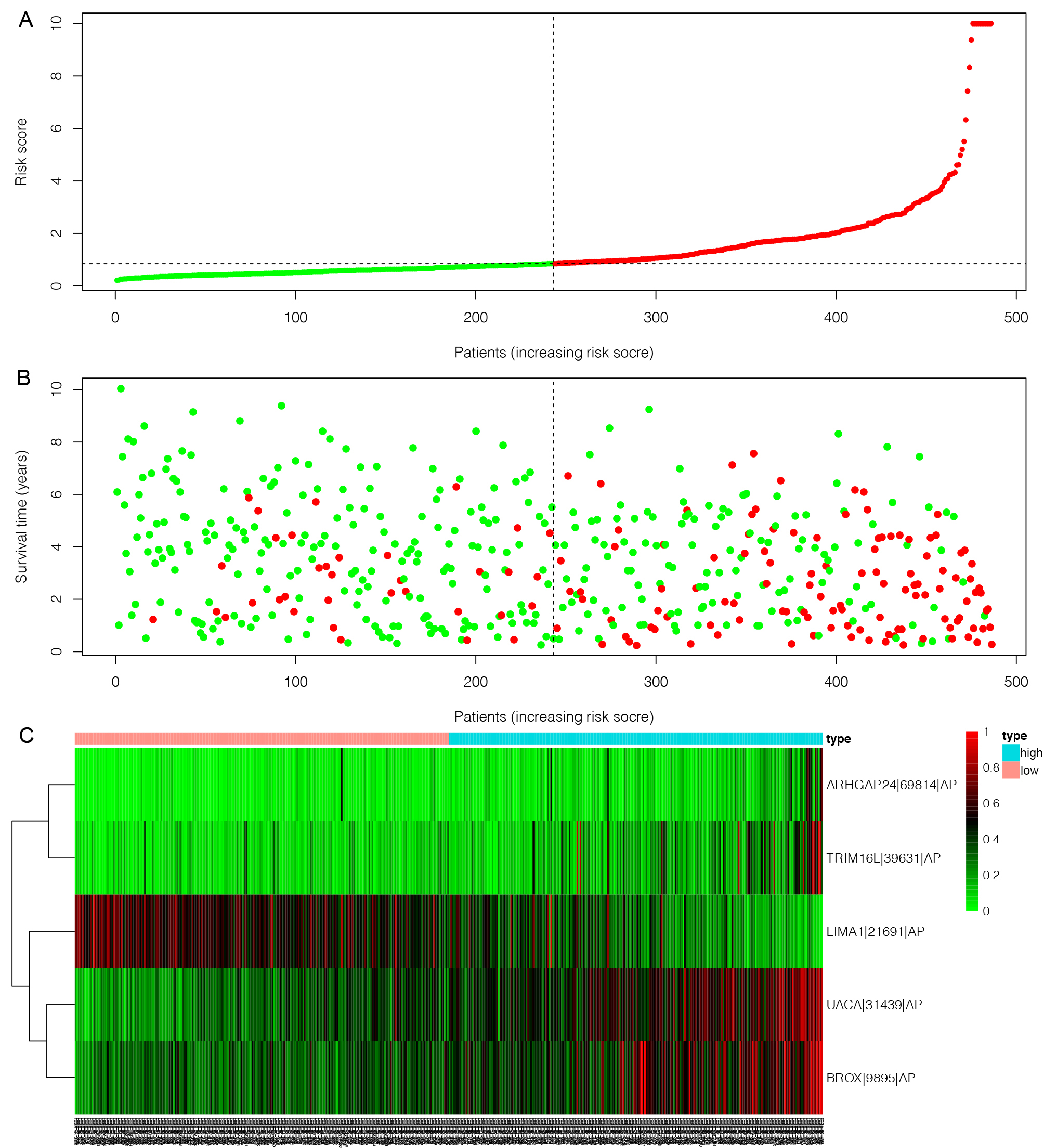

Supplement: Supplementary Materials — Figure S1: the tenfold cross-validation penalized LASSO logistic regression for AA. LASSO: least absolute shrinkage and selection operator; AA: alternate acceptor site. Figure S2: the tenfold cross-validation penalized LASSO logistic regression for AD. LASSO: least absolute shrinkage and selection operator; AD: alternative donor site. Figure S3: the tenfold cross-validation penalized LASSO logistic regression for AP. LASSO: least absolute shrinkage and selection operator; AP: alternate promoter. Figure S4: The tenfold cross-validation penalized LASSO logistic regression for AT. LASSO: least absolute shrinkage and selection operator; AT: alternative terminator. Figure S5: the tenfold cross-validation penalized LASSO logistic regression for ES. LASSO: least absolute shrinkage and selection operator; ES: exon skip. Figure S6: the tenfold cross-validation penalized LASSO logistic regression for ME. LASSO: least absolute shrinkage and selection operator; ME: mutually exclusive exon. Figure S7: the tenfold cross-validation penalized LASSO logistic regression for RI. LASSO: least absolute shrinkage and selection operator; RI: retained intron exons. Figure S8: details of prognosis signatures of AA. (A) The risk scores of KIRC patients' distribution basing on the median value. (B) The green dots mean survivors, and the red dots mean death cases. (C) The heat map shows the alteration of the percent spliced in value from low risk score to high risk score. AA: alternate acceptor site; KIRC: kidney renal clear cell carcinoma. Figure S9: details of prognosis signatures of AD. (A) The risk scores of KIRC patients' distribution basing on the median value. (B) The green dots mean survivors, and the red dots mean death cases. (C) The heat map shows the alteration of the percent spliced in value from low risk score to high risk score. AD: alternative donor site; KIRC: kidney renal clear cell carcinoma. Figure S10: details of prognosis signatures of AP. (A) The risk scores of KIRC pati [file 5576933.f1.zip › 5576933.f1/Figure S10 (1).jpg]

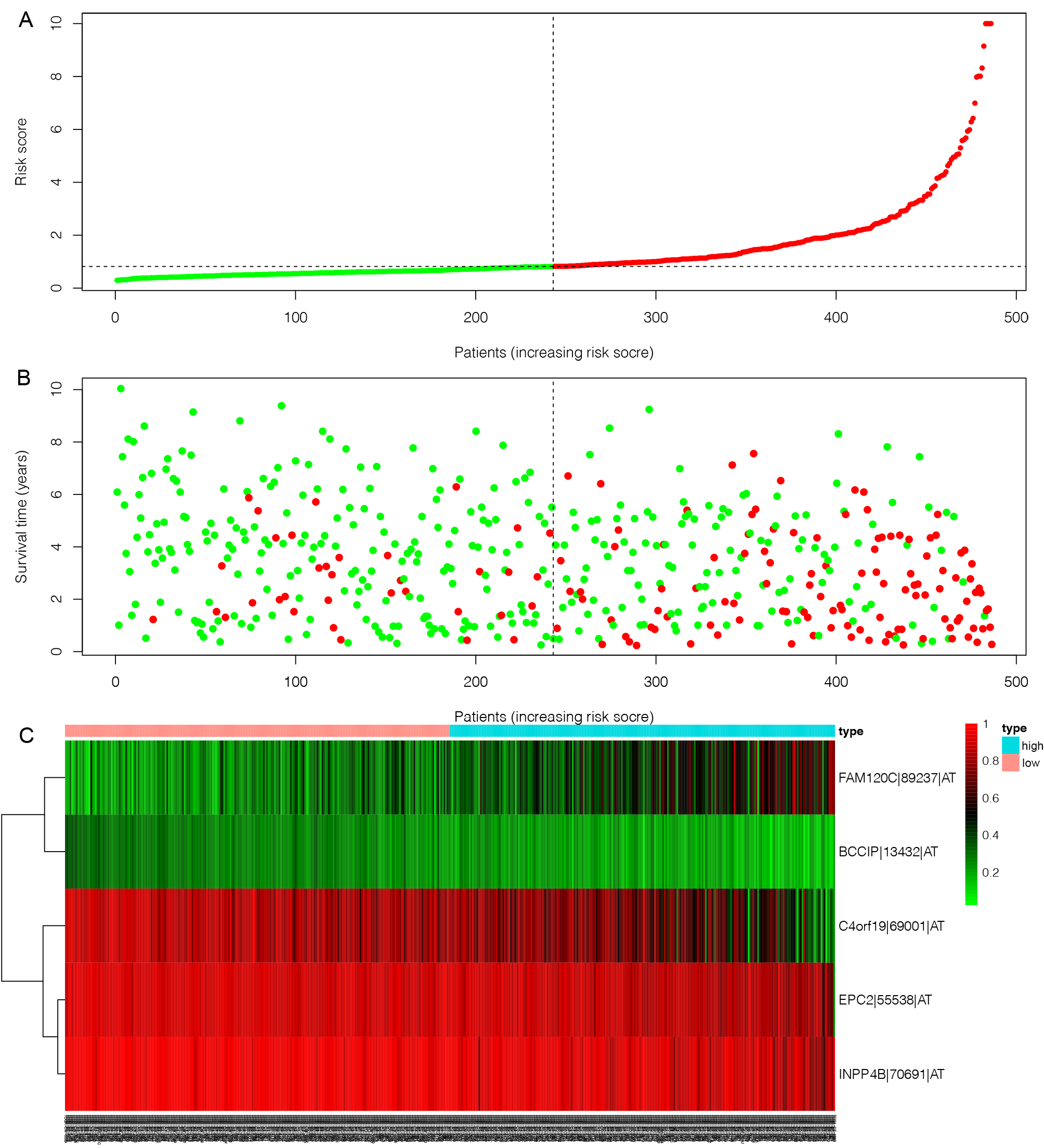

Supplement: Supplementary Materials — Figure S1: the tenfold cross-validation penalized LASSO logistic regression for AA. LASSO: least absolute shrinkage and selection operator; AA: alternate acceptor site. Figure S2: the tenfold cross-validation penalized LASSO logistic regression for AD. LASSO: least absolute shrinkage and selection operator; AD: alternative donor site. Figure S3: the tenfold cross-validation penalized LASSO logistic regression for AP. LASSO: least absolute shrinkage and selection operator; AP: alternate promoter. Figure S4: The tenfold cross-validation penalized LASSO logistic regression for AT. LASSO: least absolute shrinkage and selection operator; AT: alternative terminator. Figure S5: the tenfold cross-validation penalized LASSO logistic regression for ES. LASSO: least absolute shrinkage and selection operator; ES: exon skip. Figure S6: the tenfold cross-validation penalized LASSO logistic regression for ME. LASSO: least absolute shrinkage and selection operator; ME: mutually exclusive exon. Figure S7: the tenfold cross-validation penalized LASSO logistic regression for RI. LASSO: least absolute shrinkage and selection operator; RI: retained intron exons. Figure S8: details of prognosis signatures of AA. (A) The risk scores of KIRC patients' distribution basing on the median value. (B) The green dots mean survivors, and the red dots mean death cases. (C) The heat map shows the alteration of the percent spliced in value from low risk score to high risk score. AA: alternate acceptor site; KIRC: kidney renal clear cell carcinoma. Figure S9: details of prognosis signatures of AD. (A) The risk scores of KIRC patients' distribution basing on the median value. (B) The green dots mean survivors, and the red dots mean death cases. (C) The heat map shows the alteration of the percent spliced in value from low risk score to high risk score. AD: alternative donor site; KIRC: kidney renal clear cell carcinoma. Figure S10: details of prognosis signatures of AP. (A) The risk scores of KIRC pati [file 5576933.f1.zip › 5576933.f1/Figure S11 (1).jpg]

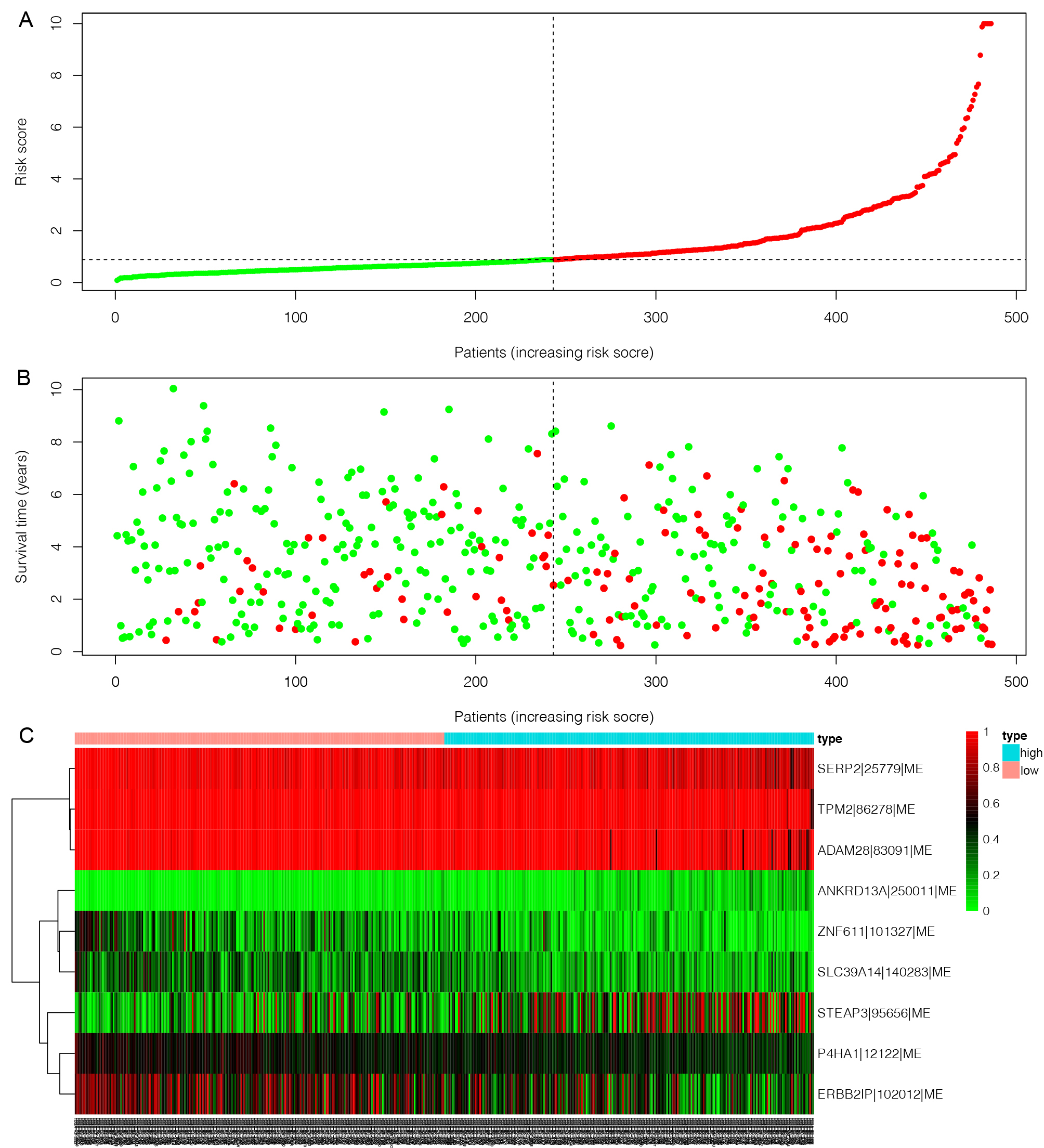

Supplement: Supplementary Materials — Figure S1: the tenfold cross-validation penalized LASSO logistic regression for AA. LASSO: least absolute shrinkage and selection operator; AA: alternate acceptor site. Figure S2: the tenfold cross-validation penalized LASSO logistic regression for AD. LASSO: least absolute shrinkage and selection operator; AD: alternative donor site. Figure S3: the tenfold cross-validation penalized LASSO logistic regression for AP. LASSO: least absolute shrinkage and selection operator; AP: alternate promoter. Figure S4: The tenfold cross-validation penalized LASSO logistic regression for AT. LASSO: least absolute shrinkage and selection operator; AT: alternative terminator. Figure S5: the tenfold cross-validation penalized LASSO logistic regression for ES. LASSO: least absolute shrinkage and selection operator; ES: exon skip. Figure S6: the tenfold cross-validation penalized LASSO logistic regression for ME. LASSO: least absolute shrinkage and selection operator; ME: mutually exclusive exon. Figure S7: the tenfold cross-validation penalized LASSO logistic regression for RI. LASSO: least absolute shrinkage and selection operator; RI: retained intron exons. Figure S8: details of prognosis signatures of AA. (A) The risk scores of KIRC patients' distribution basing on the median value. (B) The green dots mean survivors, and the red dots mean death cases. (C) The heat map shows the alteration of the percent spliced in value from low risk score to high risk score. AA: alternate acceptor site; KIRC: kidney renal clear cell carcinoma. Figure S9: details of prognosis signatures of AD. (A) The risk scores of KIRC patients' distribution basing on the median value. (B) The green dots mean survivors, and the red dots mean death cases. (C) The heat map shows the alteration of the percent spliced in value from low risk score to high risk score. AD: alternative donor site; KIRC: kidney renal clear cell carcinoma. Figure S10: details of prognosis signatures of AP. (A) The risk scores of KIRC pati [file 5576933.f1.zip › 5576933.f1/Figure S12 (1).jpg]

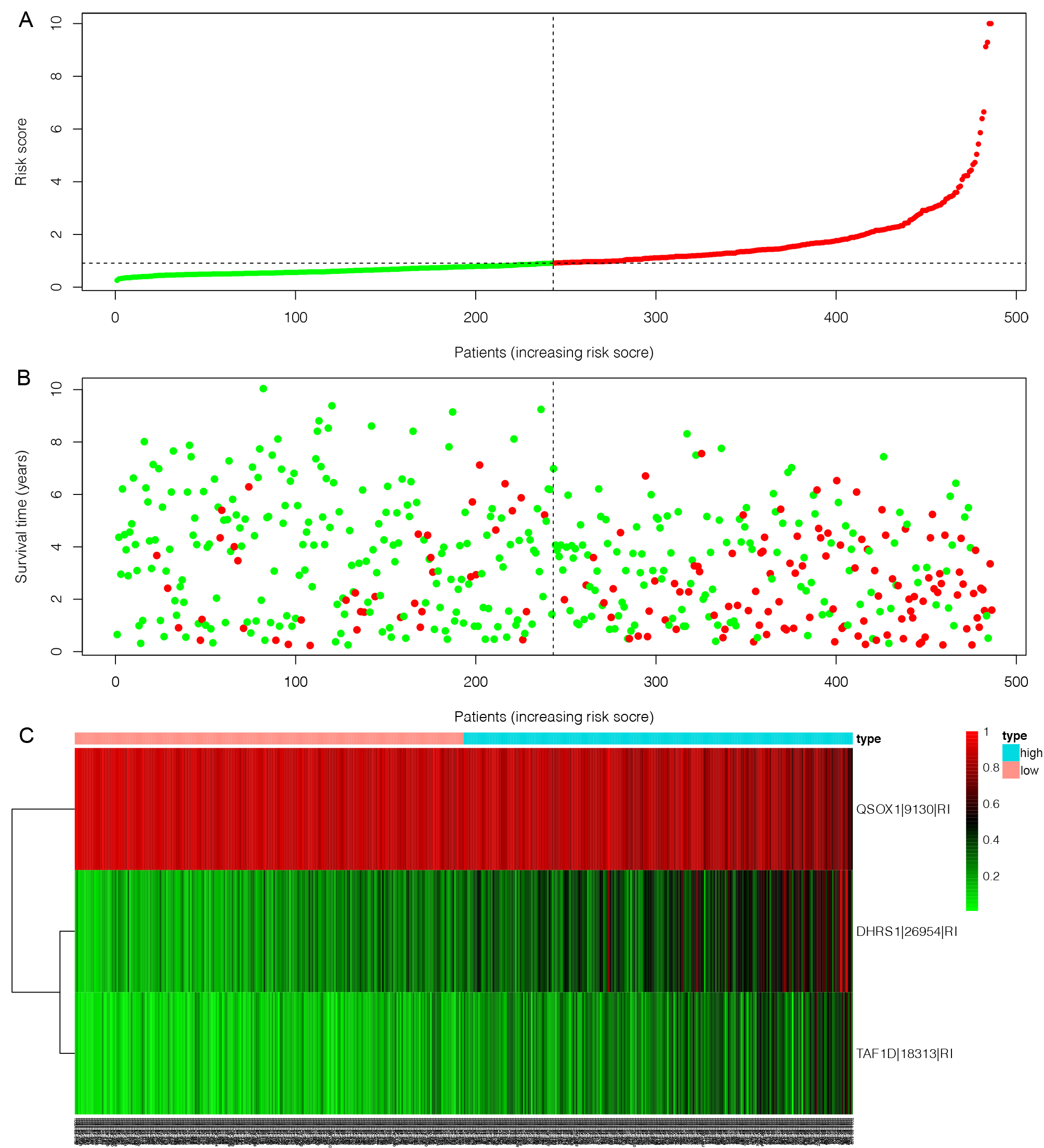

Supplement: Supplementary Materials — Figure S1: the tenfold cross-validation penalized LASSO logistic regression for AA. LASSO: least absolute shrinkage and selection operator; AA: alternate acceptor site. Figure S2: the tenfold cross-validation penalized LASSO logistic regression for AD. LASSO: least absolute shrinkage and selection operator; AD: alternative donor site. Figure S3: the tenfold cross-validation penalized LASSO logistic regression for AP. LASSO: least absolute shrinkage and selection operator; AP: alternate promoter. Figure S4: The tenfold cross-validation penalized LASSO logistic regression for AT. LASSO: least absolute shrinkage and selection operator; AT: alternative terminator. Figure S5: the tenfold cross-validation penalized LASSO logistic regression for ES. LASSO: least absolute shrinkage and selection operator; ES: exon skip. Figure S6: the tenfold cross-validation penalized LASSO logistic regression for ME. LASSO: least absolute shrinkage and selection operator; ME: mutually exclusive exon. Figure S7: the tenfold cross-validation penalized LASSO logistic regression for RI. LASSO: least absolute shrinkage and selection operator; RI: retained intron exons. Figure S8: details of prognosis signatures of AA. (A) The risk scores of KIRC patients' distribution basing on the median value. (B) The green dots mean survivors, and the red dots mean death cases. (C) The heat map shows the alteration of the percent spliced in value from low risk score to high risk score. AA: alternate acceptor site; KIRC: kidney renal clear cell carcinoma. Figure S9: details of prognosis signatures of AD. (A) The risk scores of KIRC patients' distribution basing on the median value. (B) The green dots mean survivors, and the red dots mean death cases. (C) The heat map shows the alteration of the percent spliced in value from low risk score to high risk score. AD: alternative donor site; KIRC: kidney renal clear cell carcinoma. Figure S10: details of prognosis signatures of AP. (A) The risk scores of KIRC pati [file 5576933.f1.zip › 5576933.f1/Figure S13 (1).jpg]

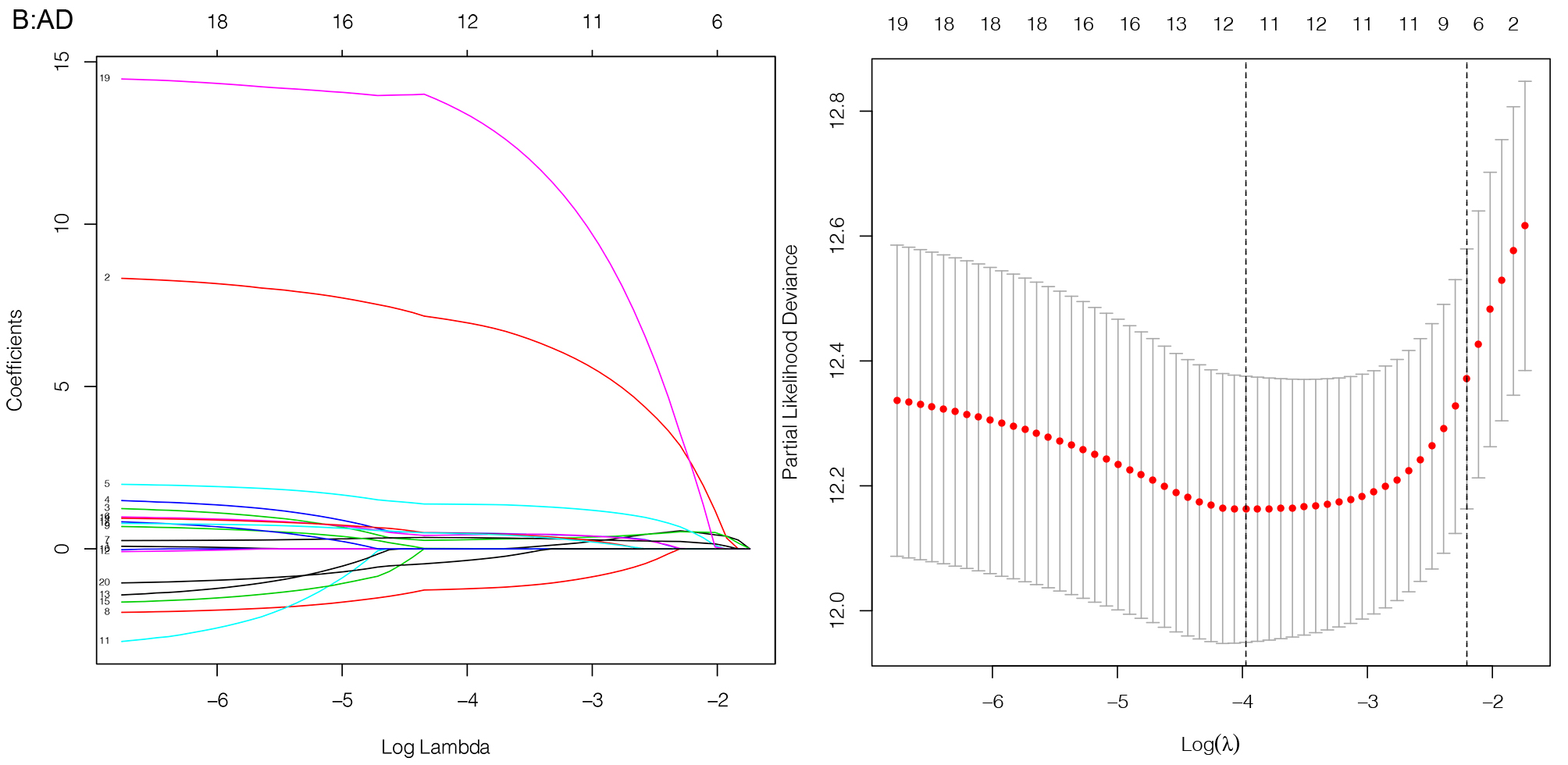

Supplement: Supplementary Materials — Figure S1: the tenfold cross-validation penalized LASSO logistic regression for AA. LASSO: least absolute shrinkage and selection operator; AA: alternate acceptor site. Figure S2: the tenfold cross-validation penalized LASSO logistic regression for AD. LASSO: least absolute shrinkage and selection operator; AD: alternative donor site. Figure S3: the tenfold cross-validation penalized LASSO logistic regression for AP. LASSO: least absolute shrinkage and selection operator; AP: alternate promoter. Figure S4: The tenfold cross-validation penalized LASSO logistic regression for AT. LASSO: least absolute shrinkage and selection operator; AT: alternative terminator. Figure S5: the tenfold cross-validation penalized LASSO logistic regression for ES. LASSO: least absolute shrinkage and selection operator; ES: exon skip. Figure S6: the tenfold cross-validation penalized LASSO logistic regression for ME. LASSO: least absolute shrinkage and selection operator; ME: mutually exclusive exon. Figure S7: the tenfold cross-validation penalized LASSO logistic regression for RI. LASSO: least absolute shrinkage and selection operator; RI: retained intron exons. Figure S8: details of prognosis signatures of AA. (A) The risk scores of KIRC patients' distribution basing on the median value. (B) The green dots mean survivors, and the red dots mean death cases. (C) The heat map shows the alteration of the percent spliced in value from low risk score to high risk score. AA: alternate acceptor site; KIRC: kidney renal clear cell carcinoma. Figure S9: details of prognosis signatures of AD. (A) The risk scores of KIRC patients' distribution basing on the median value. (B) The green dots mean survivors, and the red dots mean death cases. (C) The heat map shows the alteration of the percent spliced in value from low risk score to high risk score. AD: alternative donor site; KIRC: kidney renal clear cell carcinoma. Figure S10: details of prognosis signatures of AP. (A) The risk scores of KIRC pati [file 5576933.f1.zip › 5576933.f1/Figure S2 (1).jpg]

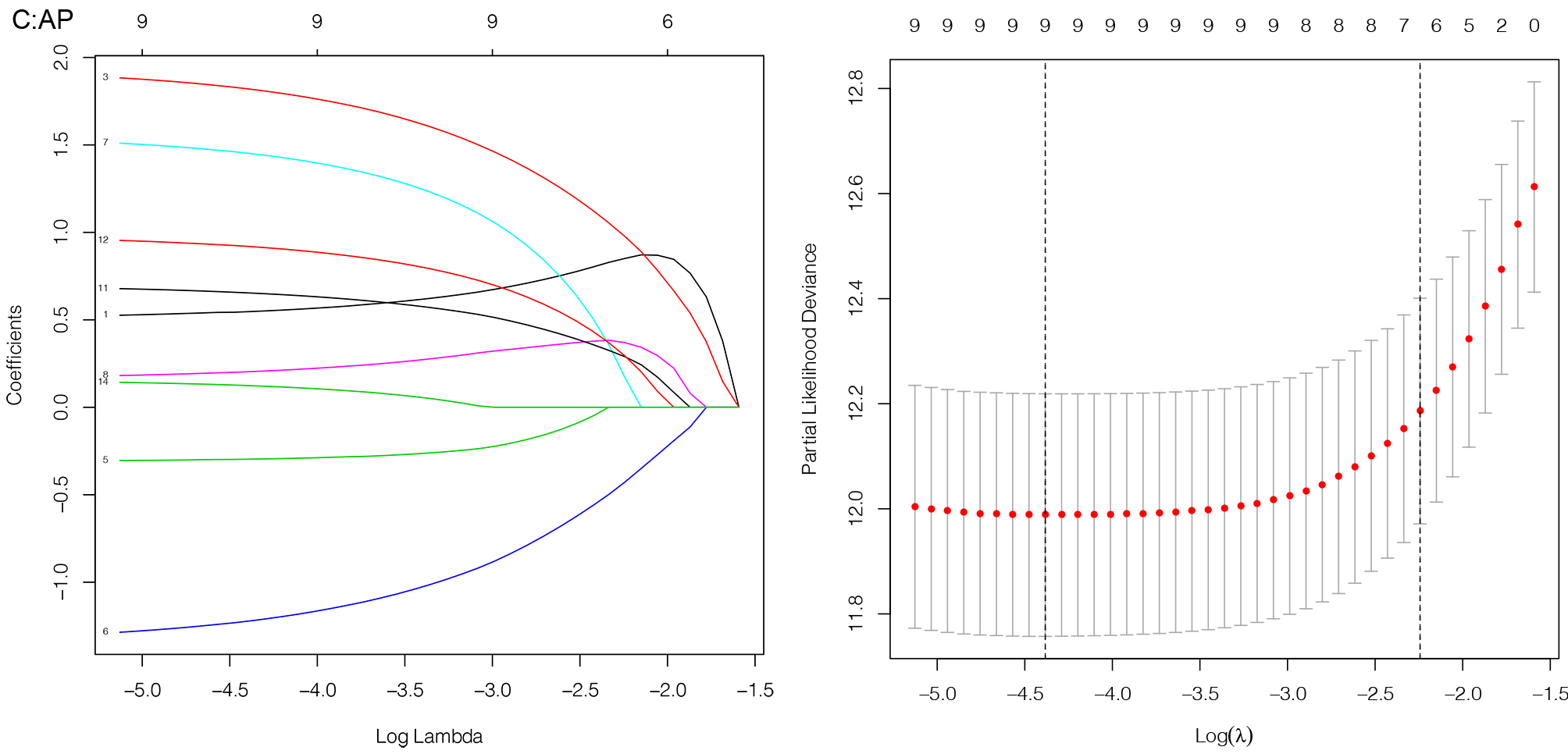

Supplement: Supplementary Materials — Figure S1: the tenfold cross-validation penalized LASSO logistic regression for AA. LASSO: least absolute shrinkage and selection operator; AA: alternate acceptor site. Figure S2: the tenfold cross-validation penalized LASSO logistic regression for AD. LASSO: least absolute shrinkage and selection operator; AD: alternative donor site. Figure S3: the tenfold cross-validation penalized LASSO logistic regression for AP. LASSO: least absolute shrinkage and selection operator; AP: alternate promoter. Figure S4: The tenfold cross-validation penalized LASSO logistic regression for AT. LASSO: least absolute shrinkage and selection operator; AT: alternative terminator. Figure S5: the tenfold cross-validation penalized LASSO logistic regression for ES. LASSO: least absolute shrinkage and selection operator; ES: exon skip. Figure S6: the tenfold cross-validation penalized LASSO logistic regression for ME. LASSO: least absolute shrinkage and selection operator; ME: mutually exclusive exon. Figure S7: the tenfold cross-validation penalized LASSO logistic regression for RI. LASSO: least absolute shrinkage and selection operator; RI: retained intron exons. Figure S8: details of prognosis signatures of AA. (A) The risk scores of KIRC patients' distribution basing on the median value. (B) The green dots mean survivors, and the red dots mean death cases. (C) The heat map shows the alteration of the percent spliced in value from low risk score to high risk score. AA: alternate acceptor site; KIRC: kidney renal clear cell carcinoma. Figure S9: details of prognosis signatures of AD. (A) The risk scores of KIRC patients' distribution basing on the median value. (B) The green dots mean survivors, and the red dots mean death cases. (C) The heat map shows the alteration of the percent spliced in value from low risk score to high risk score. AD: alternative donor site; KIRC: kidney renal clear cell carcinoma. Figure S10: details of prognosis signatures of AP. (A) The risk scores of KIRC pati [file 5576933.f1.zip › 5576933.f1/Figure S3 (1).jpg]

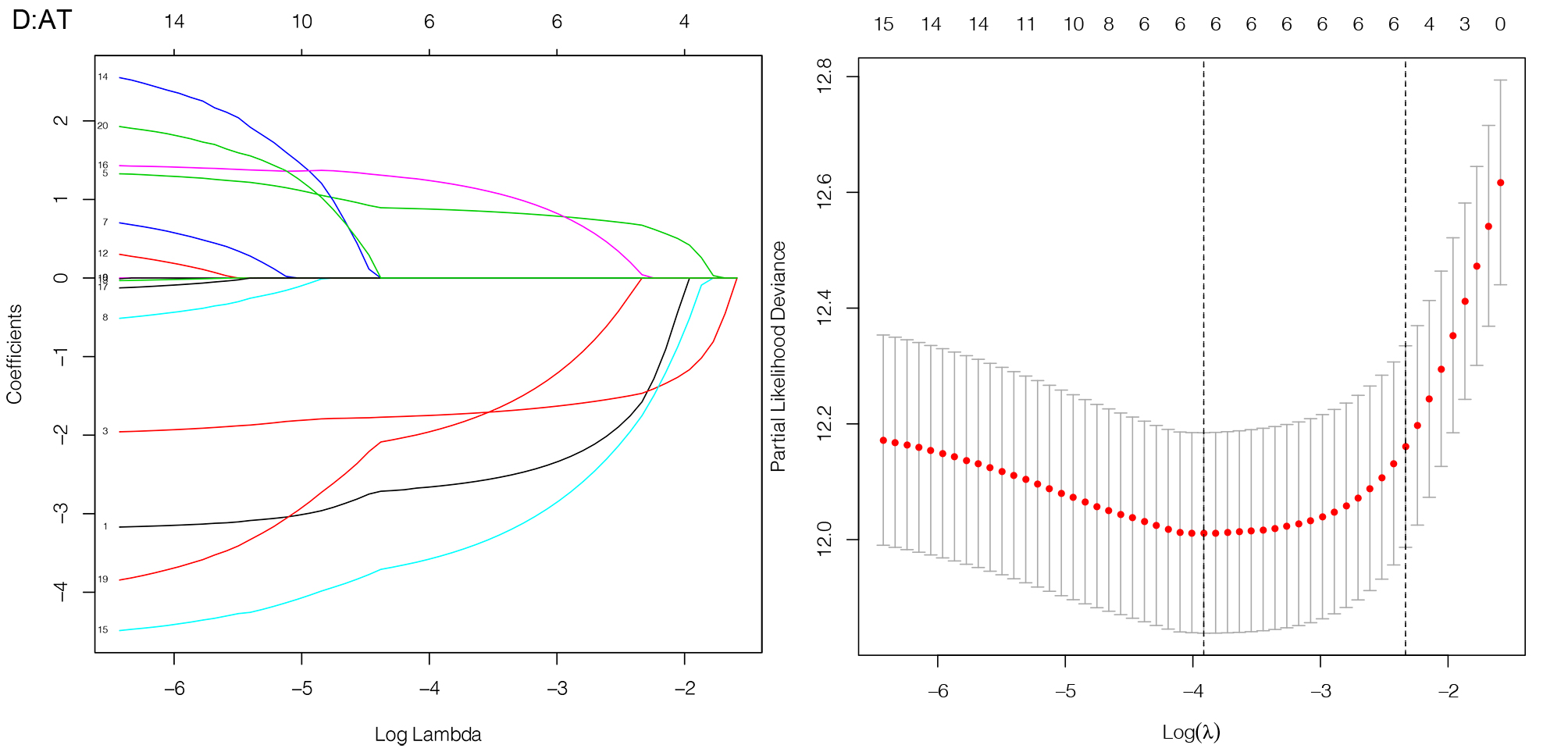

Supplement: Supplementary Materials — Figure S1: the tenfold cross-validation penalized LASSO logistic regression for AA. LASSO: least absolute shrinkage and selection operator; AA: alternate acceptor site. Figure S2: the tenfold cross-validation penalized LASSO logistic regression for AD. LASSO: least absolute shrinkage and selection operator; AD: alternative donor site. Figure S3: the tenfold cross-validation penalized LASSO logistic regression for AP. LASSO: least absolute shrinkage and selection operator; AP: alternate promoter. Figure S4: The tenfold cross-validation penalized LASSO logistic regression for AT. LASSO: least absolute shrinkage and selection operator; AT: alternative terminator. Figure S5: the tenfold cross-validation penalized LASSO logistic regression for ES. LASSO: least absolute shrinkage and selection operator; ES: exon skip. Figure S6: the tenfold cross-validation penalized LASSO logistic regression for ME. LASSO: least absolute shrinkage and selection operator; ME: mutually exclusive exon. Figure S7: the tenfold cross-validation penalized LASSO logistic regression for RI. LASSO: least absolute shrinkage and selection operator; RI: retained intron exons. Figure S8: details of prognosis signatures of AA. (A) The risk scores of KIRC patients' distribution basing on the median value. (B) The green dots mean survivors, and the red dots mean death cases. (C) The heat map shows the alteration of the percent spliced in value from low risk score to high risk score. AA: alternate acceptor site; KIRC: kidney renal clear cell carcinoma. Figure S9: details of prognosis signatures of AD. (A) The risk scores of KIRC patients' distribution basing on the median value. (B) The green dots mean survivors, and the red dots mean death cases. (C) The heat map shows the alteration of the percent spliced in value from low risk score to high risk score. AD: alternative donor site; KIRC: kidney renal clear cell carcinoma. Figure S10: details of prognosis signatures of AP. (A) The risk scores of KIRC pati [file 5576933.f1.zip › 5576933.f1/Figure S4 (1).jpg]

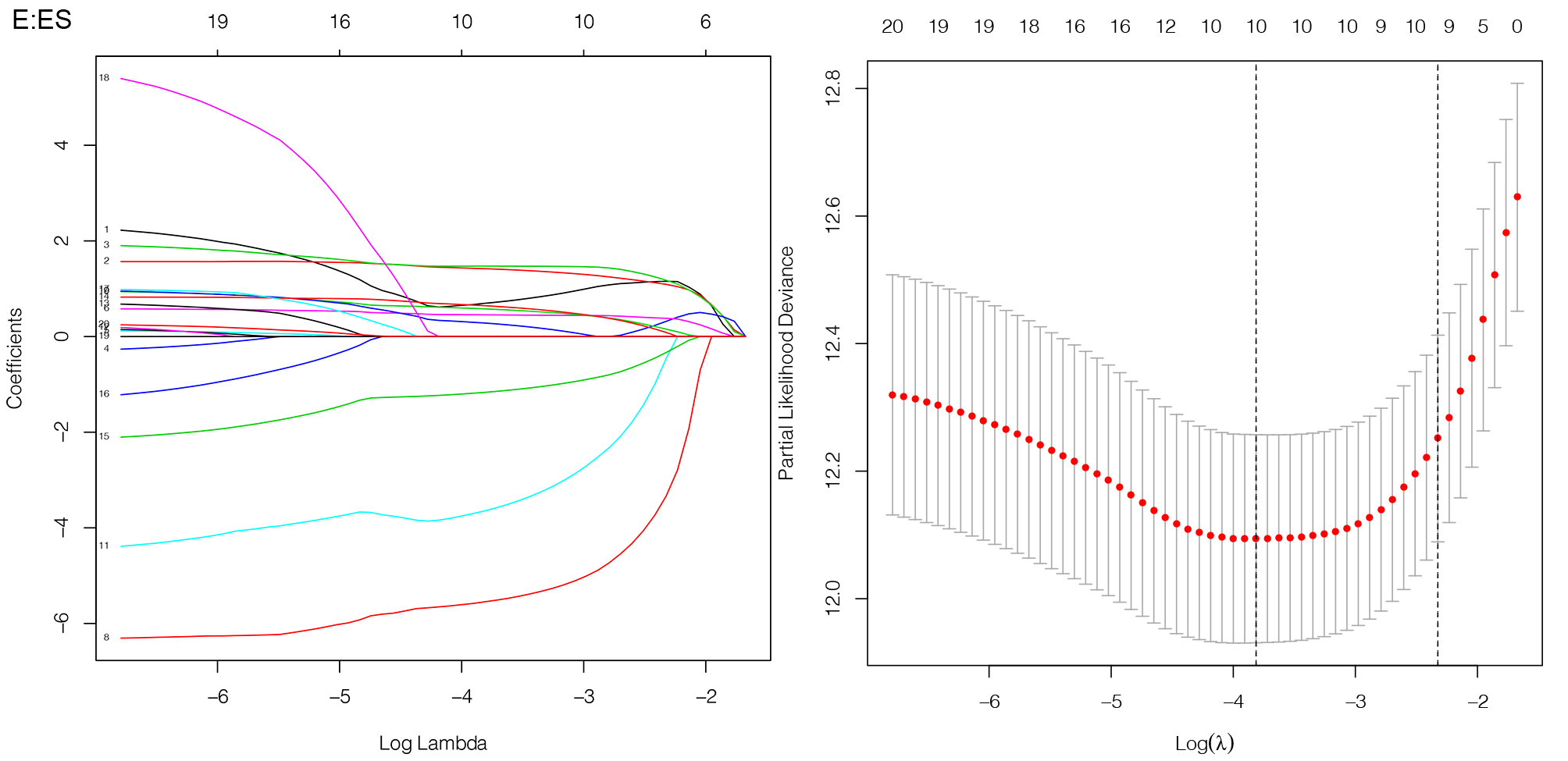

Supplement: Supplementary Materials — Figure S1: the tenfold cross-validation penalized LASSO logistic regression for AA. LASSO: least absolute shrinkage and selection operator; AA: alternate acceptor site. Figure S2: the tenfold cross-validation penalized LASSO logistic regression for AD. LASSO: least absolute shrinkage and selection operator; AD: alternative donor site. Figure S3: the tenfold cross-validation penalized LASSO logistic regression for AP. LASSO: least absolute shrinkage and selection operator; AP: alternate promoter. Figure S4: The tenfold cross-validation penalized LASSO logistic regression for AT. LASSO: least absolute shrinkage and selection operator; AT: alternative terminator. Figure S5: the tenfold cross-validation penalized LASSO logistic regression for ES. LASSO: least absolute shrinkage and selection operator; ES: exon skip. Figure S6: the tenfold cross-validation penalized LASSO logistic regression for ME. LASSO: least absolute shrinkage and selection operator; ME: mutually exclusive exon. Figure S7: the tenfold cross-validation penalized LASSO logistic regression for RI. LASSO: least absolute shrinkage and selection operator; RI: retained intron exons. Figure S8: details of prognosis signatures of AA. (A) The risk scores of KIRC patients' distribution basing on the median value. (B) The green dots mean survivors, and the red dots mean death cases. (C) The heat map shows the alteration of the percent spliced in value from low risk score to high risk score. AA: alternate acceptor site; KIRC: kidney renal clear cell carcinoma. Figure S9: details of prognosis signatures of AD. (A) The risk scores of KIRC patients' distribution basing on the median value. (B) The green dots mean survivors, and the red dots mean death cases. (C) The heat map shows the alteration of the percent spliced in value from low risk score to high risk score. AD: alternative donor site; KIRC: kidney renal clear cell carcinoma. Figure S10: details of prognosis signatures of AP. (A) The risk scores of KIRC pati [file 5576933.f1.zip › 5576933.f1/Figure S5 (1).jpg]

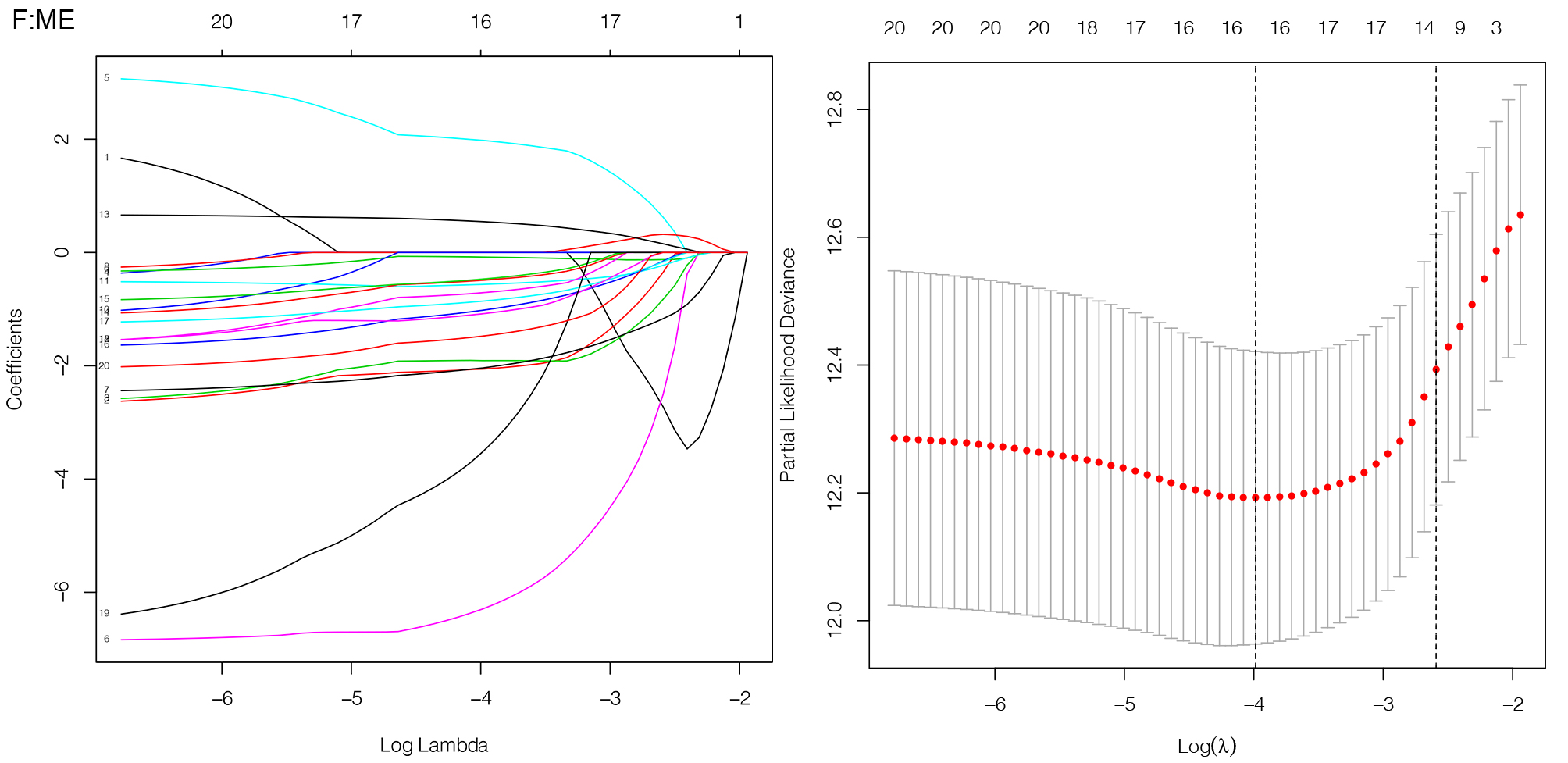

Supplement: Supplementary Materials — Figure S1: the tenfold cross-validation penalized LASSO logistic regression for AA. LASSO: least absolute shrinkage and selection operator; AA: alternate acceptor site. Figure S2: the tenfold cross-validation penalized LASSO logistic regression for AD. LASSO: least absolute shrinkage and selection operator; AD: alternative donor site. Figure S3: the tenfold cross-validation penalized LASSO logistic regression for AP. LASSO: least absolute shrinkage and selection operator; AP: alternate promoter. Figure S4: The tenfold cross-validation penalized LASSO logistic regression for AT. LASSO: least absolute shrinkage and selection operator; AT: alternative terminator. Figure S5: the tenfold cross-validation penalized LASSO logistic regression for ES. LASSO: least absolute shrinkage and selection operator; ES: exon skip. Figure S6: the tenfold cross-validation penalized LASSO logistic regression for ME. LASSO: least absolute shrinkage and selection operator; ME: mutually exclusive exon. Figure S7: the tenfold cross-validation penalized LASSO logistic regression for RI. LASSO: least absolute shrinkage and selection operator; RI: retained intron exons. Figure S8: details of prognosis signatures of AA. (A) The risk scores of KIRC patients' distribution basing on the median value. (B) The green dots mean survivors, and the red dots mean death cases. (C) The heat map shows the alteration of the percent spliced in value from low risk score to high risk score. AA: alternate acceptor site; KIRC: kidney renal clear cell carcinoma. Figure S9: details of prognosis signatures of AD. (A) The risk scores of KIRC patients' distribution basing on the median value. (B) The green dots mean survivors, and the red dots mean death cases. (C) The heat map shows the alteration of the percent spliced in value from low risk score to high risk score. AD: alternative donor site; KIRC: kidney renal clear cell carcinoma. Figure S10: details of prognosis signatures of AP. (A) The risk scores of KIRC pati [file 5576933.f1.zip › 5576933.f1/Figure S6 (1).jpg]

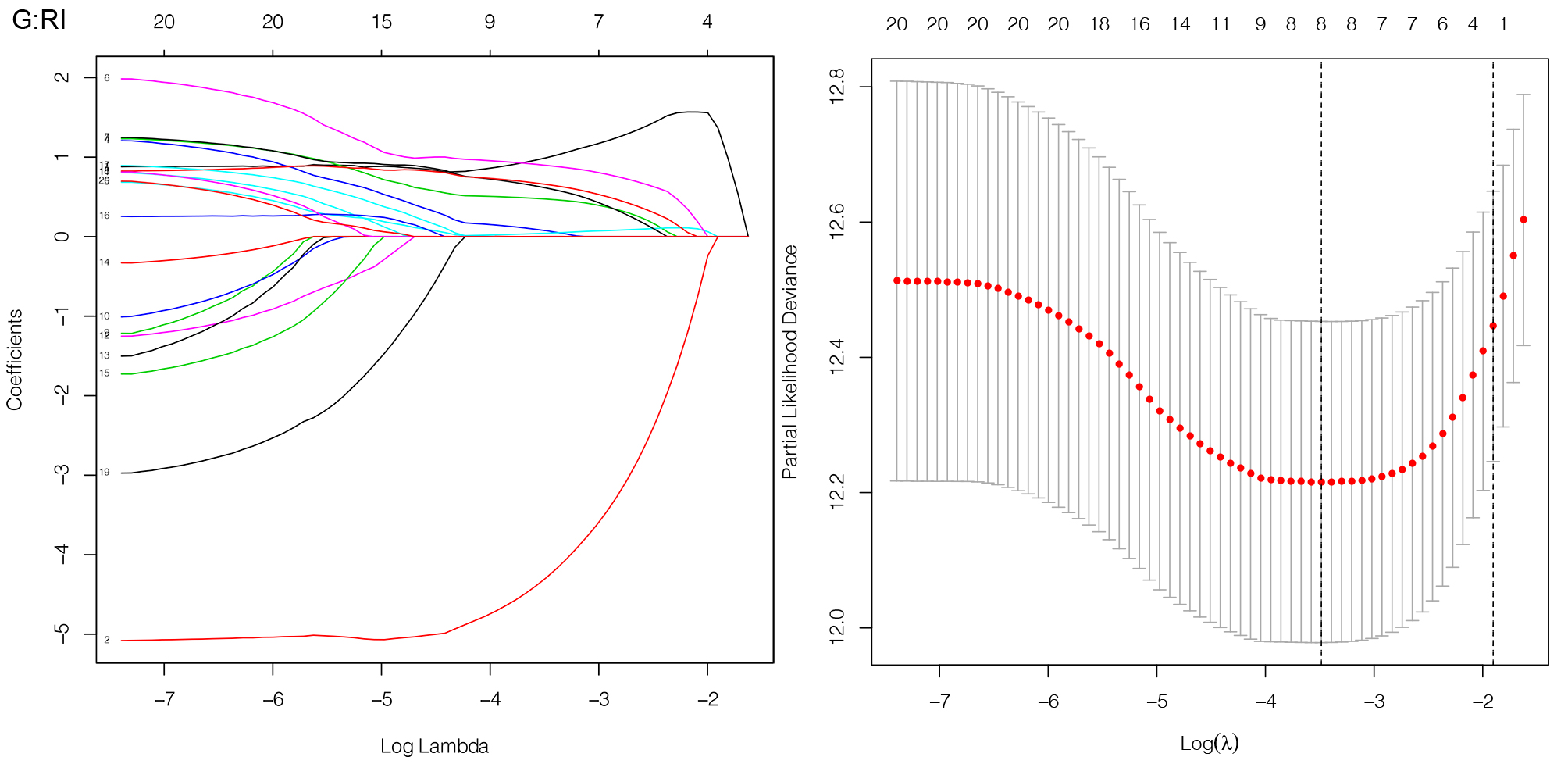

Supplement: Supplementary Materials — Figure S1: the tenfold cross-validation penalized LASSO logistic regression for AA. LASSO: least absolute shrinkage and selection operator; AA: alternate acceptor site. Figure S2: the tenfold cross-validation penalized LASSO logistic regression for AD. LASSO: least absolute shrinkage and selection operator; AD: alternative donor site. Figure S3: the tenfold cross-validation penalized LASSO logistic regression for AP. LASSO: least absolute shrinkage and selection operator; AP: alternate promoter. Figure S4: The tenfold cross-validation penalized LASSO logistic regression for AT. LASSO: least absolute shrinkage and selection operator; AT: alternative terminator. Figure S5: the tenfold cross-validation penalized LASSO logistic regression for ES. LASSO: least absolute shrinkage and selection operator; ES: exon skip. Figure S6: the tenfold cross-validation penalized LASSO logistic regression for ME. LASSO: least absolute shrinkage and selection operator; ME: mutually exclusive exon. Figure S7: the tenfold cross-validation penalized LASSO logistic regression for RI. LASSO: least absolute shrinkage and selection operator; RI: retained intron exons. Figure S8: details of prognosis signatures of AA. (A) The risk scores of KIRC patients' distribution basing on the median value. (B) The green dots mean survivors, and the red dots mean death cases. (C) The heat map shows the alteration of the percent spliced in value from low risk score to high risk score. AA: alternate acceptor site; KIRC: kidney renal clear cell carcinoma. Figure S9: details of prognosis signatures of AD. (A) The risk scores of KIRC patients' distribution basing on the median value. (B) The green dots mean survivors, and the red dots mean death cases. (C) The heat map shows the alteration of the percent spliced in value from low risk score to high risk score. AD: alternative donor site; KIRC: kidney renal clear cell carcinoma. Figure S10: details of prognosis signatures of AP. (A) The risk scores of KIRC pati [file 5576933.f1.zip › 5576933.f1/Figure S7 (1).jpg]

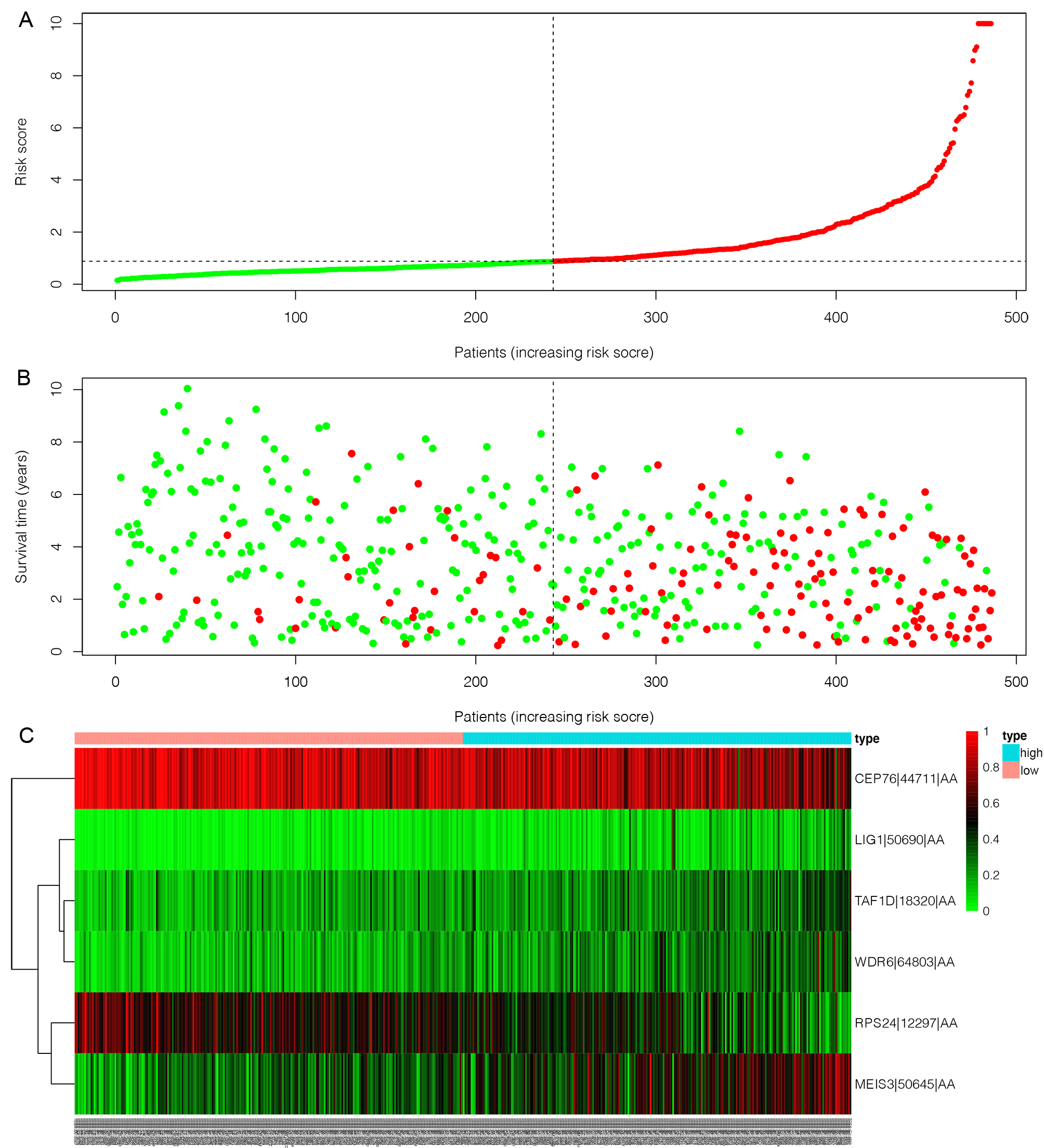

Supplement: Supplementary Materials — Figure S1: the tenfold cross-validation penalized LASSO logistic regression for AA. LASSO: least absolute shrinkage and selection operator; AA: alternate acceptor site. Figure S2: the tenfold cross-validation penalized LASSO logistic regression for AD. LASSO: least absolute shrinkage and selection operator; AD: alternative donor site. Figure S3: the tenfold cross-validation penalized LASSO logistic regression for AP. LASSO: least absolute shrinkage and selection operator; AP: alternate promoter. Figure S4: The tenfold cross-validation penalized LASSO logistic regression for AT. LASSO: least absolute shrinkage and selection operator; AT: alternative terminator. Figure S5: the tenfold cross-validation penalized LASSO logistic regression for ES. LASSO: least absolute shrinkage and selection operator; ES: exon skip. Figure S6: the tenfold cross-validation penalized LASSO logistic regression for ME. LASSO: least absolute shrinkage and selection operator; ME: mutually exclusive exon. Figure S7: the tenfold cross-validation penalized LASSO logistic regression for RI. LASSO: least absolute shrinkage and selection operator; RI: retained intron exons. Figure S8: details of prognosis signatures of AA. (A) The risk scores of KIRC patients' distribution basing on the median value. (B) The green dots mean survivors, and the red dots mean death cases. (C) The heat map shows the alteration of the percent spliced in value from low risk score to high risk score. AA: alternate acceptor site; KIRC: kidney renal clear cell carcinoma. Figure S9: details of prognosis signatures of AD. (A) The risk scores of KIRC patients' distribution basing on the median value. (B) The green dots mean survivors, and the red dots mean death cases. (C) The heat map shows the alteration of the percent spliced in value from low risk score to high risk score. AD: alternative donor site; KIRC: kidney renal clear cell carcinoma. Figure S10: details of prognosis signatures of AP. (A) The risk scores of KIRC pati [file 5576933.f1.zip › 5576933.f1/Figure S8 (1).jpg]

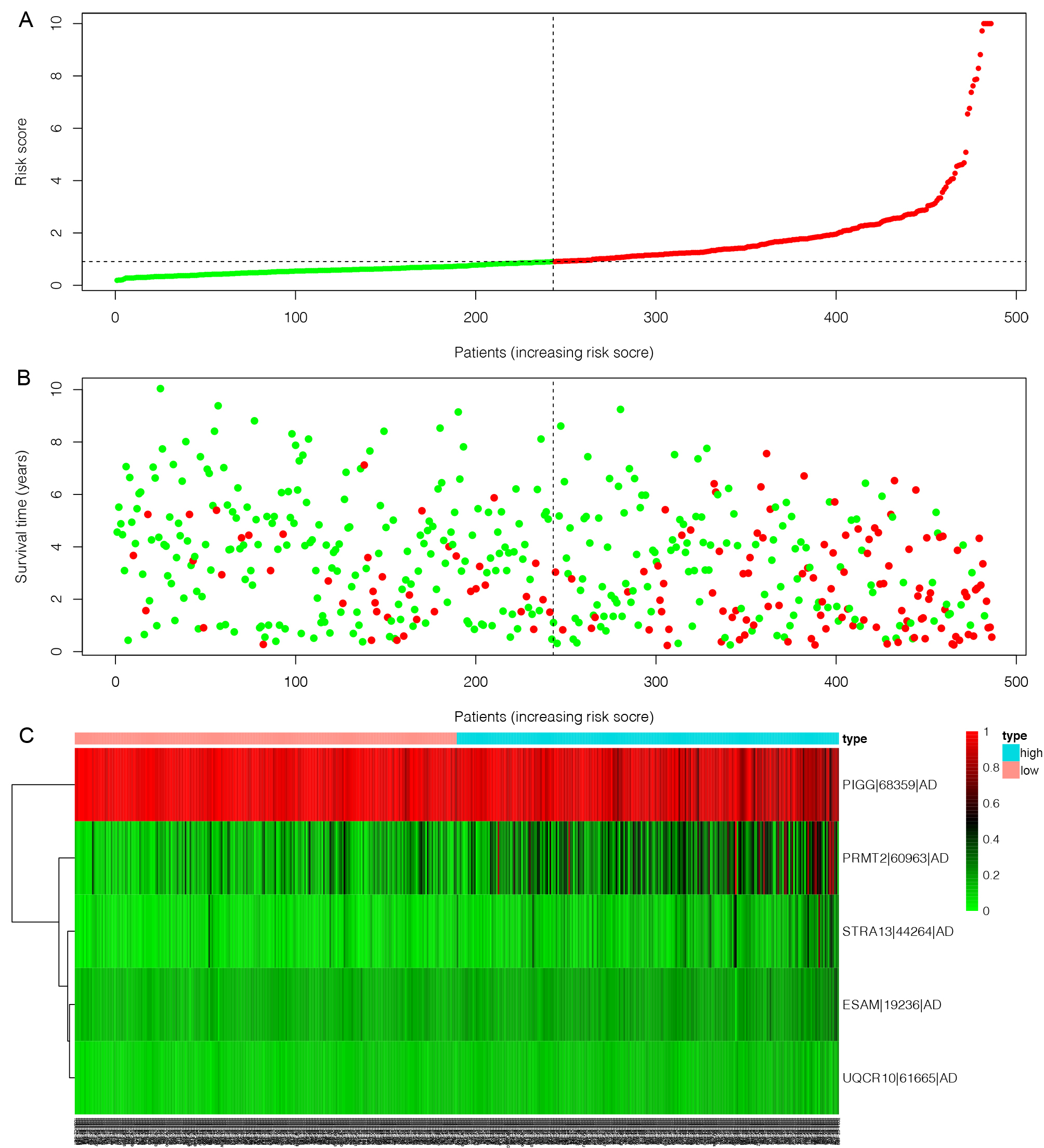

Supplement: Supplementary Materials — Figure S1: the tenfold cross-validation penalized LASSO logistic regression for AA. LASSO: least absolute shrinkage and selection operator; AA: alternate acceptor site. Figure S2: the tenfold cross-validation penalized LASSO logistic regression for AD. LASSO: least absolute shrinkage and selection operator; AD: alternative donor site. Figure S3: the tenfold cross-validation penalized LASSO logistic regression for AP. LASSO: least absolute shrinkage and selection operator; AP: alternate promoter. Figure S4: The tenfold cross-validation penalized LASSO logistic regression for AT. LASSO: least absolute shrinkage and selection operator; AT: alternative terminator. Figure S5: the tenfold cross-validation penalized LASSO logistic regression for ES. LASSO: least absolute shrinkage and selection operator; ES: exon skip. Figure S6: the tenfold cross-validation penalized LASSO logistic regression for ME. LASSO: least absolute shrinkage and selection operator; ME: mutually exclusive exon. Figure S7: the tenfold cross-validation penalized LASSO logistic regression for RI. LASSO: least absolute shrinkage and selection operator; RI: retained intron exons. Figure S8: details of prognosis signatures of AA. (A) The risk scores of KIRC patients' distribution basing on the median value. (B) The green dots mean survivors, and the red dots mean death cases. (C) The heat map shows the alteration of the percent spliced in value from low risk score to high risk score. AA: alternate acceptor site; KIRC: kidney renal clear cell carcinoma. Figure S9: details of prognosis signatures of AD. (A) The risk scores of KIRC patients' distribution basing on the median value. (B) The green dots mean survivors, and the red dots mean death cases. (C) The heat map shows the alteration of the percent spliced in value from low risk score to high risk score. AD: alternative donor site; KIRC: kidney renal clear cell carcinoma. Figure S10: details of prognosis signatures of AP. (A) The risk scores of KIRC pati [file 5576933.f1.zip › 5576933.f1/Figure S9 (1).jpg]
